# Balancing Histone Deacetylase (HDAC) Inhibition and Drug‐likeness: Biological and Physicochemical Evaluation of Class I Selective HDAC Inhibitors

**DOI:** 10.1002/cmdc.202100755

**Published:** 2022-02-18

**Authors:** Linda Schäker‐Hübner, Reza Haschemi, Thomas Büch, Fabian B. Kraft, Birke Brumme, Andrea Schöler, Robert Jenke, Jens Meiler, Achim Aigner, Gerd Bendas, Finn K. Hansen

**Affiliations:** ^1^ Institut für Wirkstoffentwicklung Medizinische Fakultät Universität Leipzig Brüderstraße 34 04103 Leipzig Germany; ^2^ Abteilung für Pharmazeutische und Zellbiologische Chemie Pharmazeutisches Institut Universität Bonn An der Immenburg 4 53121 Bonn Germany; ^3^ Rudolf-Boehm-Institut für Pharmakologie und Toxikologie, Klinische Pharmakologie Medizinische Fakultät Universität Leipzig Härtelstraße 16–18 04107 Leipzig Germany; ^4^ University Cancer Center Leipzig (UCCL) Universitätsklinikum Leipzig Liebigstraße 22, Haus 7 04103 Leipzig Germany

**Keywords:** cancer, drug design, epigenetics, histone deacetylases, inhibitors

## Abstract

Herein we report the structure‐activity and structure‐physicochemical property relationships of a series of class I selective *ortho*‐aminoanilides targeting the “foot‐pocket” in HDAC1&2. To balance the structural benefits and the physicochemical disadvantages of these substances, we started with a set of HDACi related to tacedinaline (CI‐994) and evaluated their solubility, lipophilicity (log D_7.4_) and inhibition of selected HDAC isoforms. Subsequently, we selected the most promising “capless” HDACi and transferred its ZBG to our previously published scaffold featuring a peptoid‐based cap group. The resulting hit compound **10 c** (**LSH‐A54)** showed favorable physicochemical properties and is a potent, selective HDAC1/2 inhibitor. The following evaluation of its slow binding properties revealed that **LSH‐A54** binds tightly to HDAC1 in an induced‐fit mechanism. The potent HDAC1/2 inhibitory properties were reflected by attenuated cell migration in a modified wound healing assay and reduced cell viability in a clonogenic survival assay in selected breast cancer cell lines.

## Graphical abstract

**In a fragment‐like approach** we selected a “foot pocket” unit (FPU) with favorable properties and transferred this FPU to our peptoid‐based HDACi scaffold (middle). The subsequently selected hit‐compound **10 c** (**LSH‐A54**) is a potent, slow‐binding inhibitor of HDAC1&2 with good selectivity over HDAC3 and shows a favorable physicochemical profile. Additional biological evaluations revealed promising anti‐cancer properties.

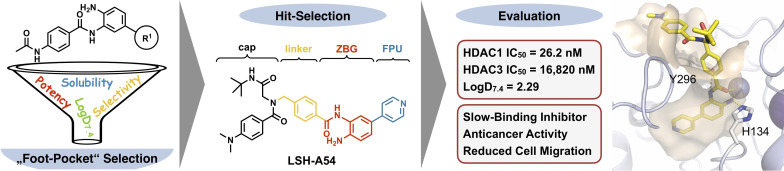

## Introduction

**Figure 1:**
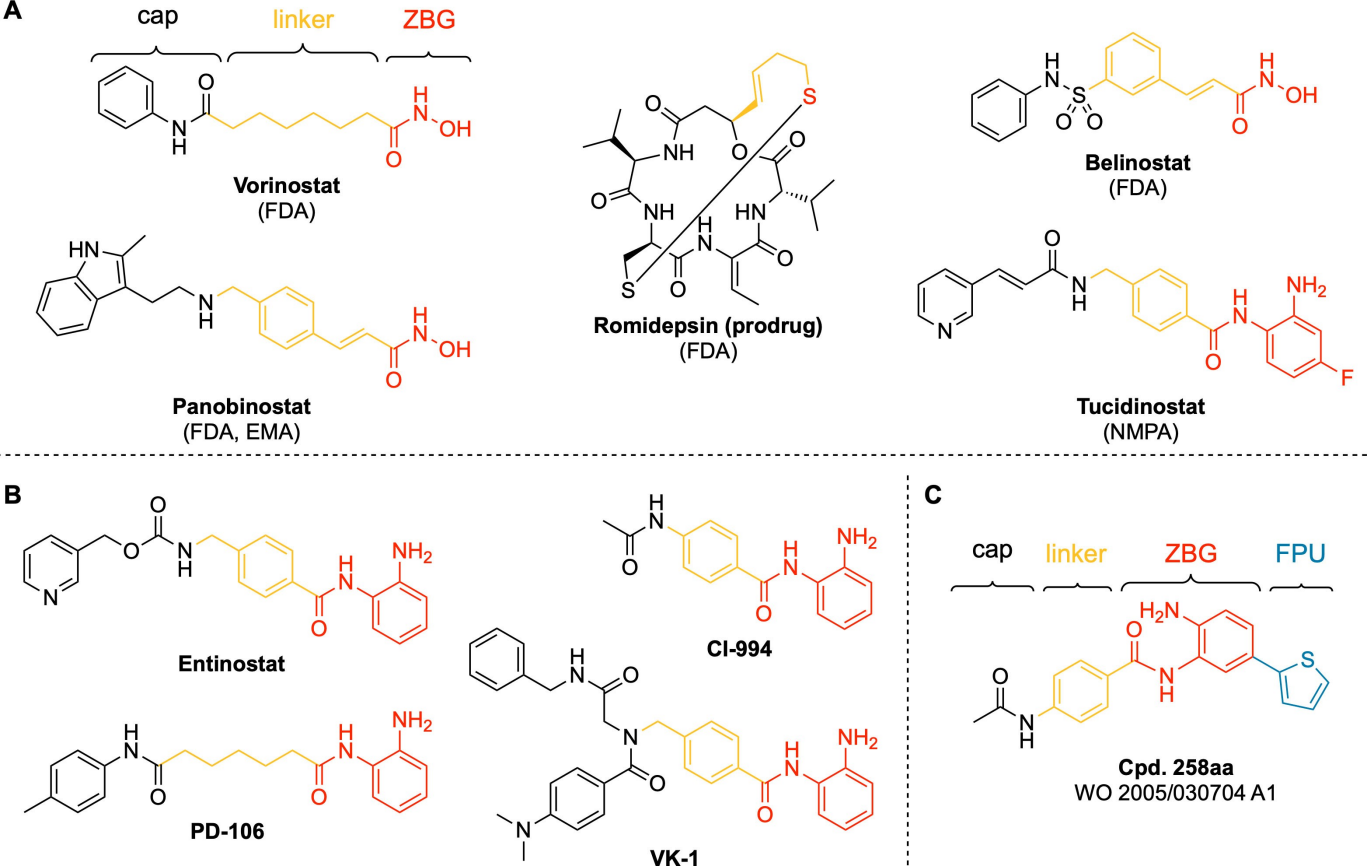
**A)** Structures of approved HDAC inhibitors: vorinostat (FDA; CTCL), romidepsin (FDA; CTCL, PTCL), belinostat (FDA, PTCL) and panobinostat (FDA, EMA; MM), tucidinostat (NMDA; PTCL, HR+ breast cancer);[^10^, ^14^] **B)** Selected class I selective HDACi. **C)** Cpd. 258 aa,^[19]^ hereafter referred to as “**6 c**”, is an example for a HDAC1/2 inhibitor derived from CI‐994 by introducing a 2‐thienyl‐substituent as “foot‐pocket” unit (FPU).

The role of epigenetic modifications and their irregularities in gene expression have been widely studied.[^1^, ^2^, ^3^] Epigenetic alterations often occur during the early stages of neoplastic growth and can lead to malignant tumors.^[4]^ More than 300 enzymes are known to modify the chromatin structure by adding (epigenetic writers), removing (epigenetic erasers) or reading (epigenetic readers) so‐called epigenetic marks and therefore defining the histone code.^[5]^ For instance, the equilibrium between histone lysine acetylation (euchromatin, transcriptionally active) and deacetylation (heterochromatin, transcriptionally repressed) is maintained by two families of enzymes: histone acetyl transferases (HATs; epigenetic writers) and histone deacetylases (HDACs; epigenetic erasers).^[5]^ The HDAC family encompasses eleven zinc‐dependent enzymes (HDAC1‐11) classified in four groups: class I, class IIa, class IIb, and class IV. Class I consists of HDAC 1, 2 and 3, which are nuclear enzymes, and HDAC8.^[5]^ Among others, HDAC1 and HDAC2 are associated with breast, prostate, gastric and hematopoietic cancers.[^5^, ^6^] Additionally, HDAC2 seems to be critically involved in cognitive processes like learning and memory^[7]^ and HDAC3 was shown to be an important factor in inflammation and neurodegenerative diseases.^[8]^ Inhibition of HDAC enzymes can result in various anti‐cancer effects[^4^, ^5^, ^9^, ^10^, ^11^, ^12^] and by now, the FDA‐approved HDAC inhibitors (HDACi) vorinostat, romidepsin, belinostat and panobinostat (Figure 1A) are established anti‐cancer drugs for the treatment of cutaneous and peripheral T‐cell lymphoma (CTCL & PTCL) as well as multiple myeloma (MM). More recently, the class I selective aminoanilide HDACi tucidinostat (chidamide) was approved for the treatment of HR+ breast cancer in combination with the aromatase inhibitor exemestane by the National Medical Products Administration of China (NMPA).[^13^, ^14^, ^15^] Notably, tucidinostat is the first HDACi that received regulatory approval for the treatment of a solid cancer.^[14]^

*ortho*‐Aminoanilides like tucidinostat as well as tacedinaline (CI‐994, Figure 1B) are HDAC class I selective inhibitors.^[16]^ From a structural point of view, the differences within class I isoforms are mainly based on the so‐called “foot‐pocket”, an internal, lipophilic cavity of the enzyme. Therefore, if the *o*‐aminoanilide group additionally bears a substituent such as a 2‐thienyl‐group (Figure 1C) to occupy the “foot‐pocket”, HDAC1/2 inhibition and selectivity is strongly increased.[^5^, ^17^, ^18^] However, introducing bulky, lipophilic substituents considerably increases molecular weight and lipophilicity and worsens water solubility. Therefore, HDACi that target the HDAC1/2 “foot‐pocket” are not necessarily drug‐like substances. Our group previously published a series of peptoid‐based aminoanilides as selective HDAC1‐3 inhibitors. The hit compounds from this study, **VK‐1** (in the publication referred to as Cpd. 2a, Figure 1B), demonstrated potent chemosensitizing properties and full reversal of cisplatin resistance in the human tongue squamous cell carcinoma cell line Cal27CisR.^[11]^ However, **VK‐1** revealed only limited aqueous solubility (0.98 μg/mL in PBS buffer, pH=7.4).

In this work, we aimed to balance the advantage of increased HDAC1/2 inhibition and selectivity over HDAC3 via targeting the “foot‐pocket” and the physicochemical disadvantages of these substances. We herein present the design, synthesis, structure‐activity, and structure‐physicochemical property relationships as well as biological evaluation of a small set of HDAC class I selective “capless” HDACi closely related to CI‐994 (Figure 1B) and three full‐sized inhibitors derived from **VK‐1**.

## Results and Discussion

**Scheme 1:**
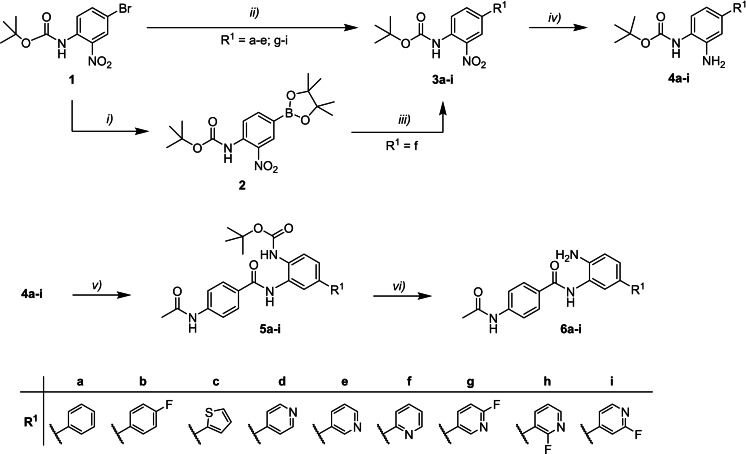
Synthesis of capless HDACi **6 a**–**i**. Reagents and conditions: *i)* **1** (1.0 equiv.), anhydrous KOAc (2.0 equiv.), PdCl_2_(dppf) (0.05 equiv.), bis(pinacolato)diboron (1.2 equiv.), anhydrous DMF (15 mL), 80 °C, 18 h, 33 %; *ii)* **1** (1.0 equiv.), R^1^‐B(OH)_2_ (1.1 equiv.), Pd(PPh_3_)_4_ (0.05 equiv.), toluene, Na_2_CO_3_ solution (2 M), EtOH, reflux, 16 h, 49–76 %; *iii)* **2** (1.0 equiv.), anhydrous Cs_2_CO_3_ (2.0 equiv.), PdCl_2_(dppf) (0.05 equiv.), 2‐bromopyridine (3.0 equiv.), anhydrous DMF, 85 °C, 16 h, 53 %; *iv)* **3 a**–**i** (1.0 equiv.), Pd(C) (5 %) (0.1 equiv.), H_2_, MeOH/DCM (75 : 25; *v/v*), rt, 2–16 h, 81 % quant.; *v)* 4‐acetamidobenzoic acid (1.2 equiv.), EDC ⋅ HCl (3.0 equiv.), HOBt ⋅ H_2_O (3.0 equiv.), DIPEA (5.0 equiv.), **4 a**–**i** (1.0 equiv.), dry DMF/dry DCM (67 : 33, *v/v*), rt, 16 h, 38–81 %; *vi)* **5 a**–**i** (1.0 equiv.), DCM/TFA (80 : 20; *v/v*), 0 °C→rt, 1–3 h, 62 % – quant.

**Design and synthesis of capless HDACi 6 a**–**i**. First, we designed a small set of capless HDACi based on CI‐994. To this end, we replaced the 2‐thienyl‐group of Cpd. 258aa (**6 c**) with a series of phenyl‐ or pyridinyl‐substituents. The synthesis of the capless HDACi **6 a**–**i** is summarized in Scheme 1. Starting from *tert*‐butyl (4‐bromo‐2‐nitrophenyl)‐carbamate (**1**), the Boc‐protected intermediates **3 a**–**e** and **3 g**–**i** were obtained via Suzuki reaction of **1** with the respective boronic acids utilizing Pd(PPh_3_)_4_ as catalyst. Due to the instability of pyridin‐2‐ylboronic acid, the intermediate *tert*‐butyl [2‐nitro‐4‐(pyridin‐2‐yl)phenyl]carbamate (**3 f**) was prepared over two steps. First, starting material **1** was borylated via miyaura borylation under basic conditions using PdCl_2_(dppf) as catalyst, yielding the dioxaborolan derivative **2**. Afterwards, compound **3 f** was obtained via Suzuki reaction of **2** with 2‐bromopyridine using PdCl_2_(dppf) in the presence of Cs_2_CO_3_. Next, the Boc‐protected phenylenediamines **4 a**–**i** were generated from the respective 2‐nitrophenyl derivatives **3 a**–**i** via hydrogenation using palladium on activated charcoal. The 2‐aminophenyl derivatives **4 a**–**i** were then coupled with 4‐acetamidobenzoic acid using EDC ⋅ HCl and HOBt ⋅ H_2_O as coupling reagents, yielding the intermediates **5 a**–**i**. Finally, Boc‐deprotection under acidic conditions gave the CI‐994‐like capless HDACi **6 a**–**i**.

All capless HDACi **6 a**–**i** were tested for their inhibitory activity against HDAC1, HDAC2, HDAC3 and HDAC6 using CI‐994 and vorinostat as controls. *ortho*‐Aminoanilides are slow‐on/slow‐off binding HDACi.^[20]^ Consequently, in order to get a more reliable impression on the selectivity profile of the capless HDACi, we decided to modify our previously reported *in vitro* HDAC inhibition assay:^[21]^ inhibitors and enzymes were preincubated prior to the addition of the fluorogenic substrate ZMAL (see Supporting Information (SI)) as was previously described in literature.[^16^, ^17^, ^20^, ^22^, ^23^] We also tested the lipophilicity (log D_7.4_) and aqueous solubility of these compounds to evaluate not only the structure‐activity, but also the structure‐physicochemical property relationships of compounds **6 a**–**i** compared to CI‐994 and vorinostat. Predictive software to estimate the lipophilicity of molecules are key tools in drug discovery with generally acceptable accuracy. However, the quality of these results is heavily influenced by the structural similarity of the compounds in question to the training set and/or the quality of the underlying data set.^[24]^ Considering this, we decided to determine lipophilicity and solubility experimentally, using the slow‐stirring method[^25^, ^26^] (lipophilicity) and the flask method^[27]^ (solubility) in combination with quantification via HPLC. The results are summarized in Table 1.

**Table 1:** Inhibitory activity against HDAC1‐3 and 6, and physicochemical properties of capless HDACi **6 a**–**i**.

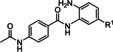							
	R^1^	Physicochemical Properties		IC_50_ [nM]			
		LogD_7.4_	Solubility^[a,e]^ [μg/mL]	HDAC1^[b,e]^	HDAC2^[b,e]^	HDAC3^[b,e]^	HDAC6^[c,e]^
CI‐994	‐H	0.84	200±1.80	636.3±114.3	696.3±10.5	262.6±30.7	>10,000^[d]^
**6 a**	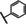	2.63	1.49±0.14	4.50±0.1	51.4±4.9	3,960±58.5	>10,000^[d]^
**6 b**	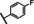	3.41	0.45±0.04	4.40±0.1	44.7±6.9	1,998±33.0	>10,000^[d]^
**6 c**	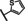	2.80	0.74±0.01	4.50±0.002	31.6±0.3	1,424±40.5	>10,000^[d]^
**6 d**	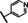	1.75	16.5±0.30	13.2±1.0	77.2±9.2	8,908±624.0	>10,000^[d]^
**6 e**	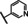	1.70	37.7±3.61	76.7 ± 9.2	100.5±5.4	635.8±49.5	>10,000^[d]^
**6 f**	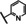	1.77	42.0±0.19	207.3±4.4	333.3±5.1	17,350±2,420	>10,000^[d]^
**6 g**	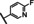	1.97	7.66±0.08	93.5±2.4	118.3±4.0	767.5±31.7	>10,000^[d]^
**6 h**	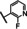	1.79	1.58±0.14	153.2±22.1	227.6±7.8	2,873±523.0	>10,000^[d]^
**6 i**	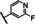	2.20	3.72±0.44	329.4±20.8	211.3 ±5.4	5,867±23.2	>10,000^[d]^
vorinostat	–	0.99	255±3.71	113.0±16.9	191.4±15.4	109.5±9.7	21.7±3.2

[a] PBS buffer, pH=7.4; [b] preincubation of enzyme and inhibitor 1 h at 25 °C; [c] preincubation of enzyme and inhibitor 15 min at 25 °C; [d] <25 % inhibition at stated concentration, [e] mean ±SD, at least two independent experiments.

As expected, all capless HDACi showed increased inhibitory activity against HDAC1/2 compared to CI‐994 and in contrast to CI‐994 preference over HDAC3. All *ortho*‐aminoanilides, including CI‐994 showed no inhibition of HDAC6. The three most lipophilic compounds **6 a**–**c** (logD_7.4_ values ranging from 2.63 to 3.41), bearing a phenyl‐ (**6 a**), 4‐fluorphenyl‐ (**6 b**) and a 2‐thienyl‐group (**6 c**) as “foot‐pocket” unit (FPU), showed very similar inhibitory activity against HDAC1 (IC_50_ values ranging from 4.4 to 4.5 nM) and HDAC2 (IC_50_ values ranging from 31.6 to 51.4 nM) as well as micromolar inhibitory activity at HDAC3 leading at least to a 312‐fold selectivity for HDAC1 over HDAC3. However, the strongly increased lipophilicity of these compounds also led to an approximately 100‐fold reduced solubility compared to CI‐994. In contrast, compounds **6 d**–**f**, bearing a 4‐pyridinyl‐ (**6 d**), 3‐pyridinyl‐ (**6 e**) and 2‐pyridinyl‐group (**6 f**) as FPU, demonstrated overall a more favorable physicochemical profile. With logD_7.4_ values ranging from 1.70 to 1.77, these compounds show moderate lipophilicity and, more importantly, the solubility is considerably higher compared to **6 a**–**c** (see Table 1). Interestingly, the position of the nitrogen in the pyridinyl‐group greatly effects HDAC inhibition and therefore determines HDAC1/2 selectivity over HDAC3: Compared to **6 a**, the 2‐pyridinyl FPU of **6 f** led to a generally diminished HDAC inhibition. Furthermore, the 3‐pyridinyl FPU of **6 e** did not only deteriorate HDAC1/2 activity, but it also resulted in improved HDAC3 inhibitory activity compared to **6 a**. Therefore, the HDAC1/2 selectivity over HDAC3 that inherently comes along with the introduction of a FPU to *ortho*‐aminoanilides was nearly leveled out. In terms of HDAC1/2 inhibition and selectivity for HDAC1 over HDAC3 (675‐fold), the 4‐pyridinyl‐group of **6 d** displayed the best results in the pyridinyl series (HDAC1 IC_50_: 13.2 nM; HDAC2 IC_50_: 77.2 nM; HDAC3 IC_50_: 8,908 nM). Compounds **6 g**–**i** bear fluoropyridinyl substituents as FPU. Overall, these compounds show somewhat increased lipophilicity (logD_7.4_ values ranging from 1.79 to 2.20) and decreased solubility compared to **6 d**–**f**. In fact, the solubility, or lack thereof, of the fluoropyridinyl compounds **6 g**–**i** is comparable to compounds **6 a**–**c**. Given the hydrophobic character of the HDAC1/2 “foot‐pocket” compounds **6 g**–**i** showed surprisingly low HDAC inhibition. With IC_50_ values ranging from 93.5 to 329.4 nM (HDAC1) and from 118.3 to 227.6 nM (HDAC2) respectively, compounds **6 g**–**i** demonstrated overall diminished HDAC1/2 activity compared to the related pyridinyl‐derivatives **6 d** or **6 e**. Interestingly, depending on the position of the introduced fluorine, HDAC3 inhibition can either be improved (compare **6 d** vs. **6 i**) or reduced (compare **6 e** vs. **6 h**). Taken together, highly lipophilic FPUs are preferred over hydrophilic FPUs and high lipophilicity is usually accompanied with low solubility and *vice versa*. However, there is one exception in our set of capless HDACi: The moderately lipophilic 4‐pyridinyl‐group of **6 d** showed acceptable solubility, seems to be well tolerated by HDAC1/2 and preserves selectivity over HDAC3. Given the only slightly diminished HDAC1/2 inhibitory activity and the physicochemical advantages of **6 d** over **6 a**–**c**, we picked the 4‐pyridinyl‐group as the most promising FPU to design full‐sized HDACi with peptoid‐based cap groups.

**Figure 2:**
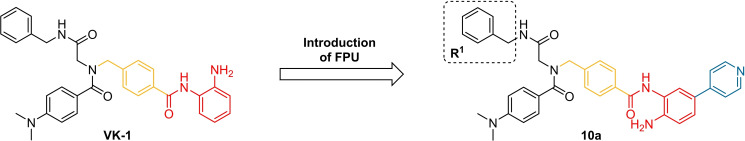
The introduction of the 4‐pyridinyl FPU to **VK‐1** leads to compound **10 a**. Compounds **10 b** & **10 c** originate from variations of the “isocyanide region” (labeled “R^1^”).

**Design and synthesis of full‐sized inhibitors 10 a**–**c**. The design of the full‐sized inhibitors **10 a**–**c** is summarized in Figure 2. For the full‐sized HDACi **10 a**–**c** we introduced the 4‐pyridinyl FPU of **6 d** to our previously published peptoid scaffold[^11^, ^12^, ^28^] which can be prepared via the ugi multi‐component reaction. As mentioned above, the most promising compound in the series reported by Krieger & Hamacher *et al*.^[11]^ was **VK‐1**. However, even without the additional molecular weight of a FPU **VK‐1** displayed high lipophilicity and low solubility (Table 2). Thus, in addition to the introduction of the 4‐pyridinyl FPU, we decided to vary the “isocyanide region” (labeled “R^1^” in Figure 2) of the scaffold, aiming for less molecular weight and a more favorable physicochemical profile.

**Table 2:** HDAC inhibition and physicochemical properties of full‐sized inhibitors **10 a**–**c**.

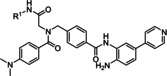							
	R^1^	Physicochemical Properties		IC_50_ [nM]			
		LogD_7.4_	Solubility^[a,e]^ [μg/mL]	HDAC1^[b,e]^	HDAC2^[b,e]^	HDAC3^[b,e]^	HDAC6^[c,e]^
**10 a**	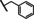	3.16	0.21±0.03	41.8±0.2	100.2±2.6	>6,666^[d]^	>10,000^[d]^
**10 b**	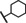	2.68	1.99±0.04	41.1±3.7	85.5±11.6	>3,333^[d]^	>10,000^[d]^
**10 c**	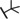	2.29	7.48±0.33	26.2±0.9	59.3±7.9	16,820±2,515	>10,000^[d]^
VK‐1	–	3.19	0.98±0.10	68.6±10.4	128.2±14.5	310.4±7.8	>10,000^[d]^
entinostat	–	n.d.	n.d.	426.1±58.7	354.0±43.1	310.5±9.8	>10,000^[d]^
vorinostat	–	0.99	255±3.71	113.0±16.9	191.4±15.4	109.5±9.7	21.7±3.2

[a] PBS buffer, pH=7.4; [b] preincubation of enzyme and inhibitor 1 h at 25 °C; [c] preincubation of enzyme and inhibitor 15 min at 25 °C; [d] <25 % inhibition at stated concentration; n.d.: not determined, [e] mean ±SD, at least two independent experiments.

**Scheme 2:**
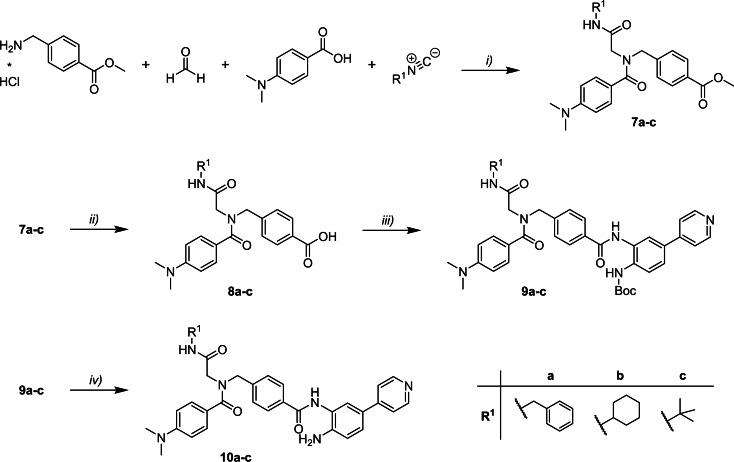
Synthesis of full‐sized inhibitors **6 a**–**i**. Reagents and conditions: *i)* methyl 4‐(aminomethyl)benzoate hydrochloride (1.2 equiv.), paraformaldehyde (1.2 equiv.), TEA (1.2 equiv.), 4‐(dimethylamino)‐benzoic acid (1.0 equiv.), respective isocyanide (1.0 equiv.), MeOH, 4 Å MS, rt, 72 h, 58–84 %; *ii)* methyl benzoate **7 a**–**c** (1.0 equiv.), NaOH‐solution (50 mg/mL, 2.5 equiv.), THF/MeOH (90 : 10; *v/v*), 40 °C, 2–24 h, 69–98 %; *iii)* **8 a**–**c** (1.2 equiv.), EDC ⋅ HCl (3.0 equiv.), HOBt ⋅ H_2_O (3.0 equiv.), DIPEA (5.0 equiv.), **4 d** (1.0 equiv.), dry DMF/dry DCM (67 : 33, *v/v*), rt, 16 h, 55–61 %; *iv)* **9 a**–**c** (1.0 equiv.), DCM/TFA (80 : 20; *v/v*), 0 °C→rt, 1–3 h, 93 % – quant.

The synthesis of compounds **10 a**–**c** is summarized in Scheme 2. The peptoid scaffold of **7 a**–**c** was prepared via ugi multi‐component reaction using methyl 4‐(aminomethyl)benzoate hydrochloride, paraformaldehyde as formaldehyde source, 4‐(dimethylamino)benzoic acid and the respective isocyanide. Next, the methyl esters of **7 a**–**c** were saponified. The resulting carboxylic acids **8 a**–**c** were coupled with the Boc‐protected ZBG **4 d**. Deprotection of the resulting Boc‐protected intermediates **9 a**–**c** under acidic conditions yielded the final products **10 a**–**c**.

**Figure 3:**
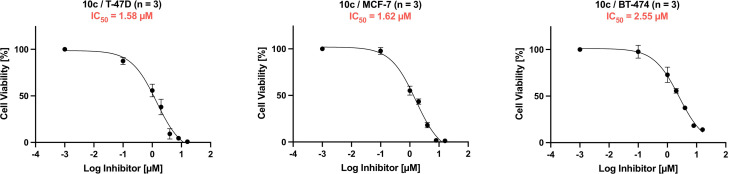
Representative dose response curves of **10 c** (mean ±SEM plotted from three independent experiments) in the breast cancer cell lines T‐47D, MCF‐7, and BT‐474. For representative dose response curves of **6 d**, **10 a**, **10 b**, entinostat, and vorinostat: see Supporting Information (SI) Figure S1–S3.

First, the full‐sized inhibitors and the reference substances **VK‐1** and entinostat were evaluated regarding their activity against HDAC1, HDAC2, HDAC3 and HDAC6 as well as their physicochemical properties. The results are summarized in Table 2. Fortunately, the introduction of the 4‐pyridinyl FPU was well tolerated in terms of lipophilicity and solubility. Compared to **VK‐1** the logD_7.4_ and solubility remained stable (compare **VK‐1** vs. **10 a**), confirming the favorable physicochemical properties of this FPU. Combined with the variations in the “isocyanide region” (**10 b**, **10 c**) we were able to considerably improve the solubility of these compounds and lower the logD_7.4_ to a moderate range (**10 b**: 2.68; **10 c**: 2.29). Interestingly, the introduction of the FPU had much less effect on HDAC1/2 inhibition in this scaffold than in the case of the capless HDACi. The HDAC1 IC_50_ values of **10 a**–**c** are very similar and range from 26.2 nM to 41.8 nM. HDAC2 IC_50_ values are also comparable, ranging from 59.3 nM to 89.1 nM. However, it seems that the less demanding cap group of **10 c** has a positive effect on HDAC1/2 inhibition. Given the slow‐binding characteristics of *ortho*‐aminoanilides, the additional FPU and the sterically demanding cap group, we suspected an even slower binding behavior of **10 a**–**c** compared to **6 d** and **VK‐1**, leading to less HDAC1/2 IC_50_ improvement than expected. The most notable differences within the full‐sized inhibitors can be observed in terms of HDAC3 inhibition. For compounds **10 a** and **10 b** we were not able to determine an IC_50_ value at HDAC3 due to insufficient solubility of these compounds. This agrees with our findings regarding HDAC1/2 inhibition and physicochemical properties, where we also found the *tert*‐butyl group of **10 c** to be favorable over the benzyl or cyclohexyl group in the “isocyanide region”. Furthermore, to get a first impression of the anti‐cancer properties of the full‐sized inhibitors we screened **10 a**‐**c** and capless HDACi **6 d** in a WST‐8 assay in three different breast cancer cell lines (T‐47D, MCF‐7, and BT‐474). The results are summarized in Table 3 and Figure 3. Compounds **10 a**–**c** displayed similar antiproliferative properties in all cell lines. Compared to entinostat (HDAC1‐3 selective) and vorinostat (pan‐HDACi), the HDAC1/2 selective inhibitors **10 a**–**c** showed comparable or enhanced antiproliferative properties. Notably, and we suspect mostly due to superior physicochemical properties, **10 c** outperformed **10 a** in all cell lines and **10 b** in two of three cases. Interestingly, the results for **6 d** were less consistent. In fact, the IC_50_ values for capless HDACi **6 d** range from IC_50_=1.01 μM (T‐47D; see Table 3) to IC_50_=11.13 μM (MCF‐7; see Table 3). Therefore, based on HDAC inhibition data, physicochemical and antiproliferative properties, we selected compound **10 c** along with **6 d** for further analysis regarding its binding kinetics, binding mode, and biological properties.

**Table 3:** IC_50_ values of selected compounds against different breast cancer entities including standard deviation (n=3).

	IC_50_ [μM]		
	T‐47D^[a,b]^	MCF‐7^[a,b]^	BT‐474^[a,c,d]^
**6 d**	1.01±0.19	11.13±3.85	>8.00^[e]^
**10 a**	2.57±0.93	3.24±0.82	3.93±0.56
**10 b**	1.45±0.09	4.33±1.54	2.93±0.43
**10 c**	1.58±0.48	1.62±0.32	2.55±0.40
entinostat	2.91±0.89	8.98±3.00	7.09±1.00
vorinostat	3.17±0.39	4.55±1.07	10.13±3.47

[a] invasive ductal carcinoma, not otherwise specified; [b] HR+ (hormone receptor positive); [c] PR+ (progesterone receptor positive); [d] HER2 overexpression; [e] <25 % inhibition at stated concentration

**IC_50_‐shift experiments and binding kinetics of selected inhibitors**. To get a first impression on the binding behavior of **6 d** and **10 c** we evaluated the effect of preincubation time on the observed IC_50_ values, as was previously reported, among others by C. James Chou and co‐workers.[^20^, ^22^] As we aimed to study the additional effects of the FPU on the binding mode of our inhibitors, we choose to include entinostat and **VK‐1** (see SI, Figure S4) as reference compounds.

**Figure 4:**
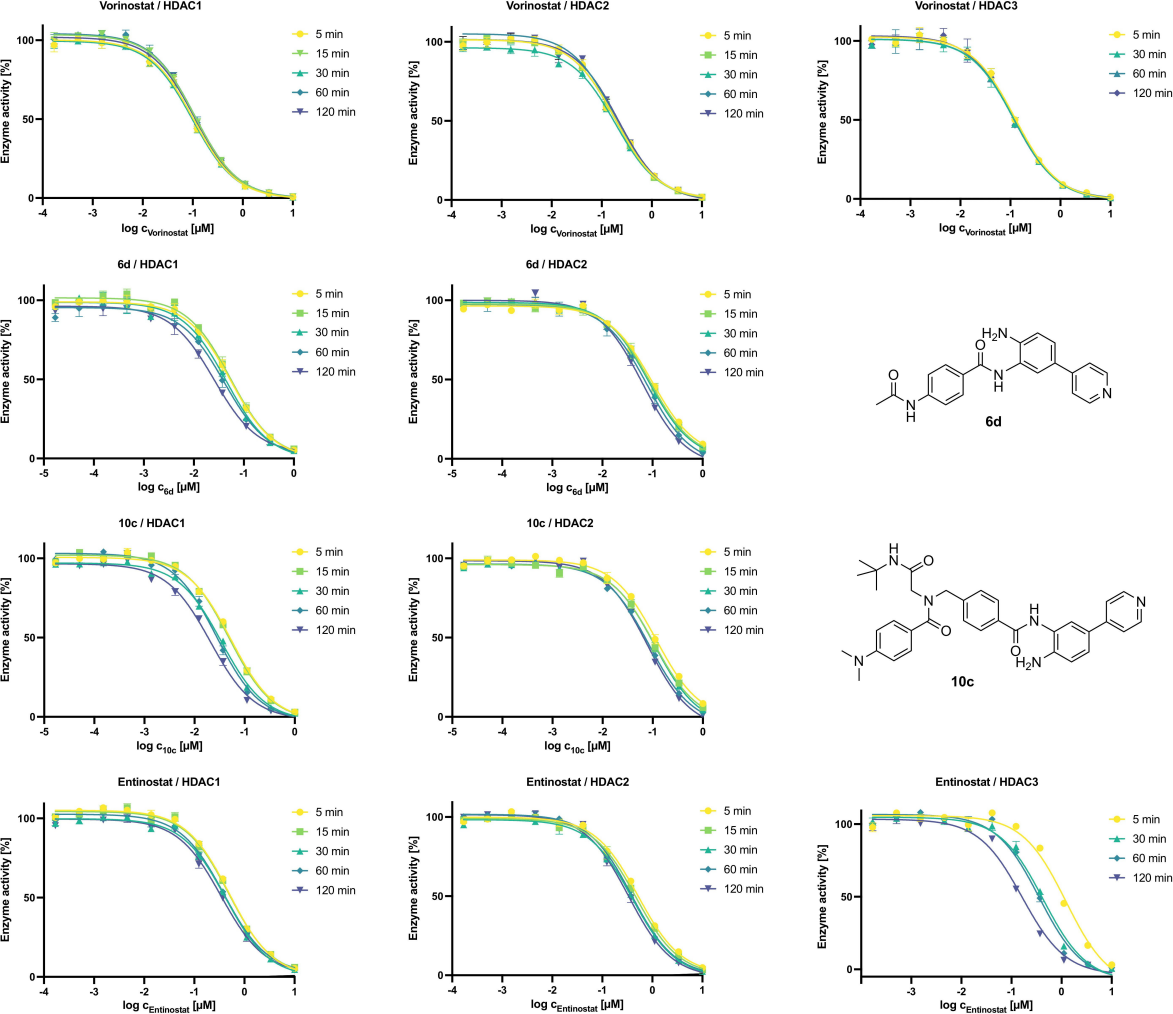
Dependence of preincubation time (time as indicated, for details see experimental procedure) and IC_50_ values for selected inhibitors and HDAC isoforms. Experiments were performed in triplicates, for IC_50_ values including P95 confidence intervals see SI, Table S1–S3.

**Figure 5:**
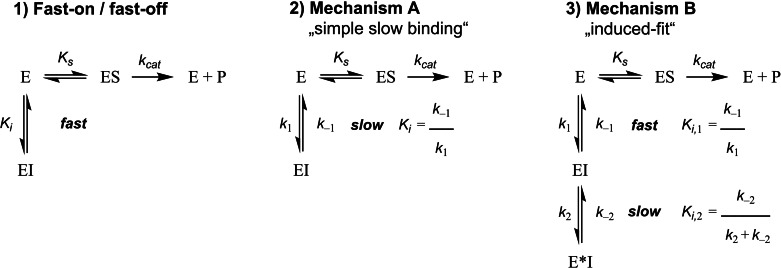
Representative examples of different kinetic mechanisms of enzyme inhibition, including the relationships between the respective association and dissociation rate constants (e. g., *k*_1_ & *k*_−1_) and the related equilibrium dissociation constant *K_i_*. **A**) Fast‐on/fast‐off binding kinetics. For competitive fast‐on/fast‐off inhibitors the half maximal inhibitory concentration (IC_50_) and the *K_i_* are directly related by the Cheng‐Prusoff equation;^[34]^ **B**) slow‐binding Mechanism I: single‐step slow‐binding, *k*_1_ & *k*_−1_ are inherently slow; **C**) slow‐binding Mechanism II: two‐step slow‐binding or “induced‐fit”. Initially, inhibitor and enzyme form an encounter complex [EI] that subsequently slowly undergoes isomerization to a binary enzyme inhibitor complex [E*I].

The results are summarized in Figure 4 (for IC_50_ values see SI, Table S1–S3). As expected, HDAC1‐3 IC_50_ values for vorinostat (top row) do not depend on preincubation time. Although, the effect is more distinct at HDAC1, both the capless inhibitor **6 d** (second row) and the full‐sized inhibitor **10 c** (third row) show a notable dependence between preincubation time and the IC_50_ values at HDAC1/2. The same effect is evident for entinostat. As expected, a similar behavior was observed for **VK‐1** (see SI, Figure S4), however, especially at HDAC1 it is far less pronounced than for the FPU bearing inhibitors **6 d** and **10 c**. This strong IC_50_‐shift depending on the preincubation time exceeds the HDAC1/2 IC_50_‐shift of entinostat and **VK‐1** by far, confirming our earlier suspicion that the introduction of the FPU additionally slows the binding of these inhibitors towards HDAC1/2. The impressive IC_50_‐shift entinostat (and **VK‐1**, see SI, Figure S4) showed at HDAC3 was previously described for *ortho*‐aminoanilide HDACi PD‐106 by Chou *et al.*.^[20]^ Following the IC_50_‐shift experiments we determined the slow‐binding characteristics of **6 d** and **10 c** using the *Progression‐Method* (see SI) described by Morrison and Walsh and other research groups.[^20^, ^29^, ^30^] There are several possible slow‐binding mechanisms.[^31^, ^32^, ^33^] The most common types “simple slow‐binding” (Mechanism I) and “induced‐fit” (Mechanism II) are described in Figure 5.

To determine the binding mechanism at the respective HDAC isoform, a series of progression curves were generated, using fixed concentrations of enzyme, substrate, and different inhibitor concentrations. The *in situ* AMC release was monitored continuously by fluorescence readings recorded every 30 s for 60 or 90 min at 37 °C. The data of each progression curve were fitted to the appropriate equation (Prism 9.1.0 for MacOS). For inhibitors showing slow‐binding Mechanism I&II (Figure 5) time‐dependent product formation follows Eq. 1:

(Eq. 1) $$\left[P\right]=v_{ss}t+\frac{v_{in}-v_{ss}}{k_{obs}}\left({1-e}^{-k_{obs}t}\right)$$

where [P] is the amount of generated AMC, which directly correlates with the amount of deacetylated substrate over time [*t*]. *V*_in_ and *v*_ss_ are the initial and final steady‐state velocities of product formation and *k*_obs_ is the apparat first‐order rate constant for the conversion from the initial velocity to the steady‐state velocity. It characterizes the formation of an enzyme–inhibitor equilibrium.

**Fast‐on/fast‐off inhibitors**. For competitive inhibitors like vorinostat with fast‐on / fast‐off binding behavior the enzyme–inhibitor equilibrium and therefore steady‐state velocity is rapidly reached. In this case Eq. 1 simplifies as follows:

(Eq. 2) $$\left[P\right]=v_{ss}t$$

Therefore, the deacetylation rate in absence (*v*_0_) and presence (*v*_i_) of the inhibitor is constant over time. In the case of substrate excess (e. g. [S]=5 times *K*_m_) the equilibrium dissociation constant *K_i_* can then be determined by plotting the ratio *v*_i_/*v*_0_ against the corresponding inhibitor concentration^[35]^ (see Eq. 3). [I] and [S] are the inhibitor and substrate concentrations, respectively and *K*_m_ is the Michaelis‐Menten constant.

(Eq. 3) $$\frac{v_{i}}{v_{0}}=\frac{1}{\frac{\left[I\right]}{K_{i}\left(1+\frac{\left[S\right]}{K_{m}}\right)}+1}$$

**Figure 6:**
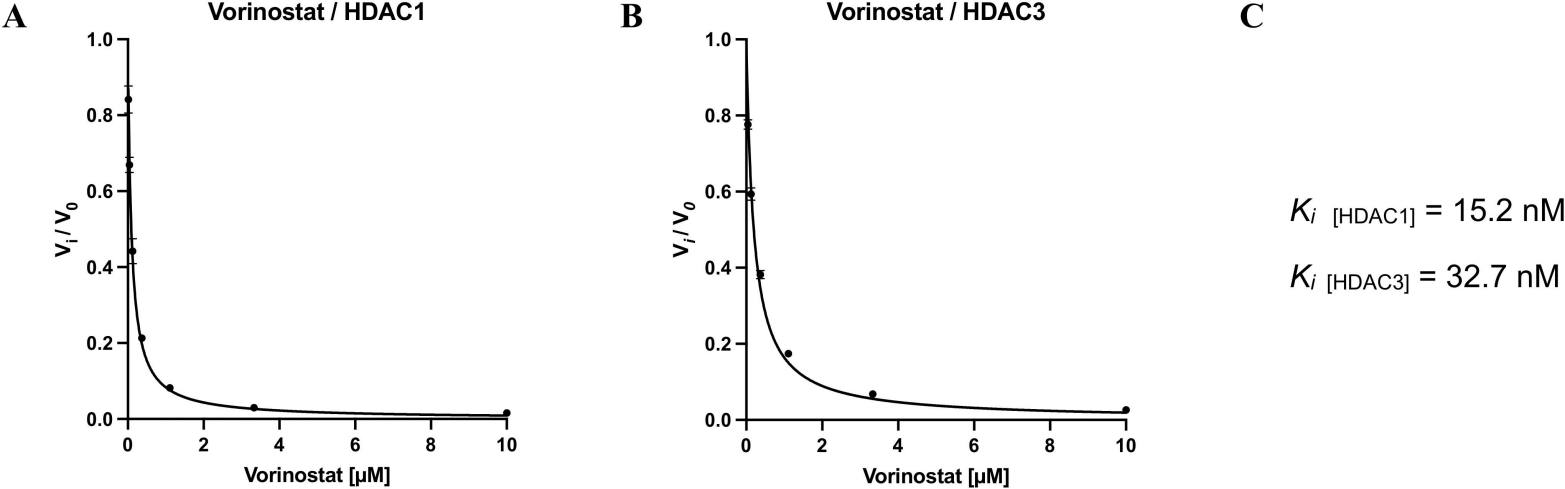
Vorinostat inhibition at HDAC1 and HDAC3. The ratio of the steady‐state velocities $v_{i}/v_{0}$ (mean ±SD) was plotted against the corresponding inhibitor concentrations. To determine the *K_i_* values the resulting curves were fitted to Eq. 3. **A**) $v_{i}/v_{0}$ ratio of HDAC1/vorinostat. **B**) $v_{i}/v_{0}$ ratio of HDAC3/vorinostat. **C**) Calculated *K_i_* values for vorinostat at HDAC1 and HDAC3. Experiments were performed in triplicates.

The results confirmed the expected fast‐on/fast‐off binding kinetic of vorinostat and are summarized in Figure 6.

**Slow‐binding inhibitors**. The slow‐binding Mechanisms I & II are distinguished by their respective relationships between *k*_obs_ and the inhibitor concentration. For the single‐step slow‐binding Mechanism I the relationship between inhibitor concentration and *k*_obs_ is linear. The association and dissociation rate constants (*k*_1_ & *k*_−1_, see Figure 5) can be obtained by fitting *k*_obs_ as a function of [I] to Eq. 4.

(Eq. 4) $$k_{obs}=k_{-1}+k_{1}\left(1+\frac{\left[S\right]}{K_{m}}\right)\left[I\right]$$

In contrast, for the two‐step slow‐binding Mechanism II, the so‐called “induced‐fit”, the relationship between inhibitor concentration and *k*_obs_ is hyperbolic. The secondary association and dissociation rate constants *k*_2_ & *k*_−2_ (see Figure 5) as well as the equilibrium dissociation constant *K*_*i*,1_ of the initial inhibitor‐enzyme encounter complex [EI] can be obtained by fitting *k*_obs_ as a function of [I] (Eq. 5.

(Eq. 5) $$k_{obs}=k_{-2}+\frac{k_{2}}{\left[I\right]+K_{i,1}\left(1+\frac{\left[S\right]}{K_{m}}\right)}\left[I\right]$$

**Figure 7:**
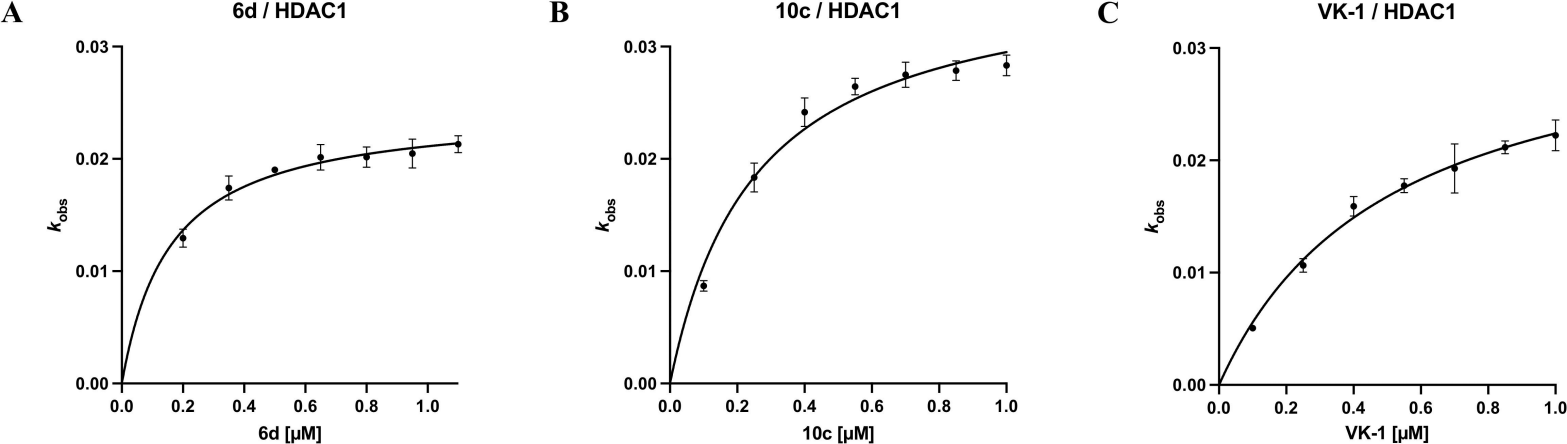
Slow‐binding at HDAC1: The apparent first‐order rate constant *k*_obs_ (mean ±SD) plotted against the corresponding inhibitor concentrations [I]. The resulting curves were fitted to Eq. 5. **A) 6 d**, the relationship between *k*_obs_ and [I] indicates slow‐binding Mechanism II; **B) 10 c**, the relationship between *k*_obs_ and [I] indicates slow‐binding Mechanism II; **C) VK‐1**, the relationship between *k*_obs_ and [I] indicates slow‐binding Mechanism II. Experiments were performed in triplicates.

The results for capless HDACi **6 d** and full‐sized inhibitor **10 c** are summarized in Figure 7. To investigate the effects of the 4‐pyridinyl FPU and the peptoid‐based cap group on the slow‐binding mechanism of the respective inhibitor at HDAC1 we included **VK‐1** (influence of the peptoid‐based cap group; no FPU) in our experiments.

For **6 d** and **10 c** the relationship between *k*_obs_ and inhibitor concentration is hyperbolic, indicating slow‐binding Mechanism II (also described as “induced‐fit”). This binding mechanism was previously reported for other HDACi bearing a FPU.^[36]^ However, the two compounds lacking a FPU, **VK‐1** and entinostat (see SI, Figure S6) also show a hyperbolic relationship between *k*_obs_ and inhibitor concentration, suggesting that they also bind following slow‐binding Mechanism II. These findings are in agreement with previous studies by Moreno‐Yruela *et al*. concerning CI‐994^[30]^ and Soragni *et al*. concerning a constitutional isomer of PD‐106 (Cpd. 109).^[37]^ All these HDACi share a *ortho*‐aminoanilide ZBG but have very diverse cap groups. Bressi *et al*. suggested that *ortho*‐aminoanilides generally bind via an “induced‐fit” mechanism proposing that the unbound, solvated ligand forms an intramolecular hydrogen bond between the carbonyl oxygen and the *ortho*‐NH_2_ group of the ZBG. This hydrogen bond does not need to be broken for the formation of the initial encounter complex [EI] of enzyme and inhibitor. Over time hydrogen bond‐breaking and reorientation of the involved hydrogen enables favorable interactions with histidine side chains and the zinc ion of the active site, allowing the transition from the initial encounter complex [EI] to the substantially more stable binary enzyme inhibitor complex [E*I].^[17]^ This would explain the two‐step slow‐binding mechanism of the evaluated compounds regardless of the presence of a FPU or the cap group. However, the cap group seems to have great influence on the observed time‐dependence of the binding of the inhibitor. **6 d** and **10 c** share the same 4‐pyridinyl FPU bearing ZBG but the capless inhibitor **6 d** does not feature a classical HDACi cap group. The sterically demanding peptoid‐based cap group seems to slow down HDAC1 binding (see IC_50_‐shift experiments **6 d** vs. **10 c** and **VK‐1** vs. entinostat, SI, Table S1). Taken together, our results indicate that, regardless of FPU and cap, *ortho*‐aminoanilide‐based HDACi bind to HDAC1 in a two‐step slow‐binding mechanism. Additionally, at HDAC3 PD‐106,^[20]^ entinostat and **VK‐1** (see SI, Figure S6) all show slow‐binding Mechanism II as well, thereby indicating that HDAC3 is also susceptible to “induced‐fit” which is in excellent agreement with a recent study by Liu *et al.*.^[38]^

**Figure 8:**
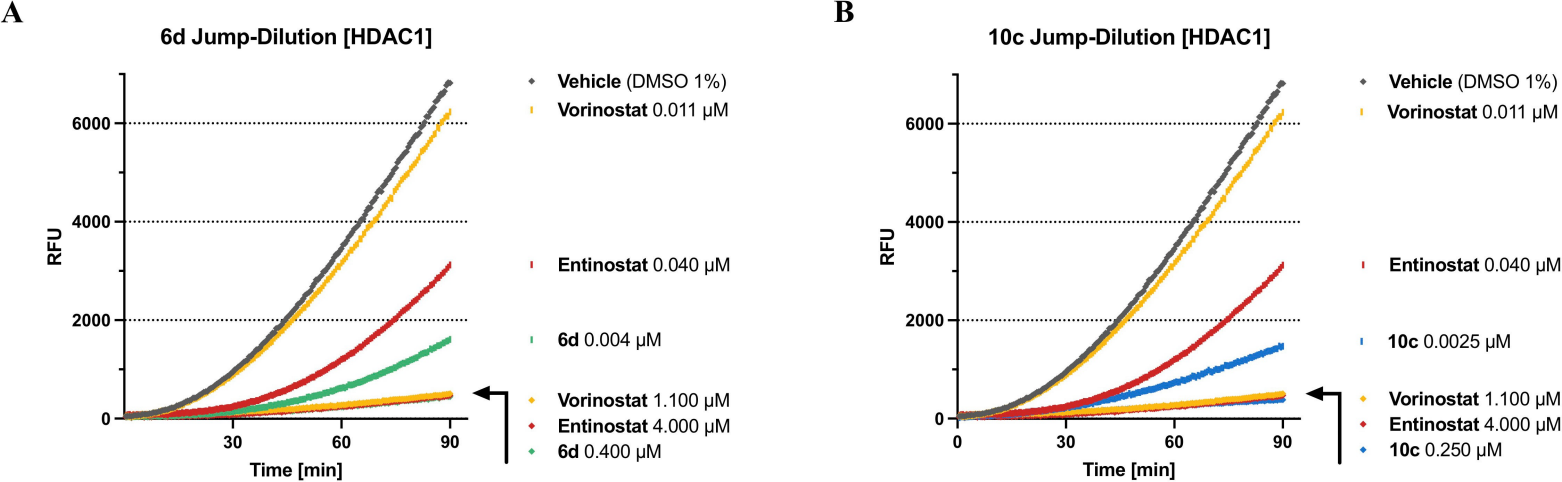
Progression curves of 100‐fold *Jump‐Dilution* experiments at HDAC1. Inhibitor concentrations are indicated on the right. Vorinostat and entinostat are included as reference inhibitors. Fluorescence of cleaved AMC is measured in relative fluorescence units (RFU). When compared to vehicle the final slope of the respective progression curves roughly indicates the amount of free enzyme. Final slope [RFU*min^−1^]: vehicle [DMSO 1 %]=111.9; vorinostat [0.011 μM]=105.0; entinostat [0.011 μM]=78.4. **A**) *Jump‐Dilution* experiment of **6 d**, final slope [RFU*min^−1^]: **6 d** [0.004 μM]=39.4; **B**) *Jump‐Dilution* experiment of **10 c**, final slope [RFU*min^−1^]: **10 c** [0.0025 μM]=26.2. Experiments were performed in triplicates.

**100‐fold** ***Jump‐Dilution*** **experiments**.^[20]^ If an enzyme–inhibitor pair forms an especially long‐lived “complex” this “complex” may also be stable upon dilution. Then, the recovery of enzyme activity reflects upon the dissociation of the inhibitor from the respective enzyme. Therefore, we assessed the dissociation behavior of our slow‐binding inhibitors **6 d** and **10 c** to determine if they are tight‐binding inhibitors of HDAC1. To this end, the respective enzyme in assay buffer was incubated with an excess of the inhibitor (at least 10‐fold IC_50_) or with blank (DMSO 1 %) for 1 hour at room temperature. Subsequently, this “incubation‐mix” was diluted 100‐fold either with the respective inhibitor at the original concentration or assay buffer. Substrate and trypsin were added to all samples and the time‐dependent *in situ* AMC release was monitored continuously by fluorescence readings recorded every 30 s for 60 or 90 min at 37 °C. The results for **6 d** and **10 c** compared to vorinostat and entinostat at HDAC1 are summarized in Figure 8 (for **VK‐1** see SI, Figure S7). As expected, due to the fast‐on / fast‐off binding characteristics of vorinostat, upon 100‐fold dilution HDAC1 regained nearly full deacetylase activity compared to vehicle control (DMSO 1 %). All examined *ortho*‐aminoanilides show to a certain degree tight binding properties towards HDAC1. In the case of the 100‐fold dilution of entinostat the deacetylase activity of HDAC1 was slowly restored. The progression curve suggests that by the end of the experiment a large part of the initially enzyme‐bound entinostat had disassociated. The 100‐fold dilutions of **6 d** and **10 c** show tight binding properties towards HDAC1 as well. However, both inhibitors disengage HDAC1 more slowly than entinostat, leading to considerably less deacetylase activity of HDAC1 within the timeframe of the experiment. The final slope of the respective progression curves (**6 d**: 39.4 RFU*min^−1^; **10 c**: 26.2 RFU*min^−1^) suggest that the dissociation of the full‐sized inhibitor from HDAC1 progresses even more slowly than the dissociation of the capless HDAC inhibitor, indicating that the cap group additionally slows down enzyme–dissociation. The results for **VK‐1** (see SI, Figure S7) back up these assumptions: despite the lack of a FPU, **VK‐1** disengages from HDAC1 more slowly than entinostat. We also assessed the disassociation behavior of entinostat and **VK‐1** at HDAC3 (see SI, Figure S8). Both inhibitors bind very tightly to HDAC3 while vorinostat disengages HDAC3 fast upon 100‐fold dilution.

**Figure 9:**
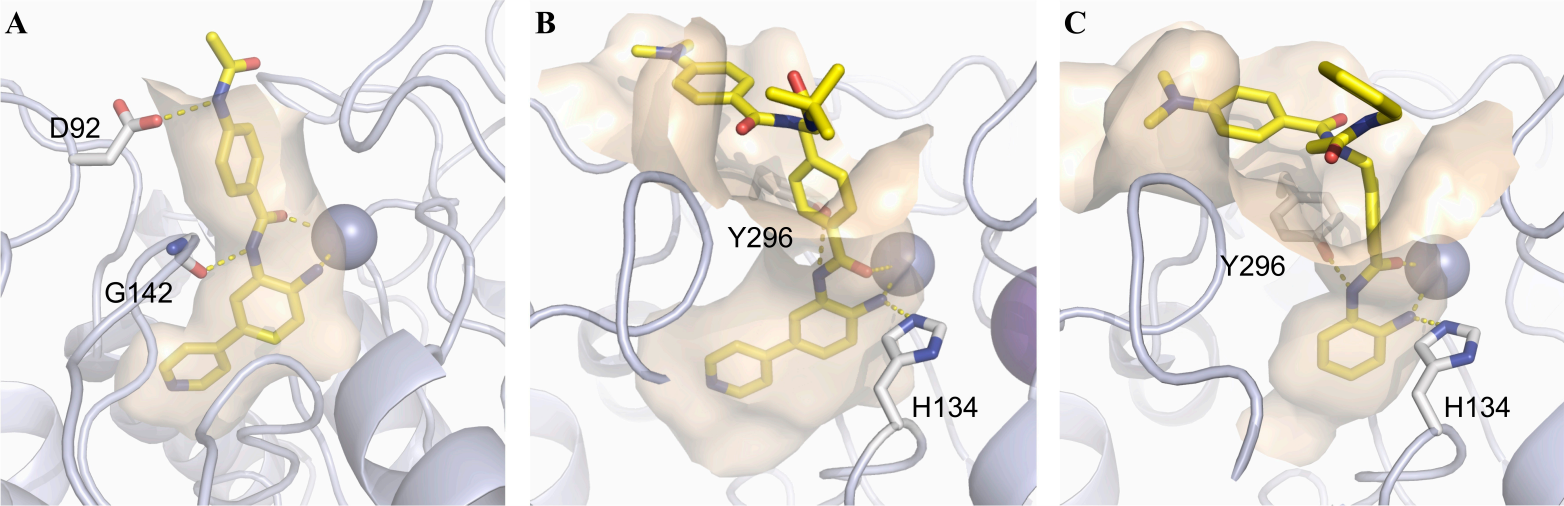
Docking pose of **6 d** (**A**), **10 c** (**B**) and **VK‐1** (**C**) in the catalytic domain of HDAC1 (PDB: 5ICN)^[39]^. Ligands are colored yellow, the catalytic Zn^2+^‐ion is shown as a gray sphere. The protein backbone is shown as light‐blue cartoon including the wheat‐colored protein surface surrounding the ligand. Side chain amino acids that show specific interactions with the ligand as well as the ligands are depicted as sticks. Polar interactions are represented by dashed lines in yellow.

**Docking of 6 d and 10 c**. For a better understanding of a possible binding mode of our inhibitors we docked **VK‐1**, **6 d** and **10 c** into HDAC1 (PDB: 5ICN).^[39]^ For further information, see **SI**, including Figure S9–S11. In the case of all compounds, the zinc‐binding occurs through the *ortho*‐aminoanilide moiety and as expected, the 4‐pyridinyl‐group of **6 d** and **10 c** clearly engages the “foot‐pocket” (see Figure 9). Both peptoid‐based inhibitors (**VK‐1** and **10 c**) form a hydrogen‐bond interaction between the Y296 side chain and the amide NH of the ZBG (see Figure 9B & 9 C). On the other hand, the capless HDACi **6 d** forms a hydrogen‐bond interaction between the amide NH of the ZBG and the carbonyl group of the G142 backbone. Although **6 d** lacks an actual cap‐group, its linker region is stabilized through a hydrogen‐bond interaction between its acetamide moiety and the side chain of D92. Interestingly, apart from hydrophobic interactions, the solvent exposed cap‐groups of **VK‐1** and **10 c** display no significant interactions with the enzyme surface.

**Anticancer properties of 6 d and 10 c**. Encouraged by the results of the WST‐8 assay we further examined the biological properties of the capless HDACi **6 d** and full‐sized inhibitor **10 c** *in vitro* and compared them to those of the FDA approved HDACi vorinostat and the clinically advanced class I selective HDACi entinostat.

**Figure 10:**
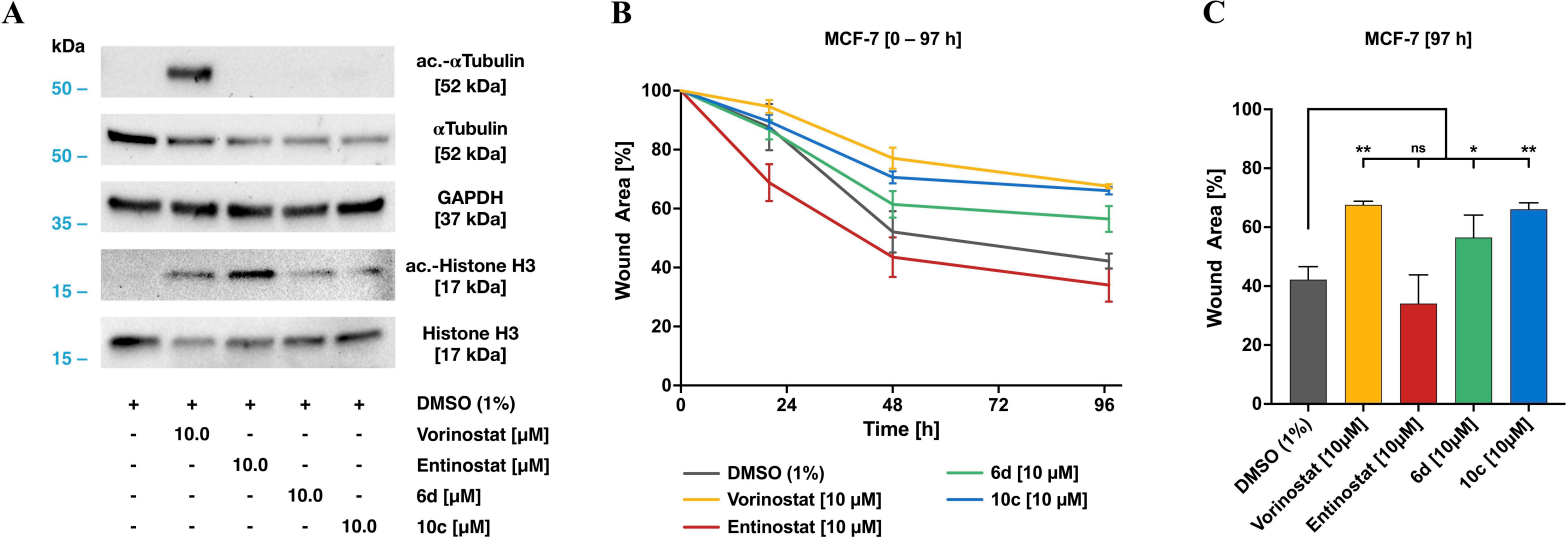
Biological evaluation of **6 d**, and **10 c** compared to the reference inhibitors vorinostat and entinostat. **A**) Target assessment by western blot. Direct target analysis of cell lysates after 24‐hour treatment with the indicated concentrations of the HDACi used. **B**) Cell migration kinetics of MCF‐7 breast cancer cells in a modified wound healing assay in the presence of selected HDACi. Time‐dependent wound closure of cell layer after 20 h, 48 h, and 97 h indicate an inhibitory efficiency of both **10 c** and **6 d**. **C**) Wound closure after 97 h compared to the respective control (t=0 h). Significances were determined by the Dunnett's test (n=3) and are indicated by asterisks (ns, not significant; *p<0.05; **p<0.01; ***p<0.001).

First, we investigated the intracellular target engagement of the inhibitors. To this end, we selected the human breast cancer MCF‐7 cell line as established tumor model and examined the hyperacetylation of histone H3 and α‐tubulin by western blot (see Figure 10A). Notably, in contrast to the pan‐HDACi vorinostat, the class I selective HDACi entinostat as well as **6 d**, and **10 c** did not affect HDAC6 activity, indicated by the non‐detectable amounts of acetylated α‐tubulin (Figure 10A). As expected, all inhibitors increased the level of acetylated histone H3 as indicator for class I HDAC inhibition compared to control, thereby confirming the selectivity profile observed in the cell‐free isoform profiling (see Table 1 & 2).

To investigate the impact of **6 d** and **10 c** on tumor cell dynamics, and thus invasive properties for tissue colonization, we performed cell migration studies using MCF‐7 cells. In a modified wound healing assay (see Figure 10B and 10C, for representative pictures of the time‐dependent wound closure see SI Figure S12) cells were preincubated with 10 μM of the indicated compounds and cell migration was followed over 97 h and compared to the DMSO treated control cells. As illustrated in Figure 10B & 10C, **10 c** and vorinostat displayed the strongest inhibitory potency compared to control. Moreover, both the HDAC1/2 selective inhibitor **10 c** as well as the pan‐HDACi vorinostat attenuated cell migration comparably and significantly. The capless HDACi **6 d** also attenuated cell migration significantly, however, the effect was less pronounced as observed for **10 c**. Notably, the HDAC1‐3 selective HDACi entinostat had no significant effect on cell migration.

**Figure 11:**
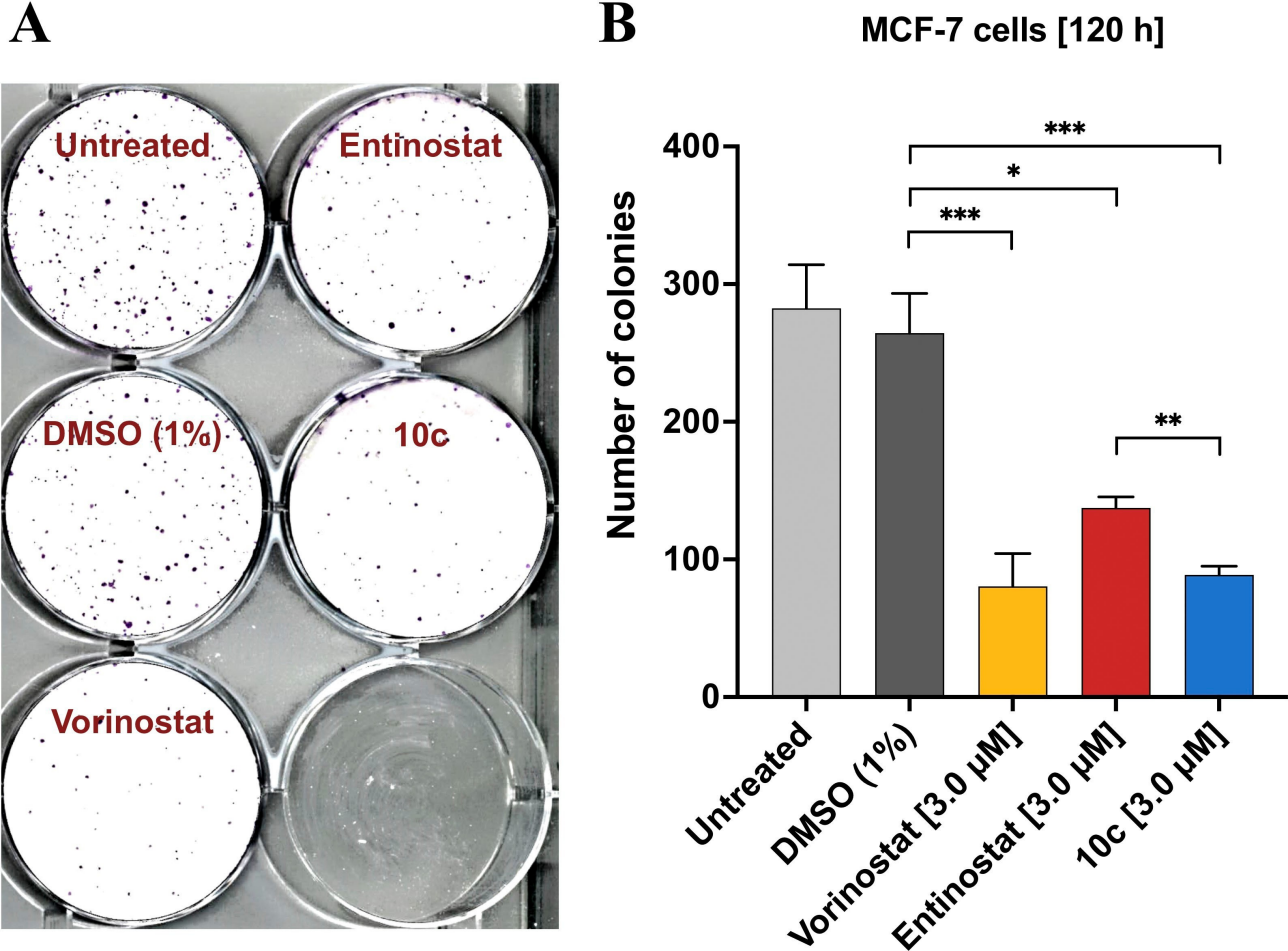
Effect of HDAC inhibition on colony formation (clonogenic assay) in MCF‐7 cells. **A**) Representative image of clonal proliferation after seeding 750 single cells per well. Prior to plating, cells were treated for 48 h with the respective agent (3.0 μM; as indicated) or left untreated. **B**) Quantitation of colony formation. Mean values ±SD (three independent experiments) of the absolute number of colonies is given. Significances were determined using the t‐test (n=3) and are indicated by asterisks (*p<0.05; **p<0.02; ***p<0.01).

Additionally, the biological effects of **10 c** were evaluated in MCF‐7 cells in a clonogenic assay (Figure 11) using the pan‐HDACi vorinostat and the class I selective HDACi entinostat as control compounds. Untreated and vehicle‐treated cells showed a plating efficiency (see Experimental Section) of 37.6 % and 35.2 %, respectively. Treatment with all three HDACi reduced the number of colonies significantly (Figure 11B), decreasing the plating efficiency to 10.7 % (vorinostat), 18.3 % (entinostat), and 11.8 % (**10 c**). Of note, **10 c** showed a higher potency in inhibiting colony formation than entinostat and was comparable in its antineoplastic activity with the FDA‐approved pan‐HDAC inhibitor vorinostat (see also: Figure 11B).

## Conclusion

In summary, we have synthesized nine capless HDACi based on the CI‐994 scaffold with nine different FPUs and investigated their structure‐activity and structure‐physicochemical property relationships. Based on the overall data set, the capless HDACi **6 d** emerged as the most promising scaffold. Its 4‐pyridinyl FPU showed favorable physicochemical properties with a moderate lipophilicity (logD_7.4_=1.75) and acceptable solubility (ten times higher than the corresponding phenyl‐analog **6 a**), a low nanomolar inhibitory activity against HDAC1 & 2 (HDAC1 IC_50_: 13.2 nM; HDAC2 IC_50_: 77.2 nM) and good selectivity over HDAC3 & 6 (HDAC3 IC_50_: 8,908 nM; HDAC6 IC_50_: >10 μM). Subsequently, the FPU of **6 d** was introduced to the peptoid‐based scaffold of **VK‐1** yielding the HDAC1/2 selective inhibitor **10 c** (hereafter referred to as **LSH‐A54**) whose antiproliferative properties were superior to the late‐stage clinical candidate entinostat (HDAC1‐3 selective) and FDA‐approved HDACi vorinostat (pan‐HDACi) in a WST‐8 assay against three different breast cancer entities. The subsequent evaluation of the time‐dependent IC_50_‐shift against HDAC1 & 2 indicated slow‐binding characteristics for both **6 d** and **LSH‐A54**. Furthermore, the assessment of the respective binding kinetics showed that the *ortho*‐aminoanilide‐based HDACi **VK‐1**, **6 d** and **LSH‐A54** bind tightly to HDAC1 in a two‐step slow‐binding mechanism regardless of FPU or cap group, but FPU as well as cap group impact the observed time‐dependence of the inhibitor‐binding. In a modified wound healing assay, **LSH‐A54** attenuated cell migration significantly and comparably to the pan‐HDACi vorinostat, whereas the HDAC1‐3 selective HDACi entinostat had no significant effect on cell migration. Furthermore, in the following clonogenic survival assay, **LSH‐A54** reduced the number of colonies significantly compared to entinostat and showed comparable activity to pan‐HDACi vorinostat. Taken together, **LSH‐A54** is a slow‐binding, selective HDAC1/2 inhibitor with encouraging effects on tumor cell dynamics and thus invasive properties for tissue colonization. Moreover, **LSH‐A54** showed promising effects on cell viability and colony formation comparable to FDA‐approved HDACi vorinostat, thereby highlighting the potential of this inhibitor as an advancement in the ongoing development of effective antineoplastic drugs.

## Experimental Section

**General Information & Chemistry**. Chemicals were obtained from abcr GmbH, Acros Organics, Carbolution Chemicals, Fluorochem, Sigma‐Aldrich, TCI Chemicals or VWR and used without further purification. Technical grade solvents were distilled prior to use. For all HPLC purposes, acetonitrile in HPLC‐grade quality (HiPerSolv CHROMANORM, *VWR*) was used. Water was purified with a Milli‐Q Simplicity 185 Water Purification System (Merck Millipore). Air‐sensitive reactions were carried out under argon atmosphere utilizing standard Schlenk techniques. **Thin‐layer chromatography (TLC)** was carried out on prefabricated plates (silica gel 60, F_254_, *Merck*). Components were visualized either by irradiation with ultraviolet light (254 nm or 366 nm) or by staining appropriately. Column Chromatography was carried out on silica gel (NORMASIL 60®, 40–63 μm, *VWR* or MACHEREY‐NAGEL silica gel 60®, 40–63 μm). If no solvent is stated an aqueous solution was prepared with purified water. Mixtures of two or more solvents are specified as “solvent A”/“solvent B”; 67 : 33; (*v*/*v*), meaning that 100 mL of the respective mixture consists of 67 mL of “solvent A” and 33 mL of “solvent B”. The uncorrected melting points were determined using a Büchi Melting Point M‐565 apparatus. **Nuclear Magnetic Resonance Spectroscopy (NMR)**: Proton (^1^H), boron (^11^B), carbon (^13^C) and fluorine (^19^F {^1^H}) NMR spectra were recorded either on a Bruker Avance III HD 400 MHz at a frequency of 400 MHz (^1^H), 128 MHz (^11^B), 101 MHz (^13^C) and 377 MHz (^19^F), a Varian/Agilent Mecury‐plus‐400 at a frequency of 400 MHz (^1^H), 128 MHz (^11^B), 101 MHz (^13^C) and 376 MHz (^19^F) or a Varian/Agilent Mecury‐plus‐300 at a frequency of 300 MHz (^1^H), 96 MHz (^11^B), 75 MHz (^13^C) and 282 MHz (^19^F). The chemical shifts are given in parts per million (ppm). As solvents deuterated chloroform (chloroform‐*d*), deuterated methanol (methanol‐*d*_4_) and deuterated dimethyl sulfoxide (DMSO‐*d*_6_) were used. The residual solvent signal (chloroform‐*d*: ^1^H NMR: 7.26 ppm, ^13^C NMR: 77.1 ppm; DMSO‐*d*_6_: ^1^H NMR: 2.50 ppm, ^13^C NMR: 39.52 ppm; Methanol‐*d*_4_: ^1^H NMR: 3.31 ppm, 4.87 ppm, ^13^C NMR: 49.00 ppm) was used for calibration. The multiplicity of each signal is reported as singlet (s), doublet (d), triplet (t), quartet (q), multiplet (m) or combinations thereof. Multiplicities and coupling constants are reported as measured and might disagree with the expected values. In the case of the peptoid compounds **8 a**–**c**, **9 a**–**c**, and **10 a**–**c**, and due to the well‐known phenomenon of *cis*/*trans*‐amide bond rotamerism in peptoids,[^12^, ^40^] certain ^1^H and ^13^C NMR signals can occur as two distinct sets of signals. In this case, the assigned signals correspond to the major rotamer conformation. ^19^F NMR spectra were recorded proton‐decoupled if not stated otherwise. **Mass Spectrometry**: High resolution electrospray ionisation mass spectra (HRMS‐ESI) were acquired either with a *Bruker Daltonik GmbH* micrOTOF coupled to a an *LC Packings* Ultimate HPLC system and controlled by micrOTOFControl3.4 and HyStar 3.2‐LC/MS or with a *Bruker Daltonik GmbH* ESI‐qTOF Impact II coupled to a *Dionex* UltiMate™ 3000 UHPLC system and controlled by micrOTOFControl 4.0 und HyStar 3.2‐LC/MS. Low resolution electrospray ionisation mass spectra (LRMS‐ESI) were acquired with an *Advion* expression® compact mass spectrometer (CMS) coupled with an automated TLC plate reader Plate Express® (*Advion*). **High Performance Liquid Chromatography (HPLC)**: For analytical purposes either a *Gynkotek* Gina 50 HPLC system (Detector: *Gynkotek* UVD340 U, Pump: *Dionex* P680 HPLC pump, column oven: *Dionex* STH 585) with a Nucleodur 100‐5 C18 ec (250×4.6 mm, *Macherey Nagel*) column, a *Thermo Fisher Scientific* UltiMate™ 3000 UHPLC system with a Nucleodur 100‐5 C18 (250×4.6 mm, *Macherey Nagel*). A flow rate of 1 mL/min and a temperature of 25 °C were set. A *Varian* ProStar system with a Nucleodur 110‐5 C18 HTec (150×32 mm, *Macherey Nagel*) column with 14 mL/min was used for preparative purposes. Detection was implemented by UV absorption measurement at a wavelength of λ=220 nm and λ=250 nm. Bidest. H_2_O (A) and MeCN (B) were used as eluents with an addition of 0.1 % TFA for eluent A. For analytical purposes after column equilibration for 5 min a linear gradient from 5 % A to 95 % B in 7 min followed by an isocratic regime of 95 % B for 10 min. For preparative purposes after column equilibration for 5 min a linear gradient from 5 % A to 95 % B in 15 min followed by an isocratic regime of 95 % B for 10 min was used. **Purity**: The purity of all final compounds was 95 % or higher. Purity was determined via HPLC at 250 nm using the protocols described above, if not stated otherwise.

The following compounds were synthesized according to literature and used without further purification: *tert*‐butyl (4‐bromo‐2‐nitrophenyl) carbamate,^[41]^ *tert*‐butyl (3‐nitro‐[1,1′‐biphenyl]‐4‐yl)carbamate,^[42]^ methyl 4‐(aminomethyl)benzoate hydrochloride,^[43]^ methyl 4‐({*N*‐[2‐(benzyl‐amino)‐2‐oxoethyl]‐4‐(dimethylamino)benzamido}methyl)benzoate,^[11]^ methyl 4‐({*N*‐[2‐(cyclohexylamino)‐2‐oxoethyl]‐4‐(dimethylamino)benz‐amido}methyl)‐benzoate,^[28]^ methyl 4‐({*N*‐[2‐(*tert*‐butylamino)‐2‐oxoethyl]‐4‐(dimethyl‐amino)benzamido}methyl)benzoate.^[28]^

Further experimental procedures and data, including characterization data, NMR spectra, and HPLC traces etc., are provided in the Supporting Information.

## Author Contributions

The manuscript was written through the contributions of all authors. All authors have given approval to the final version of the manuscript.

## Abbreviations

AMC: 7‐amino‐4‐methyl‐2*H*‐chromen‐2‐one
BSA: bovine serum albumin
Cpd.: compound
CTCL: cutaneous T‐cell lymphoma
DCM: dichloromethane
DIPEA: *N,N*‐diiso‐propylethylamine;
EDC*HCl: 1‐ethyl‐3‐(3‐dimethylaminopropyl) carbodiimide hydrochlorid
EMA: European Medicines Agency
EtOAc: ethyl acetate
EtOH: ethanol
FDA: Food and Drug Administration
HAT: histone acetyl transferase
HDAC: histone deacetylases
HOBt: benzotriazol‐1‐ol
HR^+^
: hormone‐receptor‐positive
KOAc: Potassium acetate
MeCN: acetonitrile
MeOH: methanol
MM: multiple myeloma
4 Å MS: molecular sieve, pore size 4 Å
NMPA: National Medical Products Administration of China
OECD: Organisation for Economic Co‐operation and Development
PBS: phosphate buffered saline
PTCL: peripheral T‐cell lymphoma
PTFE: polytetrafluoroethylene
RFU: relative fluorescence units
TEA: triethylamine
TFA: trifluoroacetic acid
WB: western blot
ZBG: zinc binding group
ZMAL: benzyl {6‐acetamido‐1‐[(4‐methyl‐2‐oxo‐2*H*‐chromen‐7‐yl)amino]‐1‐oxohexan‐2‐yl}carbamate

## Conflict of interest

The authors declare no conflict of interest.

1

## Supporting information

As a service to our authors and readers, this journal provides supporting information supplied by the authors. Such materials are peer reviewed and may be re‐organized for online delivery, but are not copy‐edited or typeset. Technical support issues arising from supporting information (other than missing files) should be addressed to the authors.

Supporting Information

## Data Availability

The data that support the findings of this study are available from the corresponding author upon reasonable request.
