# Supplementary material for: Balancing Histone Deacetylase (HDAC) Inhibition and Drug‐likeness: Biological and Physicochemical Evaluation of Class I Selective HDAC Inhibitors
Source: ChemMedChem. 2022 Feb 18;17(9):e202100755. doi: 10.1002/cmdc.202100755 (PMC9303312; doi:10.1002/cmdc.202100755)
Supplement: Supplementary file 1 — Supporting Information [file CMDC-17-0-s001.pdf]

ChemMedChem

Supporting Information

Balancing Histone Deacetylase (HDAC) Inhibition and Drug-likeness: Biological and Physicochemical Evaluation of Class I Selective HDAC Inhibitors

Linda Schäker-Hübner, Reza Haschemi, Thomas Büch, Fabian B. Kraft, Birke Brumme, Andrea Schöler, Robert Jenke, Jens Meiler, Achim Aigner, Gerd Bendas, and Finn K. Hansen*

Table of Contents

SUPPLEMENTAL TABLES AND FIGURES **S3**

CYTOTOXICITY SCREENING OF 6D AND 10A-C S3
IC_{50}-SHIFT EXPERIMENTS. S5
DETERMINATION OF BINDING MECHANISM VIA *PROGRESSION-METHOD*. S7
100-FOLD *JUMP-DILUTION* EXPERIMENTS S9
DOCKING OF 6D (LSH-A33), 10C (LSH-A54) AND VK-1 .... S11
MODIFIED WOUND HEALING ASSAY AT MCF-7 BREAST CANCER CELLS. S13

EXPERIMENTAL SECTION **S14**

GENERAL INFORMATION & CHEMISTRY. S14
HDAC ASSAYS S39
PHYSICOCHEMICAL PROPERTIES S41
DOCKING S43
BIOLOGICAL EVALUATION S46
DETERMINATION OF LIPOPHILICITY AND SOLUBILITY (EXAMPLE) S49

NMR SPECTRA OF SYNTHESIZED COMPOUNDS BY NUMBER **S54**

SYNTHESIS OF FRAGMENT COMPOUNDS 6A-I S54
SYNTHESIS OF FULL-SIZED INHIBITORS 10A-C S99

HPLC CHROMATOGRAMS OF TARGET COMPOUNDS **S110**

REFERENCES **S117**

Cytotoxicity Screening of 6d and 10a-c.

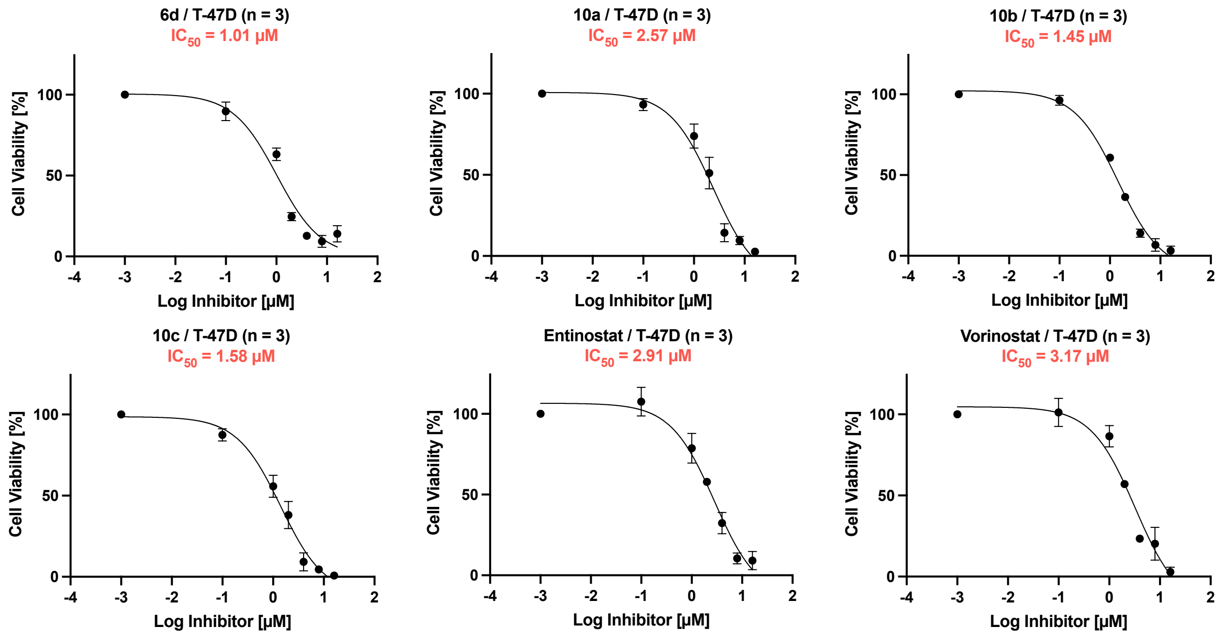

Figure S1. Representative dose response curves (mean ± SEM plotted from three independent experiments) of selected compounds at the T-47D breast cancer cell line.

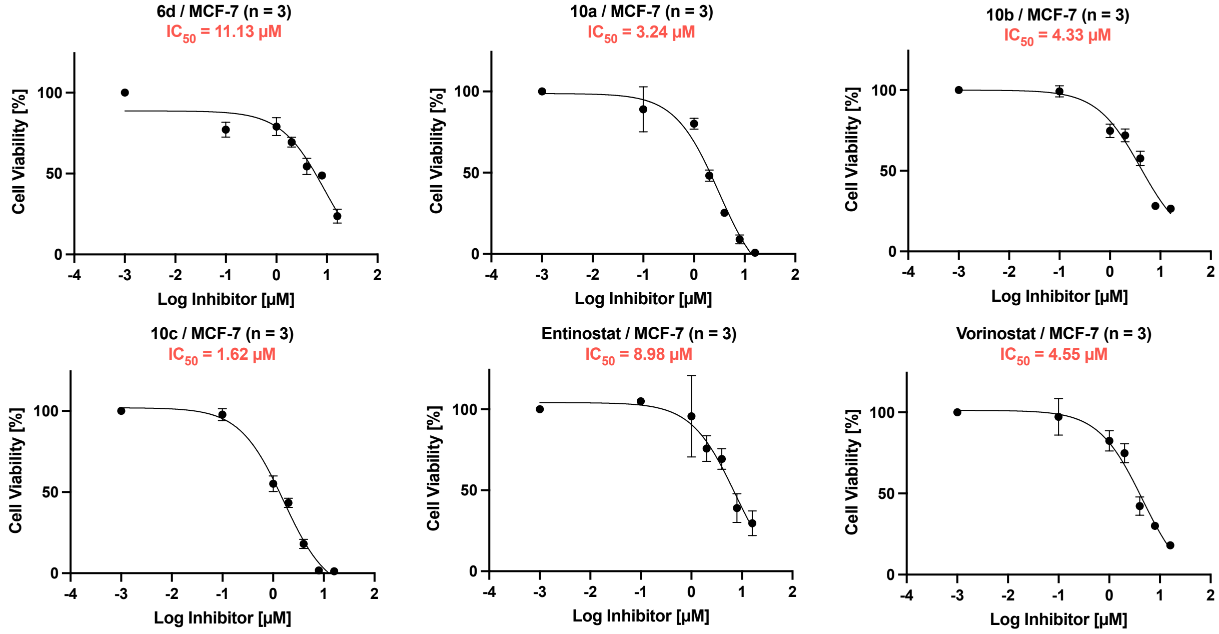

Figure S2. Representative dose response curves (mean ± SEM plotted from three independent experiments) of selected compounds at the MCF-7 breast cancer cell line.

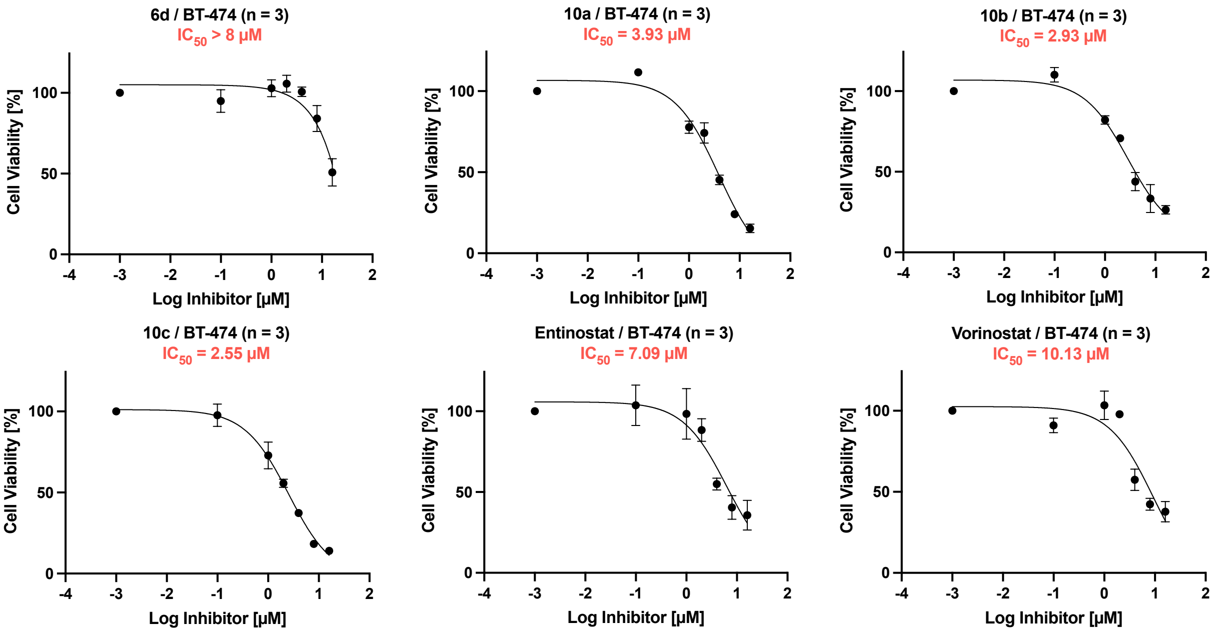

Figure S3. Representative dose response curves (mean ± SEM plotted from three independent experiments) of selected compounds at the BT-474 breast cancer cell line.

IC$_{50}$-shift experiments.

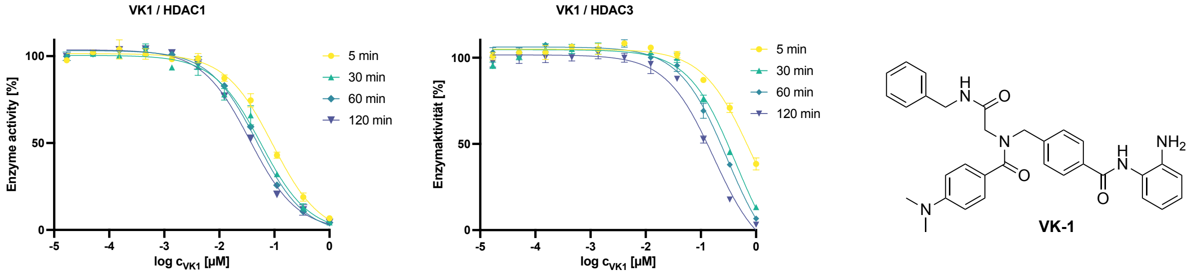

Figure S4. Dependence of preincubation time as indicated and IC$_{50}$ values of **VK-1** at selected HDAC isoforms. Experiments were performed in triplicates.

Table S1. Dependence of preincubation time and HDAC1 IC$_{50}$ values for selected inhibitors and HDAC isoforms, including P95 intervals. Experiments were performed in triplicates.

	HDAC1 IC$_{50}$ [nM]				
preincubation time [a]	5 min	15 min	30 min	60 min	120 min
**vorinostat**	103.9 (95.6 – 113.0) [b]	111.0 (102.4 – 120.4) [b]	99.0 (92.6 – 106.0) [b]	110.0 (101.1 – 119.8) [b]	110.5 (97.2 – 125.6) [b]
**6d**	54.1 (48.2 – 60.8) [b]	53.5 (46.0 – 62.2) [b]	44.5 (37.8 – 52.3) [b]	40.8 (33.8 – 49.3) [b]	25.4 (21.8 – 29.6) [b]
**10c**	53.0 (47.0 – 59.8) [b]	48.9 (44.0 – 54.4) [b]	34.7 (31.6 – 38.1) [b]	27.9 (24.8 – 31.4) [b]	20.7 (18.4 – 23.4) [b]
**VK-1**	93.0 (79.6 – 108.8) [b]	n.d.	57.4 (47.0 – 70.2) [b]	48.3 (42.4 – 55.0) [b]	35.6 (31.3 – 40.5) [b]
**entinostat**	500.4 (445.5 – 561.8) [b]	489.2 (425.5 – 562.5) [b]	419.9 (370.5 – 475.9) [b]	387.7 (345.4 – 435.3) [b]	345.3 (303.7 – 392.5) [b]

[a] preincubation at 20°C; [b] 95% confidence intervals for IC$_{50}$ [nM]; n.d.: not determined.

Table S2. Dependence of preincubation time and HDAC2 IC_{50} values for selected inhibitors and HDAC isoforms, including P95 intervals. Experiments were performed in triplicates.

	HDAC2 IC_{50} [nM]				
preincubation time [a]	5 min	15 min	30 min	60 min	120 min
**vorinostat**	188.7 (170.3 – 209.0) [b]	186.2 (172.2 – 201.3) [b]	185.9 (168.8 – 204.8) [b]	0,1814 (127.3 – 257.3) [b]	0,2144 (190.3 – 241.5) [b]
**6d**	98.2 (85.6 – 112.7) [b]	89.0 (78.0 – 101.4) [b]	83.0 (74.6 – 92.2) [b]	77.4 (66.0 – 90.8) [b]	63.2 (54.6 – 73.2) [b]
**10c**	117.4 (102.1 – 135.2) [b]	106.4 (89.8 – 126.2) [b]	109.0 (101.1 – 117.6) [b]	84.0 (75.8 – 93.1) [b]	78.1 (69.9 – 87.3) [b]
**VK-1**	n.d.	n.d.	n.d.	n.d.	n.d.
**entinostat**	515.7 (463.1 – 574.4) [b]	467.7 (423.8 – 516.2) [b]	396.3 (356.6 – 440.2) [b]	384.6 (353.9 – 418.0) [b]	334.5 (294.9 – 379.3) [b]

[a] preincubation at 20°C; [b] 95% confidence intervals for IC_{50} [nM]; n.d.: not determined.

Table S3. Dependence of preincubation time and HDAC3 IC_{50} values for selected inhibitors and HDAC isoforms, including P95 intervals. Experiments were performed in triplicates.

	HDAC3 IC_{50} [nM]				
preincubation time [a]	5 min	15 min	30 min	60 min	120 min
**vorinostat**	124.3 (112.8 – 137.0) [b]	n.d.	119.2 (106.7 – 133.2) [b]	119.6 (105.3 – 135.9) [b]	112.2 (97.6 – 129.0) [b]
**6d**	n.d.	n.d.	n.d.	n.d.	n.d.
**10c**	n.d.	n.d.	n.d.	n.d.	n.d.
**VK-1**	794.5 (539.3 – 1270) [b]	n.d.	432.2 (321.8 – 595.5) [b]	298.8 (250.4 – 358.6) [b]	171.2 (139.4 – 211.2) [b]
**entinostat**	1225 (994.9 – 1512) [b]	n.d.	428.8 (365.0 – 503.7) [b]	357.2 (291.1 – 438.6) [b]	162.7 (137.2 – 192.9) [b]

[a] preincubation at 20°C; [b] 95% confidence intervals for IC_{50} [nM]; n.d.: not determined.

Determination of binding mechanism via *Progression-Method*.

MICHALIS-MENTEN constant (K_m)

A B

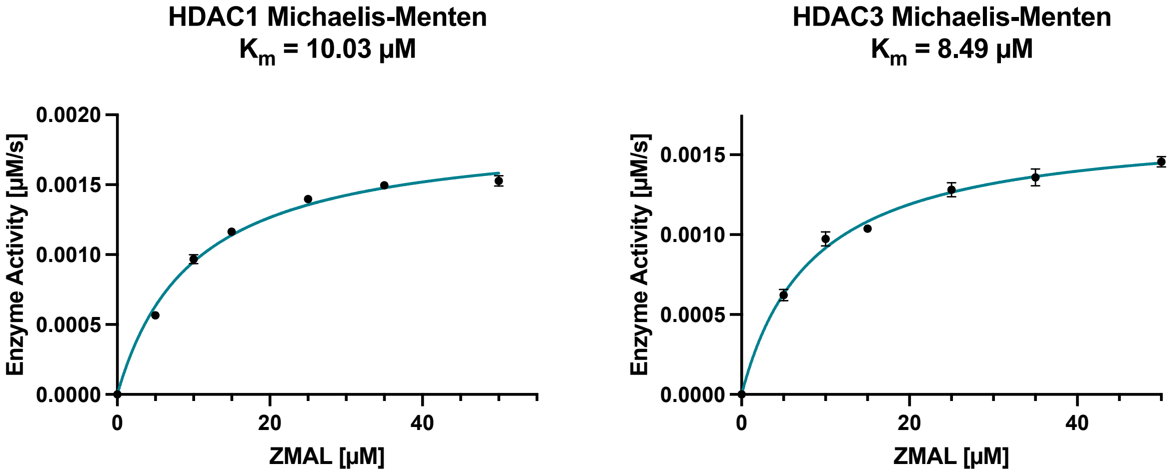

Figure S5. K_m determination for HDAC1 (A) and HDAC3 (B) using a series of substrate concentrations. Steady-state velocities [µM*s^{-1}] (mean ± SD) were plotted against the corresponding substrate concentrations [µM] and fitted to the MICHALIS-MENTEN equation yielding the respective MICHALIS-MENTEN constants: K_m HDAC1 = 10.03 µM; K_m HDAC3 = 8.49 µM. Experiments were performed in triplicates.

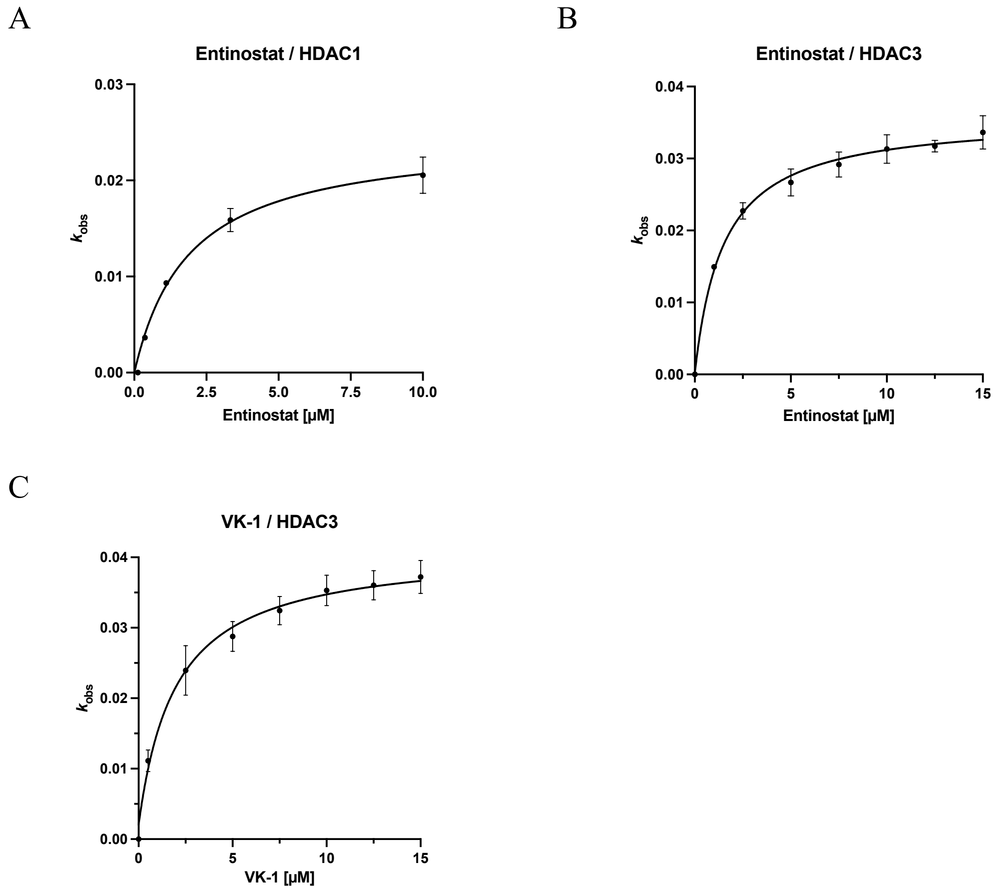

Figure S6. Slow-binding characteristics of **entinostat** and **VK-1**. The apparent first-order rate constants k_{obs} (mean ± SD) were plotted against the corresponding inhibitor concentrations [I]. The resulting curves were fitted to **Eq. 5**. (**S6A–C**) Inhibitor concentrations are indicated. **A**) **entinostat** at HDAC1, the relationship between k_{obs} and [I] indicates slow-binding Mechanism II; **B**) **entinostat**/HDAC3, the relationship between k_{obs} and [I] indicates slow-binding Mechanism II; **C**) **VK-1**/HDAC3, the relationship between k_{obs} and [I] indicates slow-binding Mechanism II. Experiments were performed in triplicates.

100-fold *Jump-Dilution* experiments.

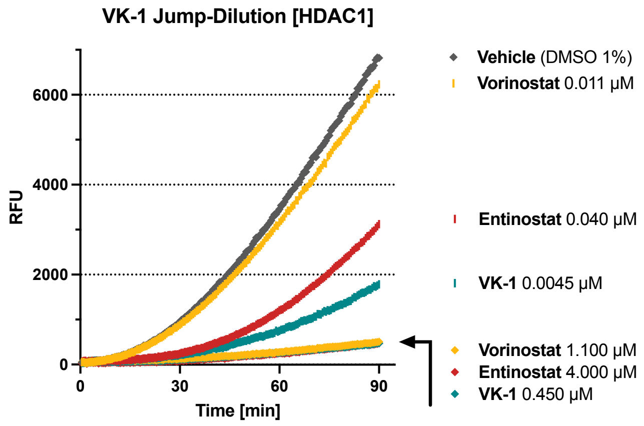

Figure S7. Progression curves of 100fold *Jump-Dilution* experiment (HDAC1) of **VK-1**. Inhibitor concentrations are indicated on the right. **Vorinostat** and **entinostat** are included as reference inhibitors. Fluorescence of cleaved AMC is measured in relative fluorescence units (RFU). When compared to vehicle the final slope of the respective progression curve roughly indicates the amount of free enzyme. Final slope [RFU*min^{-1}]: **vehicle** [DMSO 1%] = 111.9; **vorinostat** [0.011 µM] = 105.0; **entinostat** [0.011 µM] = 78.4; **VK-1** [0.0045 µM] = 39.2. Experiments were performed in triplicates.

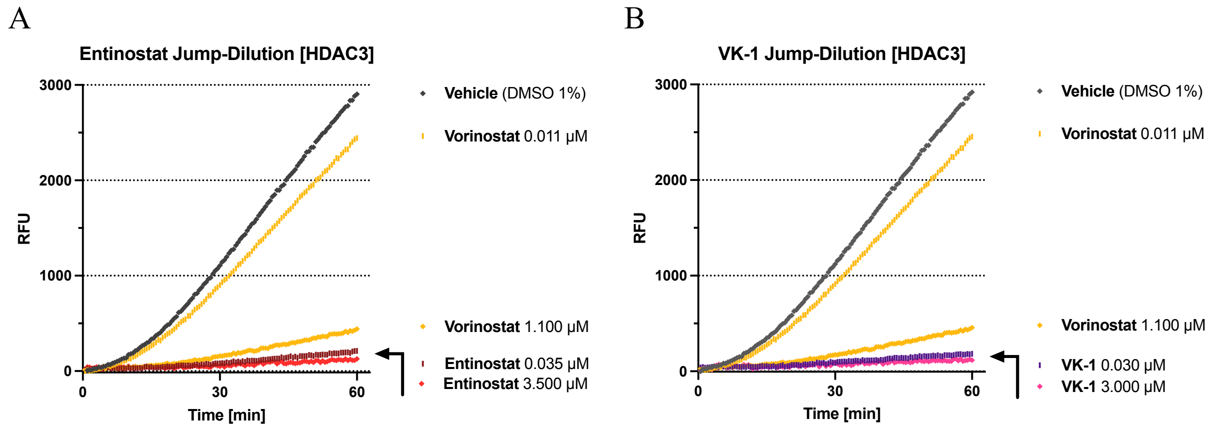

Figure S8. Progression curves of 100fold *Jump-Dilution* experiments at HDAC3. Inhibitor concentrations are indicated on the right. **Vorinostat** is included as reference inhibitor. Fluorescence of cleaved AMC is measured in relative fluorescence units (RFU). When compared to vehicle the final slope of the respective progression curves roughly indicates the amount of free enzyme. Final slope [RFU*min^{-1}]: **vehicle** [DMSO 1%] = 56.7; **vorinostat** [0.011 µM] = 50.8. **A**) *Jump-Dilution* experiment of **entinostat**, final slope [RFU*min^{-1}]: **entinostat** [0.035 µM] = 4.65.; **B**) *Jump-Dilution* experiment of **VK-1**, final slope [RFU*min^{-1}]: **VK-1** [0.030 µM] = 2.51. Experiments were performed in triplicates.

Docking of 6d (LSH-A33), 10c (LSH-A54) and VK-1

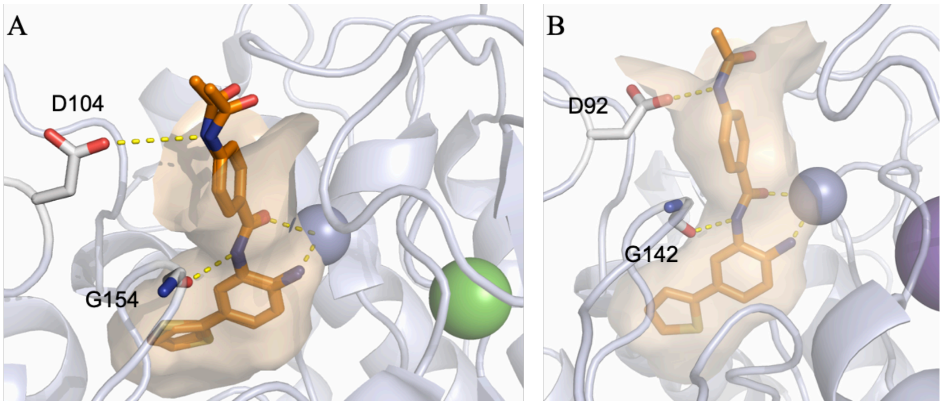

Figure S9. Model Building based on PDB ID: 4LY1. **A**) Crystal structure of the catalytic domain of human HDAC2 in complex with inhibitor **6c** (PDB: 4LY1)[1]. **B**) Docking pose of inhibitor **6c** in human HDAC1 (PDB: 5ICN)[2]. The ligand is colored orange, the Zn^{2+}-ion is shown as a grey sphere, sodium and potassium are shown as green and purple spheres respectively. The protein backbone is shown as light-blue cartoon including the wheat-colored protein surface surrounding the ligand. Side chain amino acids that show specific interactions with the ligand as well as the ligands are depicted as sticks. Polar interactions are represented by dashed, yellow lines.

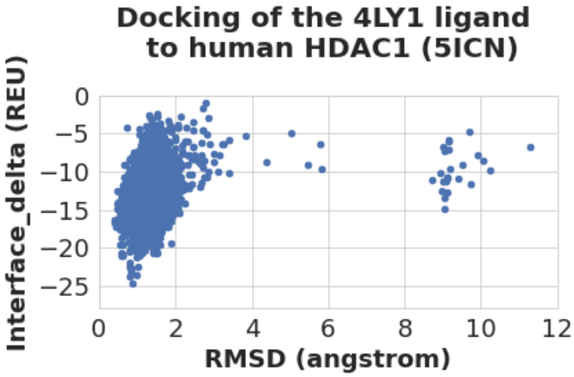

Figure S10. Ligand RMSD vs. interface_delta plots for the docking of the 4LY1-ligand to the human HDAC1 enzyme (PDB: 5ICN). It was plotted against the best scoring model.

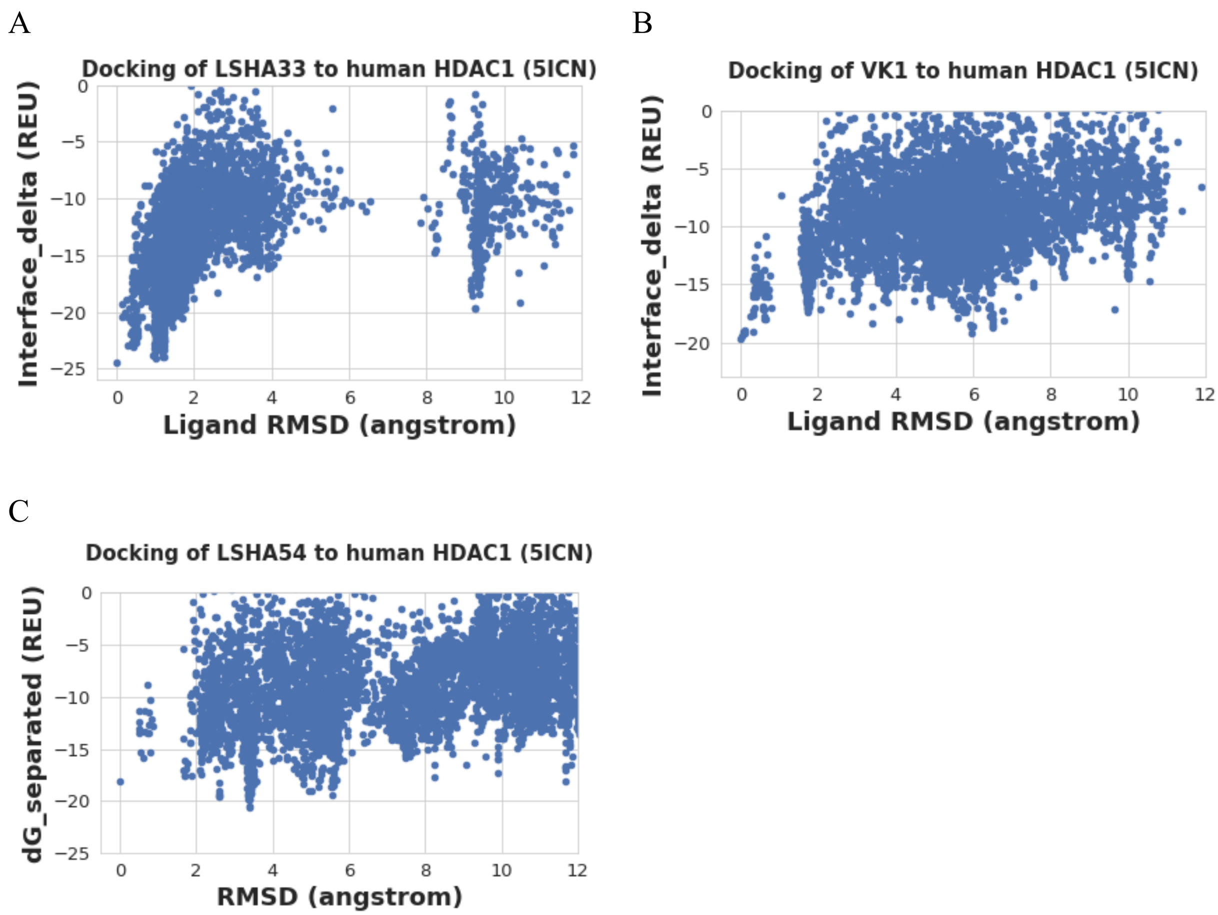

Figure S11. Ligand RMSD vs. interface_delta plots for **6d** (LSH-A33) **(A)**, **VK-1** **(B)** and **10c** (LSH-A54) **(C)** after the focus refinement docking. **6d** (LSH-A33) and **VK-1** are plotted against the best scoring model. **10c** (LSH-A54) is plotted against the model with the highest accordance to the predicted binding modes of **6d** (LSH-A33) and **VK-1**.

Modified Wound Healing Assay at MCF-7 Breast Cancer Cells.

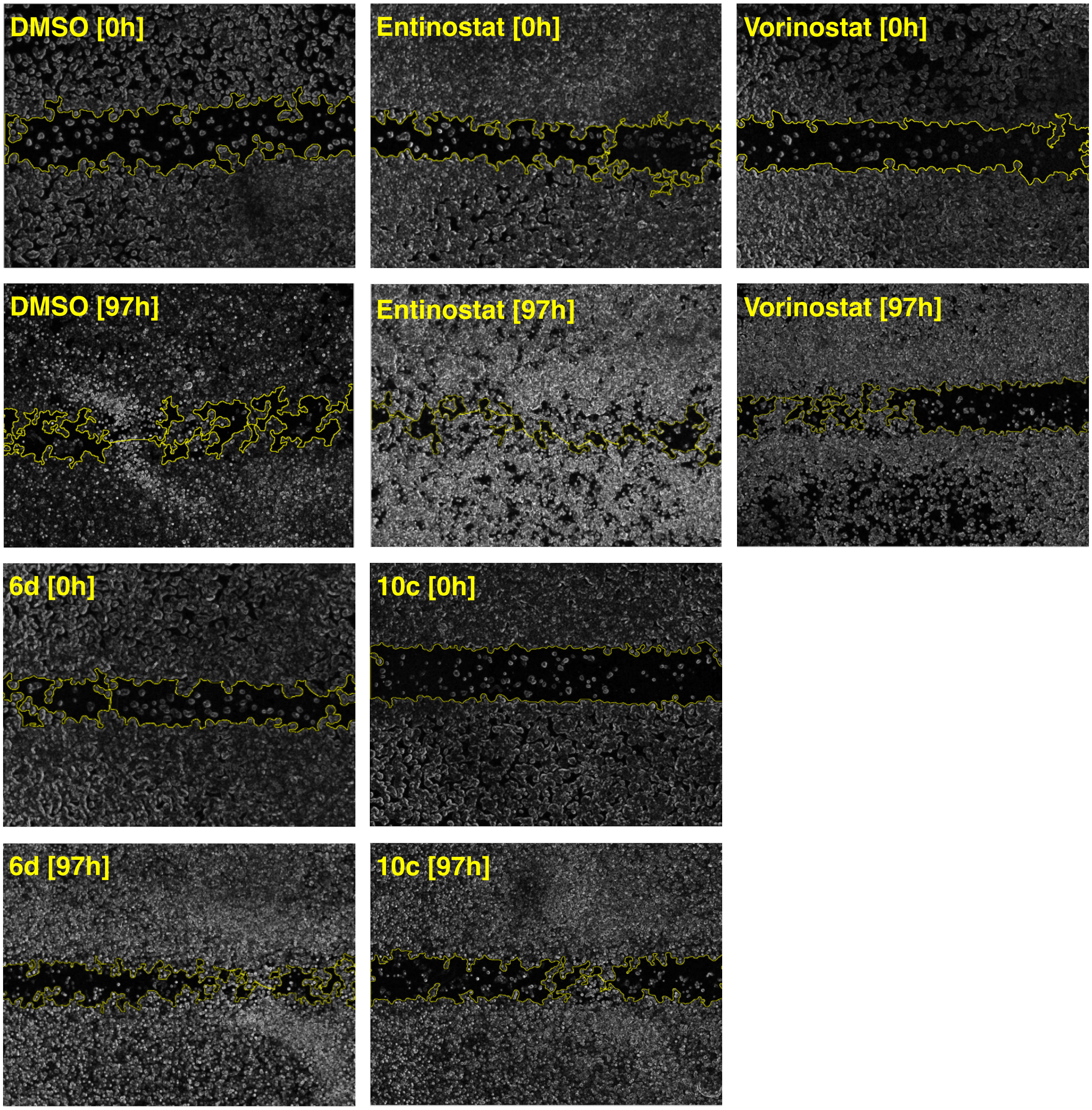

Figure S12. Modified wound healing assay at MCF-7 cells and the impact of vehicle (1% DMSO) or selected HDACi (10 µM): Representative pictures of the time-dependent wound closure. Incubation time, and HDACi or vehicle as indicated.

EXPERIMENTAL SECTION

General Information & Chemistry.

Chemicals were obtained from abcr GmbH, Acros Organics, Carbolution Chemicals, Fluorochem, Sigma-Aldrich, TCI Chemicals or VWR and used without further purification. Technical grade solvents were distilled prior to use. For all HPLC purposes, acetonitrile in HPLC-grade quality (HiPerSolv CHROMANORM, *VWR*) was used. Water was purified with a Milli-Q Simplicity 185 Water Purification System (Merck Millipore). Air-sensitive reactions were carried out under argon atmosphere utilizing standard Schlenk techniques. **Thin-layer chromatography (TLC)** was carried out on prefabricated plates (silica gel 60, F_{254}, *Merck*). Components were visualized either by irradiation with ultraviolet light (254 nm or 366 nm) or by staining appropriately. Column Chromatography was carried out on silica gel (NORMASIL 60®, 40-63 µm, *VWR* or MACHEREY-NAGEL silica gel 60®, 40-63 µm). If no solvent is stated an aqueous solution was prepared with purified water. Mixtures of two or more solvents are specified as "solvent A"/"solvent B"; 67:33; (*v*/*v*), meaning that 100 mL of the respective mixture consists of 67 mL of "solvent A" and 33 mL of "solvent B". The uncorrected melting points were determined using a Büchi Melting Point M-565 apparatus. **Nuclear Magnetic Resonance Spectroscopy (NMR):** Proton (^{1}H), boron (^{11}B), carbon (^{13}C) and fluorine (^{19}F {^{1}H}) NMR spectra were recorded either on a Bruker Avance III HD 400 MHz at a frequency of 400 MHz (^{1}H), 128 MHz (^{11}B), 101 MHz (^{13}C) and 377 MHz (^{19}F), a Varian/Agilent Mecury-plus-400 at a frequency of 400 MHz (^{1}H), 128 MHz (^{11}B), 101 MHz (^{13}C) and 376 MHz (^{19}F) or a Varian/Agilent Mecury-plus-300 at a frequency of 300 MHz (^{1}H), 96 MHz (^{11}B), 75 MHz (^{13}C) and 282 MHz (^{19}F). The chemical shifts are given in parts per million (ppm). As solvents deuterated chloroform

(chloroform-*d*), deuterated methanol (methanol-d_4) and deuterated dimethyl sulfoxide (DMSO-d_6) were used. The residual solvent signal (chloroform-*d*: ^{1}H NMR: 7.26 ppm, ^{13}C NMR: 77.1 ppm; DMSO-d_6: ^{1}H NMR: 2.50 ppm, ^{13}C NMR: 39.52 ppm; Methanol-d_4: ^{1}H NMR: 3.31 ppm, 4.87 ppm, ^{13}C NMR: 49.00 ppm) was used for calibration. The multiplicity of each signal is reported as singlet (s), doublet (d), triplet (t), quartet (q), multiplet (m) or combinations thereof. Multiplicities and coupling constants are reported as measured and might disagree with the expected values. In the case of the peptoid compounds **8a-c**, **9a-c**, and **10a-c**, and due to the well-known phenomenon of *cis*/*trans*-amide bond rotamerism in peptoids[3,4], certain ^{1}H and ^{13}C NMR signals can occur as two distinct sets of signals. In this case, the assigned signals correspond to the major rotamer conformation. ^{19}F NMR spectra were recorded proton-decoupled if not stated otherwise. **Mass Spectrometry:** High resolution electrospray ionisation mass spectra (HRMS-ESI) were acquired either with a *Bruker Daltonik GmbH* micrOTOF coupled to a an *LC Packings* Ultimate HPLC system and controlled by micrOTOFControl3.4 and HyStar 3.2-LC/MS or with a *Bruker Daltonik GmbH* ESI-qTOF Impact II coupled to a *Dionex* UltiMateTM 3000 UHPLC system and controlled by micrOTOFControl 4.0 und HyStar 3.2-LC/MS. Low resolution electrospray ionisation mass spectra (LRMS-ESI) were acquired with an *Advion* expression® compact mass spectrometer (CMS) coupled with an automated TLC plate reader Plate Express® (*Advion*). **High Performance Liquid Chromatography (HPLC)**: For analytical purposes either a *Gynkotek* Gina 50 HPLC system (Detector: *Gynkotek* UVD340U, Pump: *Dionex* P680 HPLC pump, column oven: *Dionex* STH 585) with a Nucleodur 100-5 C18 ec (250 x 4.6 mm, *Macherey Nagel*) column, a *Thermo Fisher Scientific* UltiMate™ 3000 UHPLC system with a Nucleodur 100-5 C18 (250 x 4.6 mm, *Macherey Nagel*). A flow rate of 1 mL/min and a temperature of 25 °C were set. A *Varian* ProStar system with a Nucleodur 110-5 C18 HTec

(150 x 32 mm, *Macherey Nagel*) column with 14 mL/min was used for preparative purposes. Detection was implemented by UV absorption measurement at a wavelength of λ = 220 nm and λ = 250 nm. Bidest. H_2O (A) and MeCN (B) were used as eluents with an addition of 0.1% TFA for eluent A. For analytical purposes after column equilibration for 5 min a linear gradient from 5% A to 95% B in 7 min followed by an isocratic regime of 95% B for 10 min. For preparative purposes after column equilibration for 5 min a linear gradient from 5% A to 95% B in 15 min followed by an isocratic regime of 95% B for 10 min was used. **Purity:** The purity of all final compounds was 95% or higher. Purity was determined via HPLC at 250 nm using the protocols described above, if not stated otherwise.

The following compounds were synthesized according to literature and used without further purification: *tert*-butyl (4-bromo-2-nitrophenyl)carbamate[5], *tert*-butyl (3-nitro-[1,1'-biphenyl]-4-yl)carbamate[6], methyl 4-(aminomethyl)benzoate hydrochloride[7], methyl 4-({*N*-[2-(benzylamino)-2-oxoethyl]-4-(dimethylamino)benzamido} methyl)benzoate[8], methyl 4-({*N*-[2-(cyclohexylamino)-2-oxoethyl]-4-(dimethylamino)benzamido}methyl)benzoate[9], methyl 4-({*N*-[2-(*tert*-butylamino)-2-oxoethyl]-4-(dimethylamino)benzamido}methyl)benzoate[9].

***tert*-Butyl (2-nitro-4-[4,4,5,5-tetramethyl-1,3,2-dioxaborolan-2-yl]phenyl)carbamate (2).** Compound **2** was synthesized via MIYAURA-borylation based on literature.[10] Under argon and using standard SCHLENK-techniques *tert*-butyl (4-bromo-2-nitrophenyl)carbamate[5] (**1**) (2.00 g, 6.30 mmol, 1.0 equiv.) was dissolved in anhydrous DMF (15 mL). Anhydrous KOAc (1.26 g, 12.6 mmol, 2.0 equiv.) was added and the mixture was purged with argon followed by addition of $PdCl_2$(dppf) (230 mg, 0.32 mmol, 0.05 equiv.) and bis(pinacolato)diboron (1.93 g, 7.57 mmol, 1.2 equiv.). Subsequently, the reaction mixture was heated to 80°C for 18 h. The reaction was monitored by TLC (cyclohexane/EtOAc; 85:15; *v*/*v*) using alizarin staining[11] for detection

(366nm). Upon completion the reaction mixture was diluted with EtOAc (500 mL), filtered through Celite® and the resulting organic layer was washed with 1 M HCl (2 x 100 mL), half saturated $NaHCO_3$-solution (2 x 100 mL) and brine (2 x 150 mL), dried over Na_2SO_4, filtered before the solvent was evaporated. For the purification of the crude product by flash column chromatography (cyclohexane/EtOAc; gradient: 95:5 → 80:20; *v/v*) SiO_2 was pre-conditioned with H_3BO_3 (5 %; *m/m*). Flash column chromatography afforded the desired product (**2**) as yellow solid. (768 mg, 2.11 mmol, 33 %); **R_f** = 0.57 (cyclohexane/EtOAc; 85:15; *v/v*); **^{1}H NMR** (400 MHz, Chloroform-*d*) δ = 9.79 (s, 1H), 8.61 (d, *J* = 1.5 Hz, 1H), 8.55 (d, *J* = 8.5 Hz, 1H), 7.97 (dd, *J* = 8.5, 1.5 Hz, 1H), 1.54 (s, 9H), 1.35 (s, 12H); **^{11}B NMR** (128 MHz, Chloroform-*d*) δ = 31.2; **HRMS-ESI (*m/z*):** $[M+Na]^+$ calcd for $C_{17}H_{25}BN_2O_6$: 387.1698, found: 387.1690. Analytical data matches with literature.[10]

***tert*-Butyl (2-nitro-4-[pyridin-2-yl]phenyl)carbamate (3f).** Under argon and using standard SCHLENK-techniques *tert*-butyl (2-nitro-4-[4,4,5,5-tetramethyl-1,3,2-dioxaborolan-2-yl]phenyl)carbamate (**2**) (300 mg, 0.83 mmol, 1.0 equiv.) was dissolved in anhydrous DMF (4 mL). Anhydrous Cs_2CO_3 (540 mg, 1.66 mmol, 2.0 equiv.) was added and the mixture was purged with argon followed by addition of $PdCl_2$(dppf) (31.0 mg, 42.0 µmol, 0.05 equiv.) and 2-bromopyridine (385 mg, 240 µL, 2.47 mmol, 3.0 equiv.). Subsequently, the reaction mixture was heated to 85 °C for 16 h under argon. Upon completion, indicated by TLC, the reaction mixture was diluted with EtOAc (100 mL) and the organic layer was washed with half saturated $NaHCO_3$-solution (2 x 50 mL), H_2O (2 x 50 mL) and brine (2 x 50 mL), dried over Na_2SO_4, filtered and evaporated under reduced pressure. Purification of the crude product by flash column chromatography (cyclohexane/EtOAc; 90:10; *v/v*) afforded the desired products as yellow solid (137 mg, 0.44 mmol, 53 %); **mp.** 197.7 – 198.8 °C; **R_f** = 0.28 (cyclohexane/EtOAc; 90:10; *v/v*);

^{1}H NMR (400 MHz, Chloroform-*d*) δ = 9.75 (s, 1H), 8.87 (d, *J* = 2.2 Hz, 1H), 8.74 – 8.65 (m, 2H), 8.28 (ddd, *J* = 9.0, 2.2, 0.6 Hz, 1H), 7.83 – 7.72 (m, 2H), 7.30 – 7.25 (m, 1H), 1.56 (s, 9H); ^{13}C NMR (101 MHz, Chloroform-*d*) δ 154.5, 152.2, 149.9, 137.3, 136.4, 136.3, 133.9, 133.2, 124.1, 122.9, 121.0, 120.1, 82.2, 28.4; **HRMS-ESI (*m/z*):** $[M+H]^+$ calcd for $C_{16}H_{17}N_3O_4$: 316.1292, found: 316.1296.

General procedure I for the synthesis of the compounds 3a-e and 3g-i. Compounds **3a-e** and **3g-i** were prepared based on literature.[6] To a solution of *tert*-butyl (4-bromo-2-nitrophenyl)carbamate (**1**) (600 mg, 1.90 mmol, 1.0 equiv.) in toluene (5.0 mL) Na_2CO_3-solution (3.0 mL, 2 M) and the respective boronic acid (2.10 mmol, 1.1 equiv.), dissolved in EtOH (3.0 mL), was added. The reaction mixture was exposed to ultrasonication for 10 minutes under argon and subsequently, $Pd(PPh_3)_4$ (110 mg, 95.0 nmol, 0.05 equiv.) was added. This solution was refluxed for 16 h under argon. Upon completion, indicated by TLC, the reaction mixture was diluted with EtOAc (50 mL) and the organic layer was washed with half saturated $NaHCO_3$-solution (2 x 30 mL, all compounds), 1 M HCl (2 x 30 mL, **3a-c** and **3g-i**), H_2O (2 x 30 mL, all compounds) and brine (2 x 30 mL, all compounds), dried over Na_2SO_4, filtered and evaporated. Purification of the crude product afforded the desired products as yellow or orange solids.

***tert*-Butyl (3-nitro-[1,1'-biphenyl]-4-yl)carbamate (3a).** Synthesis according to General Procedure **I**. Flash column chromatography (cyclohexane/EtOAc; gradient: 99:1 → 95:5; *v/v*) afforded the desired product as an orange solid (430 mg, 1.37 mmol, 72 %); **R_f** = 0.52 (cyclohexane/EtOAc; 95:5; *v/v*); **^{1}H NMR** (400 MHz, Chloroform-*d*) δ = 9.67 (s, 1H), 8.63 (d, *J* = 8.9 Hz, 1H), 8.42 (d, *J* = 2.3 Hz, 1H), 7.85 (dd, *J* = 8.9, 2.3 Hz, 1H), 7.63 – 7.55 (m, 2H), 7.52 – 7.43 (m, 2H), 7.42 – 7.36 (m, 1H), 1.56 (s, 9H); **HRMS-ESI (*m/z*):** $[2M+Na]^+$ calcd for $C_{17}H_{18}N_2O_4$: 651.2425, found: 651.2410. Analytical data matches with literature.[6]

***tert*-Butyl (4'-fluoro-3-nitro-[1,1'-biphenyl]-4-yl)carbamate (3b).** Synthesis according to General Procedure **I**. Flash column chromatography (cyclohexane/EtOAc; gradient: 98:2 → 95:5; *v/v*) afforded the desired product as an orange solid (421 mg, 1.25 mmol, 66 %); **mp.** 158.0 – 159.2°C; **R$_f$** = 0.50 (cyclohexane/EtOAc; 95:5; *v/v*); **^{1}H NMR** (300 MHz, Chloroform-*d*) δ = 9.66 (s, 1H), 8.63 (dt, *J* = 8.9, 0.4 Hz, 1H), 8.37 (dd, *J* = 2.3, 0.4 Hz, 1H), 7.79 (ddd, *J* = 8.9, 2.3, 0.5 Hz, 1H), 7.59 – 7.50 (m, 2H), 7.21 – 7.10 (m, 2H), 1.56 (d, *J* = 0.4 Hz, 9H); **^{13}C NMR** (101 MHz, Chloroform-*d*) δ = 163.0 (d, *J* = 248.0 Hz), 152.3, 136.3, 135.1, 134.5 (d, *J* = 3.5 Hz), 134.3, 134.1, 128.5 (d, *J* = 8.3 Hz), 123.7, 121.4, 116.2 (d, *J* = 21.6 Hz), 82.2, 28.4; **^{19}F NMR** (282 MHz, Chloroform-*d*) δ = -114.2; **HRMS-ESI (*m/z*):** [M+Na]$^+$ calcd for $C_{17}H_{17}FN_2O_4$: 355.1065, found: 355.1075.

***tert*-Butyl (2-nitro-4-[thiophen-2-yl]phenyl)carbamate (3c).** Synthesis according to General Procedure **I**. Flash column chromatography (cyclohexane/EtOAc; gradient: 97:3 → 95:5; *v/v*) afforded the desired product as an orange solid (400 mg, 1.25 mmol, 66 %); **mp.** 128.8 – 130.9 °C; **R$_f$** = 0.59 (cyclohexane/EtOAc; 95:5; *v/v*); **^{1}H NMR** (400 MHz, Chloroform-*d*) δ = 9.65 (s, 1H), 8.59 (d, *J* = 8.9 Hz, 1H), 8.40 (d, *J* = 2.2 Hz, 1H), 7.81 (ddd, *J* = 8.9, 2.3, 0.6 Hz, 1H), 7.37 – 7.29 (m, 2H), 7.10 (dd, *J* = 5.1, 3.6 Hz, 1H), 1.56 (s, 9H); **^{13}C NMR** (101 MHz, Chloroform-*d*) δ = 152.3, 141.5, 136.2, 135.0, 133.1, 128.9, 128.5, 125.8, 124.1, 122.5, 121.3, 82.2, 28.4; **HRMS-ESI (*m/z*):** [M+Na]$^+$ calcd for $C_{15}H_{16}N_2O_4S$: 343.0723, found: 343.0730.

***tert*-Butyl (2-nitro-4-[pyridin-4-yl]phenyl)carbamate (3d).** Synthesis according to General Procedure **I**. Flash column chromatography (cyclohexane/EtOAc; gradient: 67:33 → 50:50; *v/v*) afforded the desired product as yellow solid (355 mg, 1.35 mmol, 71 %); **mp.** 103.8 – 105.9 °C; **R$_f$** = 0.21 (cyclohexane/EtOAc; 67:33; *v/v*); **^{1}H NMR** (300 MHz, Chloroform-*d*) δ = 9.77 – 9.70 (m, 1H), 8.75 – 8.68 (m, 3H), 8.50 (dd, *J* = 2.3, 0.4 Hz, 1H), 7.89 (ddd, *J* = 8.9, 2.3, 0.6 Hz, 1H),

7.56 – 7.47 (m, 2H), 1.56 (s, 9H); **^{13}C NMR** (75 MHz, Chloroform-*d*) δ = 152.1, 150.6, 145.5, 136.6, 136.2, 133.9, 131.8, 124.2, 121.6, 121.1, 82.5, 28.3; **HRMS-ESI (*m/z*):** $[M+H]^+$ calcd for $C_{16}H_{17}N_3O_4$: 316.1292, found: 316.1305.

***tert*-Butyl (2-nitro-4-[pyridin-3-yl]phenyl)carbamate (3e).** Synthesis according to General Procedure **I**. Flash column chromatography (cyclohexane/EtOAc; 67:33; *v/v*) afforded the desired product as yellow solid (292 mg, 0.93 mmol, 49 %); **mp.** 110.9 – 112.3 °C; **R_f** = 0.36 (cyclohexane/EtOAc; 67:33; *v/v*); **^{1}H NMR** (400 MHz, Chloroform-*d*) δ = 9.71 (s, 1H), 8.85 (dd, *J* = 2.4, 0.8 Hz, 1H), 8.70 (d, *J* = 8.9 Hz, 1H), 8.64 (dd, *J* = 4.8, 1.6 Hz, 1H), 8.42 (d, *J* = 2.2 Hz, 1H), 7.88 (dddd, *J* = 7.9, 2.3, 1.7, 0.4 Hz, 1H), 7.84 (ddd, *J* = 8.9, 2.3, 0.5 Hz, 1H), 7.44 – 7.36 (m, 1H), 1.56 (s, 9H); **^{13}C NMR** (101 MHz, Chloroform-*d*) δ = 152.2, 149.4, 148.0, 136.3, 135.8, 134.1, 134.1, 134.0, 131.8, 124.1, 123.9, 121.6, 82.3, 28.3; **HRMS-ESI (*m/z*):** $[M+H]^+$ calcd for $C_{16}H_{17}N_3O_4$: 316.1292, found: 316.1296.

***tert*-Butyl (4-[6-fluoropyridin-3-yl]-2-nitrophenyl)carbamate (3g).** Synthesis according to General Procedure **I**. Flash column chromatography (cyclohexane/EtOAc; 95:5; *v/v*) afforded the desired product as yellow solid (466 mg, 1.44 mmol, 76 %); **mp.** 147.5 – 148.1 °C; **R_f** = 0.25 (cyclohexane/EtOAc; 95:5; *v/v*); **^{1}H NMR** (400 MHz, Chloroform-*d*) δ = 9.70 (s, 1H), 8.71 (d, *J* = 8.9 Hz, 1H), 8.44 (d, *J* = 2.6 Hz, 1H), 8.38 (d, *J* = 2.3 Hz, 1H), 7.98 (ddd, *J* = 8.5, 7.4, 2.7 Hz, 1H), 7.79 (dd, *J* = 8.9, 2.3 Hz, 1H), 7.05 (dd, *J* = 8.5, 3.1 Hz, 1H), 1.56 (s, 9H); **^{13}C NMR** (101 MHz, Chloroform-*d*) δ = 163.6 (d, *J* = 240.8 Hz), 152.2, 145.8 (d, *J* = 15.1 Hz), 139.5 (d, *J* = 8.1 Hz), 136.3, 135.9, 134.0, 132.3, 130.6, 124.0, 121.8, 110.0 (d, *J* = 37.8 Hz), 82.4, 28.4; **^{19}F NMR** (377 MHz, Chloroform-*d*) δ = -68.9; **HRMS-ESI (*m/z*):** $[M+H]^+$ calcd for $C_{16}H_{16}FN_3O_4$: 334.1198, found: 334.1197.

***tert*-Butyl (4-[2-fluoropyridin-3-yl]-2-nitrophenyl)carbamate (3h).** Synthesis according to General Procedure **I**. Flash column chromatography (cyclohexane/EtOAc; 90:10; *v/v*) afforded the desired product as yellow solid (166 mg, 0.50 mmol, 55 %); **mp.** 84.1 – 85.6 °C; $\mathbf{R_f}$ = 0.21 (cyclohexane/EtOAc; 90:10; *v/v*); **^{1}H NMR** (300 MHz, Chloroform-*d*) δ = 9.72 (s, 1H), 8.70 (dt, *J* = 9.0, 0.3 Hz, 1H), 8.43 (ddd, *J* = 2.3, 1.0, 0.4 Hz, 1H), 8.24 (ddd, *J* = 4.9, 1.9, 1.3 Hz, 1H), 7.90 (ddd, *J* = 9.9, 7.5, 2.0 Hz, 1H), 7.84 (dddd, *J* = 8.9, 2.3, 1.7, 0.5 Hz, 1H), 7.32 (ddd, *J* = 7.4, 4.8, 1.8 Hz, 1H), 1.56 (s, 9H); **^{13}C NMR** (75 MHz, Chloroform-*d*) δ = 176.5, 160.4 (d, *J* = 240.3 Hz), 152.2, 147.3 (d, *J* = 14.9 Hz), 140.3 (d, *J* = 4.0 Hz), 136.1, 136.0, 135.9 (d, *J* = 3.9 Hz), 127.7 (d, *J* = 5.2 Hz), 126.0 (d, *J* = 3.5 Hz), 122.2 (d, *J* = 4.6 Hz), 121.2 82.4, 28.3; **^{19}F NMR** (282 MHz, Chloroform-*d*) δ = -70.6; **HRMS-ESI (*m/z*):** $[M–H]^-$ calcd for $C_{16}H_{16}FN_3O_4$: 332.1052, found: 332.1042.

***tert*-Butyl (4-[2-fluoropyridin-4-yl]-2-nitrophenyl)carbamate (3i).** Synthesis according to General Procedure **I**. Flash column chromatography (cyclohexane/EtOAc; 95:5; *v/v*) afforded the desired product as yellow solid (434 mg, 1.31 mmol, 69 %); **mp.** 131.0 – 132.8 °C; $\mathbf{R_f}$ = 0.28 (cyclohexane/EtOAc; 95:5; *v/v*); **^{1}H NMR** (400 MHz, Chloroform-*d*) δ = 9.76 (s, 1H), 8.75 (d, *J* = 8.9 Hz, 1H), 8.49 (d, *J* = 2.3 Hz, 1H), 8.34 – 8.27 (m, 1H), 7.88 (ddd, *J* = 9.0, 2.3, 0.6 Hz, 1H), 7.41 (dt, *J* = 5.3, 1.7 Hz, 1H), 7.14 (td, *J* = 1.6, 0.6 Hz, 1H), 1.56 (s, 9H); **^{13}C NMR** (101 MHz, Chloroform-*d*) δ = 164.7 (d, *J* = 239.0 Hz), 152.1, 151.0 (d, *J* = 8.4 Hz), 148.7 (d, *J* = 15.6 Hz), 137.1, 136.1, 133.9, 130.6 (d, *J* = 3.5 Hz), 124.3, 121.6, 119.0 (d, *J* = 4.1 Hz), 106.9 (d, *J* = 38.5 Hz), 82.6, 28.3; **^{19}F NMR** (377 MHz, Chloroform-*d*) δ = - 67.1; **HRMS-ESI (*m/z*):** $[M+H]^+$ calcd for $C_{16}H_{16}FN_3O_4$: 334.1198, found: 334.1195.

General procedure II for the synthesis of the compounds 4a-i. The respective *tert*-butyl (2-nitrophenyl)carbamate derivative **3a-i** (1.0 equiv.) was dissolved in MeOH/DCM (75:25; *v/v*,

40 mL/mmol) and Pd(C) (5%) (0.1 equiv.) was added. The reaction mixture was purged with hydrogen three times and left stirring at room temperature 2 – 16 h. Upon completion, indicated by TLC, the organic layer was filtered through Celite®. The Celite® pad was washed thoroughly with MeOH/DCM (50:50; *v/v*) or EtOAc and the resulting organic layer was evaporated, affording the respective product as white, greyish or yellow solid.

***tert*-Butyl (3-amino-[1,1'-biphenyl]-4-yl)carbamate (4a).** Starting from **3a** (353 mg, 1.11 mmol, 1.0 equiv.) the reaction according to General Procedure **II** provided **4a** as white crystals (317 mg, 1.11 mmol, quant.); **mp.** 152.4 – 153.3 °C; **^{1}H NMR** (400 MHz, Chloroform-*d*) δ = 7.57 – 7.49 (m, 2H), 7.44 – 7.37 (m, 2H), 7.35 (d, *J* = 8.7 Hz, 1H), 7.34 – 7.29 (m, 1H), 7.08 – 7.01 (m, 2H), 6.32 (s, 1H), 3.83 (s, 2H), 1.53 (s, 9H); **^{13}C NMR** (101 MHz, Chloroform-*d*) δ = 154.0, 140.9, 139.4, 128.8, 127.3, 127.1, 125.1, 125.0, 124.6, 119.2, 116.8, 80.9, 28.5; **HRMS-ESI (*m/z*):** $[M+H]^+$ calcd for $C_{17}H_{20}N_2O_2$: 285.1598, found: 285.1605.

***tert*-Butyl (3-amino-4'-fluoro-[1,1'-biphenyl]-4-yl)carbamate (4b).** Starting from **3b** (340 mg, 1.02 mmol, 1.0 equiv.) the reaction according to General Procedure **II** provided **4b** as white crystals (305 mg, 1.01 mmol, 99 %); **mp.** 186.0 – 187.8 °C; **^{1}H NMR** (400 MHz, Chloroform-*d*) δ = 7.52 – 7.42 (m, 2H), 7.33 (d, *J* = 8.0 Hz, 1H), 7.14 – 7.03 (m, 2H), 6.97 (d, *J* = 7.5 Hz, 2H), 6.29 (s, 1H), 3.92 (s, 2H), 1.53 (s, 9H); **^{13}C NMR** (101 MHz, Chloroform-*d*) δ = 162.4 (d, *J* = 246.1 Hz), 153.8, 139.7, 138.3, 136.9 (d, *J* = 2.9 Hz), 128.5 (d, *J* = 8.0 Hz), 125.0, 124.3, 118.7, 116.3, 115.5 (d, *J* = 21.4 Hz), 80.8, 28.4; **^{19}F NMR** (377 MHz, Chloroform-*d*) δ = -116.0; **HRMS-ESI (*m/z*):** $[M+H]^+$ calcd for $C_{17}H_{19}FN_2O_2$: 303.1503, found: 303.1512.

***tert*-Butyl (2-amino-4-[thiophen-2-yl]phenyl)carbamate (4c).** Starting from **3c** (340 mg, 1.06 mmol, 1.0 equiv.) the reaction according to General Procedure **II** provided **4c** as greyish orange solid (308 mg, 1.06 mmol, quant.); **mp.** 161.3 – 162.9 °C; **^{1}H NMR** (400 MHz, Chloroform-*d*) δ

= 7.30 (d, J = 8.3 Hz, 1H), 7.23 (td, J = 4.6, 3.9, 1.2 Hz, 2H), 7.08 – 7.03 (m, 2H), 7.02 (d, J = 2.0 Hz, 1H), 6.23 (s, 1H), 3.80 (s, 2H), 1.52 (s, 9H); **^{13}C NMR** (101 MHz, Chloroform-*d*) δ = 153.7, 144.1, 140.0, 132.3, 127.9, 124.8, 124.7, 124.4, 122.8, 117.5, 115.0, 80.7, 28.3; **HRMS-ESI (*m/z*):** $[M+H]^+$ calcd for $C_{15}H_{18}N_2O_2S$: 291.1162, found: 291.1167.

***tert*-Butyl (2-amino-4-[pyridin-4-yl]phenyl)carbamate (4d).** Starting from **3d** (275 mg, 0.87 mmol, 1.0 equiv.) the reaction according to General Procedure **II** provided **4d** as pale-yellow solid (250 mg, 0.87 mmol, quant.); **mp.** 178.9 – 182.1 °C (decomp.); **^{1}H NMR** (400 MHz, Chloroform-*d*) δ = 8.67 – 8.58 (m, 2H), 7.52 – 7.43 (m, 3H), 7.08 (dd, J = 8.2, 2.1 Hz, 1H), 7.05 (d, J = 2.0 Hz, 1H), 6.37 (s, 1H), 3.60 (s, 2H), 1.53 (s, 9H); **^{13}C NMR** (101 MHz, Chloroform-*d*) δ = 153.7, 149.9, 148.4, 140.0, 135.5, 126.4, 124.8, 121.6, 118.6, 116.2, 81.1, 77.5, 77.2, 76.8, 28.5; **HRMS-ESI (*m/z*):** $[M+H]^+$ calcd for $C_{16}H_{19}N_3O_2$: 286.1550, found: 286.1562.

***tert*-Butyl (2-amino-4-[pyridin-3-yl]phenyl)carbamate (4e).** Starting from **3e** (872 mg, 2.77 mmol, 1.0 equiv.) the reaction according to General Procedure **II** provided **4e** as pale-yellow solid (637 mg, 1.53 mmol, 81 %); **mp.** 172.8 °C (decomp.); **^{1}H NMR** (300 MHz, Chloroform-*d*) δ = 8.79 (dd, J = 2.4, 0.9 Hz, 1H), 8.55 (dd, J = 4.8, 1.7 Hz, 1H), 7.82 (ddd, J = 7.9, 2.4, 1.6 Hz, 1H), 7.41 (d, J = 8.0 Hz, 1H), 7.33 (ddd, J = 7.9, 4.8, 0.9 Hz, 1H), 7.05 – 6.93 (m, 2H), 6.39 (s, 1H), 3.81 (s, 2H), 1.52 (s, 9H); **^{13}C NMR** (101 MHz, Chloroform-*d*) δ = 153.9, 148.1, 148.0, 140.3, 136.6, 135.5, 134.5, 125.2, 125.1, 123.7, 118.6, 116.2, 81.0, 28.5; **HRMS-ESI (*m/z*):** $[M+H]^+$ calcd for $C_{16}H_{19}N_3O_2$: 286.1566, found: 286.1562.

***tert*-Butyl (2-amino-4-[pyridin-2-yl]phenyl)carbamate (4f).** Starting from **3f** (120 mg, 0.38 mmol, 1.0 equiv.) the reaction according to General Procedure **II** provided **4f** as yellow solid (108 mg, 0.38 mmol, quant.); **mp.** 106.2 – 107.6 °C; **^{1}H NMR** (400 MHz, Chloroform-*d*) δ = 8.66 (dt, J = 4.9, 1.3 Hz, 1H), 7.74 (td, J = 7.7, 1.8 Hz, 1H), 7.69 (dt, J = 8.1, 1.2 Hz, 1H), 7.54 (d,

J = 2.0 Hz, 1H), 7.49 (d, J = 8.3 Hz, 1H), 7.37 (dd, J = 8.3, 2.0 Hz, 1H), 7.22 (ddd, J = 7.3, 4.8, 1.3 Hz, 1H), 6.44 (s, 1H), 3.84 (s, 2H), 1.52 (s, 9H); **^{13}C NMR** (101 MHz, Chloroform-*d*) δ = 156.8, 153.7, 149.1, 139.3, 137.4, 135.9, 126.9, 123.8, 122.1, 120.7, 118.7, 116.6, 80.9, 28.5; **HRMS-ESI (*m/z*):** $[M+H]^+$ calcd for $C_{16}H_{19}N_3O_2$: 286.1554, found: 286.1562.

***tert*-Butyl (2-amino-4-[6-fluoropyridin-3-yl]phenyl)carbamate (4g).** Starting from **3g** (420 mg, 1.26 mmol, 1.0 equiv.) the reaction according to General Procedure **II** provided **4g** as pale pink crystals (335 mg, 1.11 mmol, 88 %); **mp.** 165.1 – 167.2 °C; **^{1}H NMR** (400 MHz, Chloroform-*d*) δ = 8.35 (d, J = 2.5 Hz, 1H), 7.89 (td, J = 8.0, 2.6 Hz, 1H), 7.40 (d, J = 8.3 Hz, 1H), 7.00 – 6.91 (m, 3H), 6.33 (s, 1H), 3.91 (s, 2H), 1.53 (s, 9H); **^{13}C NMR** (101 MHz, Chloroform-*d*) δ = 163.1 (d, J = 238.8 Hz), 153.9, 145.7 (d, J = 14.7 Hz), 140.1, 139.6 (d, J = 7.8 Hz), 134.6, 125.3, 118.7, 116.2, 109.4 (d, J = 37.5 Hz), 81.1, 28.5; **^{19}F NMR** (377 MHz, Chloroform-*d*) δ = - 70.8; **HRMS-ESI (*m/z*):** $[M+H]^+$ calcd for $C_{16}H_{18}FN_3O_2$: 304.1456, found: 304.1460.

***tert*-Butyl (2-amino-4-[2-fluoropyridin-3-yl]phenyl)carbamate (4h).** Starting from **3h** (250 mg, 0.75 mmol, 1.0 equiv.) the reaction according to General Procedure **II** provided **4h** as grey crystals (214 mg, 0.71 mmol, 95 %); **mp.** 146.1 – 146.9 °C (decomp.); **^{1}H NMR** (400 MHz, Chloroform-*d*) δ = 8.18 – 8.11 (m, 1H), 7.82 (ddd, J = 9.6, 7.4, 2.0 Hz, 1H), 7.43 (d, J = 8.2 Hz, 1H), 7.23 (ddd, J = 7.4, 4.8, 1.8 Hz, 1H), 7.05 (t, J = 1.9 Hz, 1H), 7.01 (dt, J = 8.2, 1.7 Hz, 1H), 6.44 (s, 1H), 3.88 (s, 2H), 1.52 (s, 9H); **^{13}C NMR** (101 MHz, Chloroform-*d*) δ = 160.5 (d, J = 240.4 Hz), 153.8, 146.2 (d, J = 14.7 Hz), 140.6 (d, J = 4.5 Hz), 138.8, 131.5 (d, J = 5.0 Hz), 126.0, 124.6, 123.6 (d, J = 28.0 Hz), 121.9 (d, J = 4.3 Hz), 120.9, 118.5, 81.1, 28.5; **^{19}F NMR** (376 MHz, Chloroform-*d*) δ = - 70.6; **HRMS-ESI (*m/z*):** $[M+H]^+$ calcd for $C_{16}H_{18}FN_3O_2$: 304.1456, found: 304.1460.

***tert*-Butyl (2-amino-4-[2-fluoropyridin-4-yl]phenyl)carbamate (4i).** Starting from **3i** (1.05 g, 3.46 mmol, 1.0 equiv.) the reaction according to General Procedure **II** provided **4i** as grey solid (913 mg, 3.01 mmol, 87 %); **mp.** 161.8 – 163.4 °C (decomp.); **^{1}H NMR** (400 MHz, Chloroform-*d*) δ = 8.21 (d, *J* = 5.3 Hz, 1H), 7.48 (d, *J* = 8.2 Hz, 1H), 7.33 (dt, *J* = 5.4, 1.7 Hz, 1H), 7.11 – 7.00 (m, 3H), 6.36 (s, 1H), 3.85 (s, 2H), 1.53 (s, 9H); **^{13}C NMR** (101 MHz, Chloroform-*d*) δ = 164.6 (d, *J* = 237.7 Hz), 153.8 (d, *J* = 8.3 Hz), 153.6, 148.0 (d, *J* = 15.6 Hz), 139.9, 134.5 (d, *J* = 3.4 Hz), 126.9, 124.6, 119.4 (d, *J* = 3.9 Hz), 118.7, 116.2, 106.8 (d, *J* = 38.0 Hz), 81.2, 28.5; **^{19}F NMR** (377 MHz, Chloroform-*d*) δ = - 68.4; **HRMS-ESI (*m/z*):** $[M+H]^+$ calcd for $C_{16}H_{18}FN_3O_2$: 304.1456, found: 304.1465.

General procedure III for the synthesis of the compounds 5a-i. To a suspension of 4-acetamidobenzoic acid (100 mg, 0.56 mmol, 1.2 equiv.) in dry DMF (2 mL), EDC•HCl (270 mg, 1.41 mmol, 3.0 equiv.), HOBt•H_2O (215 mg, 1.41 mmol, 3.0 equiv.) and DIPEA (305 mg, 400 µL, 2.35 mmol, 5.0 equiv.) were added and the reaction mixture was allowed to stir at room temperature until a clear solution had formed. Subsequently, the respective amine **4a-i** (0.47 mmol, 1.0 equiv.), dissolved in dry DCM (1 mL), was added and the reaction mixture was allowed to stir at room temperature for 16 h. Upon completion, the reaction mixture was diluted with EtOAc (75 mL) and the organic layer was washed with half saturated $NaHCO_3$-solution (2 x 40 mL, all compounds), 1 M HCl (2 x 40 mL, **5a-c** and **5g-i**), H_2O (2 x 40 mL, all compounds) and brine (2 x 40 mL, all compounds), dried over Na_2SO_4, filtered and evaporated. Purification of the crude product afforded the desired products as pale-yellow or white solids.

***tert*-Butyl (3-[4-acetamidobenzamido]-[1,1'-biphenyl]-4-yl)carbamate (5a).** Synthesis according to General Procedure **III**. Flash column chromatography (DCM/MeOH/TEA; 98.5:1.5:0.1; *v/v/v*) afforded the desired product as a white solid (141 mg, 0.32 mmol, 67 %);

mp. > 227.4 °C (decomp.); **R$_f$** = 0.29 (DCM/MeOH/TEA; 95:5:0.1; *v/v/v*); **^{1}H NMR** (400 MHz, DMSO-*d*$_6$) δ = 10.25 (s, 1H), 9.84 (s, 1H), 8.77 (s, 1H), 7.94 (d, *J* = 8.6 Hz, 2H), 7.85 (d, *J* = 2.2 Hz, 1H), 7.74 (d, *J* = 8.7 Hz, 2H), 7.68 – 7.61 (m, 3H), 7.51 (dd, *J* = 8.5, 2.2 Hz, 1H), 7.46 (t, *J* = 7.7 Hz, 2H), 7.36 (t, *J* = 7.3 Hz, 1H), 2.10 (s, 3H), 1.47 (s, 9H); **^{13}C NMR** (101 MHz, DMSO-*d*$_6$) δ = 168.8, 164.9, 153.4, 142.6, 139.3, 135.9, 131.1, 130.1, 129.0, 128.6, 128.3, 127.3, 126.4, 124.1, 124.1, 123.7, 118.2, 79.8, 28.0, 24.1; **HRMS-ESI (*m/z*):** [M+Na]$^+$ calcd for $C_{26}H_{27}N_3O_4$: 468.1894, found: 468.1902.

***tert*-Butyl (3-[4-acetamidobenzamido]-4'-fluoro-[1,1'-biphenyl]-4-yl)carbamate (5b).** Synthesis according to General Procedure **III**. Flash column chromatography (DCM/MeOH/TEA; gradient; 99.5:0.5:0.1 → 98.5:1.5:0.1; *v/v/v*) afforded the desired product as a white solid (122 mg, 0.26 mmol, 56 %); **mp.** > 221.8 °C (decomp.); **R$_f$** = 0.25 (DCM/MeOH/TEA; 97.5:2.5:0.1; *v/v/v*); **^{1}H NMR** (400 MHz, DMSO-*d*$_6$) δ = 10.24 (s, 1H), 9.83 (s, 1H), 8.77 (s, 1H), 7.94 (d, *J* = 8.7 Hz, 2H), 7.83 (d, *J* = 2.2 Hz, 1H), 7.74 (d, *J* = 8.7 Hz, 2H), 7.71 – 7.66 (m, 2H), 7.63 (d, *J* = 8.5 Hz, 1H), 7.49 (dd, *J* = 8.5, 2.2 Hz, 1H), 7.34 – 7.24 (m, 2H), 2.09 (s, 3H), 1.47 (s, 9H); **^{13}C NMR** (75 MHz, DMSO-*d*$_6$) δ = 168.8, 164.9, 161.8 (d, *J* = 244.2 Hz), 153.4, 142.6, 135.8 (d, *J* = 3.3 Hz), 134.8, 131.1, 130.1, 128.6, 128.3 (d, *J* = 8.2 Hz), 128.3, 124.0, 123.9 (d, *J* = 35.6 Hz), 118.2, 115.7 (d, *J* = 21.3 Hz), 79.8, 28.0, 24.1; **^{19}F NMR** (377 MHz, DMSO-*d*$_6$) δ = - 115.7; **HRMS-ESI (*m/z*):** [M+Na]$^+$ calcd for $C_{26}H_{26}FN_3O_4$: 486.1800, found: 486.1796.

***tert*-Butyl (2-[4-acetamidobenzamido]-4-[thiophen-2-yl]phenyl)carbamate (5c).** Synthesis according to General Procedure **III**. Flash column chromatography (DCM/MeOH/TEA; 98:2:0.1; *v/v/v*) afforded the desired product as a white solid (79.3 mg, 0.18 mmol, 38 %); **mp.** > 214.7 °C (decomp.); **R$_f$** = 0.29 (DCM/MeOH/TEA; 95:5:0.1; *v/v/v*); **^{1}H NMR** (400 MHz, DMSO-*d*$_6$) δ = 10.25 (s, 1H), 9.83 (s, 1H), 8.72 (s, 1H), 7.97 – 7.91 (m, 2H), 7.82 (d, *J* = 2.1 Hz, 1H),

7.77 – 7.71 (m, 2H), 7.60 (d, *J* = 8.5 Hz, 1H), 7.54 – 7.48 (m, 2H), 7.45 (dd, *J* = 3.6, 1.2 Hz, 1H), 7.13 (dd, *J* = 5.1, 3.6 Hz, 1H), 2.09 (s, 3H), 1.46 (s, 9H); **^{13}C NMR** (101 MHz, DMSO-*d*$_6$) δ = 168.8, 165.0, 153.3, 142.7, 142.6, 131.1, 130.1, 129.6, 128.6, 128.5, 128.2, 125.4, 124.2, 123.4, 122.8, 122.5, 118.1, 79.8, 28.0, 24.2; **HRMS-ESI (*m/z*):** $[M+Na]^+$ calcd for $C_{24}H_{25}N_3O_4S$: 474.1458, found: 474.1439.

***tert*-Butyl (2-[4-acetamidobenzamido]-4-[pyridin-4-yl]phenyl)carbamate (5d).** Synthesis according to General Procedure **III**. Flash column chromatography (DCM/MeOH/TEA; gradient; 98:2:0.1 → 94:6:0.1; *v/v/v*) afforded the desired product as a pale-yellow solid (128 mg, 0.29 mmol, 62 %); **mp.** > 153.9 °C (decomp.); **R$_f$** = 0.34 (DCM/MeOH/TEA; 90:10:0.1; *v/v/v*); **^{1}H NMR** (400 MHz, DMSO-*d*$_6$) δ = 10.25 (s, 1H), 9.87 (s, 1H), 8.84 (s, 1H), 8.65 – 8.59 (m, 2H), 7.97 (d, *J* = 2.2 Hz, 1H), 7.97 – 7.92 (m, 2H), 7.76 – 7.72 (m, 3H), 7.70 – 7.65 (m, 3H), 2.10 (s, 3H), 1.47 (s, 9H); **^{13}C NMR** (101 MHz, DMSO-*d*$_6$) δ = 168.8, 165.1, 153.2, 150.3, 146.1, 142.6, 133.0, 132.4, 129.9, 128.7, 128.2, 124.5, 123.9, 123.8, 120.8, 118.2, 80.0, 28.0, 24.2; **HRMS-ESI (*m/z*):** $[M+H]^+$ calcd for $C_{25}H_{26}N_4O_4$: 447.2027, found: 447.2022.

***tert*-Butyl (2-[4-acetamidobenzamido]-4-[pyridin-3-yl]phenyl)carbamate (5e).** Synthesis according to General Procedure **III**. Flash column chromatography (DCM/MeOH/TEA; gradient; 98:2:0.1 → 94:6:0.1; *v/v/v*) afforded the desired product as a white solid (134 mg, 0.30 mmol, 64 %); **mp.** > 215.4 °C (decomp.); **R$_f$** = 0.42 (DCM/MeOH/TEA; 90:10:0.1; *v/v/v*); **^{1}H NMR** (400 MHz, DMSO-*d*$_6$) δ = 10.25 (s, 1H), 9.86 (s, 1H), 8.88 (dd, *J* = 2.4, 0.8 Hz, 1H), 8.80 (s, 1H), 8.56 (dd, *J* = 4.7, 1.6 Hz, 1H), 8.05 (ddd, *J* = 8.0, 2.4, 1.6 Hz, 1H), 7.98 – 7.92 (m, 2H), 7.90 (d, *J* = 2.2 Hz, 1H), 7.77 – 7.72 (m, 2H), 7.70 (d, *J* = 8.5 Hz, 1H), 7.59 (dd, *J* = 8.5, 2.2 Hz, 1H), 7.48 (ddd, *J* = 8.0, 4.8, 0.9 Hz, 1H), 2.10 (s, 3H), 1.47 (s, 9H); **^{13}C NMR** (101 MHz, DMSO-*d*$_6$) δ = 168.8, 165.0, 153.3, 148.3, 147.3, 142.6, 134.8, 133.7, 132.6, 131.9, 130.1, 128.6, 128.2, 124.4,

124.1, 123.9, 123.9, 118.1, 79.9, 28.0, 24.1; **HRMS-ESI (*m/z*):** $[M+H]^+$ calcd for $C_{25}H_{26}N_4O_4$: 447.2027, found: 447.2034.

***tert*-Butyl (2-[4-acetamidobenzamido]-4-[pyridin-2-yl]phenyl)carbamate (5f).** Starting from **4f** (90 mg, 0.32 mmol, 1.0 equiv.) synthesis according to General Procedure **III**. Flash column chromatography (DCM/MeOH/TEA; gradient; 98:2:0.1 → 96:4:0.1; *v/v/v*) afforded the desired product as a white solid (99.2 mg, 0.22 mmol, 69 %); **mp.** > 220.2 °C (decomp.); $\mathbf{R_f}$ = 0.65 (DCM/MeOH/TEA; 90:10:0.1; *v/v/v*); ^{1}**H NMR** (400 MHz, DMSO-d_6) δ = 10.25 (s, 1H), 9.88 (s, 1H), 8.77 (s, 1H), 8.65 (ddd, J = 4.8, 1.8, 0.9 Hz, 1H), 8.28 (d, J = 2.1 Hz, 1H), 7.98 – 7.94 (m, 2H), 7.95 – 7.91 (m, 2H), 7.87 (ddd, J = 8.0, 7.3, 1.8 Hz, 1H), 7.77 – 7.70 (m, 3H), 7.33 (ddd, J = 7.3, 4.8, 1.2 Hz, 1H), 2.10 (s, 3H), 1.47 (s, 9H); ^{13}**C NMR** (101 MHz, DMSO-d_6) δ = 168.8, 165.0, 155.1, 153.2, 149.5, 142.5, 137.2, 134.2, 132.8, 129.6, 128.6, 128.2, 124.4, 123.6, 123.4, 122.4, 119.8, 118.1, 79.9, 28.0, 24.1; **HRMS-ESI (*m/z*):** $[M+H]^+$ calcd for $C_{25}H_{26}N_4O_4$: 447.2027, found: 447.2035.

***tert*-Butyl (2-[4-acetamidobenzamido]-4-[6-fluoropyridin-3-yl]phenyl)carbamate (5g).** Synthesis according to General Procedure **III**. Flash column chromatography (DCM/MeOH/TEA; 97.5:2.5:0.1; *v/v/v*) afforded the desired product as a white solid (175 mg, 0.38 mmol, 81 %); **mp.** > 228.4 °C (decomp.); $\mathbf{R_f}$ = 0.38 (DCM/MeOH/TEA; 90:10:0.1; *v/v/v*); ^{1}**H NMR** (400 MHz, DMSO-d_6) δ = 10.25 (s, 1H), 9.86 (s, 1H), 8.80 (s, 1H), 8.53 (d, J = 2.5 Hz, 1H), 8.31 – 8.21 (m, 1H), 7.94 (d, J = 8.3 Hz, 2H), 7.88 (d, J = 2.1 Hz, 1H), 7.74 (d, J = 8.0 Hz, 2H), 7.70 (d, J = 8.3 Hz, 1H), 7.57 (dd, J = 8.5, 2.2 Hz, 1H), 7.28 (dd, J = 8.6, 2.9 Hz, 1H), 2.10 (s, 3H), 1.47 (s, 9H); ^{13}**C NMR** (101 MHz, DMSO-d_6) δ = 168.8, 165.0, 162.4 (d, J = 236.5 Hz), 153.3, 145.0 (d, J = 15.1 Hz), 142.6, 140.00 (d, J = 8.0 Hz), 133.4 (d, J = 3.9 Hz), 132.0, 131.4, 130.1, 128.6, 128.2, 124.4, 124.0, 123.9, 118.2, 109.7 (d, J = 37.8 Hz), 79.9, 28.0, 24.2; ^{19}**F NMR** (377 MHz,

DMSO-d_6) δ = - 71.3; **HRMS-ESI (*m/z*):** $[M+Na]^+$ calcd for $C_{25}H_{25}FN_4O_4$: 487.1752, found: 487.1733.

***tert*-Butyl (2-[4-acetamidobenzamido]-4-[2-fluoropyridin-3-yl]phenyl)carbamate (5h).** Synthesis according to General Procedure **III**. Flash column chromatography (DCM/MeOH/TEA; gradient; 99.5:0.5:0.1 → 96.5:3.5:0.1; *v/v/v*) afforded the desired product as a white solid (98.1 mg, 0.21 mmol, 45 %); **mp.** > 223.8 °C (decomp.); $\mathbf{R_f}$ = 0.18 (DCM/MeOH/TEA; 96.5:3.5:0.1; *v/v/v*); **^{1}H NMR** (400 MHz, DMSO-d_6) δ = 10.24 (s, 1H), 9.86 (s, 1H), 8.83 (s, 1H), 8.23 (dt, *J* = 4.9, 1.5 Hz, 1H), 8.11 (ddd, *J* = 10.0, 7.5, 2.0 Hz, 1H), 7.94 (d, *J* = 8.7 Hz, 2H), 7.80 (t, *J* = 1.6 Hz, 1H), 7.72 (dd, *J* = 10.0, 8.6 Hz, 3H), 7.52 – 7.43 (m, 2H), 2.09 (s, 3H), 1.47 (s, 8H); **^{13}C NMR** (101 MHz, DMSO-d_6) δ = 168.8, 165.0, 159.5 (d, *J* = 237.1 Hz), 153.3, 146.3 (d, *J* = 14.9 Hz), 142.6, 141.1 (d, *J* = 4.1 Hz), 132.1, 129.7, 128.8 (d, *J* = 5.0 Hz), 128.6, 128.2, 126.3 (d, *J* = 3.4 Hz), 125.8 (d, *J* = 3.2 Hz), 123.7, 122.7 (d, *J* = 4.1 Hz), 122.2 (d, *J* = 28.1 Hz), 118.1, 79.9, 28.0, 24.1; **^{19}F NMR** (377 MHz, DMSO-d_6) δ = - 71.9; **HRMS-ESI (*m/z*):** $[M+Na]^+$ calcd for $C_{25}H_{25}FN_4O_4$: 487.1752, found: 487.1730.

***tert*-Butyl (2-[4-acetamidobenzamido]-4-[2-fluoropyridin-4-yl]phenyl)carbamate (5i).** Synthesis according to General Procedure **III**. Flash column chromatography (DCM/MeOH/TEA; 99:1:0.1; *v/v/v*) afforded the desired product as a white solid (141 mg, 0.30 mmol, 64 %); **mp.** > 141.4 °C (decomp.); $\mathbf{R_f}$ = 0.22 (DCM/MeOH/TEA; 97.5:2.5:0.1; *v/v/v*); **^{1}H NMR** (300 MHz, DMSO-d_6) δ = 10.26 (s, 1H), 9.88 (s, 1H), 8.87 (s, 1H), 8.28 (d, *J* = 5.3 Hz, 1H), 8.01 (d, *J* = 2.1 Hz, 1H), 7.99 – 7.92 (m, 2H), 7.81 – 7.70 (m, 4H), 7.67 (dt, *J* = 5.4, 1.8 Hz, 1H), 7.49 (p, *J* = 0.7 Hz, 1H), 2.10 (s, 3H), 1.47 (s, 9H); **^{13}C NMR** (75 MHz, DMSO-d_6) δ = 168.8, 165.1, 164.2 (d, *J* = 234.3 Hz), 153.1, 152.3 (d, *J* = 8.5 Hz), 148.1 (d, *J* = 15.9 Hz), 142.6, 133.8, 131.0 (d, *J* = 3.5 Hz), 129.7, 128.7, 128.2, 125.0, 124.3, 123.5, 119.2 (d, *J* = 3.7 Hz), 118.1, 106.0 (d, *J* = 38.7 Hz),

80.0, 28.0, 24.1; **^{19}F NMR** (282 MHz, DMSO-d_6) δ = - 68.8; **HRMS-ESI (*m/z*):** $[M+Na]^+$ calcd for $C_{25}H_{25}FN_4O_4$: 487.1752, found: 487.1737.

General procedure IV for the synthesis of the compounds 6a-i and 10a-c. The respective Boc-protected intermediate **5a-i** or **9a-c** (1.0 equiv.) was dissolved in DCM/TFA (80:20; *v/v*, 20 mg/mL) at 0°C. Subsequently, the reaction mixture was allowed to warm to room temperature and left stirring for 1 – 3 h. Upon completion, indicated by TLC, the solvent was evaporated under reduced pressure. Afterwards, the oily residue was dissolved in EtOAc (50 – 200 mL) and the resulting organic layer was washed with $NaHCO_3$-solution (5%; 3 x 30 – 75 mL), and brine (2 x 30 – 75 mL), dried over Na_2SO_4, filtered and evaporated, yielding the desired products as pale yellow or off-white solids.

4-Acetamido-*N*-(4-amino-[1,1'-biphenyl]-3-yl)benzamide (6a). Starting from **5a** (135 mg, 0.30 mmol, 1.0 equiv.) synthesis according to General Procedure **IV** provided **6a** as white solid (85.3 mg, 0.25 mmol, 83 %); **mp.** > 211.9 °C (decomp.); **^{1}H NMR** (400 MHz, DMSO-d_6) δ = 10.20 (s, 1H), 9.64 (s, 1H), 8.01 – 7.93 (m, 2H), 7.76 – 7.68 (m, 2H), 7.58 – 7.54 (m, 2H), 7.53 (d, *J* = 2.2 Hz, 1H), 7.39 (t, *J* = 7.7 Hz, 2H), 7.33 (dd, *J* = 8.3, 2.2 Hz, 1H), 7.26 – 7.21 (m, 1H), 6.87 (d, *J* = 8.3 Hz, 1H), 5.09 (s, 2H), 2.09 (s, 3H); **^{13}C NMR** (101 MHz, DMSO-d_6) δ = 168.7, 164.9, 142.7, 142.1, 140.2, 128.8, 128.7, 128.7, 128.2, 126.0, 125.5, 124.7, 124.6, 123.8, 118.0, 116.5, 24.1; **HRMS-ESI (*m/z*):** $[M+H]^+$ calcd for $C_{21}H_{19}N_3O_2$: 346.1550, found: 346.1558.

4-Acetamido-*N*-(4-amino-4'-fluoro-[1,1'-biphenyl]-3-yl)benzamide (6b). Starting from **5b** (98.2 mg, 0.21 mmol, 1.0 equiv.) synthesis according to General Procedure **IV** provided **6b** as an off-white solid (75.0 mg, 0.21 mmol, quant.); **mp.** 225.3 – 226.7 °C (decomp.); **^{1}H NMR** (400 MHz, DMSO-d_6) δ = 10.23 (s, 1H), 9.64 (s, 1H), 8.01 – 7.93 (m, 2H), 7.76 – 7.67 (m, 2H), 7.63 – 7.53 (m, 2H), 7.49 (d, *J* = 2.2 Hz, 1H), 7.29 (dd, *J* = 8.3, 2.3 Hz, 1H), 7.25 – 7.17 (m, 2H),

6.86 (d, J = 8.4 Hz, 1H), 5.10 (s, 2H), 2.09 (s, 3H); **^{13}C NMR** (101 MHz, DMSO-d_6) δ = 168.8, 164.9, 161.0 (d, J = 242.5 Hz), 142.7, 142.2, 136.7 (d, J = 3.0 Hz), 128.7 (2C), 127.3 (d, J = 7.9 Hz), 127.2, 124.7, 124.5, 123.8, 118.0, 116.6, 115.5 (d, J = 21.1 Hz), 24.1; **^{19}F NMR** (376 MHz, DMSO-d_6) δ = - 117.5; **HRMS-ESI (*m/z*):** $[M+H]^+$ calcd for $C_{21}H_{18}FN_3O_2$: 364.1456, found: 364.1475.

4-Acetamido-*N*-(2-amino-5-[thiophen-2-yl]phenyl)benzamide (6c). Starting from **5c** (50.1 mg, 0.11 mmol, 1.0 equiv.) synthesis according to General Procedure **IV** provided **6c** as grey solid (37.9 mg, 0.11 mmol, quant.); **mp.** 225.3 °C, > 232.5 °C (decomp.); **^{1}H NMR** (400 MHz, DMSO-d_6) δ = 10.20 (s, 1H), 9.63 (s, 1H), 8.00 – 7.93 (m, 2H), 7.74 – 7.67 (m, 2H), 7.47 (d, J = 2.2 Hz, 1H), 7.35 (dd, J = 5.1, 1.1 Hz, 1H), 7.29 (dd, J = 8.3, 2.2 Hz, 1H), 7.24 (dd, J = 3.6, 1.2 Hz, 1H), 7.05 (dd, J = 5.1, 3.6 Hz, 1H), 6.82 (d, J = 8.3 Hz, 1H), 5.14 (s, 2H), 2.09 (s, 3H); **^{13}C NMR** (101 MHz, DMSO-d_6) δ = 168.7, 164.9, 144.2, 143.0, 142.2, 128.7, 128.6, 128.2, 123.9, 123.8, 123.6, 123.2, 122.3, 121.0, 118.0, 116.4, 24.1; **HRMS-ESI (*m/z*):** $[M+H]^+$ calcd for $C_{19}H_{17}N_3O_2S$: 352.1114, found: 352.1117.

4-Acetamido-*N*-(2-amino-5-[pyridin-4-yl]phenyl)benzamide (6d). Starting from **5d** (94.2 mg, 0.21 mmol, 1.0 equiv.) synthesis according to General Procedure **IV** provided **6d** as yellow solid (46.2 mg, 0.13 mmol, 62 %); **mp.** > 256.3 °C (decomp.); **^{1}H NMR** (400 MHz, DMSO-d_6) δ =10.45 (s, 1H), 9.75 (s, 1H), 8.54 – 8.47 (m, 2H), 8.04 – 7.96 (m, 2H), 7.79 – 7.71 (m, 2H), 7.70 (d, J = 2.2 Hz, 1H), 7.62 – 7.56 (m, 2H), 7.50 (dd, J = 8.4, 2.2 Hz, 1H), 6.90 (d, J = 8.4 Hz, 1H), 5.44 (s, 2H), 2.10 (s, 3H); **^{13}C NMR** (101 MHz, DMSO-d_6) δ = 168.8, 165.0, 149.8, 147.0, 144.9, 142.3, 128.7, 128.5, 125.2, 124.8, 124.0, 123.6, 119.7, 118.0, 116.3, 24.1; **HRMS-ESI (*m/z*):** $[M+H]^+$ calcd for $C_{20}H_{18}N_4O_2$: 347.1503, found: 347.1518.

4-Acetamido-*N*-(2-amino-5-[pyridin-3-yl]phenyl)benzamide (6e). Starting from **5e** (103 mg, 0.23 mmol, 1.0 equiv.) synthesis according to General Procedure **IV** provided **6e** as an off-white solid (70.3 mg, 0.20 mmol, 87 %); **mp.** > 165.6 °C (decomp.); **^{1}H NMR** (300 MHz, DMSO-d_6) δ = 10.20 (s, 1H), 9.65 (s, 1H), 8.79 (s, 1H), 8.44 (d, *J* = 4.7 Hz, 1H), 8.00 – 7.91 (m, 3H), 7.74 – 7.67 (m, 2H), 7.57 (d, *J* = 2.2 Hz, 1H), 7.43 – 7.35 (m, 2H), 6.90 (d, *J* = 8.3 Hz, 1H), 5.20 (s, 2H), 2.09 (s, 3H); **^{13}C NMR** (75 MHz, DMSO-d_6) δ = 168.7, 164.9, 147.0, 146.6, 143.5, 142.2, 135.6, 132.7, 128.7, 128.7, 125.0, 124.8, 124.6, 123.8, 123.8, 118.0, 116.6, 24.1; **HRMS-ESI (*m/z*):** $[M+H]^+$ calcd for $C_{20}H_{18}N_4O_2$: 347.1503, found: 347.1520.

4-Acetamido-*N*-(2-amino-5-[pyridin-2-yl]phenyl)benzamide (6f). Starting from **5f** (49.9 mg, 0.11 mmol, 1.0 equiv.) synthesis according to General Procedure **IV** provided **6f** as cream coloured solid (35.2 mg, 0.10 mmol, 91 %); **mp.** 217.3 – 219.1 °C; **^{1}H NMR** (400 MHz, DMSO-d_6) δ = 10.20 (s, 1H), 9.65 (s, 1H), 8.54 (dt, *J* = 4.6, 1.4 Hz, 1H), 8.01 – 7.94 (m, 3H), 7.80 – 7.67 (m, 5H), 7.19 (ddd, *J* = 6.0, 4.8, 2.7 Hz, 1H), 6.86 (d, *J* = 8.4 Hz, 1H), 5.27 (s, 2H), 2.09 (s, 3H); **^{13}C NMR** (101 MHz, DMSO-d_6) δ = 168.7, 165.0, 156.1, 149.1, 144.5, 142.1, 136.9, 128.7, 126.6, 125.3, 124.8, 123.3, 120.9, 118.4, 118.0, 115.9, 24.1; **HRMS-ESI (*m/z*):** $[M+H]^+$ calcd for $C_{20}H_{18}N_4O_2$: 347.1503, found: 347.1506.

4-Acetamido-*N*-(2-amino-5-[6-fluoropyridin-3-yl]phenyl)benzamide (6g). Starting from **5g** (144 mg, 0.31 mmol, 1.0 equiv.) synthesis according to General Procedure **IV** provided **6g** as an off-white solid (107 mg, 0.29 mmol, 94 %); **mp.** > 300.3 °C (decomp.); **^{1}H NMR** (400 MHz, DMSO-d_6) δ = 10.20 (s, 1H), 9.65 (s, 1H), 8.42 (dt, *J* = 2.0, 0.9 Hz, 1H), 8.14 (td, *J* = 8.3, 2.7 Hz, 1H), 8.00 – 7.93 (m, 2H), 7.74 – 7.67 (m, 2H), 7.55 (d, *J* = 2.2 Hz, 1H), 7.37 (dd, *J* = 8.4, 2.3 Hz, 1H), 7.19 (dd, *J* = 8.6, 2.8 Hz, 1H), 6.89 (d, *J* = 8.3 Hz, 1H), 5.20 (s, 2H), 2.09 (s, 3H); **^{13}C NMR** (101 MHz, DMSO-d_6) δ = 168.7, 165.0, 161.7 (d, *J* = 234.2 Hz), 143.9 (d, *J* = 14.9 Hz), 143.5,

142.2, 138.9 (d, *J* = 7.9 Hz), 134.3 (d, *J* = 4.4 Hz), 128.72, 128.66, 125.0, 124.8, 123.8, 123.5, 118.0, 116.5, 109.4 (d, *J* = 37.6 Hz), 24.1; **^{19}F NMR** (377 MHz, DMSO-*d*$_6$) δ = -73.1; **HRMS-ESI (*m/z*):** [M+H]$^+$ calcd for $C_{20}H_{17}FN_4O_2$: 365.1408, found: 365.1403.

4-Acetamido-*N*-(2-amino-5-[2-fluoropyridin-3-yl]phenyl)benzamide (6h). Starting from **5h** (77.0 mg, 0.17 mmol, 1.0 equiv.) synthesis according to General Procedure **IV** provided **6h** as an off-white solid (58.5 mg, 0.16 mmol, 94 %); **mp.** 216.4 – 218.0 °C; **^{1}H NMR** (400 MHz, DMSO-*d*$_6$) δ = 10.20 (s, 1H), 9.64 (s, 1H), 8.11 (dt, *J* = 4.9, 1.6 Hz, 1H), 8.02 (ddd, *J* = 10.5, 7.5, 1.9 Hz, 1H), 7.98 – 7.94 (m, 2H), 7.74 – 7.67 (m, 2H), 7.48 (t, *J* = 1.8 Hz, 1H), 7.40 (ddd, *J* = 7.5, 4.8, 2.0 Hz, 1H), 7.28 (dt, *J* = 8.4, 2.0 Hz, 1H), 6.89 (d, *J* = 8.4 Hz, 1H), 5.29 (s, 2H), 2.09 (s, 3H); **^{13}C NMR** (101 MHz, DMSO-*d*$_6$) δ = 168.7, 165.0, 159.5 (d, *J* = 236.5 Hz), 144.8 (d, *J* = 15.0 Hz), 143.7, 142.2, 140.1 (d, *J* = 4.7 Hz), 128.7, 128.6, 127.1 (d, *J* = 3.5 Hz), 126.8 (d, *J* = 3.1 Hz), 123.3, 123.0 (d, *J* = 27.8 Hz), 122.6 (d, *J* = 4.1 Hz), 120.6 (d, *J* = 5.3 Hz), 118.0, 116.0, 24.1; **^{19}F NMR** (377 MHz, DMSO-*d*$_6$) δ = - 71.9; **HRMS-ESI (*m/z*):** [M+H]$^+$ calcd for $C_{20}H_{17}FN_4O_2$: 365.1408, found: 365.1410.

4-Acetamido-*N*-(2-amino-5-[2-fluoropyridin-4-yl]phenyl)benzamide (6i). Starting from **5i** (94.3 mg, 0.20 mmol, 1.0 equiv.) synthesis according to General Procedure **IV** provided **6i** as an off-white solid (50.5 mg, 0.14 mmol, 70 %); **mp.** 259.8 – 262.1 °C (decomp.); **^{1}H NMR** (300 MHz, DMSO-*d*$_6$) δ = 10.20 (s, 1H), 9.63 (s, 1H), 8.16 (d, *J* = 5.5 Hz, 1H), 8.02 – 7.92 (m, 2H), 7.77 – 7.66 (m, 3H), 7.60 – 7.53 (m, 2H), 7.35 (d, *J* = 1.5 Hz, 1H), 6.88 (d, *J* = 8.5 Hz, 1H), 5.47 (s, 2H), 2.09 (s, 3H); **^{13}C NMR** (75 MHz, DMSO-*d*$_6$) δ = 168.7, 165.1, 164.4 (d, *J* = 233.2 Hz), 153.0 (d, *J* = 8.7 Hz), 147.7 (d, *J* = 16.4 Hz), 145.6, 142.2, 128.8, 128.6, 125.7, 125.3, 123.4, 122.8 (d, *J* = 3.5 Hz), 118.0, 117.9 (d, *J* = 3.3 Hz), 116.0, 104.2 (d, *J* = 38.6 Hz),

24.1; **^{19}F NMR** (282 MHz, DMSO-d_6) δ = - 69.5; **HRMS-ESI (*m/z*):** $[M+Na]^+$ calcd for $C_{20}H_{17}FN_4O_2$: 387.1228, found: 387.1238.

General procedure V for the synthesis of the compounds 8a-c. The respective methyl benzoate **7a-c** (1.0 equiv.) was dissolved in THF/MeOH (90:10; *v/v*, 15 mg/mL) and a NaOH-solution (50 mg/mL, 2.5 equiv.) was added. The reaction mixture was heated to 40 °C for 2 – 24 h. Upon completion of the reaction, 1 M HCl (3.0 equiv.) was added, and the resulting solution was concentrated *in vacuo* and diluted with H_2O/EtOAc (50:50, *v/v*). Upon phase separation the aqueous layer was extracted with EtOAc (3 x). Subsequently, the combined organic phases were washed with brine (2 x 75 mL), dried over Na_2SO_4, filtered and evaporated under reduced pressure, yielding the desired products **8a-c** as white solids.

4-({*N*-[2-(Benzylamino)-2-oxoethyl]-4-(dimethylamino)benzamido}methyl)benzoic acid (8a). Starting from **7a** (690 mg, 1.50 mmol, 1.0 equiv.) synthesis according to General Procedure **V** provided **8a** as white solid (463 mg, 1.04 mmol, 69 %); **mp.** 172.9 – 174.8 °C (decomp.); **^{1}H NMR** (400 MHz, DMSO-d_6) δ = 12.87 (s, 1H), 8.44 (t, *J* = 5.8 Hz, 1H), 7.94 (d, *J* = 7.7 Hz, 2H), 7.41 (d, *J* = 7.9 Hz, 2H), 7.37 – 7.20 (m, 9H), 6.69 – 6.61 (m, 2H), 4.71 (s, 2H), 4.31 (d, *J* = 5.8 Hz, 2H), 3.92 (s, 2H), 2.92 (s, 6H); **^{13}C NMR** (75 MHz, DMSO-d_6) δ = 171.8, 168.2, 167.2, 151.3, 142.7, 139.2, 129.7, 129.6, 128.6 (2C), 128.3, 127.3, 126.9, 121.9, 111.0, 51.1 (confirmed by ^{1}H-^{13}C-HSQC), 49.0 (confirmed by ^{1}H-^{13}C-HSQC), 42.2, 39.7; **HRMS-ESI (*m/z*):** $[M+H]^+$ calcd for $C_{26}H_{27}N_3O_4$: 446.2074, found: 446.2086.

4-({*N*-[2-(Cyclohexylamino)-2-oxoethyl]-4-(dimethylamino)benzamido}methyl)benzoic acid (8b). Starting from **7b** (300 mg, 0.66 mmol, 1.0 equiv.) synthesis according to General Procedure **V** provided **8b** as white solid (274 mg, 0.63 mmol, 95 %); **mp.** 222.1 – 223.5 °C (decomp.); **^{1}H NMR** (400 MHz, DMSO-d_6) δ = 12.92 (s, 1H), 7.93 (d, *J* = 8.0 Hz, 2H), 7.77 (d,

J = 7.8 Hz, 1H), 7.40 (d, *J* = 8.0 Hz, 2H), 7.33 (d, *J* = 8.2 Hz, 2H), 6.68 (d, *J* = 8.5 Hz, 2H), 4.65 (s, 2H), 3.81 (s, 2H), 3.55 (qd, *J* = 8.8, 7.2, 5.2 Hz, 1H), 2.93 (s, 6H), 1.75 – 1.60 (m, 4H), 1.53 (dt, *J* = 12.7, 3.9 Hz, 1H), 1.32 – 1.14 (m, 2H), 1.11 (tt, *J* = 12.7, 6.3 Hz, 3H); **^{13}C NMR** (101 MHz, DMSO-d_6) δ = 171.7, 167.1, 166.8, 151.2, 142.7, 129.6, 129.6, 128.6, 127.8, 122.0, 111.0, 51.2 (confirmed by ^{1}H-^{13}C-HSQC), 48.8 (confirmed by ^{1}H-^{13}C-HSQC), 47.6, 39.5 (confirmed by ^{1}H-^{13}C-HSQC), 32.3, 25.2, 24.5; **HRMS-ESI (*m/z*):** [M+H]$^+$ calcd for $C_{25}H_{31}N_3O_4$: 438.2387, found: 438.2380.

4-({*N*-[2-(*tert*-Butylamino)-2-oxoethyl]-4-(dimethylamino)benzamido}methyl)benzoic acid (8c). Starting from **7c** (300 mg, 0.71 mmol, 1.0 equiv.) synthesis according to General Procedure **V** provided **8c** as white solid (269 mg, 0.65 mmol, 98 %); **mp.** 228.7 – 230.8 °C (decomp.); **^{1}H NMR** (400 MHz, DMSO-d_6) δ = 7.96 – 7.89 (m, 2H), 7.47 (s, 1H), 7.39 (d, *J* = 7.9 Hz, 2H), 7.36 – 7.29 (m, 2H), 6.71 – 6.64 (m, 2H), 4.64 (s, 2H), 3.78 (s, 2H), 2.93 (s, 6H), 1.23 (s, 9H); **^{13}C NMR** (101 MHz, DMSO-d_6) δ = 171.7, 167.3, 167.2, 151.2, 142.8, 129,7, 129.6, 128.6, 127.6, 122.1, 111.0, 51.8, 50.3, 49.2, 39,4 (confirmed by ^{1}H-^{13}C-HSQC), 28.5; **HRMS-ESI (*m/z*):** [M+H]$^+$ calcd for $C_{23}H_{29}N_3O_4$: 412.2231, found: 412.2223.

General procedure VI for the synthesis of the compounds 9a-c. To a suspension of the respective carboxylic acid **8a-c** (200 mg, 1.2 equiv.) in dry DMF (2 mL), EDC•HCl (3.0 equiv.), HOBt•H_2O (3.0 equiv.) and DIPEA (5.0 equiv.) were added, and the reaction mixture was allowed to stir at room temperature until a clear solution has formed. Subsequently, **4d** (1.0 equiv.), dissolved in dry DCM (1 mL), was added and the reaction mixture was allowed to stir at room temperature for 16 h. Upon completion, the reaction mixture was diluted with EtOAc (150 mL) and the organic layer was washed with H_2O (2 x 50 mL) and brine (2 x 50 mL), dried over Na_2SO_4,

filtered and evaporated. Purification of the crude product by flash column chromatography (DCM/MeOH/TEA; 97:3:0.1; *v/v/v*) afforded the desired products as pale-yellow solids.

***tert*-Butyl {2-[4-({*N*-[2-(benzylamino)-2-oxoethyl]-4-(dimethylamino)benzamido}methyl) benzamido]-4-(pyridin-4-yl)phenyl}carbamate (9a).** Starting from **8a** (200 mg, 0.45 mmol, 1.2 equiv.) and **4d** (108 mg, 0.38 mmol, 1.0 equiv.) synthesis according to General Procedure **VI** provided **9a** as pale-yellow solid (148 mg, 0.21 mmol, 55 %); **mp.** 136.7 – 138.8 °C (decomp.); $\mathbf{R_f}$ = 0.11 (DCM/MeOH/TEA; 97:3:0.1; *v/v/v*); **^{1}H NMR** (400 MHz, DMSO-d_6) δ = 9.96 (s, 1H), 8.85 (s, 1H), 8.66 – 8.60 (m, 2H), 8.47 (t, *J* = 6.0 Hz, 1H), 8.01 (s, 1H), 7.99 (d, *J* = 2.0 Hz, 2H), 7.78 (d, *J* = 8.6 Hz, 1H), 7.71 – 7.66 (m, 3H), 7.49 (d, *J* = 8.0 Hz, 2H), 7.38 – 7.31 (m, 4H), 7.29 – 7.23 (m, 3H), 6.70 – 6.63 (m, 2H), 4.74 (s, 2H), 4.33 (d, *J* = 5.9 Hz, 2H), 3.94 (s, 2H), 2.94 (s, 6H), 1.47 (s, 9H); **^{13}C NMR** (101 MHz, DMSO-d_6) δ = 171.8, 168.2, 165.4, 153.2, 151.2, 150.2, 146.1, 141.7, 139.2, 133.1, 133.0, 132.3, 129.7, 128.6, 128.4, 128.3, 128.0, 127.3, 126.8, 124.6, 124.1, 123.7, 120.8, 110.9, 80.0, 42.1, 28.0; **HRMS-ESI (*m/z*):** $[M+H]^+$ calcd for $C_{42}H_{44}N_6O_5$: 713.3446, found: 713.3453.

***tert*-Butyl {2-[4-({*N*-[2-(cyclohexylamino)-2-oxoethyl]-4-(dimethylamino)benzamido} methyl)benzamido]-4-(pyridin-4-yl)phenyl}carbamate (9b).** Starting from **8b** (200 mg, 0.46 mmol, 1.2 equiv.) and **4d** (108 mg, 0.38 mmol, 1.0 equiv.) synthesis according to General Procedure **VI** provided **9b** as pale-yellow solid (162 mg, 0.23 mmol, 61 %); **mp.** > 150.9 °C (decomp.); $\mathbf{R_f}$ = 0.12 (DCM/MeOH/TEA; 97:3:0.1; *v/v/v*); **^{1}H NMR** (300 MHz, DMSO-d_6) δ = 9.96 (s, 1H), 8.85 (s, 1H), 8.67 – 8.59 (m, 2H), 7.99 (dd, *J* = 5.3, 3.0 Hz, 3H), 7.83 – 7.74 (m, 2H), 7.72 – 7.63 (m, 3H), 7.47 (d, *J* = 8.0 Hz, 2H), 7.35 (d, *J* = 8.3 Hz, 2H), 6.69 (d, *J* = 8.6 Hz, 2H), 4.69 (s, 2H), 3.84 (s, 2H), 3.62 – 3.53 (m, 1H), 2.93 (s, 6H), 1.77 – 1.37 (m, 4H), 1.47 (s, 9H), 1.36 – 1.02 (m, 6H); **^{13}C NMR** (75 MHz, DMSO-d_6) δ = 171.7, 166.9, 165.4, 153.2, 151.2,

150.2, 146.2, 141.8, 133.1, 133.0, 132.3, 129.7, 128.7, 128.6, 128.0, 128.0, 124.5, 124.1, 123.8, 120.8, 111.0, 80.0, 47.6, 39.7, 32.3, 28.0, 25.2, 24.5; **HRMS-ESI (*m/z*):** $[M+H]^+$ calcd for $C_{41}H_{48}N_6O_5$: 705.3759, found: 705.3762.

***tert*-Butyl {2-[4-({*N*-[2-(*tert*-butylamino)-2-oxoethyl]-4-(dimethylamino)benzamido} methyl)benzamido]-4-(pyridin-4-yl)phenyl}carbamate (9c).** Starting from **8c** (200 mg, 0.49 mmol, 1.2 equiv.) and **4d** (117 mg, 0.41 mmol, 1.0 equiv.) synthesis according to General Procedure **VI** provided **9c** as pale-yellow solid (162 mg, 0.24 mmol, 59 %); **mp.** > 148.8 °C (decomp.); $\mathbf{R_f}$ = 0.11 (DCM/MeOH/TEA; 97:3:0.1; *v/v/v*); ^{1}**H NMR** (400 MHz, DMSO-d_6) δ = 9.96 (s, 1H), 8.85 (s, 1H), 8.66 – 8.60 (m, 2H), 7.99 (dd, *J* = 5.3, 3.0 Hz, 3H), 7.77 (d, *J* = 8.6 Hz, 1H), 7.72 – 7.65 (m, 3H), 7.52 – 7.41 (m, 3H), 7.34 (d, *J* = 8.3 Hz, 2H), 6.69 (d, *J* = 8.8 Hz, 2H), 4.68 (s, 2H), 3.81 (s, 2H), 2.94 (s, 6H), 1.47 (s, 9H), 1.26 (s, 9H); ^{13}**C NMR** (101 MHz, DMSO-d_6) δ = 171.7, 167.3, 165.4, 153.2, 151.2, 150.2, 146.2, 141.9, 133.1, 133.0, 132.3, 129.8, 128.7, 128.6, 128.0, 124.5, 124.1, 123.8, 120.8, 111.0, 80.0, 51.9, 50.3, 49.0, 28.5, 28.0; **HRMS-ESI (*m/z*):** $[M+H]^+$ calcd for $C_{39}H_{46}N_6O_5$: 679.3602, found: 679.3604.

***N*-(4-{[2-Amino-5-(pyridin-4-yl)phenyl]carbamoyl}benzyl)-*N*-[2-(benzylamino)-2-oxoethyl]-4-(dimethylamino)benzamide (10a).** Starting from **9a** (97.2 mg, 0.14 mmol, 1.0 equiv.) synthesis according to General Procedure **IV** provided **10a** as yellow solid (81.5 mg, 0.13 mmol, 93 %); **mp.** 114.1, > 131.8 °C (decomp.); ^{1}**H NMR** (400 MHz, DMSO-d_6 + TFA (1.0 equiv.)) δ = 9.75 (s, 1H), 8.71 (d, *J* = 6.6 Hz, 2H), 8.49 (t, *J* = 6.1 Hz, 1H), 8.22 (d, *J* = 6.4 Hz, 2H), 8.03 (d, *J* = 8.1 Hz, 2H), 7.98 (d, *J* = 2.4 Hz, 1H), 7.83 (dd, *J* = 8.7, 2.3 Hz, 1H), 7.46 (d, *J* = 8.0 Hz, 2H), 7.39 – 7.22 (m, 7H), 6.93 (d, *J* = 8.7 Hz, 1H), 6.67 (d, *J* = 8.7 Hz, 2H), 4.74 (s, 2H), 4.33 (d, *J* = 5.9 Hz, 2H), 3.93 (s, 2H), 2.94 (s, 6H); ^{13}**C NMR** (101 MHz, DMSO-d_6 + TFA (1.0 equiv.)) δ = 171.7, 168.2, 165.7, 155.1, 151.2, 148.3, 141.3, 141.2, 139.3, 133.3, 128.8, 128.6,

128.3, 128.2, 127.6, 127.3, 126.8, 123.2, 120.6, 119.8, 115.8, 111.0, 51.4, 49.5, 42.1; **HRMS-ESI (*m/z*):** $[M+H]^+$ calcd for $C_{37}H_{36}N_6O_3$: 613.2922, found: 613.2926.

***N*-(4-{[2-Amino-5-(pyridin-4-yl)phenyl]carbamoyl}benzyl)-*N*-[2-(cyclohexylamino)-2-oxoethyl]-4-(dimethylamino)benzamide (10b).** Starting from **9b** (119 mg, 0.17 mmol, 1.0 equiv.) synthesis according to General Procedure **IV** provided **10b** as yellow solid (96.9 mg, 0.16 mmol, 94 %); **mp.** 120.3, > 137.4 °C (decomp.); **^{1}H NMR** (400 MHz, DMSO-d_6 + TFA (1.0 equiv.)) δ = 9.77 (s, 1H), 8.71 (d, *J* = 6.5 Hz, 2H), 8.20 (d, *J* = 6.4 Hz, 2H), 8.03 (d, *J* = 8.0 Hz, 2H), 7.97 (d, *J* = 2.3 Hz, 1H), 7.81 (dd, *J* = 8.7, 2.5 Hz, 2H), 7.44 (d, *J* = 8.0 Hz, 2H), 7.35 (d, *J* = 8.4 Hz, 2H), 6.94 (d, *J* = 8.6 Hz, 1H), 6.69 (d, *J* = 8.5 Hz, 2H), 4.69 (s, 2H), 3.84 (s, 2H), 3.64 – 3.53 (m, 1H), 2.94 (s, 6H), 1.70 (ddt, *J* = 24.0, 12.4, 3.5 Hz, 2H), 1.55 (dt, *J* = 13.1, 3.9 Hz, 1H), 1.36 – 1.02 (m, 6H); **^{13}C NMR** (101 MHz, DMSO-d_6 + TFA (1.0 equiv.)) δ = 171.7, 166.9, 165.6, 154.9, 151.2, 148.2, 141.5, 141.3, 133.3, 128.8, 128.6, 128.2, 127.5, 127.2, 123.2, 122.2, 120.6, 119.9, 115.9, 111.0, 51.4, 49.1, 47.6, 32.4, 25.2, 24.5; **HRMS-ESI (*m/z*):** $[M+Na]^+$ calcd for $C_{36}H_{40}N_6O_3$: 627.3054, found: 627.3078.

***N*-(4-{[2-Amino-5-(pyridin-4-yl)phenyl]carbamoyl}benzyl)-*N*-[2-(*tert*-butylamino)-2-oxoethyl]-4-(dimethylamino)benzamide (10c).** Starting from **9c** (116 mg, 0.17 mmol, 1.0 equiv.) synthesis according to General Procedure **IV** provided **10c** as yellow solid (98.9 mg, 0.17 mmol, quant.); **mp.** 108.7, > 136.6 °C (decomp.); **^{1}H NMR** (400 MHz, DMSO-d_6 + TFA (1.0 equiv.)) δ = 9.77 (s, 1H), 8.72 (d, *J* = 6.6 Hz, 2H), 8.22 (d, *J* = 6.6 Hz, 2H), 8.03 (d, *J* = 7.9 Hz, 2H), 7.99 (d, *J* = 2.3 Hz, 1H), 7.82 (dd, *J* = 8.7, 2.4 Hz, 1H), 7.51 (s, 1H), 7.44 (d, *J* = 8.0 Hz, 2H), 7.35 (d, *J* = 8.4 Hz, 2H), 6.94 (d, *J* = 8.7 Hz, 1H), 6.70 (d, *J* = 8.5 Hz, 2H), 4.68 (s, 2H), 3.81 (s, 2H), 2.94 (s, 6H), 1.26 (s, 9H); **^{13}C NMR** (101 MHz, DMSO-d_6 + TFA (1.0 equiv.)) δ = 171.7, 167.4, 165.7, 155.2, 151.1, 148.3, 141.4, 141.2, 133.3, 128.7, 128.6, 128.2, 127.6, 127.5, 127.2, 123.2, 122.5,

120.7, 119.8, 115.9, 111.1, 51.8, 50.3, 49.3, 39.8, 28.5; **HRMS-ESI (*m/z*):** $[M+H]^+$ calcd for $C_{34}H_{38}N_6O_3$: 579.3078, found: 579.3080.

HDAC Assays

Preincubation assay for HDAC1-3 and HDAC6. *In vitro* inhibitory activity against HDAC1-3 and HDAC6 was determined using a modified protocol based on our previously published assay.[12] For compounds and controls, 3fold serial dilutions of the respective DMSO-stock solution in assay buffer (50 mM Tris−HCl, pH 8.0, 137 mM NaCl, 2.7 mM KCl, 1.0 mM $MgCl_2$•6 H_2O, 0.1 mg/mL BSA), were prepared and 5.0 µL of this serial dilutions were transferred into OptiPlate-96 black micro-plates (PerkinElmer). Then, 25 µL assay buffer and 10 µL enzyme solution (human recombinant HDAC1 (BPS Bioscience, Catalog# 50051); HDAC2 (BPS Bioscience, Catalog# 50052); HDAC3/NcoR2 (BPS Bioscience, Catalog# 50003); HDAC6 (BPS Bioscience, Catalog# 50006) were added. Enzyme and inhibitor were preincubated at 25 °C for 60 minutes (HDAC1-3) or 15 minutes (HDAC6). Afterwards, the fluorogenic substrate ZMAL (Z-Lys(Ac)-AMC[13]; 10 µL; 75 µM in assay buffer) was added. The total assay volume (50 µL, max. 1% DMSO) was incubated at 37 °C for 90 min. Subsequently, 50 µL trypsin solution (0.4 mg/mL trypsin in buffer: 50 mM Tris−HCl, pH 8.0, 100 mM NaCl) was added, followed by additional 30 minutes of incubation at 37 °C. Fluorescence (excitation: 355 nm, emission: 460 nm) was measured using an Ascent Fluoroskan microplate reader (Thermo Scientific). Compounds were tested at least twice in duplicates; the 50% inhibitory concentration (IC_{50}) was determined by plotting normalized dose response curves using nonlinear regression (Prism 9 for MacOS).

IC_{50}-shift experiments at HDAC1-3. For IC_{50}-shift experiments we used the preincubation assay for HDAC1-3 as stated above and varied the preincubation period as follows: HDAC1-3

enzymes and the respective inhibitor dilutions were preincubated at 20 °C for 5 to 120 minutes. Afterwards, the assay protocol was continued as stated above. Compounds were tested in triplicates and the assay was rerun if necessary. The respective IC_{50} values and P95 confidence intervals were determined using Prism 9 for MacOS.

Determination of binding kinetics via the *Progression Method*.[14] HDAC1 and HDAC3 deacetylase activities were evaluated at varying inhibitor concentrations. Appropriate inhibitor concentrations were chosen based on the previously determined IC_{50} values. To ensure substrate excess during the experiment, the substrate concentration was set to five times K_m. K_m was determined using a series of substrate concentrations. The respective steady-state velocities were plotted against the corresponding substrate concentrations [S] and fitted to the MICHALIS-MENTEN equation (K_m HDAC1 = 10.03 µM; K_m HDAC3 = 8.49 µM). For the progression curves, the respective enzyme was incubated with the fluorogenic substrate and inhibitor in assay buffer (90 µL; 50 mM Tris−HCl, pH 8.0, 137 mM NaCl, 2.7 mM KCl, 1.0 mM MgCl2•6 H2O, 0.1 mg/mL BSA) and 10 µL trypsin solution (50 ng/µL; buffer: 50 mM Tris−HCl, pH 8.0, 100 mM NaCl). The total assay volume (100 µL) contained the following final concentrations: HDAC1 (Lot #: 200109; 220 pg/µL), HDAC3 (Lot #: 190327; 260 pg/µL), Z-Lys(Ac)-AMC[13] (50.0 µM for HDAC1, 42.5 µM for HDAC3) and trypsin (5 ng/µL[15] for HDAC1&3). *In situ* AMC release was monitored continuously by fluorescence readings (excitation: 355 nm, emission: 460 nm; Ascent Fluoroskan microplate reader, Thermo Scientific) recorded every 0.5 min for 60 or 90 minutes at 37°C. The relationship between AMC concentration and relative fluorescence units (RFU) was determined and the measured RFU were transformed into the respective AMC concentration in µM. Within the steady-state timeframe (HDAC1: 25 – 90 min; HDAC3: 15 – 60 min) the data of each progression curve were fit to either obtain the apparent first-order rate

constant k_{obs} (Eq. 1) or the linear conversion rates v_i and v_0, respectively (Eq. 2). For fast-on / fast-off inhibitors the ratio v_i/v_0 was plotted against the corresponding inhibitor concentrations. The resulting curves were fit to Eq. 3 to determine the K_i values. The apparent first-order rate constants k_{obs} derived from the respective progression curves of slow-binding inhibitors were replotted against the corresponding inhibitor concentrations [I] and the curves were either fit to Eq. 4 or Eq. 6. Compounds were tested in triplicates and the assay was rerun if necessary. Data were fitted to the relevant equations using Prism 9 for MacOS.

100-fold *Jump-Dilution* experiments. In assay buffer (50 mM Tris−HCl, pH 8.0, 137 mM NaCl, 2.7 mM KCl, 1.0 mM $MgCl_2$•6 H_2O, 0.1 mg/mL BSA) HDAC1 (22 ng/µL) or HDAC3 (26 ng/µL) were incubated with an inhibitor-concentrate (at least 10fold IC_{50}) or blank (DMSO 1%) for 1 hour at room temperature. Afterwards, this "incubation-mix" was diluted 100fold either in the presence of the respective inhibitors at their original concentrations or solely with assay buffer. Substrate and trypsin (10 µL; 50 ng/µL; buffer: 50 mM Tris−HCl, pH 8.0, 100 mM NaCl) were added to all samples. The total assay volume (100 µL) contained the following final concentrations: HDAC1 (Lot #: 200109; 220 pg/µL), HDAC3 (Lot #: 190327; 260 pg/µL), Z-Lys(Ac)-AMC[13] (HDAC1: 50.0 µM; HDAC3: 42.5 µM) and trypsin (5 ng/µL[15] for HDAC1&3). The time-dependent *in situ* AMC release was monitored continuously by fluorescence readings (excitation: 355 nm, emission: 460 nm; TECAN Infinite® 200 PRO M Nano$^+$ microplate reader) recorded every 0.5 min for 60 or 90 minutes at 37°C.

Physicochemical Properties

$LogD_{7.4}$. $LogD_{7.4}$ was determined using the OECD slow-stirring method.[16,17] In short, a glass vial (20x40 mm) was equipped with a magnetic stirring bar (10 mm; PTFE covered, cylindrical),

a sample of the respective test substance (0.1 – 1.0 mg), 2.0 mL 1-octanol-saturated PBS buffer (pH = 7.4; 137 mM NaCl, 2.7 mM KCl, 10 mM Na_2HPO_4, 1.8 mM KH_2PO_4) and 2.0 mL PBS-saturated 1-octanol. The vial was firmly sealed, placed on a magnetic stirrer and the biphasic system was left stirring (1000 rpm) at 25 °C for 48 h. 4 h into the experiment all vials were checked for residual suspended solid test substance. Vials with residual solid particles were excluded from the experiment. After 48 h the stirring was stopped, and the phases were separated. The concentration of the test substance in the respective 1-octanol and PBS buffer phase was quantified by comparison with a six-point calibration curve using HPLC/UV detection. As detection wavelength a local maximum between 230 nm and 350 nm was selected individually for each test substance. $LogD_{7.4}$ values were determined by performing three slow-stirring experiments (three independent experimental units). HPLC quantification of each 1-octanol or PBS buffer phase was carried out in duplicates.

Solubility. The solubility of test substances in PBS buffer (pH = 7.4; 137 mM NaCl, 2.7 mM KCl, 10 mM Na_2HPO_4, 1.8 mM KH_2PO_4) was determined using the OECD flask method.[18] In short, in a glass vial (20x40 mm) was equipped with a magnetic stirring bar (10 mm; PTFE covered, cylindrical), a pulverized sample of the respective test substance (1.5 – 5.0 mg) and 3.0 mL PBS buffer was added. The vial was firmly sealed, placed on a magnetic stirrer and the system was left stirring (1000 rpm) at 25 °C for 72 h. All vials were checked periodically for residual suspended solid test substance. Vials without residual solid particles were excluded from the experiment. After 72 h the stirring was stopped, and the remaining particles were filtered off using a syringe filter (PTFE membrane, pore size: 0.45 μm). The concentration of the test substance in the PBS buffer phase was quantified by comparison with a six-point calibration curve using HPLC/UV detection. The detection wavelength was selected individually for each test

substance (local maximum between 230 nm and 350 nm). Solubility was determined by performing two independent experiments. HPLC quantification of each PBS buffer phase was carried out in duplicate.

Docking

Docking studies of 6d (LSH-A33)**, 10c** (LSH-A54) **and VK-1 to human HDAC1 with RosettaLigand.** The crystal structure of human HDAC1 (PDB: 5ICN)[2] was obtained from the Protein Data Bank (PDB, www.rcsb.org). Chain A, corresponding to the metastasis-associated protein MTA1, chain C, corresponding to a histone 4 based peptide inhibitor, and inositol-6-phosphate were deleted. All heteroatom records were removed, except for the metal ions (one zinc atom and two potassium atoms). The structure was optimized to the closest local energy minimum using RosettaRelax with coordinate constraints on the backbone and metal ion restraints.[19] Ligand input files for **6d** (LSH-A33), **10c** (LSH-A54) and **VK-1** were created with ChemDraw. An initial 3D conformer with hydrogen atoms was constructed in Chem3D and energetically minimized using the MM2 force field, followed by the production of an ensemble of 1000 low-energy conformers with BCL:ConformerGenerator.[20] One conformer was placed in the binding pocket of HDAC1. A constraint file was constructed using the known distance of the zinc binding of *ortho*-aminoanilides.[1] Ligand docking was performed with RosettaLigand for an initial 5000 models. All models with a negative interface score were subsequently submitted to another round of focused refinement using RosettaLigand. For **VK-1** and **6d** (LSH-A33) the best scoring models were identified by their predicted binding energy.[21–23] The model for **10c** (LSH-A54) was chosen by the highest binding mode conformity to the predicted **VK-1** and **6d** (LSH-A33) binding modes.

Rosetta version 3.12 was used. The following commands were executed throughout the modeling process:

- Relax input starting structures:

```
rosetta/source/bin/relax.linuxgccrelease -s 5ICN_prepared.pdb rosetta/source/database/
-constrain_relax_to_start_coords -in:auto_setup_metals -nstruct 25 -out:prefix relaxed_
-ignore_unrecognized_res -overwrite -ignore_waters False
```

- Options for RosettaLigand:

```
-in:file:extra_res_fa ligand.params
-packing
	-ex1
	-ex2
	-no_optH false
	-flip_HNQ true
	-ignore_ligand_chi true
-parser
	-protocol dock.xml

-mistakes
	-restore_pre_talaris_2013_behavior true

-constraints:cst_file constraint_5ICN_aminoanilid.cst

-out:path:all ../output/
```

- RosettaScripts protocol for executing RosettaLigand:

```
<ROSETTASCRIPTS>

		<SCOREFXNS>
			<ScoreFunction name="ligand_soft_rep" weights="ligand_soft_rep">
			</ScoreFunction>
			<ScoreFunction name="hard_rep" weights="ligand">
			</ScoreFunction>
		</SCOREFXNS>

		<LIGAND_AREAS>
			<LigandArea name="inhibitor_dock_sc" chain="X" cutoff="6.0" add_nbr_radius="true"
all_atom_mode="false"/>
			<LigandArea name="inhibitor_final_sc" chain="X" cutoff="6.0" add_nbr_radius="true"
all_atom_mode="false"/>
			<LigandArea name="inhibitor_final_bb" chain="X" cutoff="7.0" add_nbr_radius="false"
all_atom_mode="true" Calpha_restraints="0.3"/>
		</LIGAND_AREAS>

		<INTERFACE_BUILDERS>
			<InterfaceBuilder name="side_chain_for_docking" ligand_areas="inhibitor_dock_sc"/>
			<InterfaceBuilder name="side_chain_for_final" ligand_areas="inhibitor_final_sc"/>
```

```
			<InterfaceBuilder name="backbone" ligand_areas="inhibitor_final_bb"
extension_window="3"/>
		</INTERFACE_BUILDERS>

		<MOVEMAP_BUILDERS>
			<MoveMapBuilder name="docking" sc_interface="side_chain_for_docking"
minimize_water="false"/>
			<MoveMapBuilder name="final" sc_interface="side_chain_for_final"
bb_interface="backbone" minimize_water="false"/>
		</MOVEMAP_BUILDERS>

		<SCORINGGRIDS ligand_chain="X" width="15">
			<ClassicGrid grid_name="classic" weight="1.0"/>
		</SCORINGGRIDS>

		<MOVERS>
			<StartFrom name="startFrom" chain="X">
				<Coordinates x="22.45" y="-18.57" z="1.00"/>
			</StartFrom>
			<ConstraintSetMover name="constraint" add_constraints="true"
cst_file="./constraint_5ICN_aminoanilid.cst"/>
			<Transform name="transform" chain="X" box_size="7.0" move_distance="0.2"
angle="20" cycles="500" repeats="1" temperature="5"/>
			<HighResDocker name="high_res_docker" cycles="6" repack_every_Nth="3"
scorefxn="ligand_soft_rep" movemap_builder="docking"/>
			<FinalMinimizer name="final" scorefxn="hard_rep" movemap_builder="final"/>
			<ClearConstraintsMover name="clearconstraints"/>
			<InterfaceScoreCalculator name="add_scores" chains="X" scorefxn="hard_rep"/>
		</MOVERS>

		<PROTOCOLS>
			<Add mover_name="startFrom"/>
			<Add mover_name="constraint"/>
			<Add mover_name="transform"/>
			<Add mover_name="high_res_docker"/>
			<Add mover_name="final"/>
			<Add mover_name="clearconstraints"/>
			<Add mover_name="add_scores"/>
		</PROTOCOLS>

</ROSETTASCRIPTS>
```

- Executing RosettaLigand:

```
cat ./listof_pdbs_5ICN | parallel -j 10 bash run_dock_parallel.sh
```

- run_dock_parallel.sh file:

```
PDB=$1

rosetta/source/bin/rosetta_scripts.linuxgccrelease -s ${PDB} -database rosetta/database/
-ignore_waters False -in:auto_setup_metals @option.txt -nstruct 200
```

Biological Evaluation

Cell culture. For **western blot** and **migration assay**, MCF-7 human breast cancer cells were cultivated in DMEM (high glucose) medium (Sigma Aldrich, St. Louis, MO, USA) and supplemented with 10% FCS, 1% L-glutamine, 100 U/mL penicillin and 100 µg/mL streptomycin (all reagents were acquired from Thermo Fisher Scientific Inc.; Waltham, MA, USA). Cells were incubated at 37 °C in a humidified atmosphere containing 5% (*v/v*) CO_2. For subcultivation, cells were detached at a confluency of about 90% with EDTA solution (0.2 g/L EDTA × 4 Na, Sigma Aldrich) for 5 min at 37 °C. Test for absence of mycoplasms were performed routinely using MycoSPY® PCR detection kit (Biontex, Munich, Germany). Cell identity was evaluated using STR profile analysis. For the **WST-8 cell viability assay** and the **clonogenic survival assay**, MCF-7, BT-474, and T47D cells were obtained by ATTC (Manassas, VA, USA). All cells were cultivated at in a humidified incubator (37°C, 5% CO_2). Cells were grown in RPMI-1640 medium ("normal culture medium"; Thermo-Fisher, Waltham, MA, USA) supplemented with 20 % (*v/v*) heat-inactivated fetal bovine serum (FBS), 1 mM sodium pyruvate, and 2 mM L-glutamine. Cells were monitored for mycoplasma contamination using the Venor GeM PCR kit (Minerva Biolabs, Berlin, Germany).

WST-8 cell viability assay. For the evaluation of HDACi effects on tumor cell viability, breast cancer cell lines (MCF-7, BT-474, or T47D, respectively) were seeded in a 96-well plate (Greiner 96-well, F-Bottom) in 100 µl "normal culture medium" in a density of 1000 cells per well, sparing the external wells, which were filled with culture medium without cells. After 24 h cells were treated with the respective inhibitor (concentration range: 0.001 – 16 µM) for 120 h. Cellular viability was determined using WST-8 (water soluble tetrazolium) reagent, according to manufacturer's protocol (PromoCell, Colorimetric Cell Viability Kit I). In brief, 10 µL of WST-8

reagent was added to the cells, incubated for 60 min and absorbance was measured at 450 nm against a reference wavelength of 620 nm. For each condition three independent experiments were performed.

Clonogenic survival assay. In addition to the WST-8 assay, effects of HDACi on cell survival were monitored using the clonogenic assay. In brief, 5×10^5 cells growing in 25 cm^2 cell culture flasks were treated with the respective agent, i.e., vehicle (DMSO), HDACi (3.0 μM) or left untreated for 48 h. Afterwards, cells were trypsinized and counted using a hemocytometer. 750 cells per condition were re-seeded into a 6-well plate and incubated in normal culture medium (without any further treatment) for 5 days. Thereafter, the medium was aspirated. The colonies were gently washed with PBS, and then stained by use of 0.5 % (w/v) crystal violet in methanol. The colonies were incubated for 30 min with the staining solution, then gently washed with deionized H_2O and dried at room temperature. Colonies were counted manually under blinded conditions. For each treatment three independent experiments were performed. Plating efficiency: [number of colonies*100]/[number of seeded cells].

Western blot. Tumor cells were centrifuged (450 g, 4 min, 22°C) and lysed with cell extraction buffer (Thermo Fisher), supplemented with 0.1 mM PMSF, Halt™ Protease Inhibitor (Thermo Fisher) and Natriumorthovanadat (Thermo Fisher). Precast gels with a polymerization degree of 4 - 15% were used (Mini-PROTEAN® TGX™ Stain-Free™; Bio-Rad Laboratories GmbH, Munich, Germany). Proteins were transferred to Trans-Blot Turbo®-PVDF membrane (Bio-Rad). The membrane was blocked with skimmed milk powder in Tris-buffered saline-Tween 20 (with 0.2% Tween 20) for 60 min, followed by three washing cycles of 10 min using Tris-buffered saline-Tween 20. Afterwards, membranes were incubated with primary antibodies for αTubulin, ac.-αTubulin, Histon H3 and ac.-Histon H3 (Cell Signal, rabbit) for a total of 60 min at room

temperature and then incubated at 4°C overnight. Membranes were rinsed again three times before applying the secondary anti-rabbit IgG HRP-conjugated mAbs (Santa Cruz Biotechnology, Texas, USA) for 90 min. Primary antibodies were diluted 1:500 and the secondary antibody was diluted 1:20.000. After rinsing of the secondary antibody, membranes were detected using Clarity™ECL Western Blotting Substrate (Bio-Rad). For quantitative determination, the StainFree™ technique was employed (Bio-Rad), as well as normalization, against the housekeeping protein GAPDH, which allows the imaging of whole lysates in SDS-PAGE before blotting and normalization against the total protein. Pixel density analysis was performed with the IMAGE LAB software (Bio-Rad).

Migration assay. 3D printed *(size: 0.5 mm gap)* culture-inserts (custom made) were used for pre-cast wound area before 5×10^5 MCF-7 cells were seeded on uncoated 24-well plates (Starlab GmbH, Hamburg, Germany), in total. After preincubation with DMSO (1%) or HDACi (10µM) for 24 h medium was removed, wells were washed once with PBS and fresh FCS-free medium with same inhibitor added. Wound healing was observed for 97 h with 10-fold magnification using a Axiovert 200 microscope (Carl-Zeiss, Jena, Germany). Migration speed was quantified as reduced scratch wound over time.

PAINS Analysis. All compounds were reviewed for pan-assay interference (PAINS) using http://zinc15.docking.org/patterns/home/. No compound was flagged as PAINS.

Determination of Lipophilicity and Solubility (Example)

General information concerning the HPLC measurement:

2	Vorinostat $logD_{7.4}$ / solubility		
**MN Nucleodur 100-5 C18 ec**			
Sample Name:	**Vorinostat 0.xx mg/ml**	*Injection Volume:*	**10,0 – 100,0**
Vial Number:	**GA1**	*Channel:*	**UV_VIS_3**
Sample Type:	**unknown**	*Wavelength:*	**240.0**
Control Program:	**Linda_LogP_1,5ml_5-95_19min**	*Bandwidth:*	**2**
Quantif. Method:	**Auswertung**	*Dilution Factor:*	**1,0000**
Recording Time:	**07.03.19**	*Sample Weight:*	**1,0000**
Run Time (min):	**19,00**	*Sample Amount:*	**1,0000**

1) Calibration of standard curve of **vorinostat**

0.18 mg/mL (injection volume: 10µL)

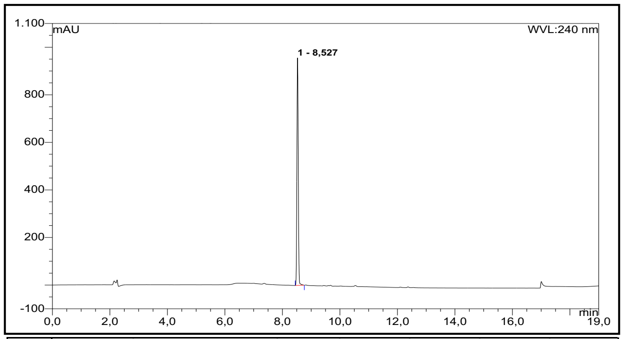

No.	Ret.Time min	Peak Name	Height mAU	Area mAU*min	Rel.Area %	Amount	Type
1	8,53	n.a.	956,666	53,290	100,00	n.a.	BMB*
Total:			956,666	53,290	100,00	0,000	

0.15 mg/mL (injection volume: 10µL)

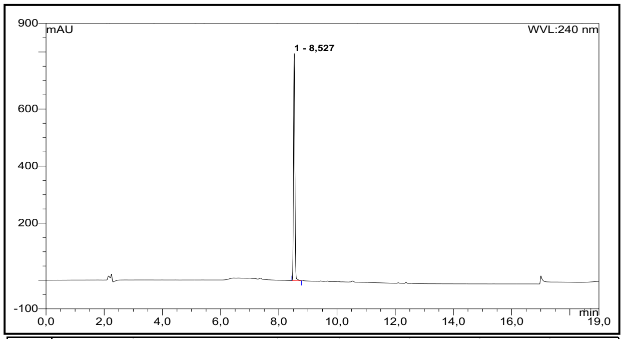

No.	Ret.Time min	Peak Name	Height mAU	Area mAU*min	Rel.Area %	Amount	Type
1	8,53	n.a.	796,036	44,436	100,00	n.a.	BMB*
Total:			796,036	44,436	100,00	0,000	

0.10 mg/mL (injection volume: 10µL)

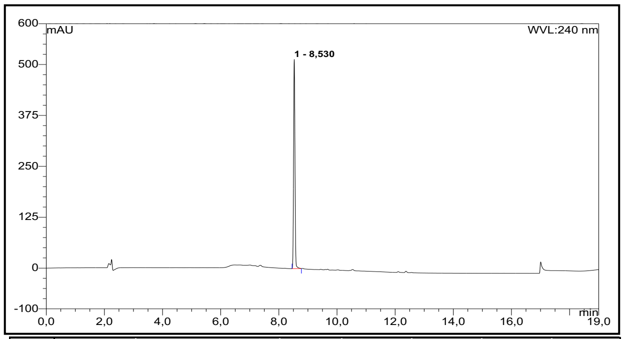

No.	Ret.Time min	Peak Name	Height mAU	Area mAU*min	Rel.Area %	Amount	Type
1	8,53	n.a.	513,629	28,795	100,00	n.a.	BMB*
Total:			513,629	28,795	100,00	0,000	

0.05 mg/mL (injection volume: 10µL)

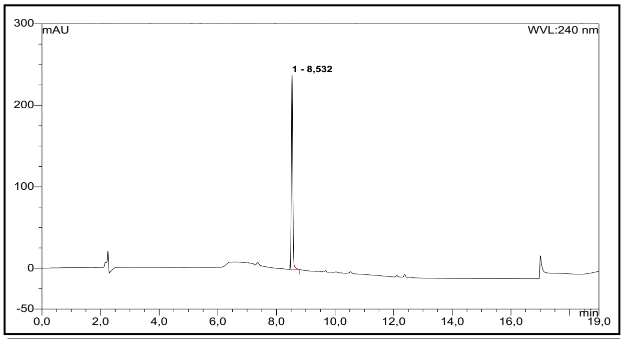

No.	Ret.Time min	Peak Name	Height mAU	Area mAU*min	Rel.Area %	Amount	Type
1	8,53	n.a.	238,893	13,687	100,00	n.a.	BMB*
Total:			238,893	13,687	100,00	0,000	

0.02 mg/mL (injection volume: 10µL)

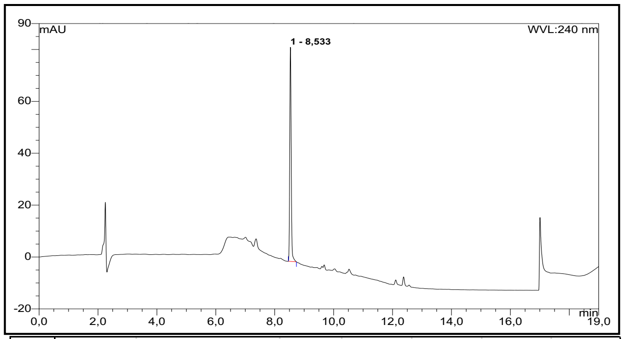

No.	Ret.Time min	Peak Name	Height mAU	Area mAU*min	Rel.Area %	Amount	Type
1	8,53	n.a.	82,550	4,803	100,00	n.a.	BMB*
Total:			82,550	4,803	100,00	0,000	

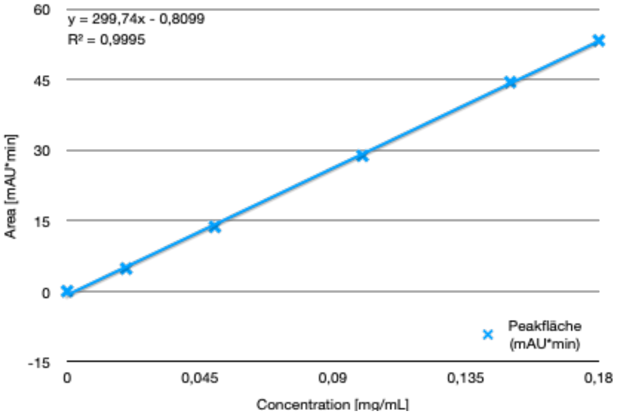

2) Determination of solubility of **vorinostat**

Solubility Experiment I (1)

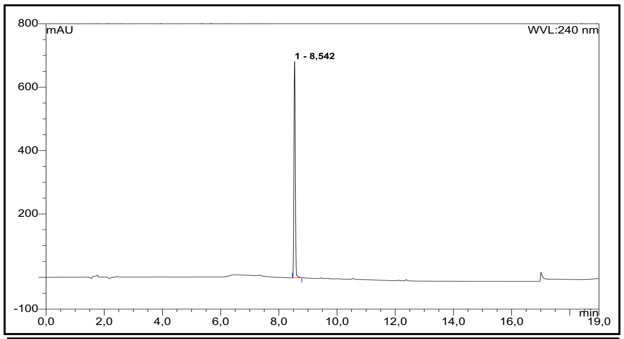

No.	Ret.Time min	Peak Name	Height mAU	Area mAU*min	Rel.Area %	Amount	Type
1	8,54	n.a.	682,247	37,299	100,00	n.a.	BMB*
Total:			682,247	37,299	100,00	0,000	

Solubility Experiment I (2)

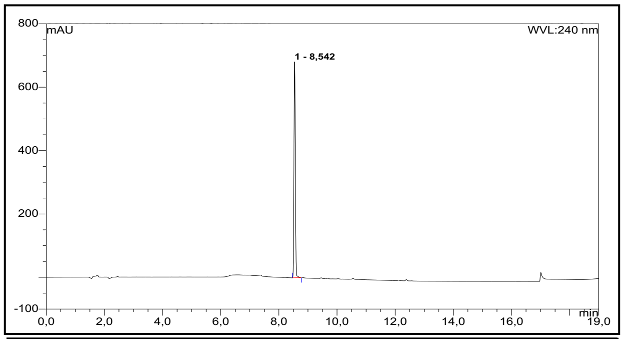

No.	Ret.Time min	Peak Name	Height mAU	Area mAU*min	Rel.Area %	Amount	Type
1	8,54	n.a.	681,020	37,301	100,00	n.a.	BMB*
Total:			681,020	37,301	100,00	0,000	

Solubility Experiment II (1)

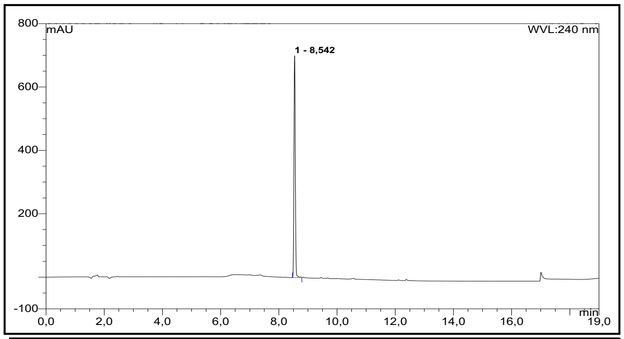

No.	Ret.Time min	Peak Name	Height mAU	Area mAU*min	Rel.Area %	Amount	Type
1	8,54	n.a.	699,536	38,410	100,00	n.a.	BMB*
Total:			699,536	38,410	100,00	0,000	

Solubility Experiment II (2)

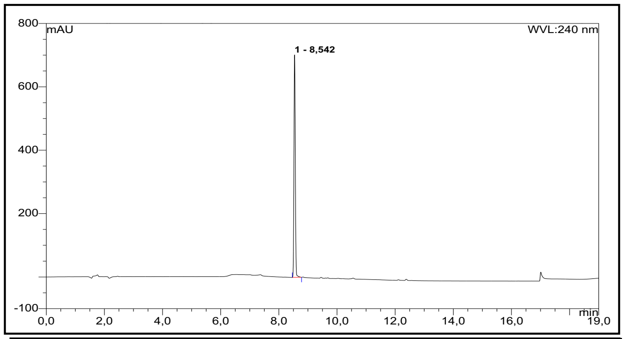

No.	Ret.Time min	Peak Name	Height mAU	Area mAU*min	Rel.Area %	Amount	Type
1	8,54	n.a.	701,908	38,416	100,00	n.a.	BMB*
Total:			701,908	38,416	100,00	0,000	

2) Determination of **vorinostat** $logD_{7.4}$

Aqueous Phase I (1)

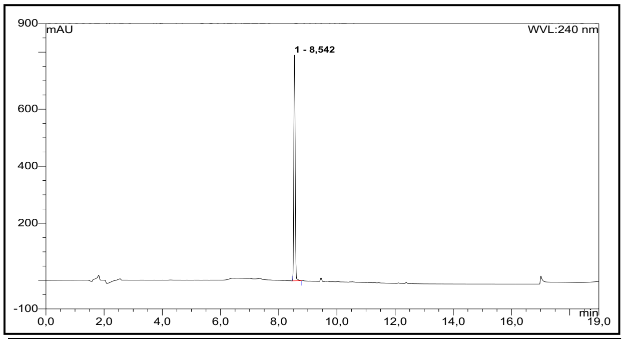

No.	Ret.Time min	Peak Name	Height mAU	Area mAU*min	Rel.Area %	Amount	Type
1	8,54	n.a.	791,021	43,316	100,00	n.a.	BMB*
Total:			791,021	43,316	100,00	0,000	

Aqueous Phase I (2)

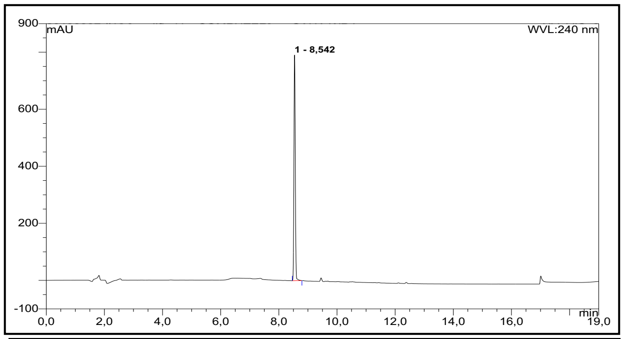

No.	Ret.Time min	Peak Name	Height mAU	Area mAU*min	Rel.Area %	Amount	Type
1	8,54	n.a.	791,202	43,315	100,00	n.a.	BMB*
Total:			791,202	43,315	100,00	0,000	

Aqueous Phase II (1)

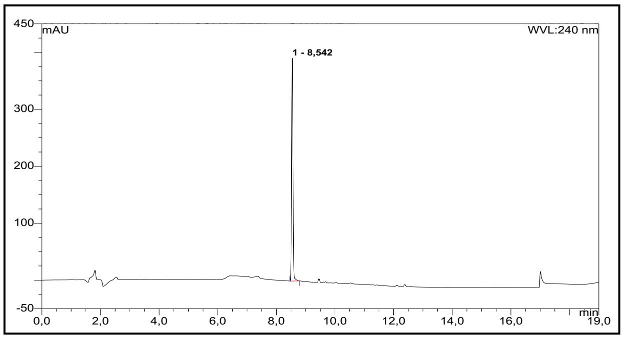

No.	Ret.Time min	Peak Name	Height mAU	Area mAU*min	Rel.Area %	Amount	Type
1	8,54	n.a.	391,227	21,685	100,00	n.a.	BMB*
Total:			391,227	21,685	100,00	0,000	

Aqueous Phase II (2)

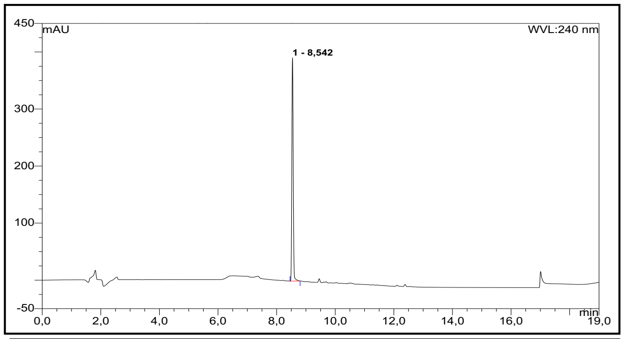

No.	Ret.Time min	Peak Name	Height mAU	Area mAU*min	Rel.Area %	Amount	Type
1	8,54	n.a.	391,206	21,702	100,00	n.a.	BMB*
Total:			391,206	21,702	100,00	0,000	

Aqueous Phase III (1)

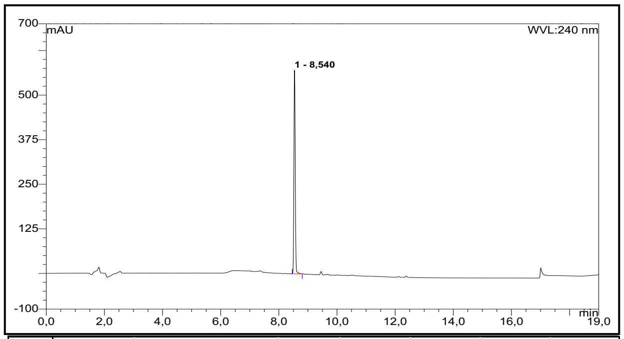

No.	Ret.Time min	Peak Name	Height mAU	Area mAU*min	Rel.Area %	Amount	Type
1	8,54	n.a.	570,888	31,241	100,00	n.a.	BMB*
Total:			570,888	31,241	100,00	0,000	

Aqueous Phase III (2)

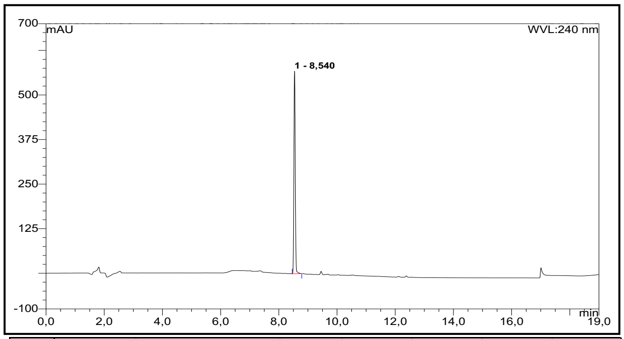

No.	Ret.Time min	Peak Name	Height mAU	Area mAU*min	Rel.Area %	Amount	Type
1	8,54	n.a.	568,182	31,236	100,00	n.a.	BMB*
Total:			568,182	31,236	100,00	0,000	

Octanol Phase I (1)

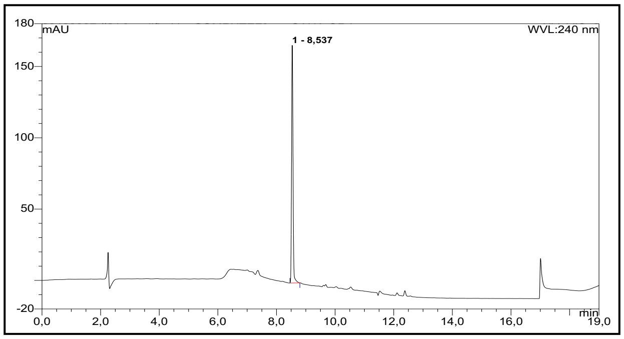

No.	Ret.Time min	Peak Name	Height mAU	Area mAU*min	Rel.Area %	Amount	Type
1	8,54	n.a.	166,602	9,615	100,00	n.a.	BMB*
Total:			166,602	9,615	100,00	0,000	

Octanol Phase I (2)

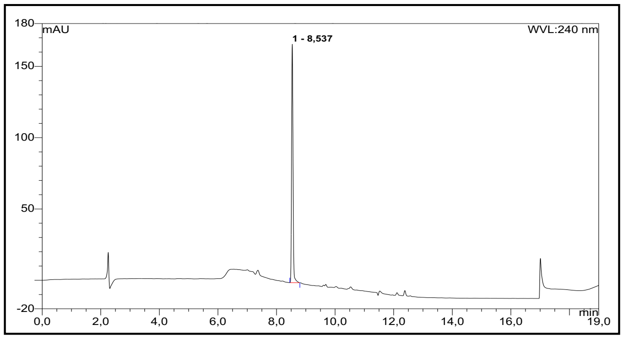

No.	Ret.Time min	Peak Name	Height mAU	Area mAU*min	Rel.Area %	Amount	Type
1	8,54	n.a.	167,240	9,654	100,00	n.a.	BMB*
Total:			167,240	9,654	100,00	0,000	

Octanol Phase II (1)

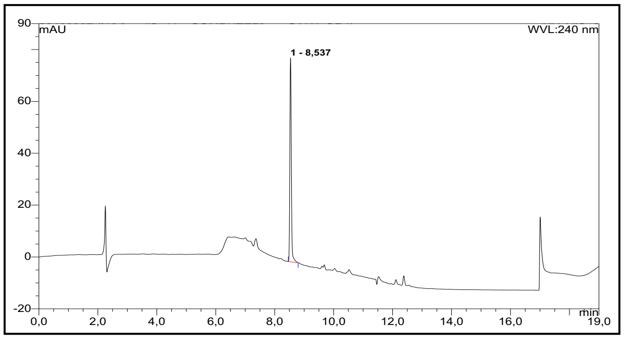

No.	Ret.Time min	Peak Name	Height mAU	Area mAU*min	Rel.Area %	Amount	Type
1	8,54	n.a.	78,620	4,652	100,00	n.a.	BMB*
Total:			78,620	4,652	100,00	0,000	

Octanol Phase II (2)

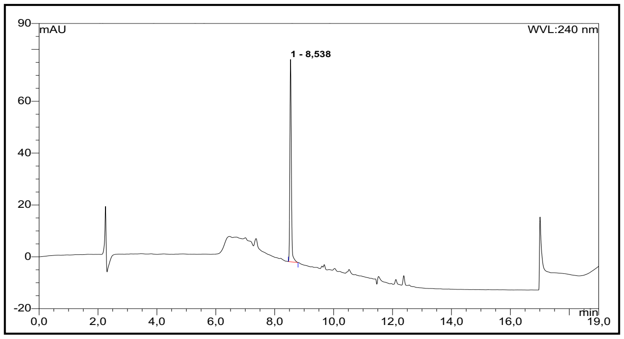

No.	Ret.Time min	Peak Name	Height mAU	Area mAU*min	Rel.Area %	Amount	Type
1	8,54	n.a.	78,046	4,628	100,00	n.a.	BMB*
Total:			78,046	4,628	100,00	0,000	

Octanol Phase III (1)

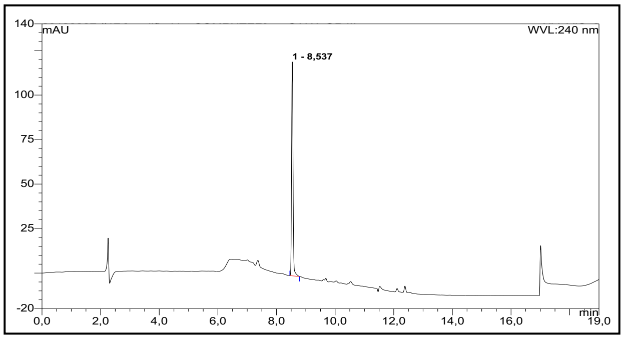

No.	Ret.Time min	Peak Name	Height mAU	Area mAU*min	Rel.Area %	Amount	Type
1	8,54	n.a.	120,198	6,975	100,00	n.a.	BMB*
Total:			120,198	6,975	100,00	0,000	

Octanol Phase III (2)

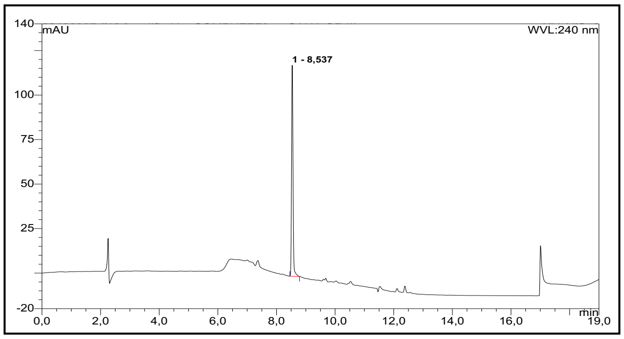

No.	Ret.Time min	Peak Name	Height mAU	Area mAU*min	Rel.Area %	Amount	Type
1	8,54	n.a.	118,564	6,933	100,00	n.a.	BMB*
Total:			118,564	6,933	100,00	0,000	

3) Calculation of solubility & $logD_{7.4}$ of **vorinostat**.

Phase O = octanol; W = aqueous	Area [mAU*min]	Dilution Factor (1 = no dilution)	Injection Volume [µL]	Vorinostat [mg/mL]	Vorinostat Mean of Duplicate [mg/mL]
WP I	43.3156	1	50	0.031604	0.031604
WP I	43.3153	1	50	0.031604	
WP II	21.6846	1	50	0.017171	0.017177
WP II	21.7022	1	50	0.017183	
WP III	31.2413	1	50	0.023548	0.023546
WP III	31.2357	1	50	0.023544	
OP III	6.9755	10	10	0.235420	0.234708
OP III	6.9328	10	10	0.233996	
OP II	4.6516	10	10	0.157890	0.157494
OP II	4.6279	10	10	0.157099	
OP I	9.6153	10	10	0.323490	0.324131
OP I	9.6537	10	10	0.324771	
Solubility I	37.2993	2	10	0.251580	0.251584
Solubility I	37.3005	2	10	0.251588	
Solubility II	38.4096	2	10	0.258988	0.259009
Solubility II	38.4160	2	10	0.259031	
	**LogD(7.4) (I) =**	**1.010978**		**Solubility (I) =**	**0.251584**
	**LogD(7.4) (II) =**	**0.962323**		**Solubility (II) =**	**0.259009**
	**LogD(7.4) (III) =**	**0.998616**		**Solubility =**	**0.25530**
	**LogD(7.4) =**	**0.99**		**SD**	**0.00371**

NMR SPECTRA OF SYNTHESIZED COMPOUNDS BY NUMBER

Synthesis of fragment compounds 6a-i

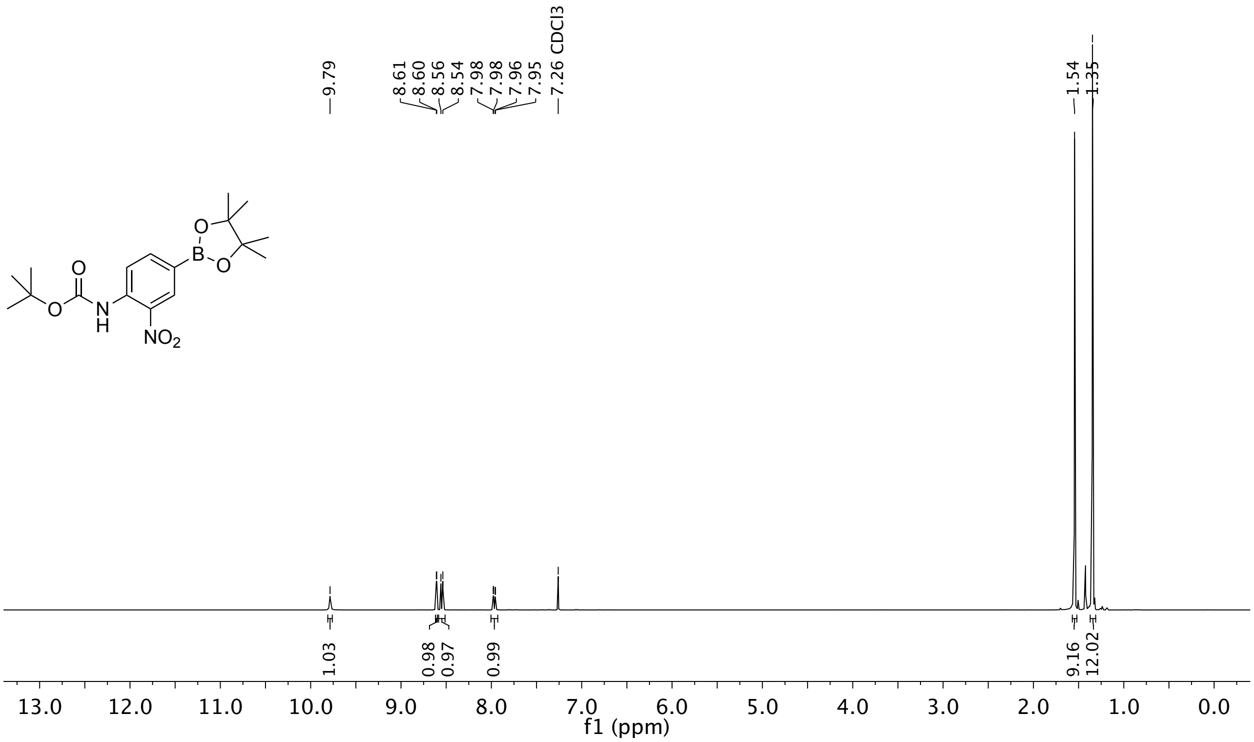

[1]**H NMR** (400 MHz, Chloroform-*d*) of **2**.

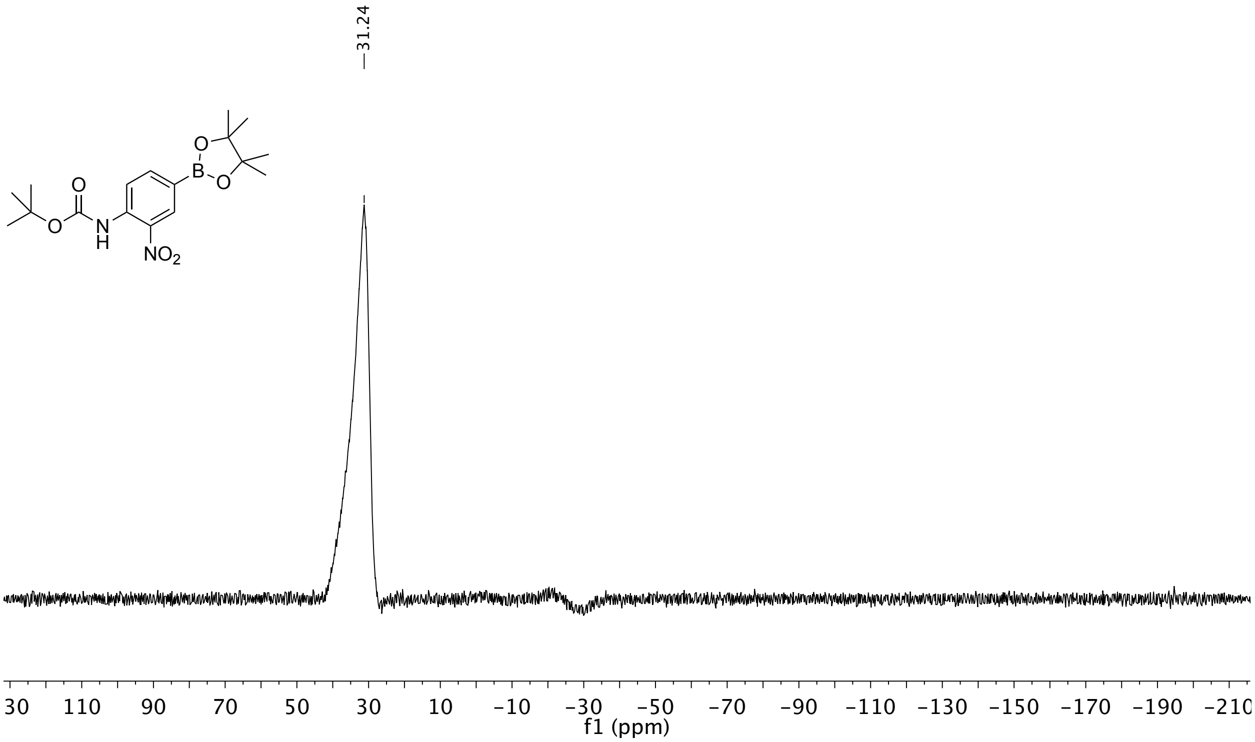

^{11}**B NMR** (128 MHz, Chloroform-*d*) of **2**.

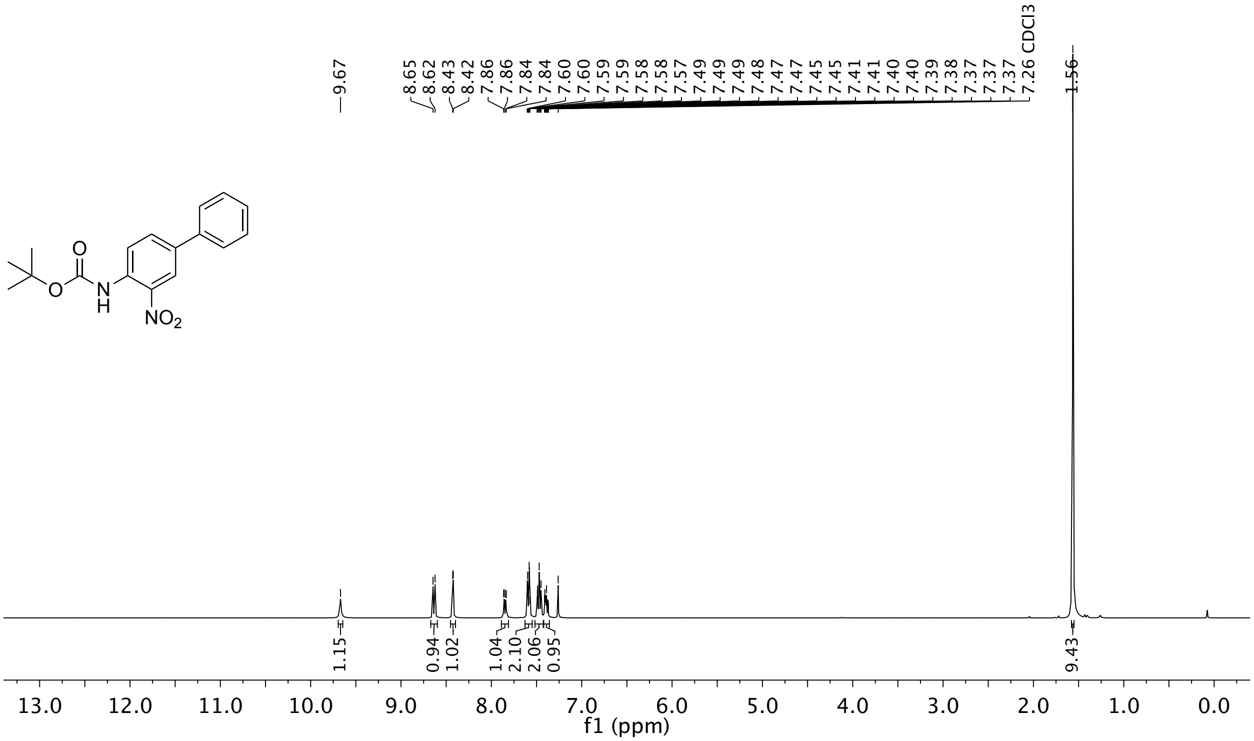

^{1}**H NMR** (400 MHz, Chloroform-*d*) of **3a**.

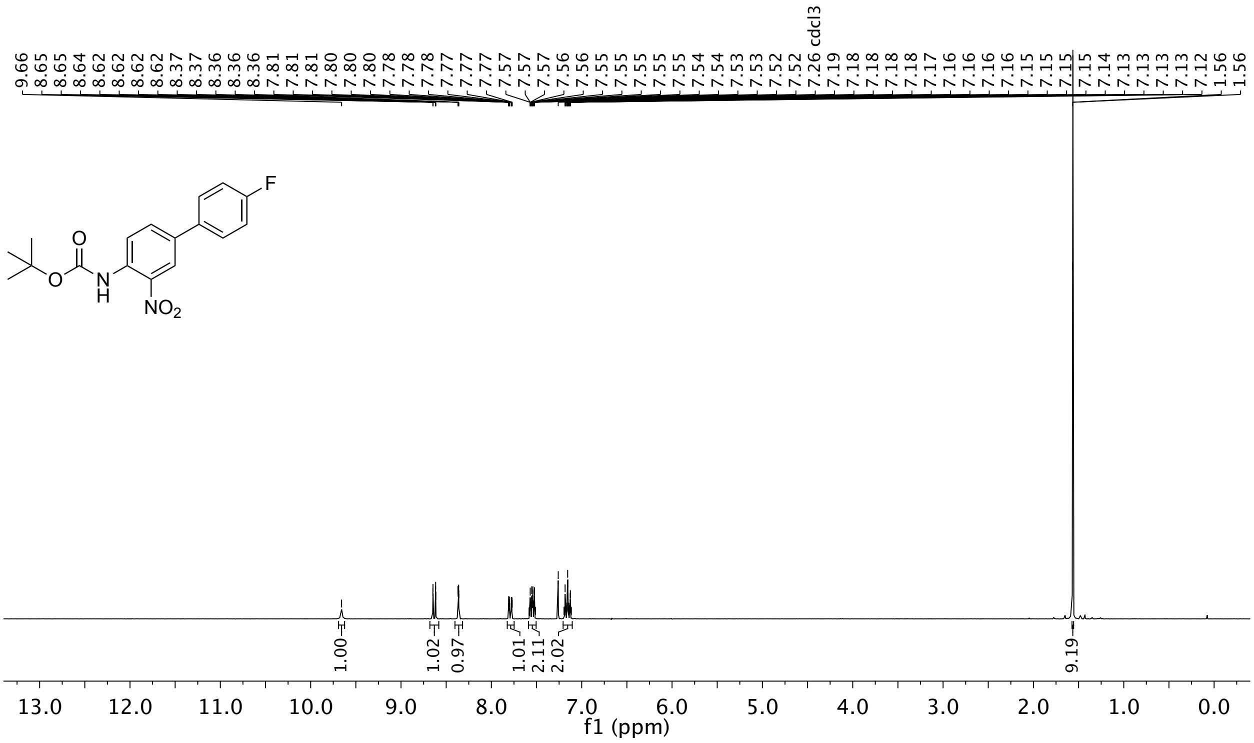

^{1}H NMR (300 MHz, Chloroform-*d*) of **3b**.

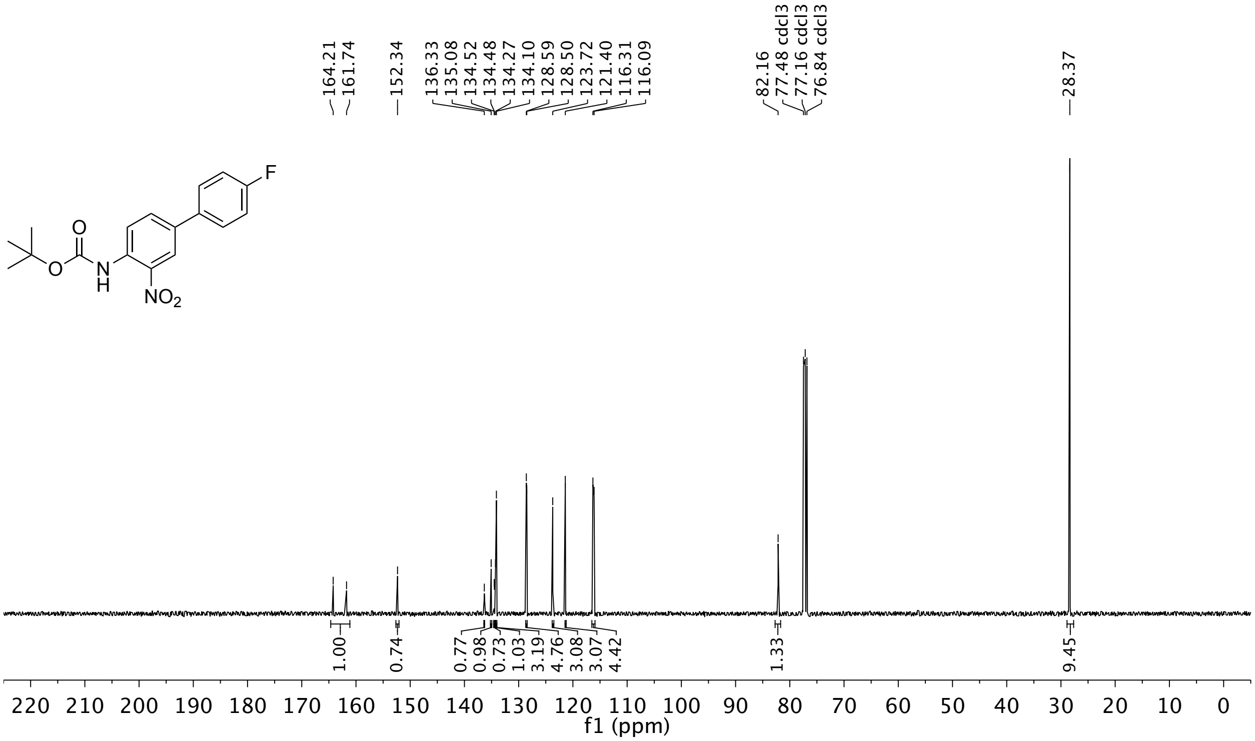

^{13}C NMR (101 MHz, Chloroform-*d*) of **3b**.

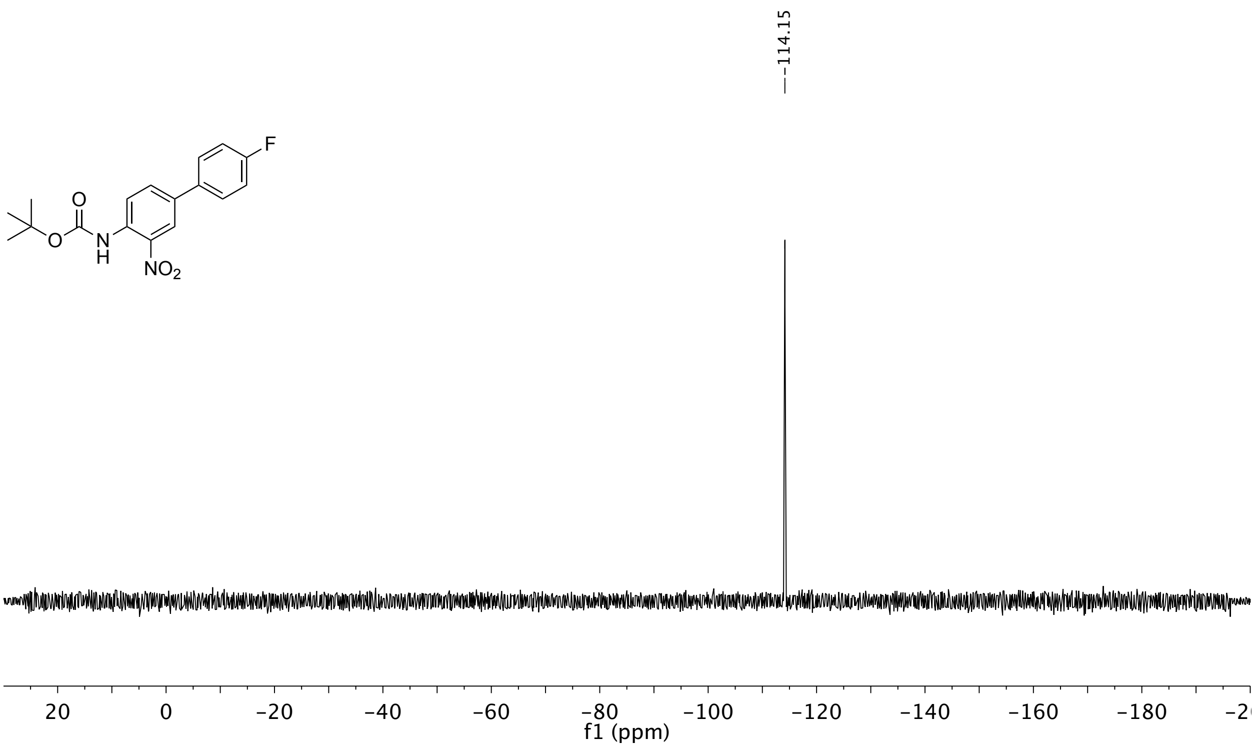

^{19}F NMR (282 MHz, Chloroform-*d*) of **3b**.

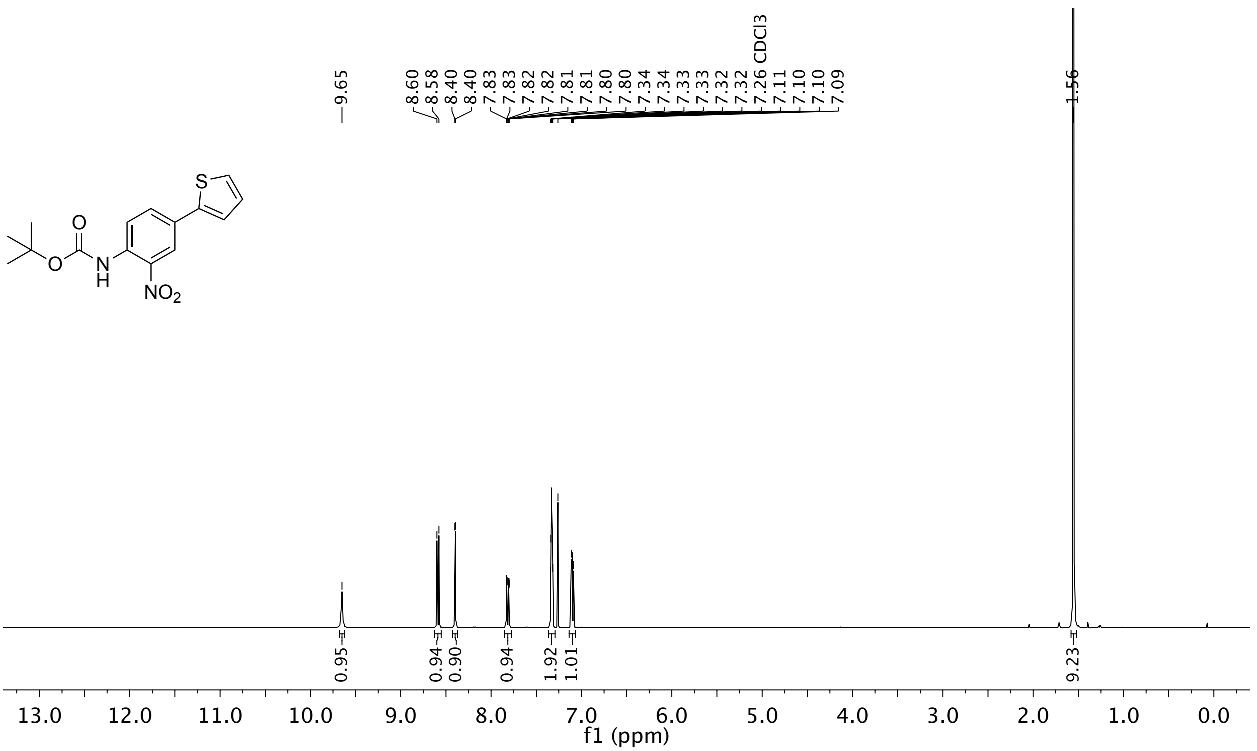

^{1}H NMR (400 MHz, Chloroform-*d*) of **3c**.

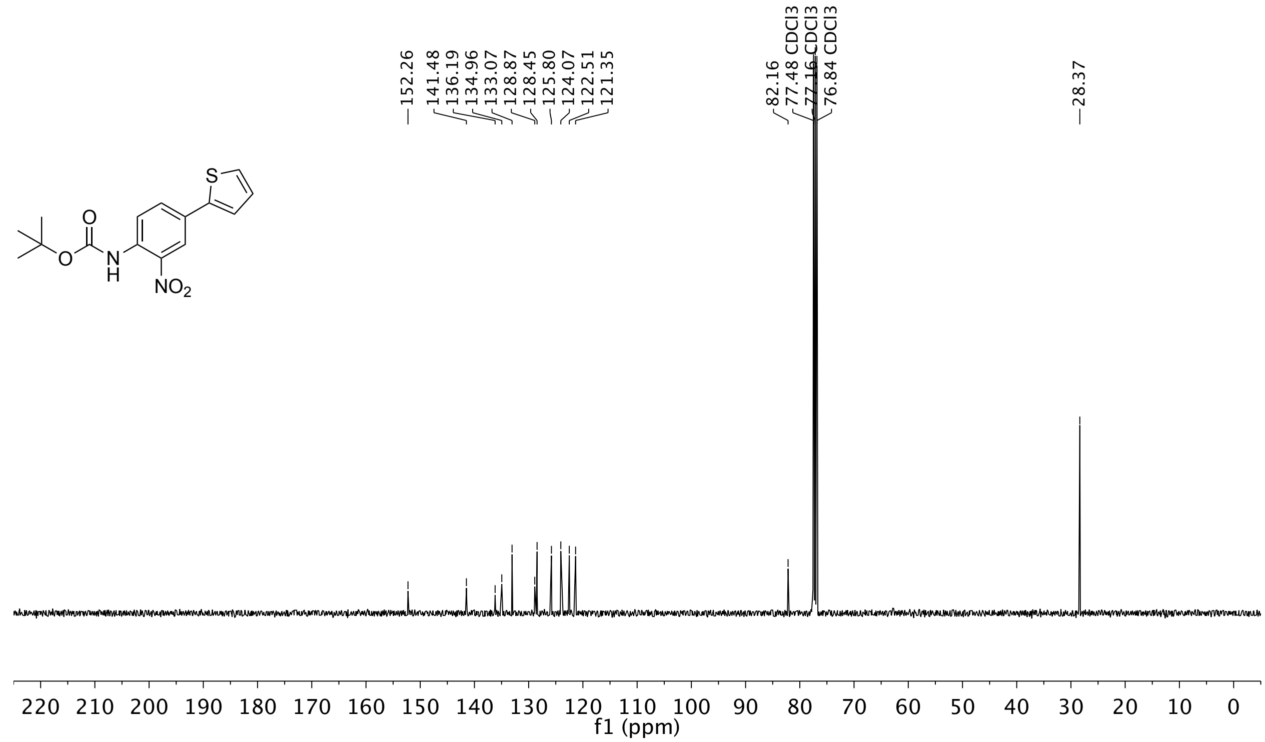

13C NMR (101 MHz, Chloroform-*d*) of **3c**.

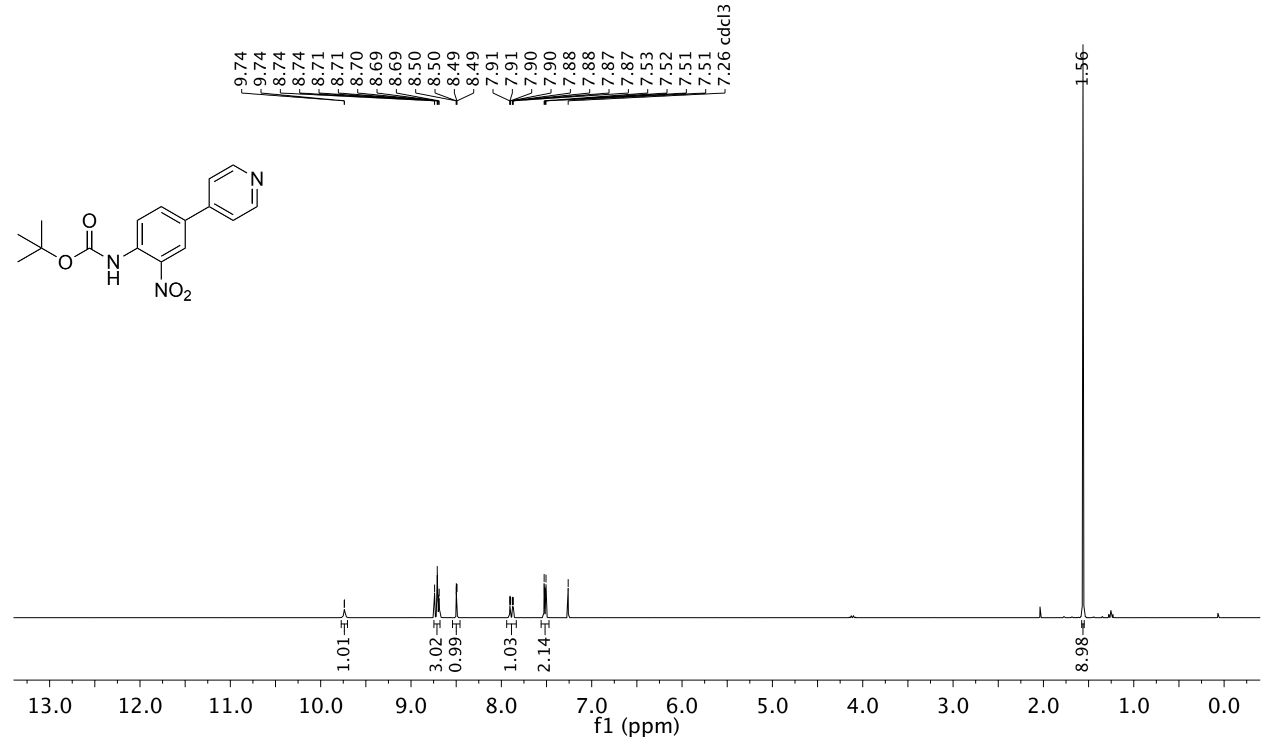

1H NMR (300 MHz, Chloroform-*d*) of **3d**.

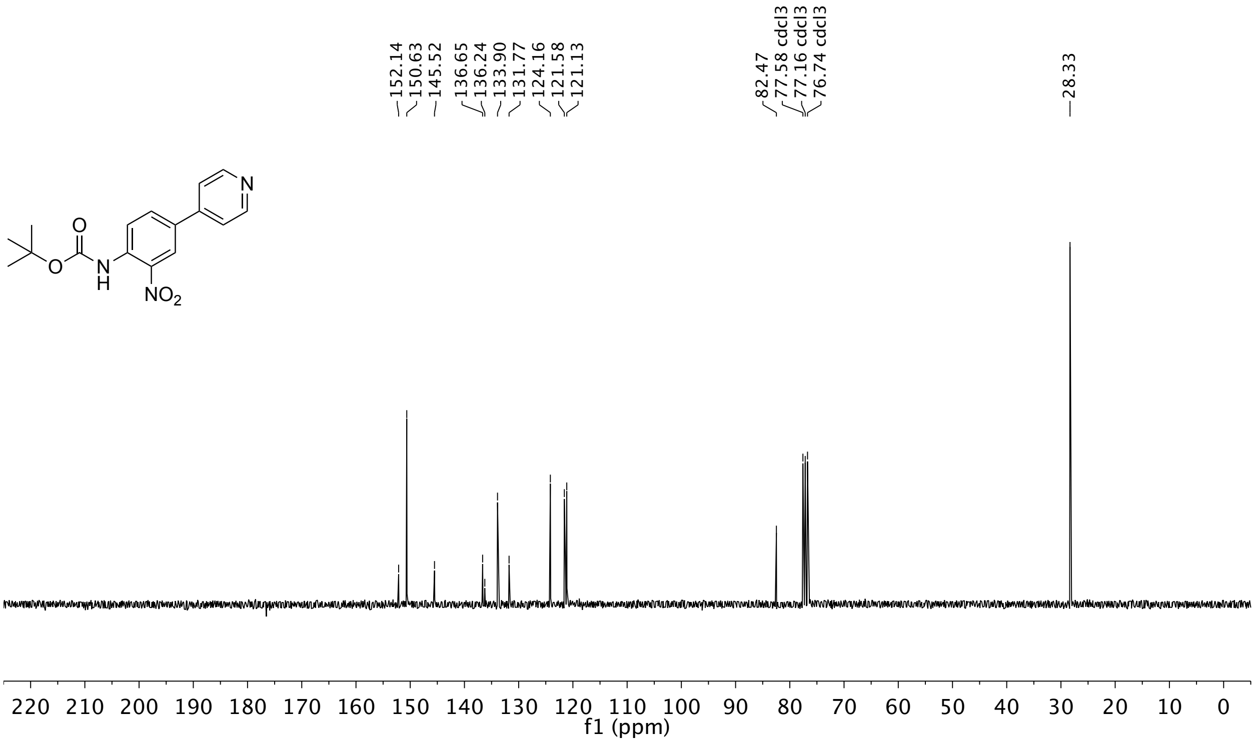

^{13}C NMR (75 MHz, Chloroform-*d*) of **3d**.

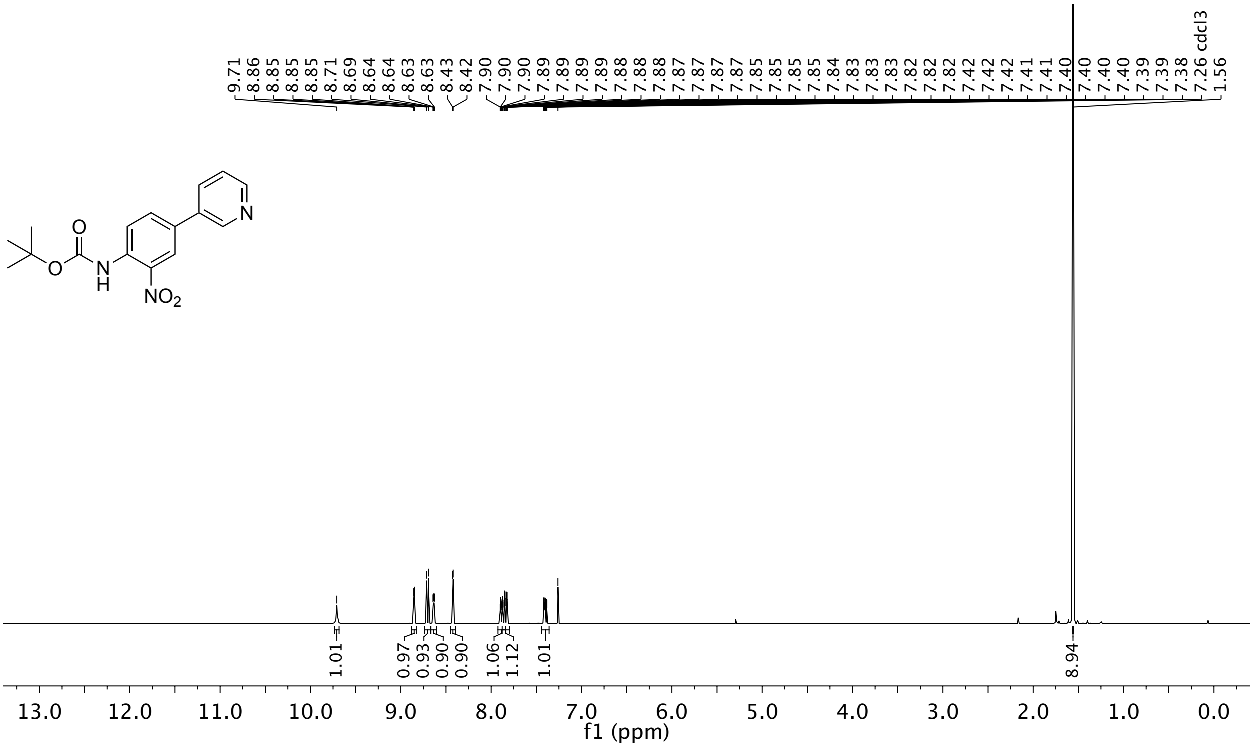

^{1}H NMR (400 MHz, Chloroform-*d*) of **3e**.

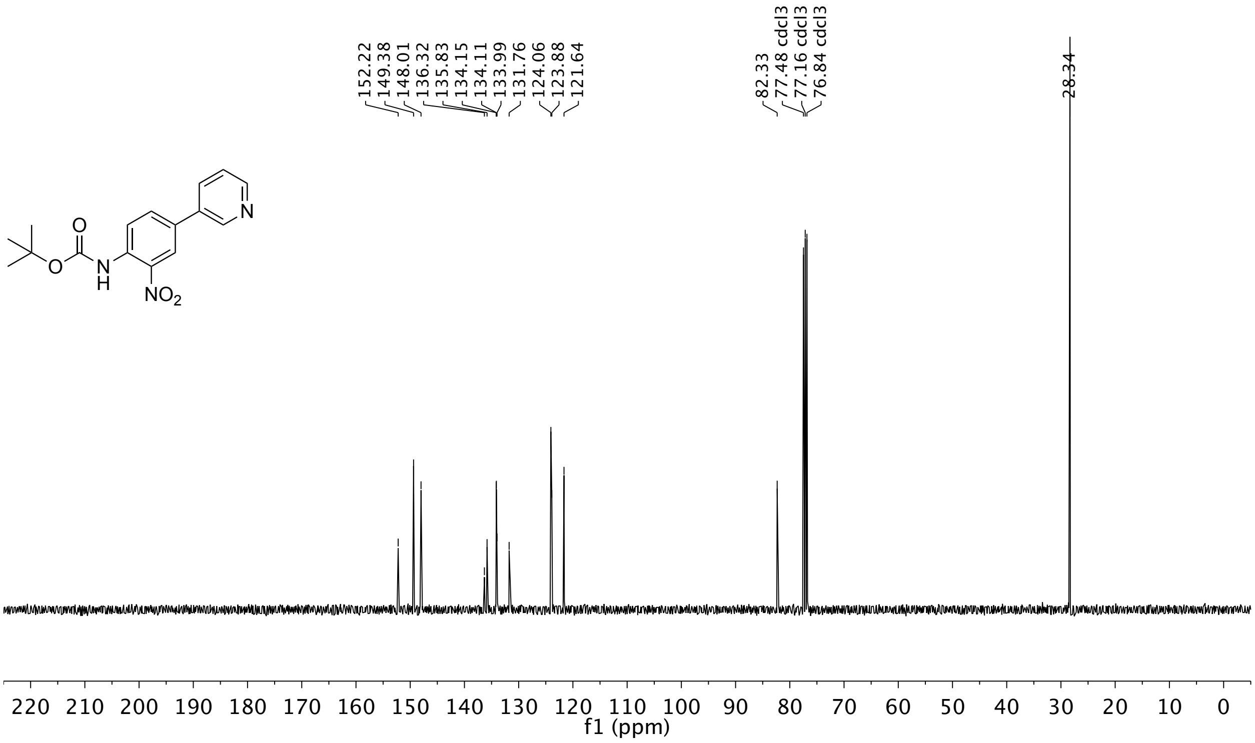

^{13}C NMR (101 MHz, Chloroform-*d*) of **3e**.

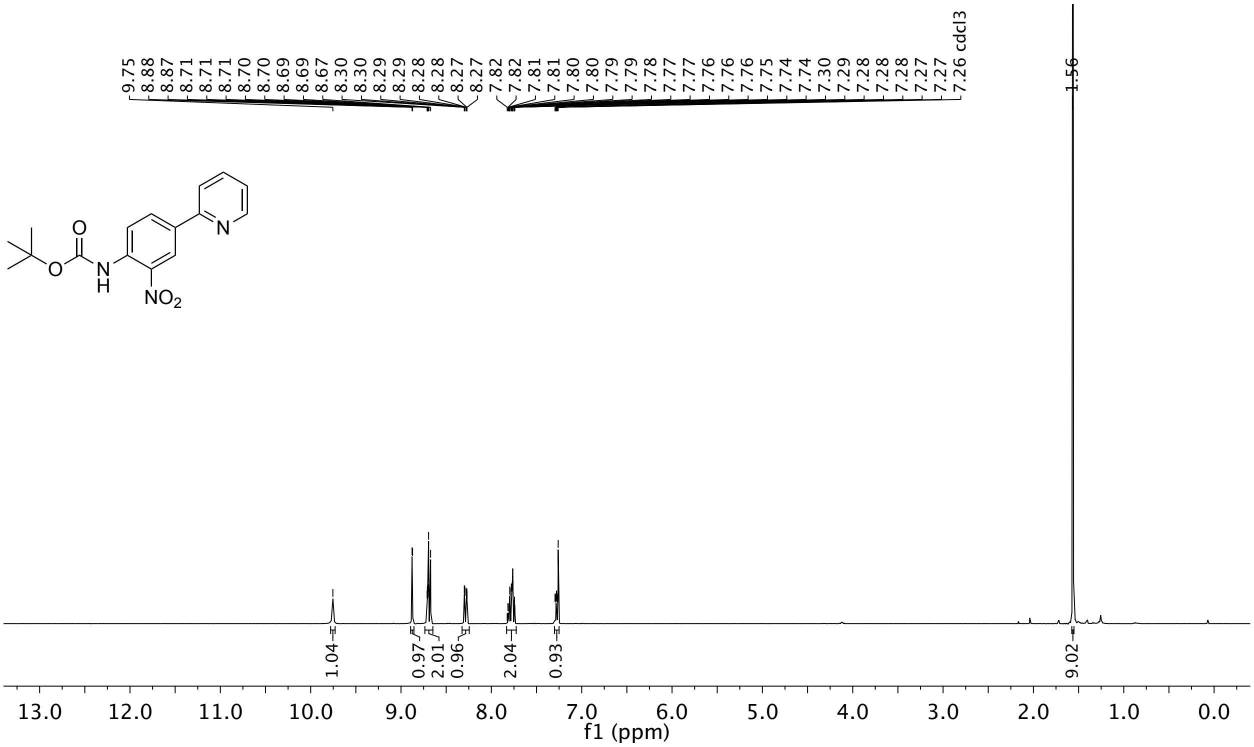

^{1}H NMR (400 MHz, Chloroform-*d*) of **3f**.

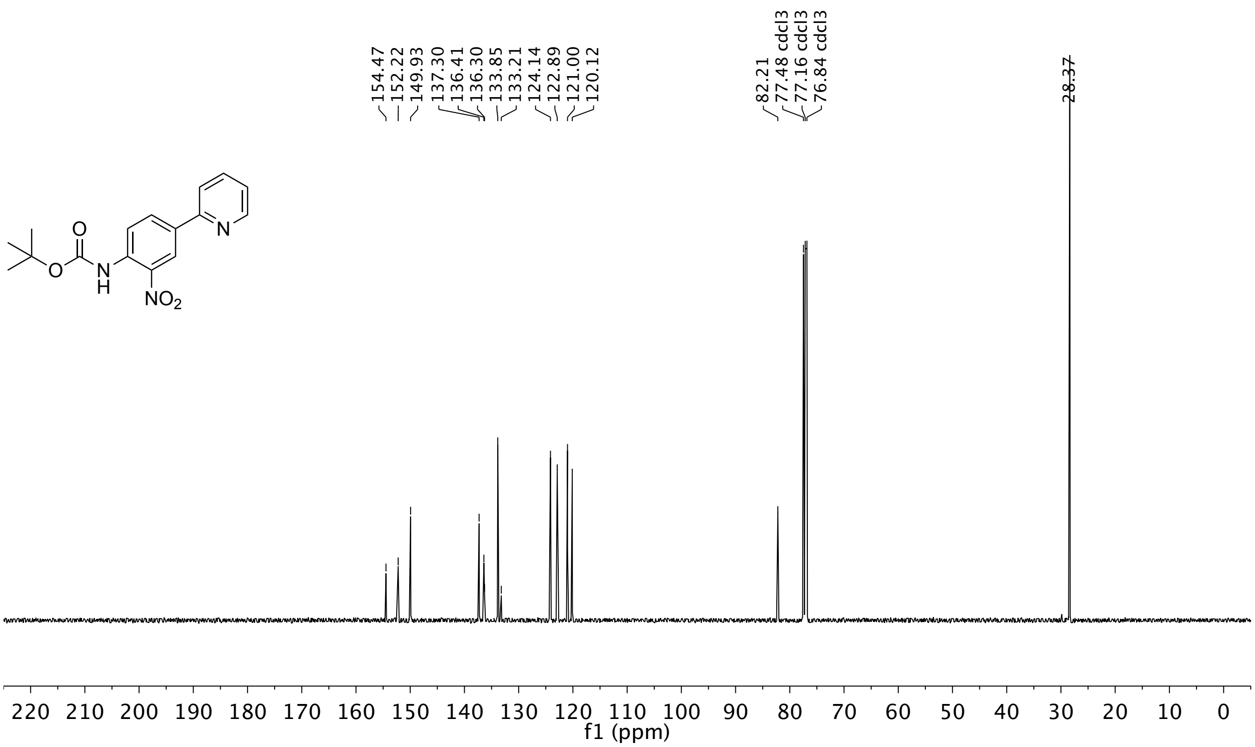

^{13}C NMR (101 MHz, Chloroform-*d*) of **3f**.

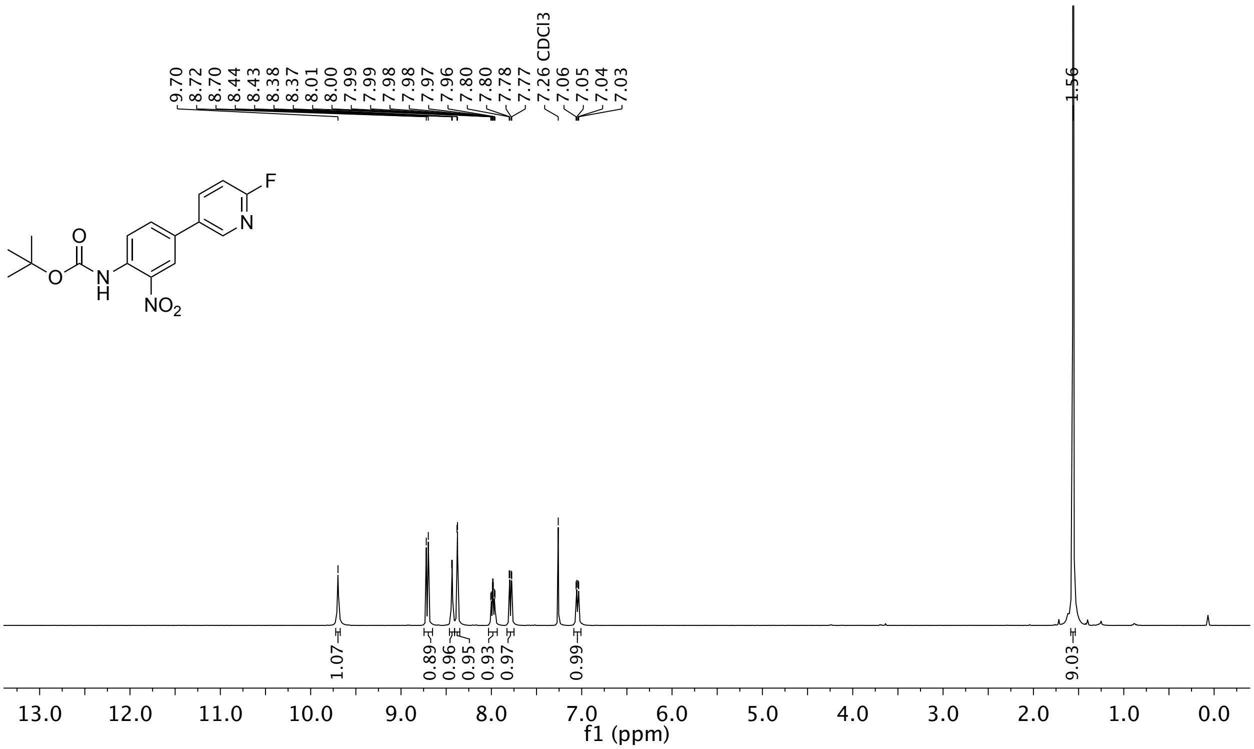

^{1}H NMR (400 MHz, Chloroform-*d*) of **3g**.

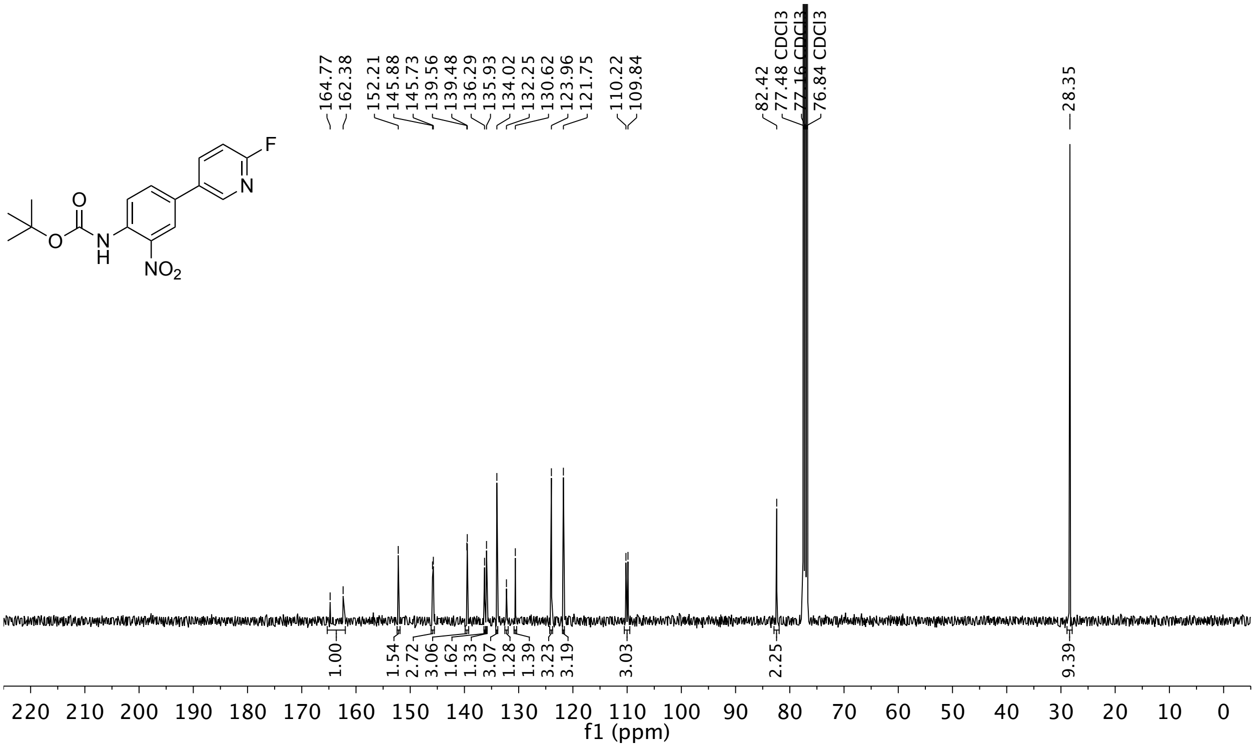

^{13}C NMR (101 MHz, Chloroform-*d*) of **3g**.

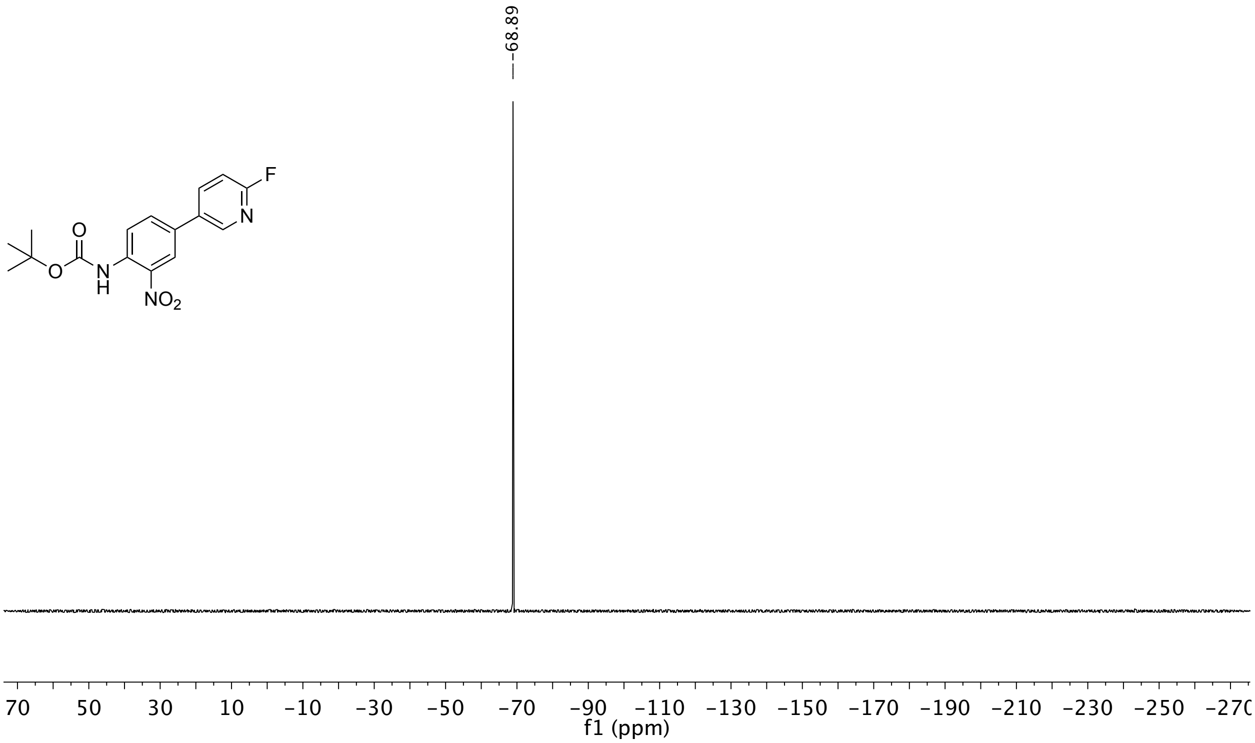

^{19}F NMR (377 MHz, Chloroform-*d*) of **3g**.

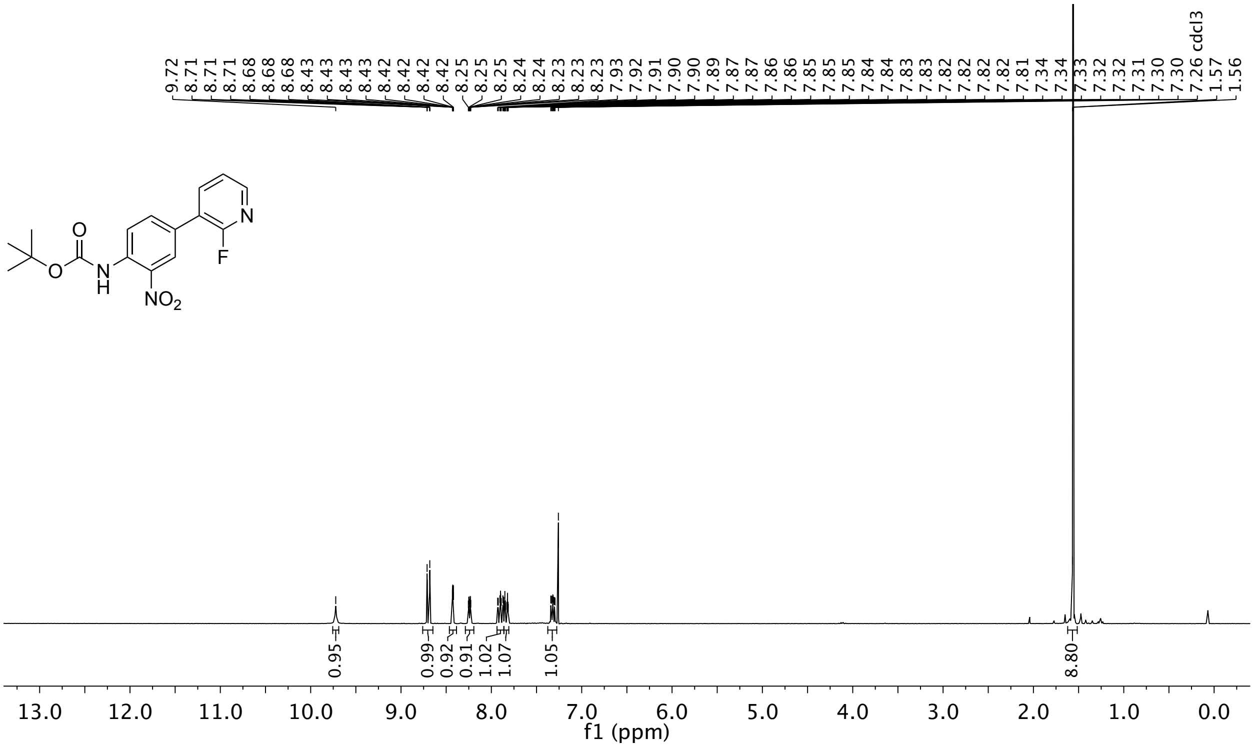

^{1}H NMR (300 MHz, Chloroform-*d*) of **3h**.

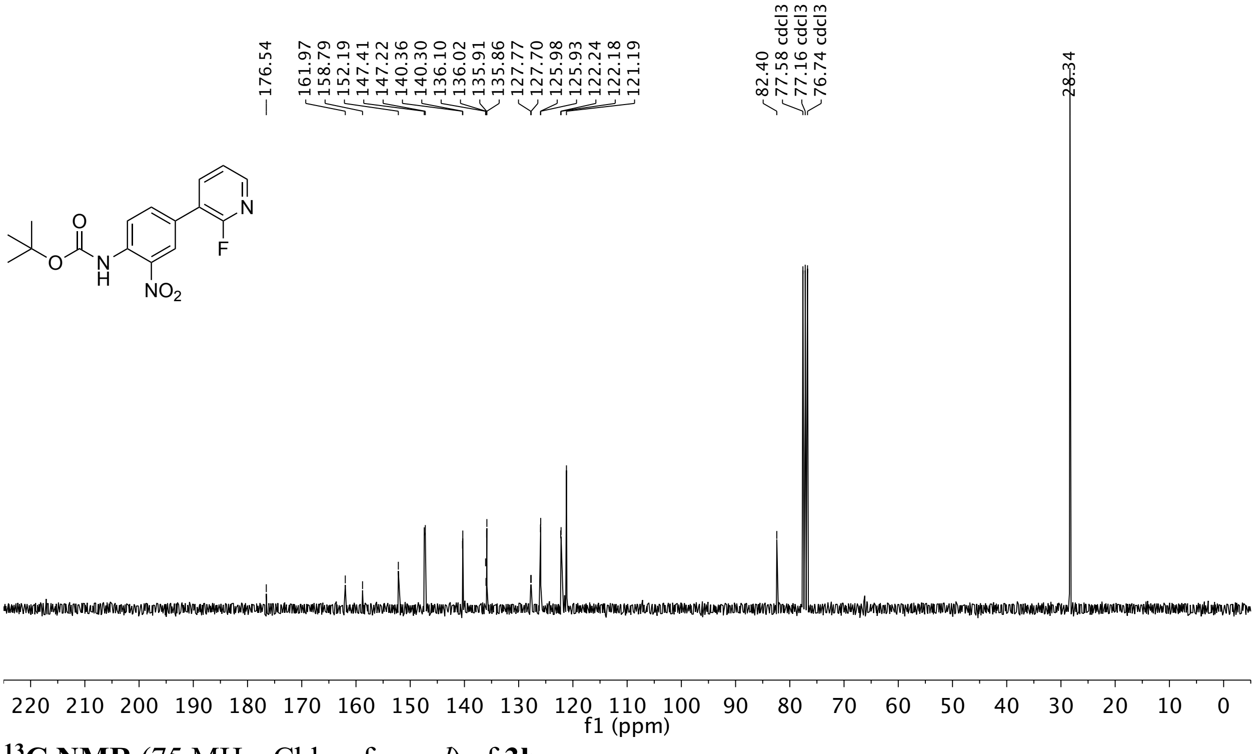

^{13}C NMR (75 MHz, Chloroform-*d*) of **3h**.

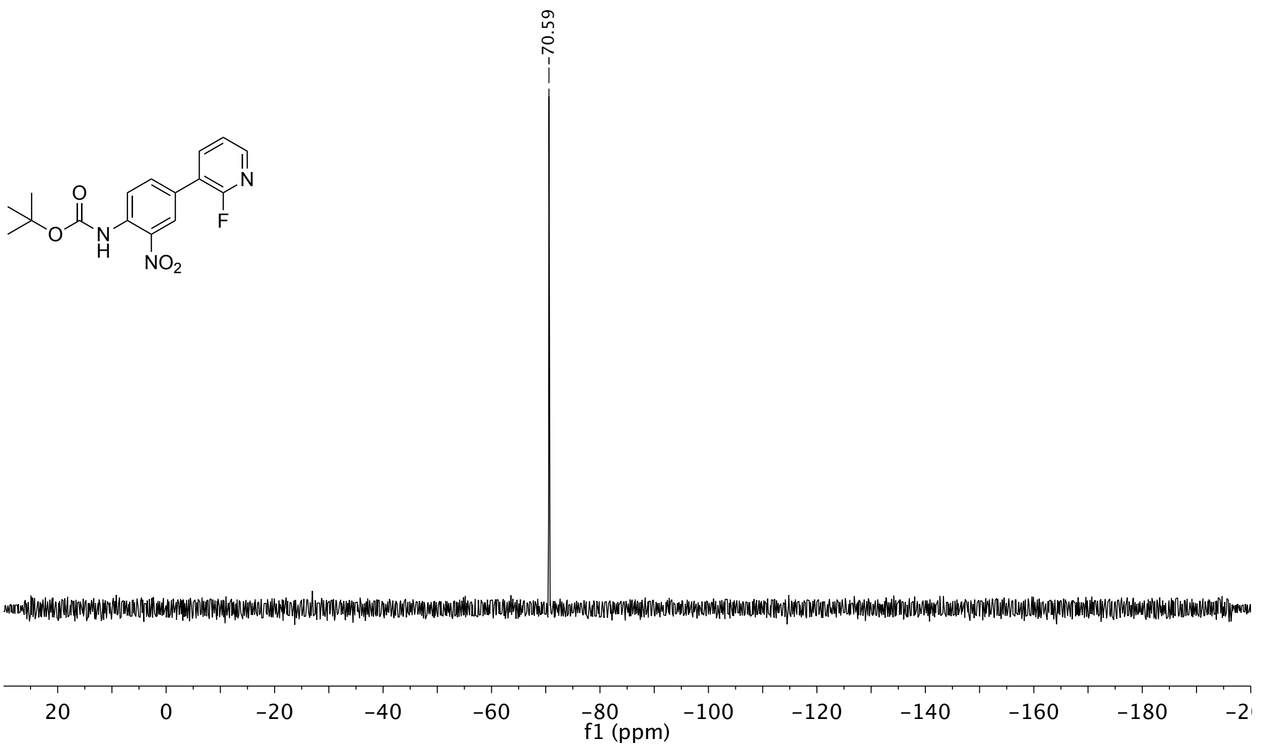

^{19}F NMR (282 MHz, Chloroform-*d*) of **3h**.

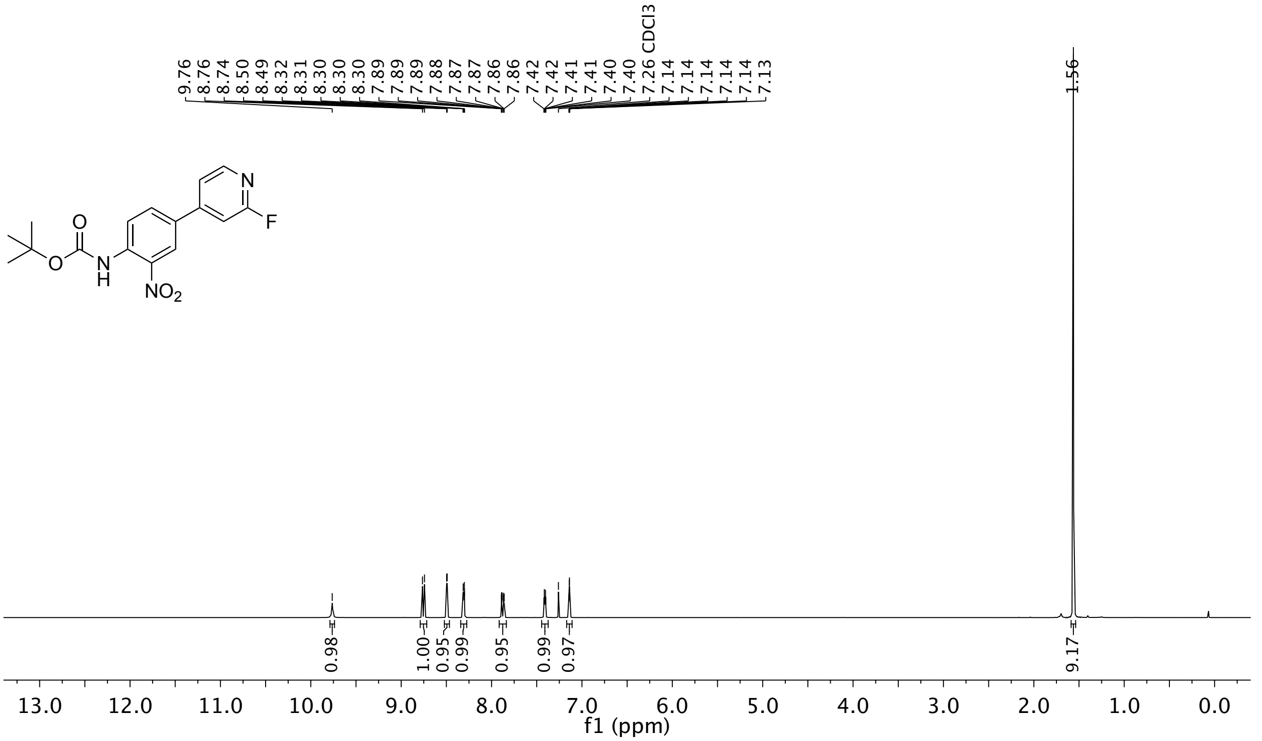

^{1}H NMR (400 MHz, Chloroform-*d*) of **3i**.

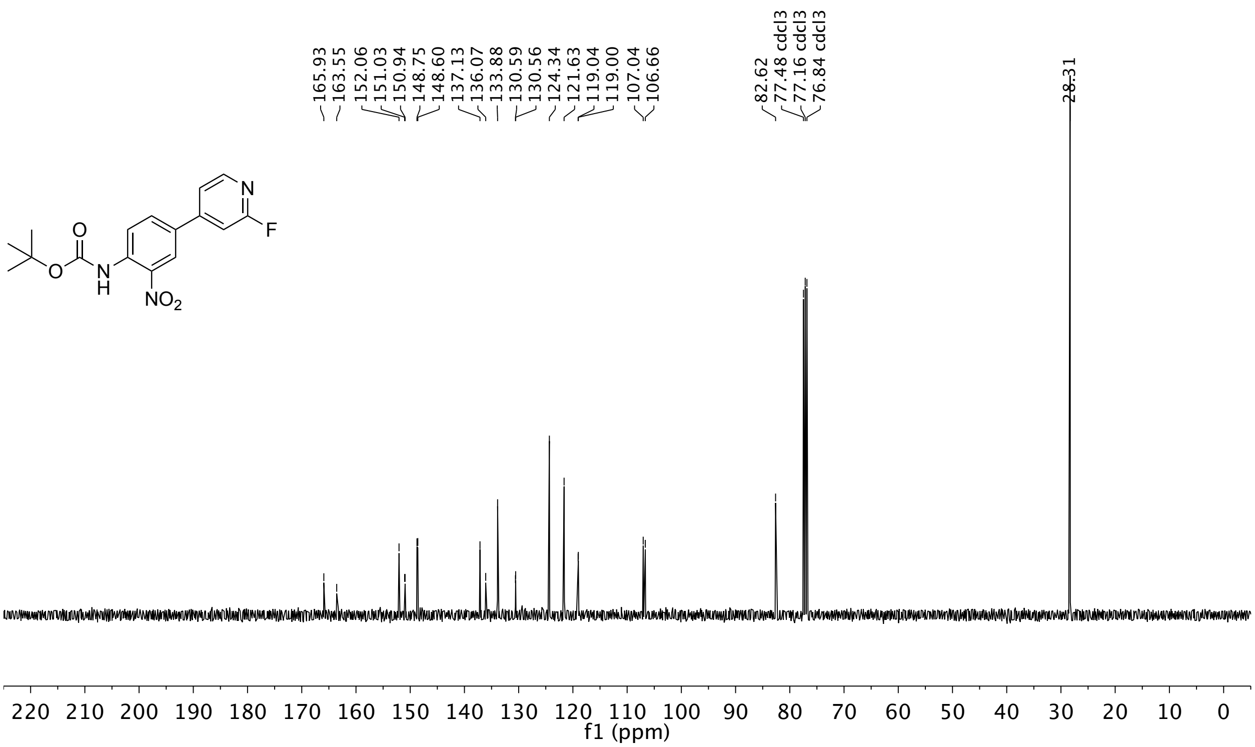

^{13}C NMR (101 MHz, Chloroform-*d*) of **3i**.

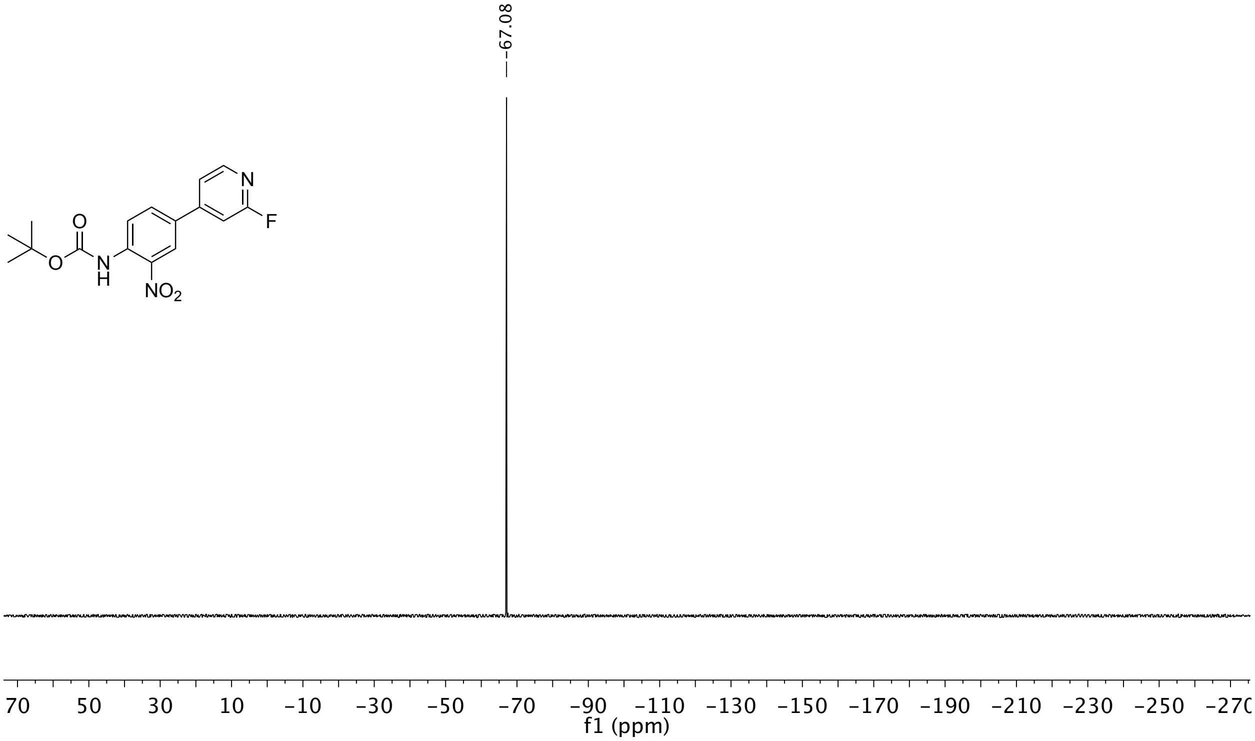

^{19}F NMR (377 MHz, Chloroform-*d*) of **3i**.

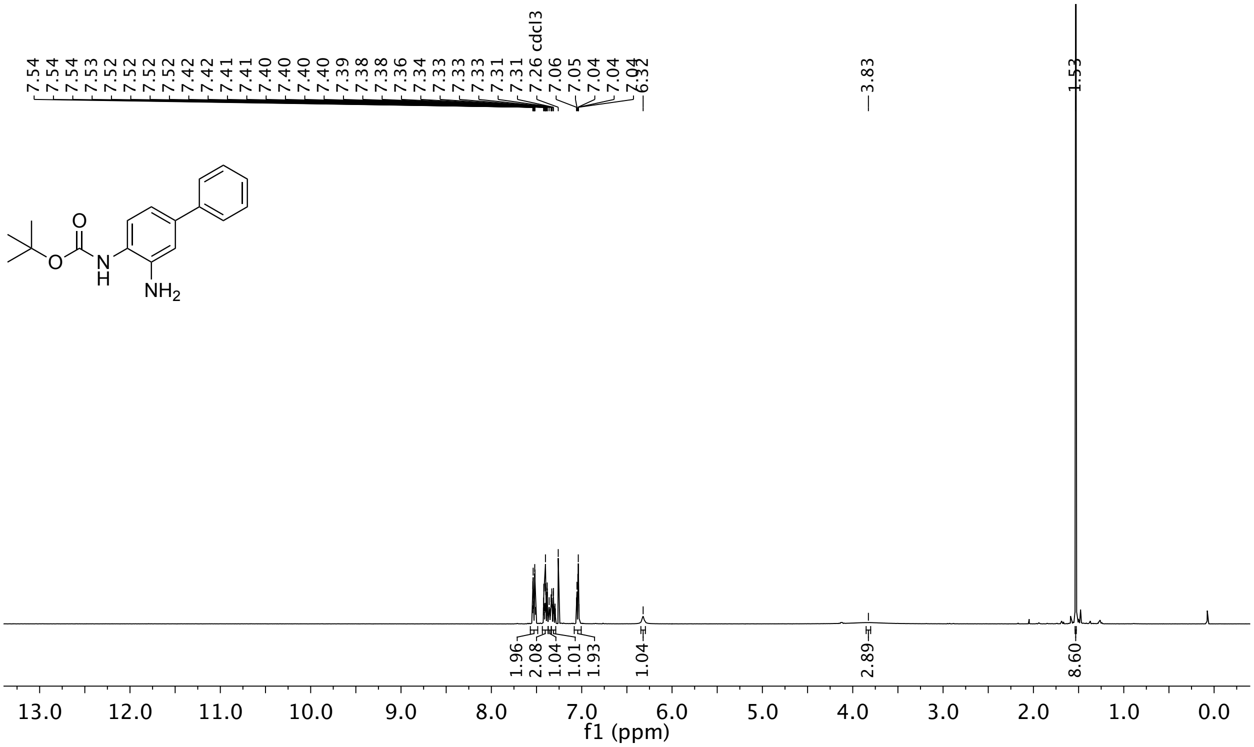

^{1}H NMR (400 MHz, Chloroform-*d*) of **4a**.

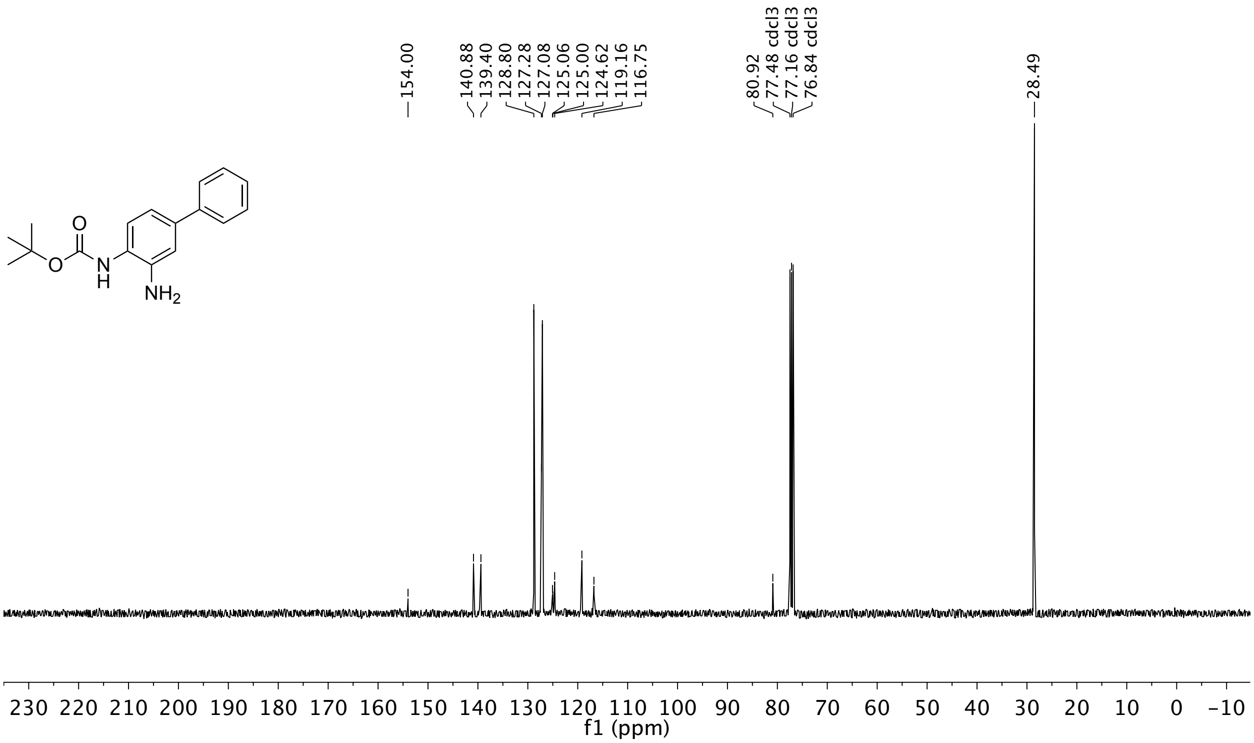

^{13}C NMR (101 MHz, Chloroform-*d*) of **4a**.

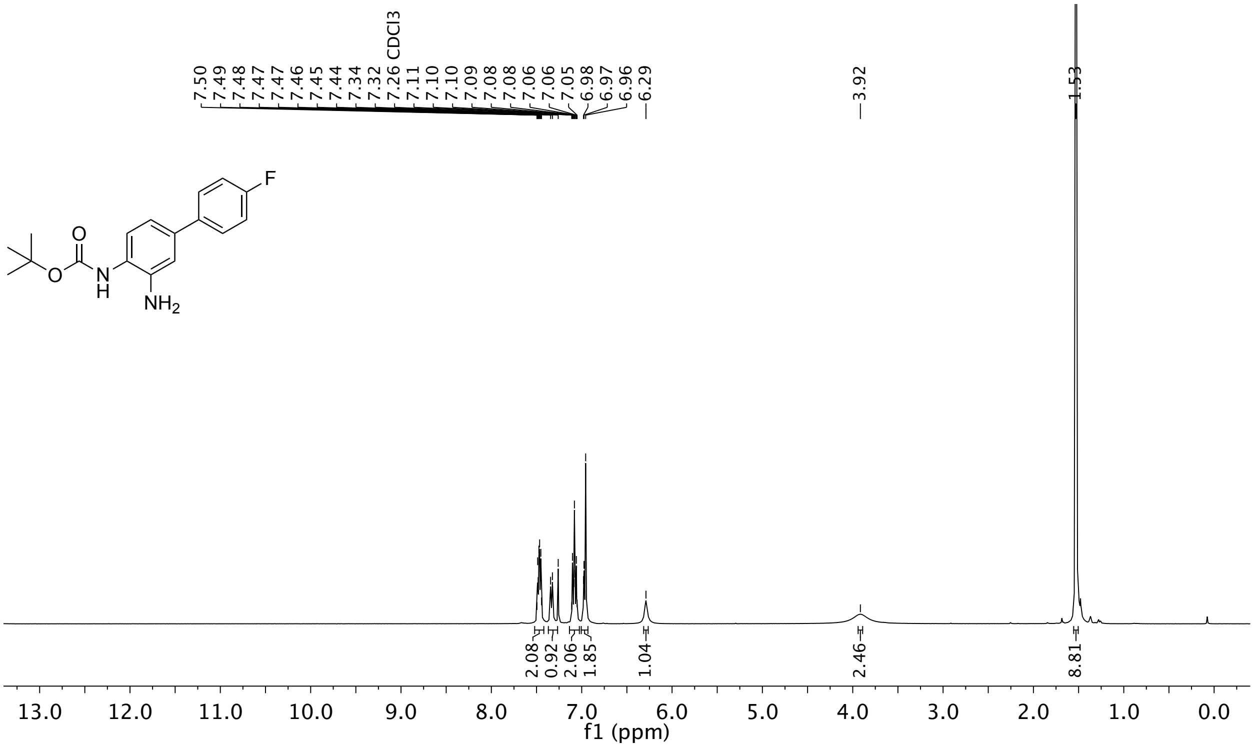

^{1}H NMR (400 MHz, Chloroform-*d*) of **4b**.

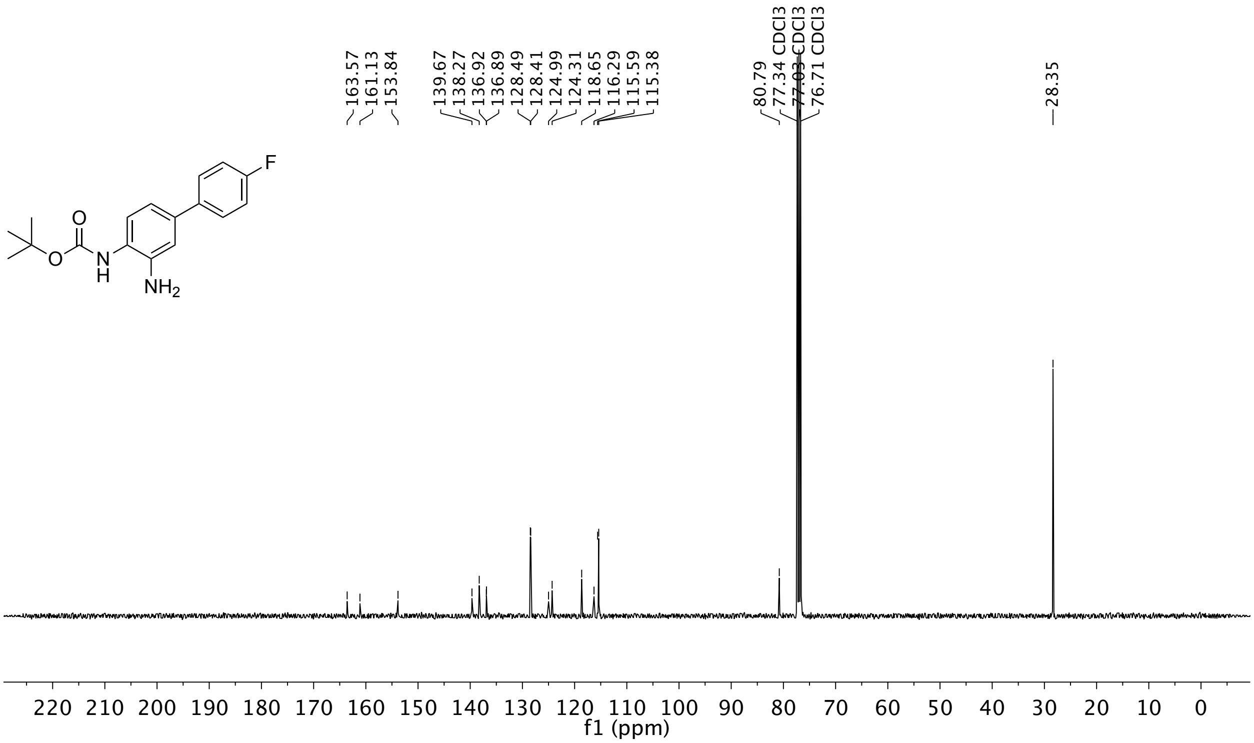

^{13}C NMR (101 MHz, Chloroform-*d*) of **4b**.

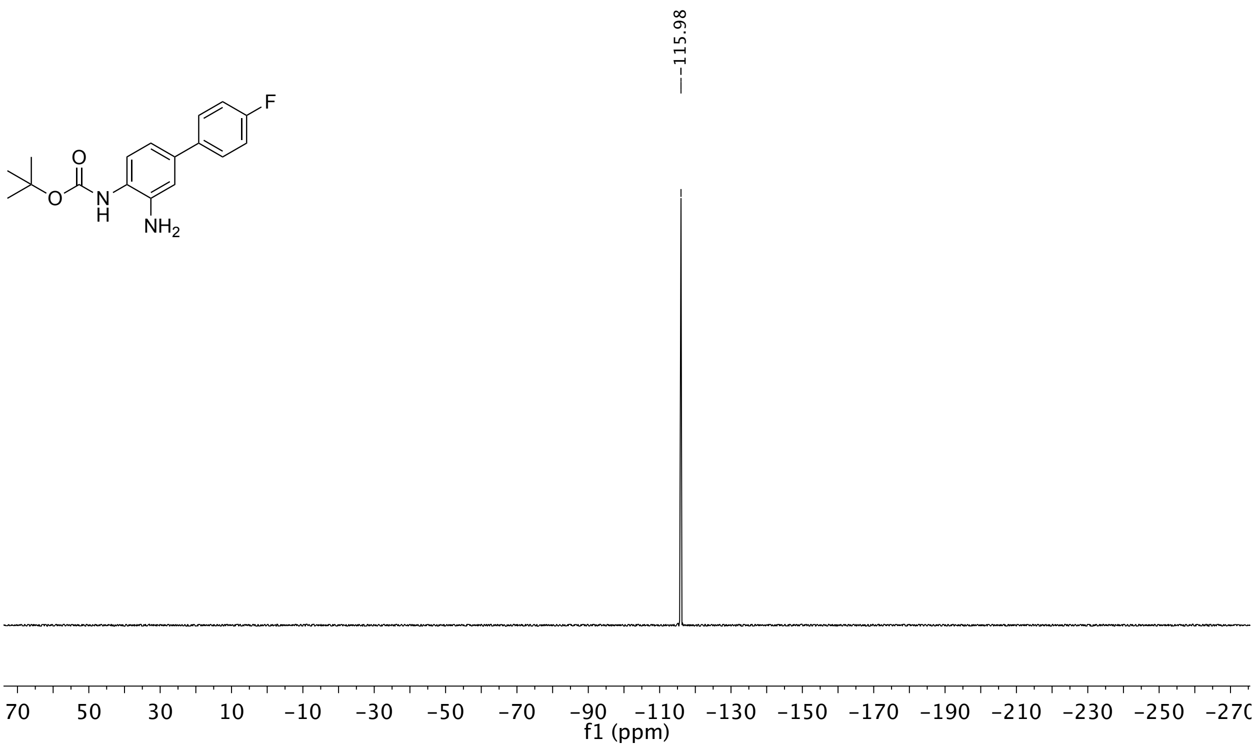

^{19}F NMR (377 MHz, Chloroform-*d*) of **4b**.

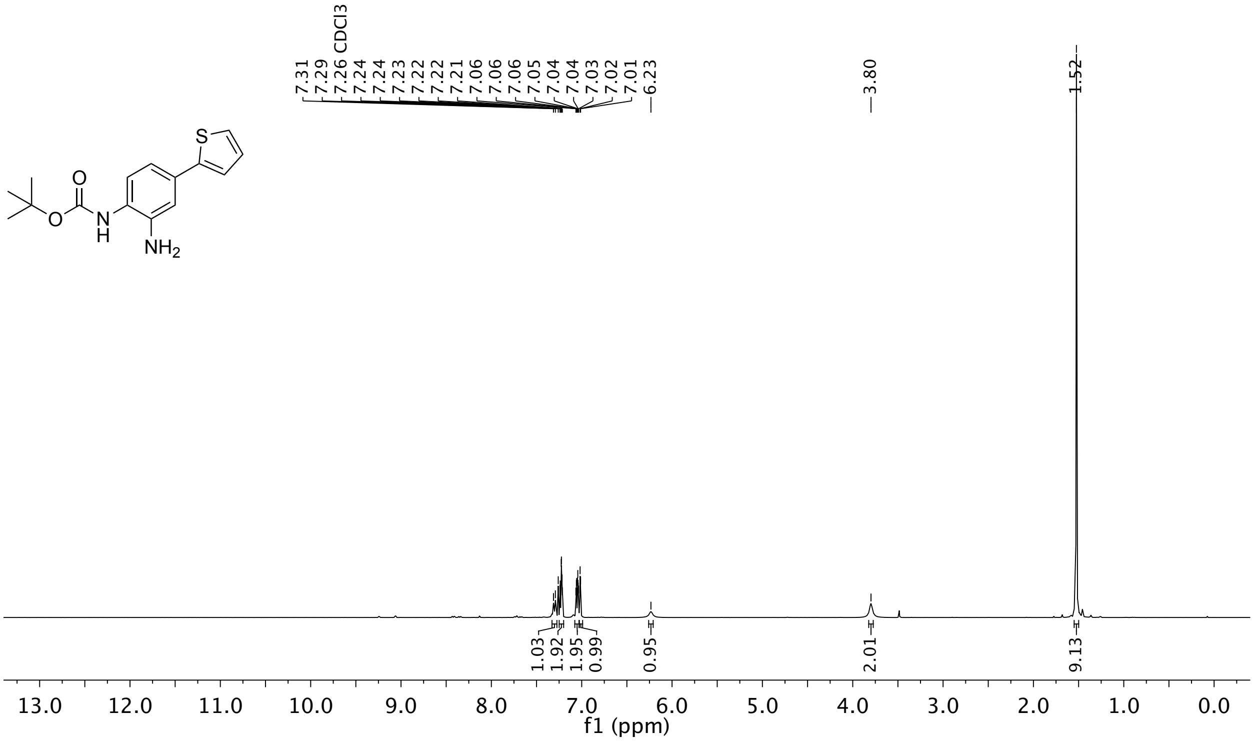

^{1}H NMR (400 MHz, Chloroform-*d*) of **4c**.

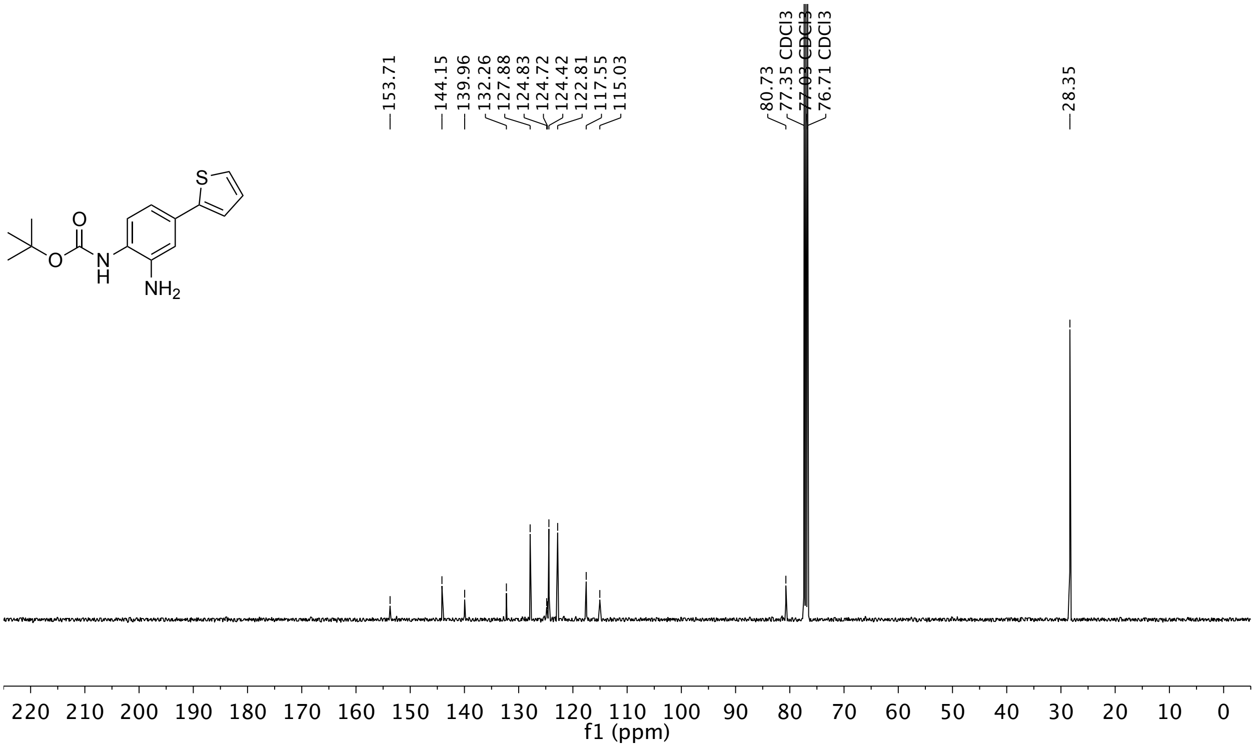

^{13}C NMR (101 MHz, Chloroform-*d*) of **4c**.

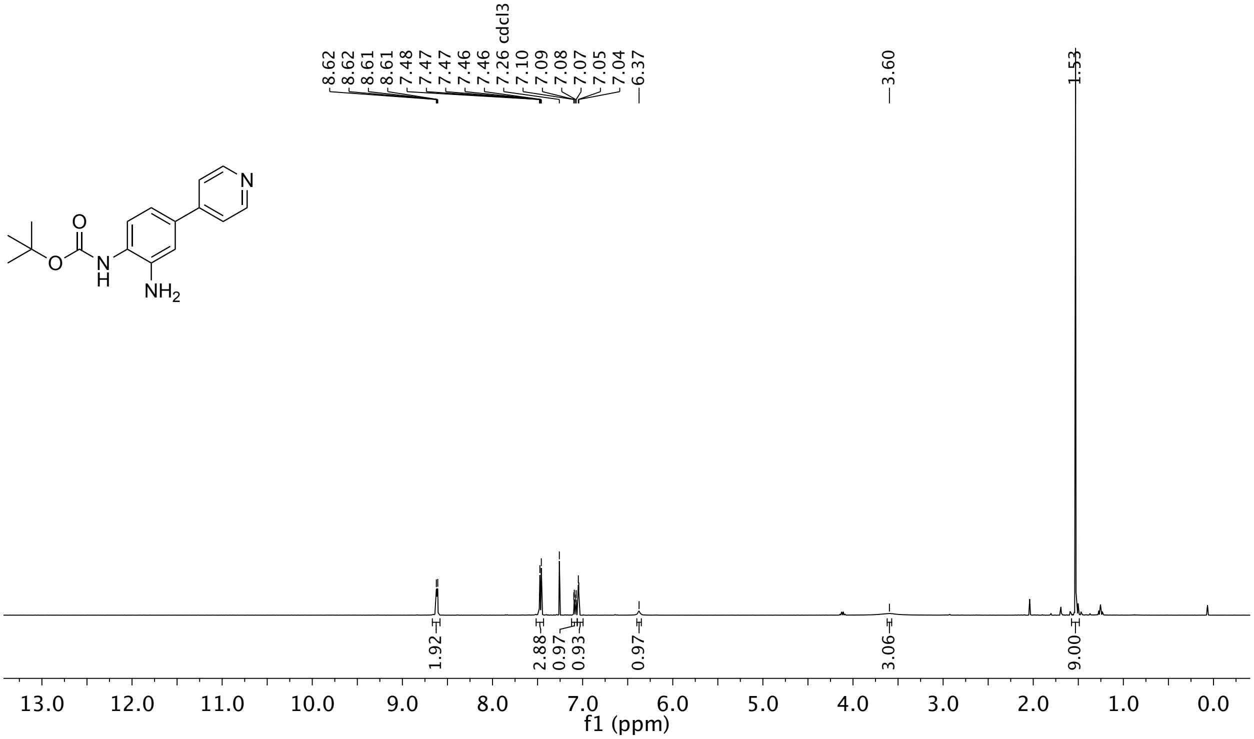

^{1}H NMR (400 MHz, Chloroform-*d*) of **4d**.

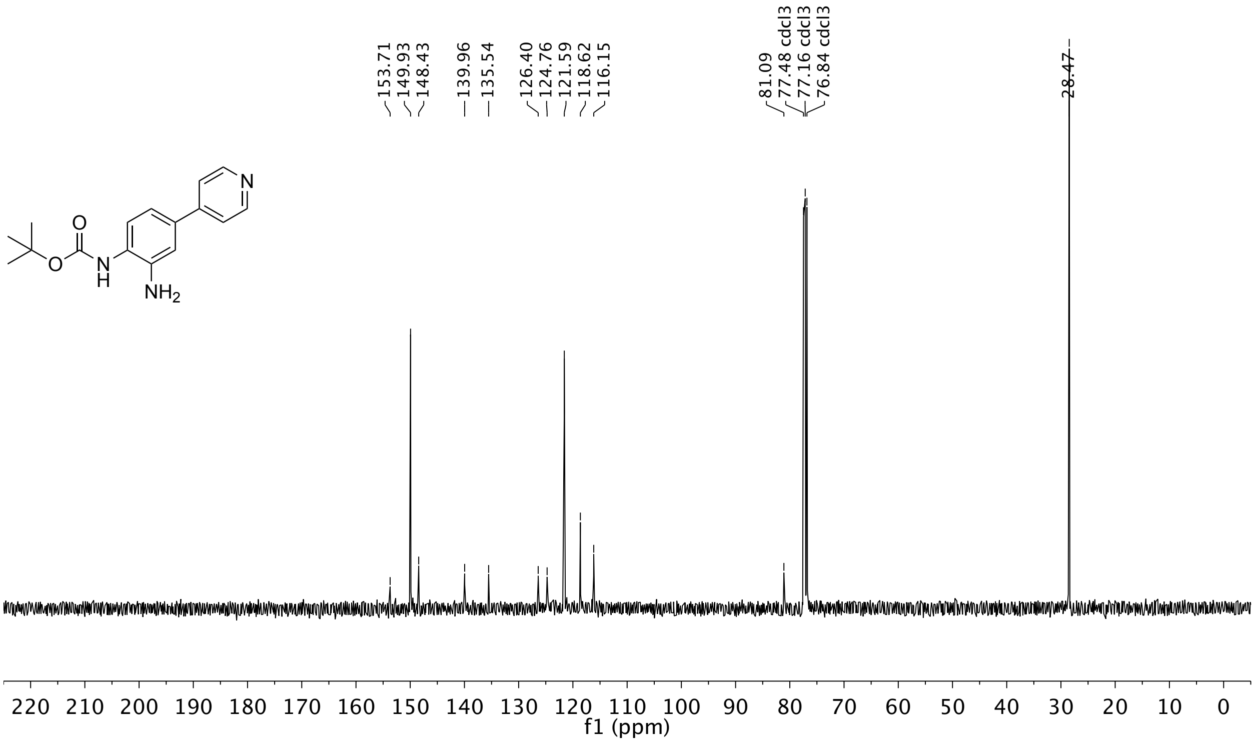

^{13}C NMR (101 MHz, Chloroform-*d*) of **4d**.

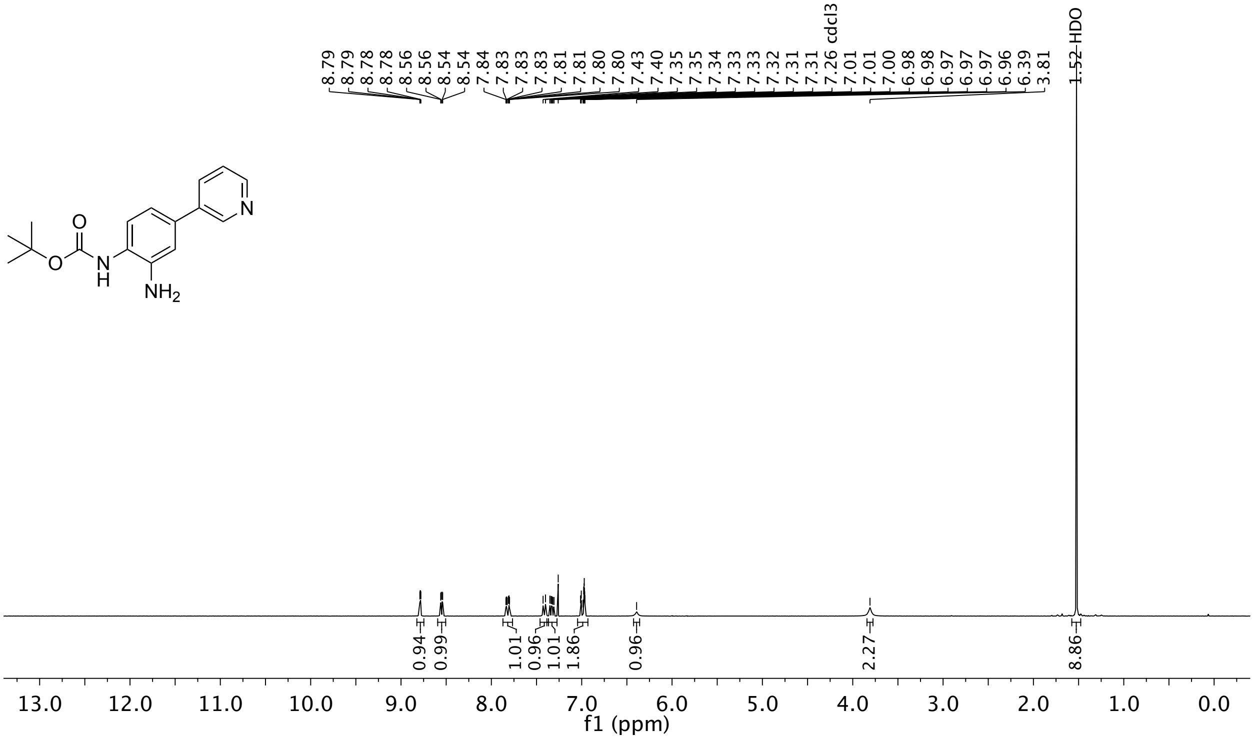

^{1}H NMR (300 MHz, Chloroform-*d*) of **4e**.

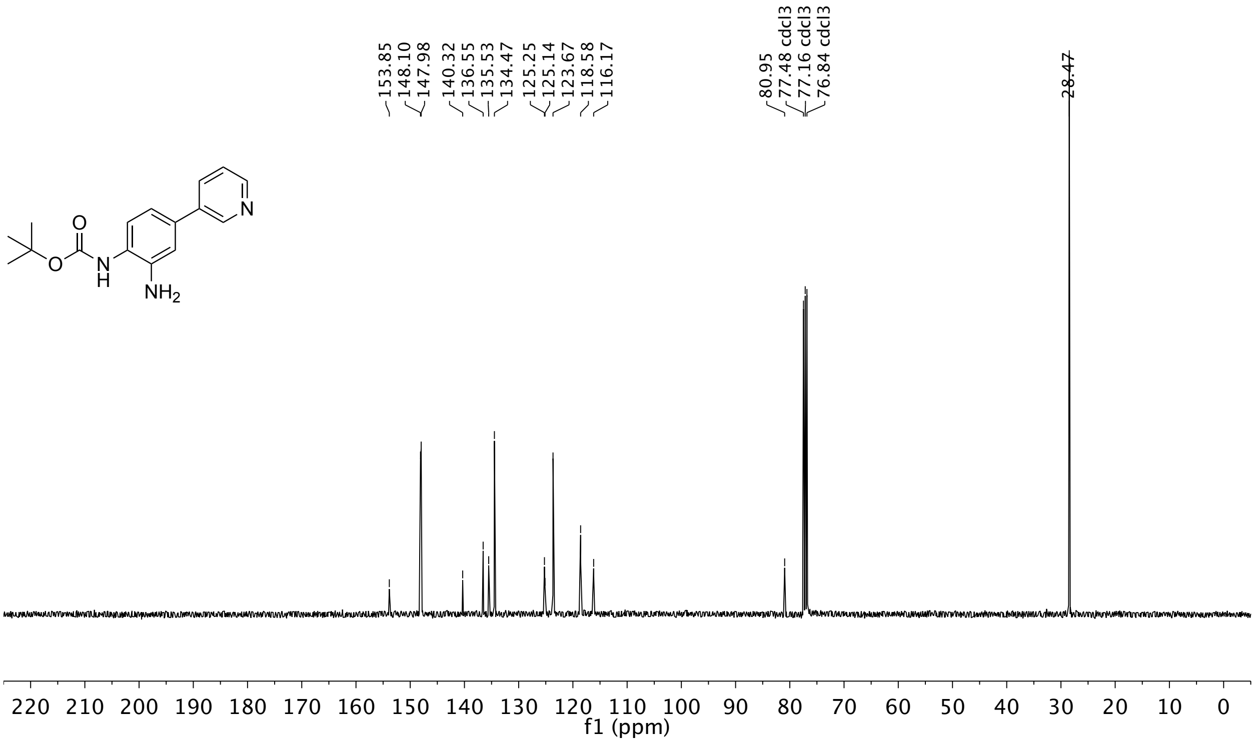

^{13}C NMR (101 MHz, Chloroform-*d*) of **4e**.

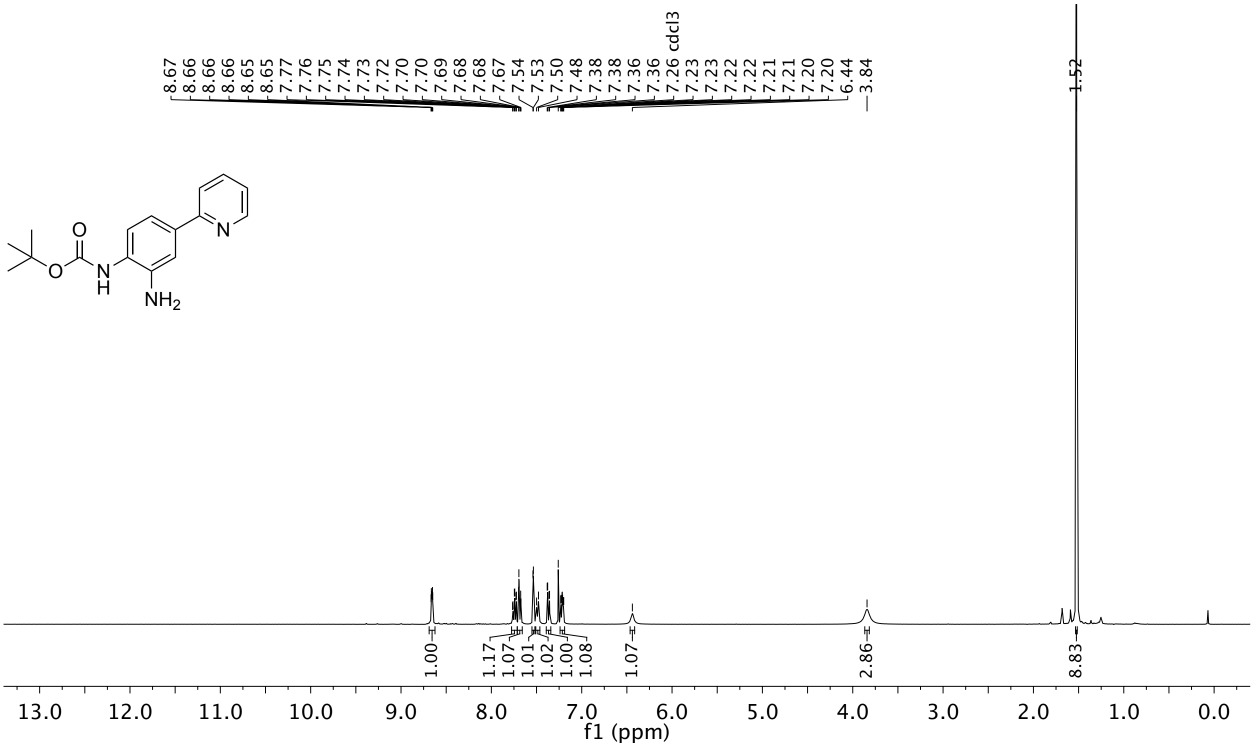

^{1}H NMR (400 MHz, Chloroform-*d*) of **4f**.

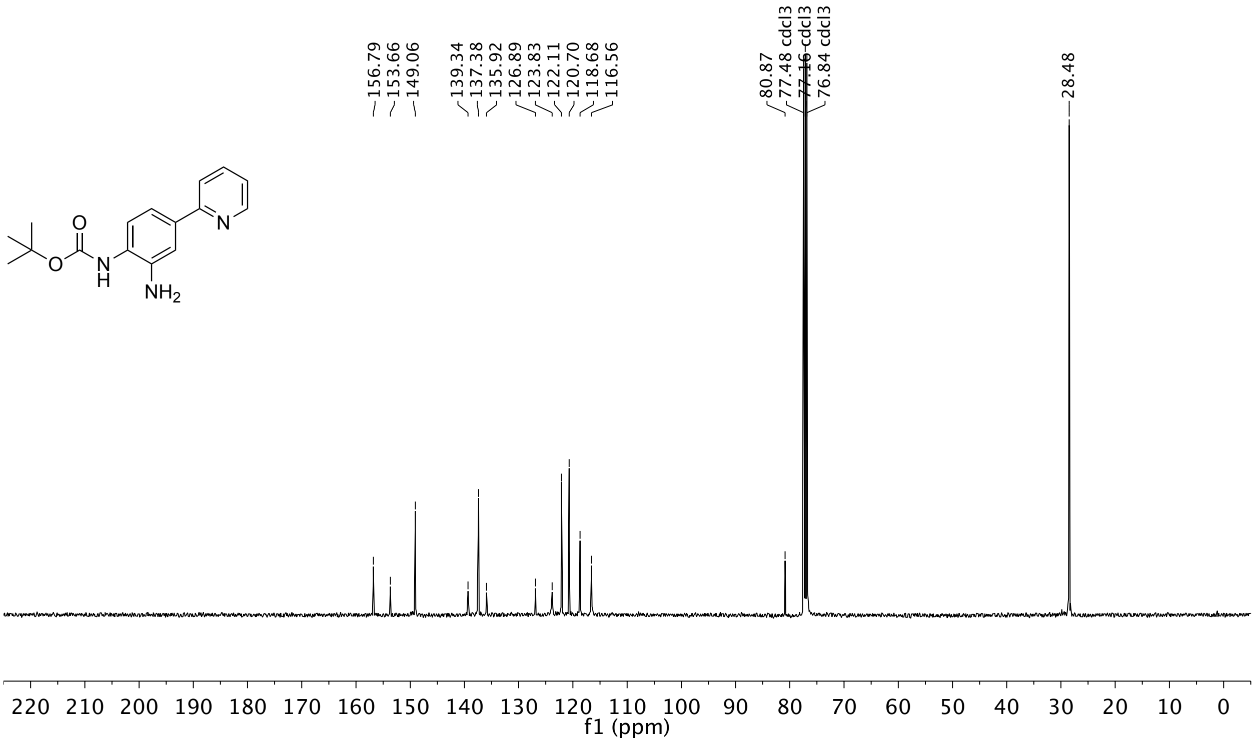

^{13}**C NMR** (101 MHz, Chloroform-*d*) of **4f**.

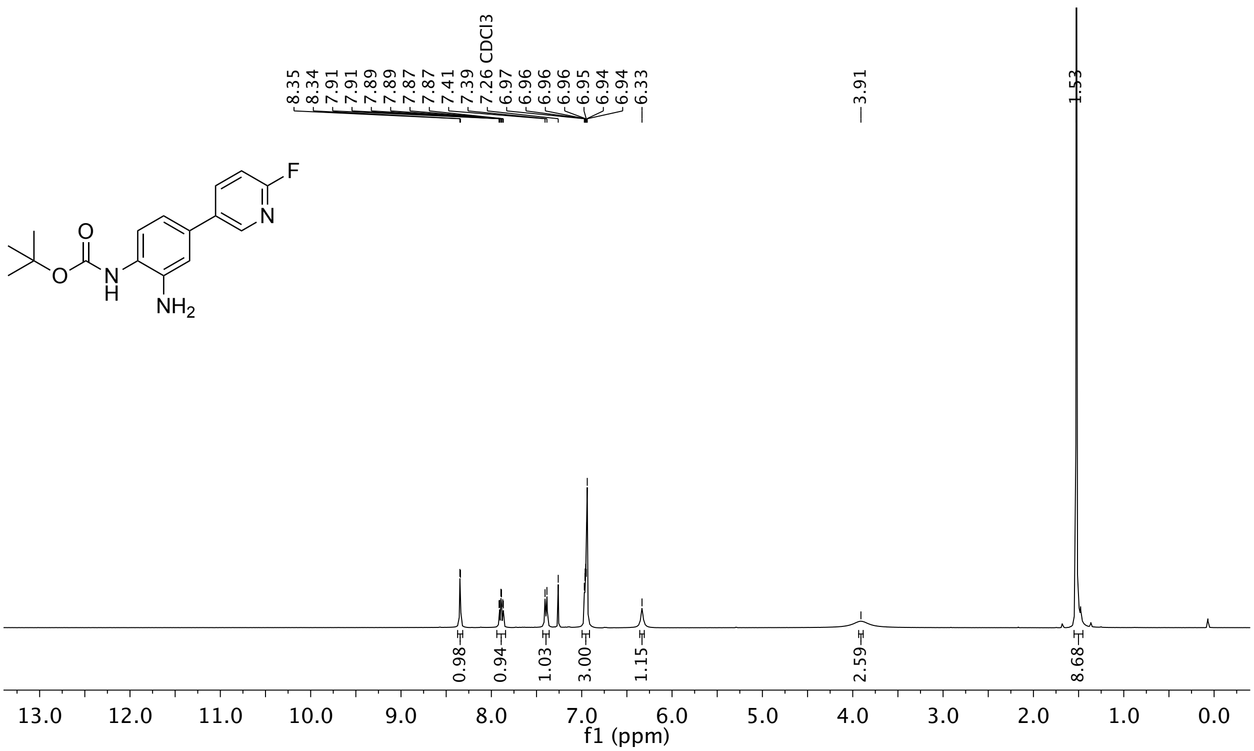

^{1}**H NMR** (400 MHz, Chloroform-*d*) of **4g**.

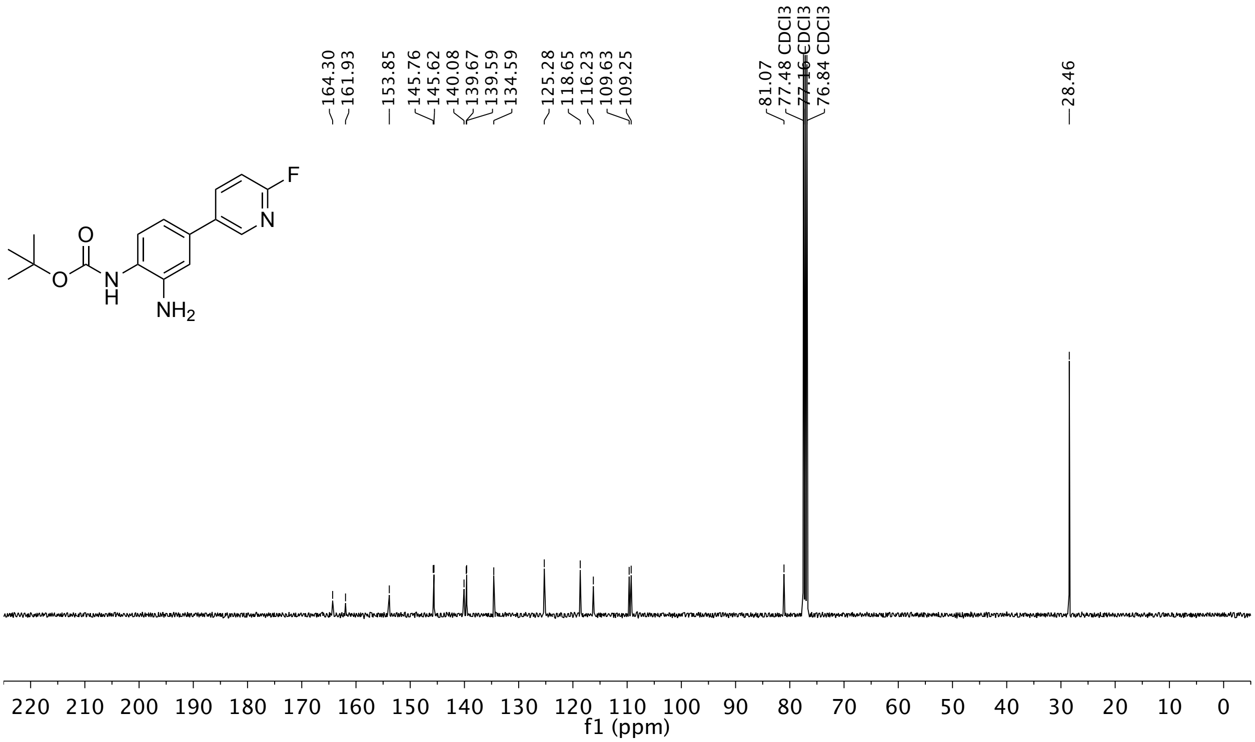

^{13}C NMR (101 MHz, Chloroform-*d*) of **4g**.

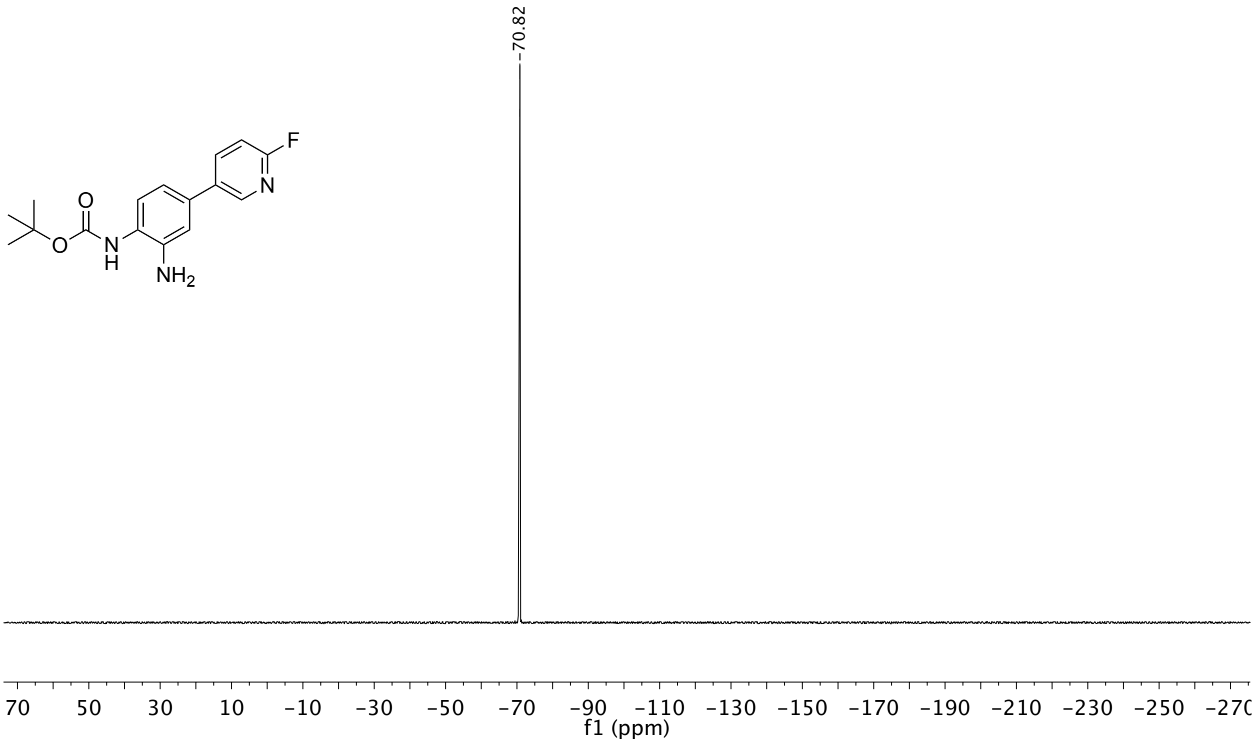

^{19}F NMR (377 MHz, Chloroform-*d*) of **4g**.

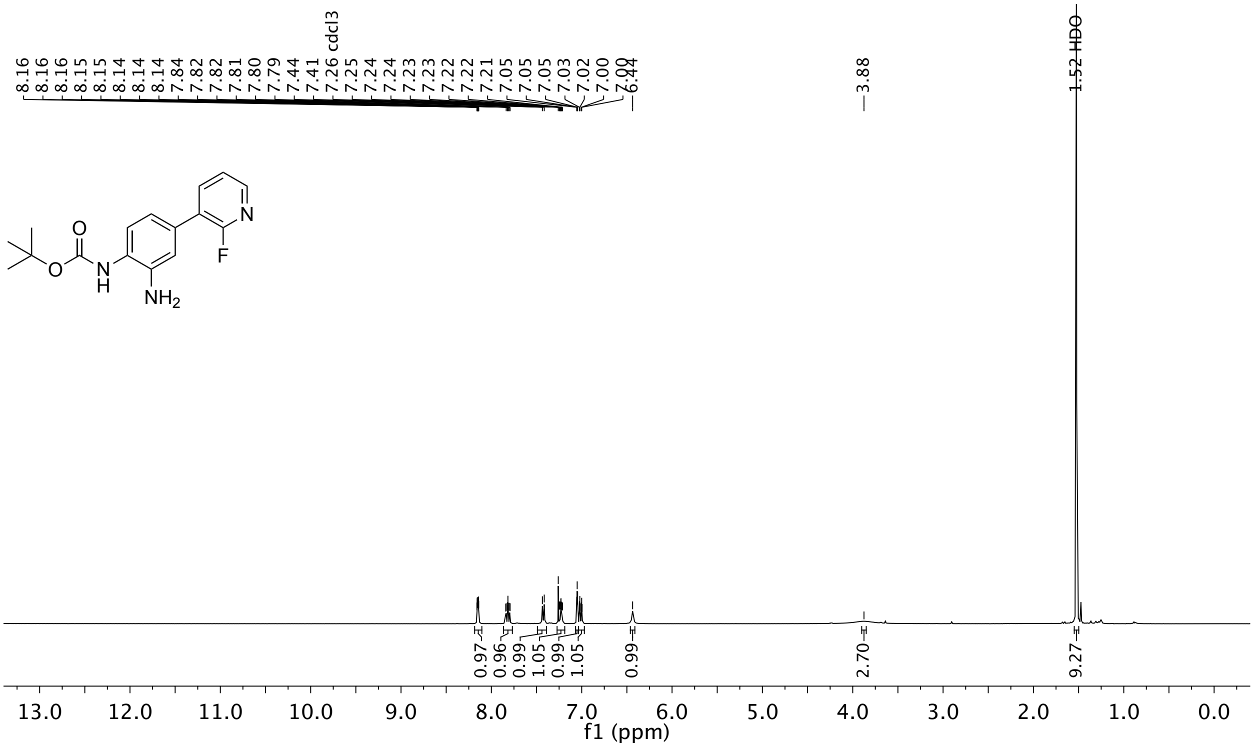

^{1}H NMR (400 MHz, Chloroform-*d*) of **4h**.

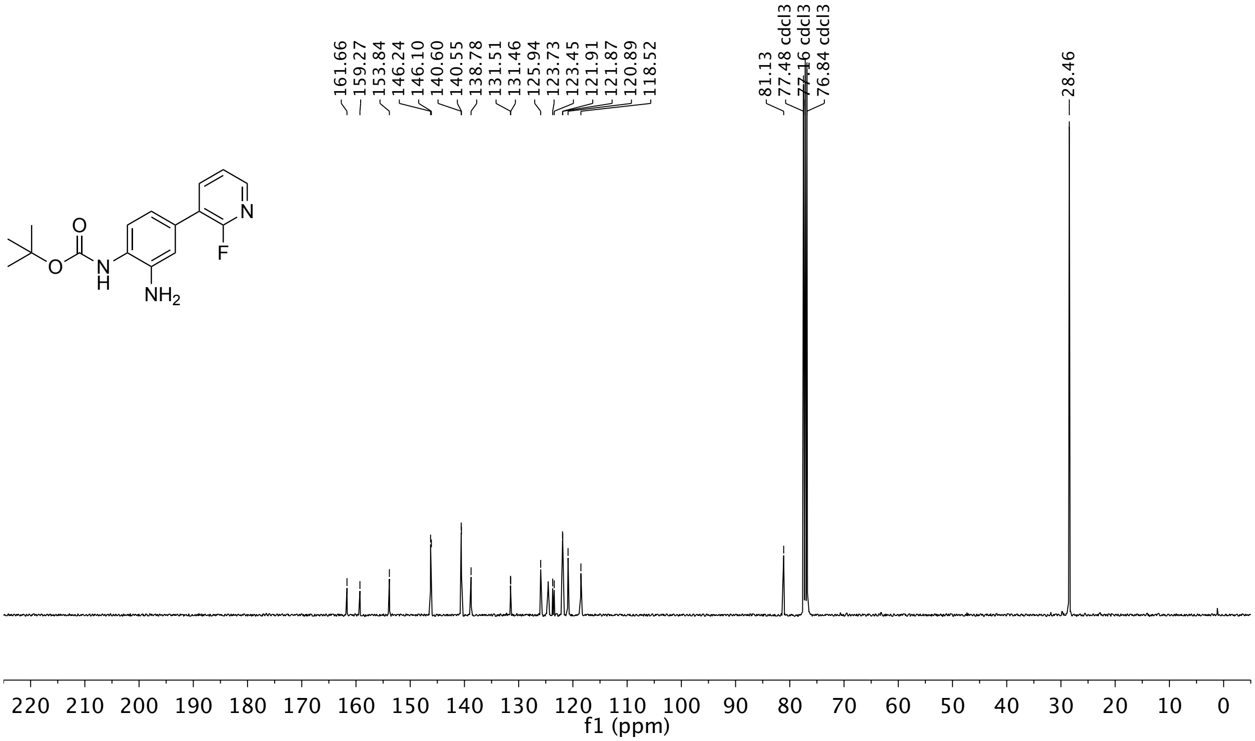

^{13}C NMR (101 MHz, Chloroform-*d*) of **4h**.

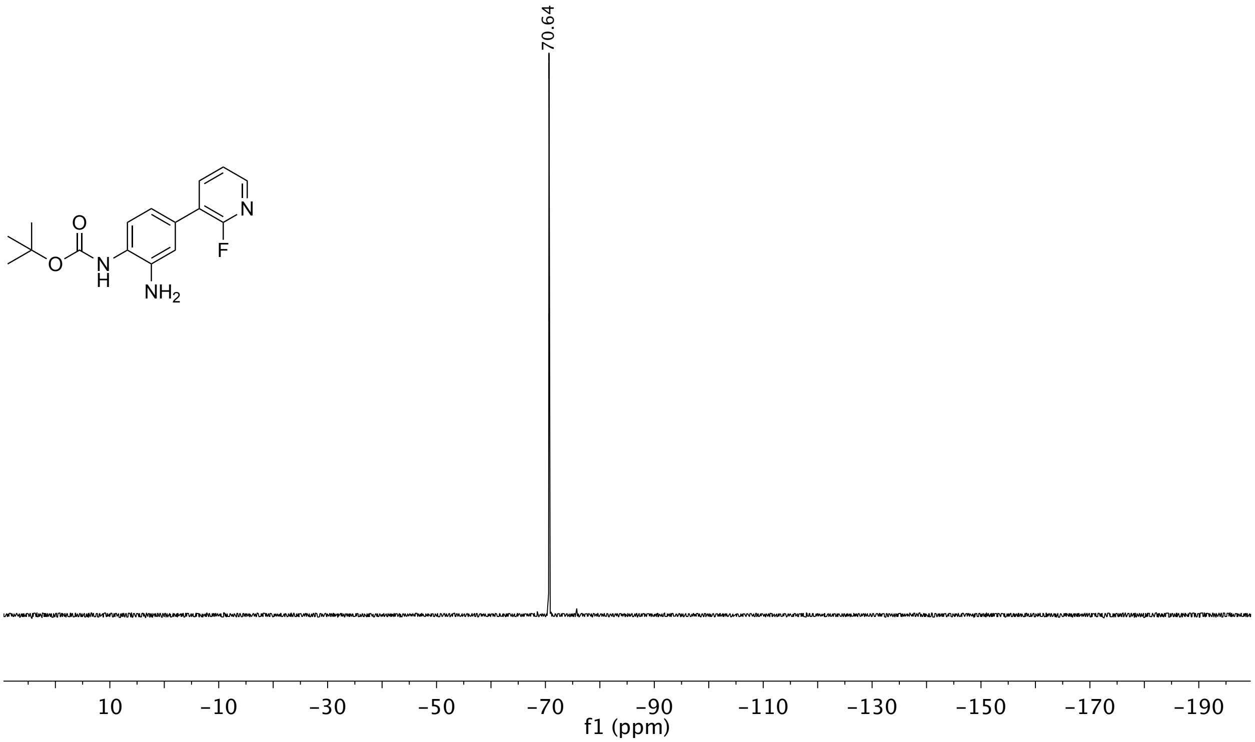

^{19}F NMR (376 MHz, Chloroform-*d*) of **4h**.

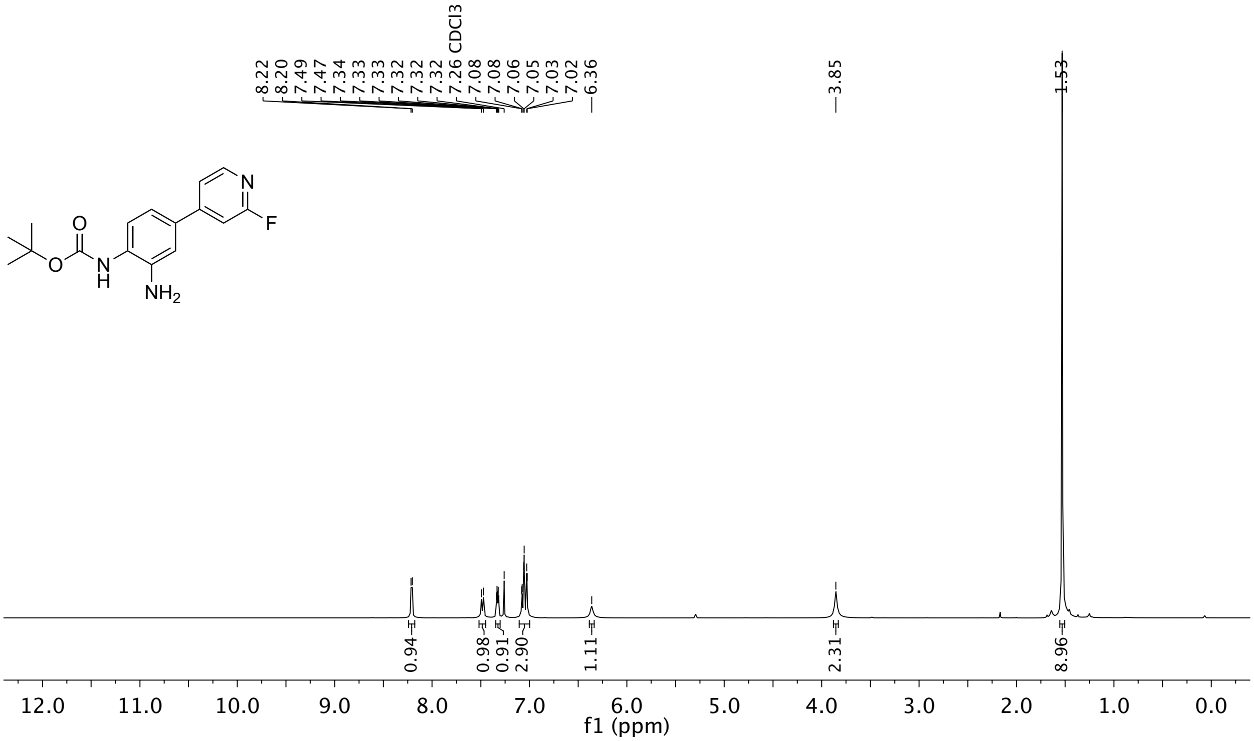

^{1}H NMR (400 MHz, Chloroform-*d*) of **4i**.

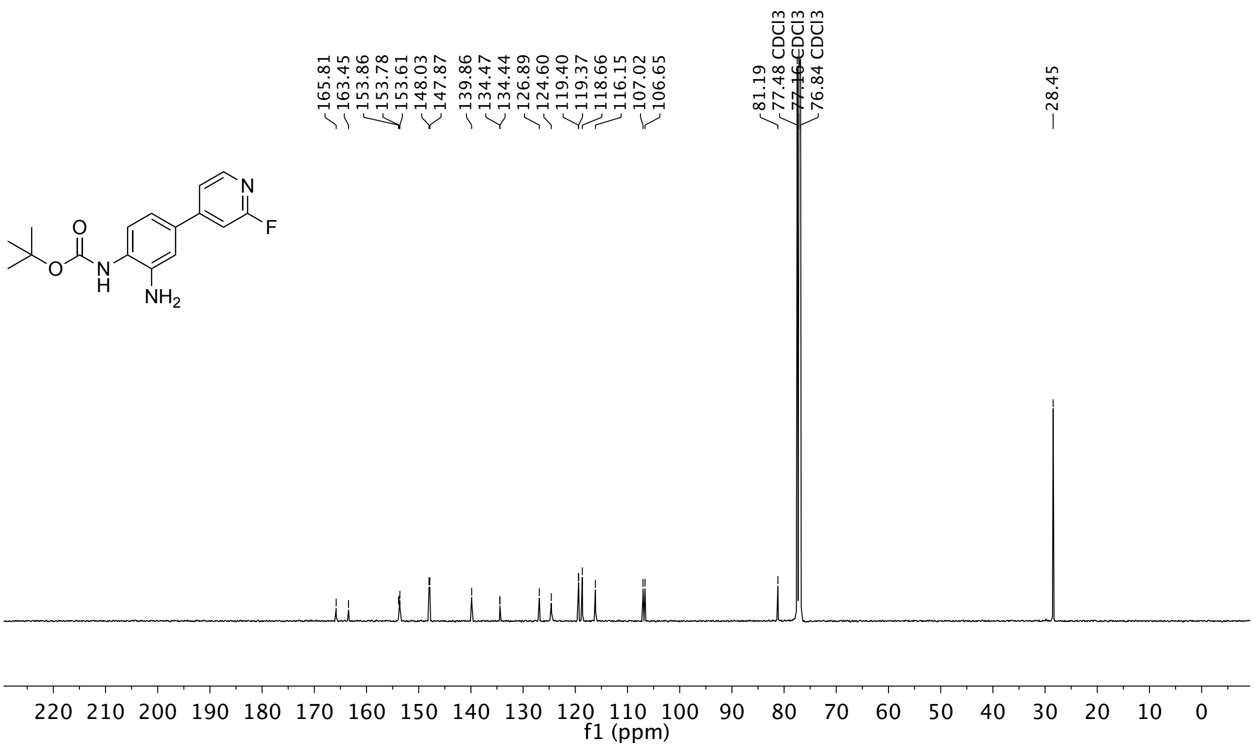

^{13}C NMR (101 MHz, Chloroform-*d*) of **4i**.

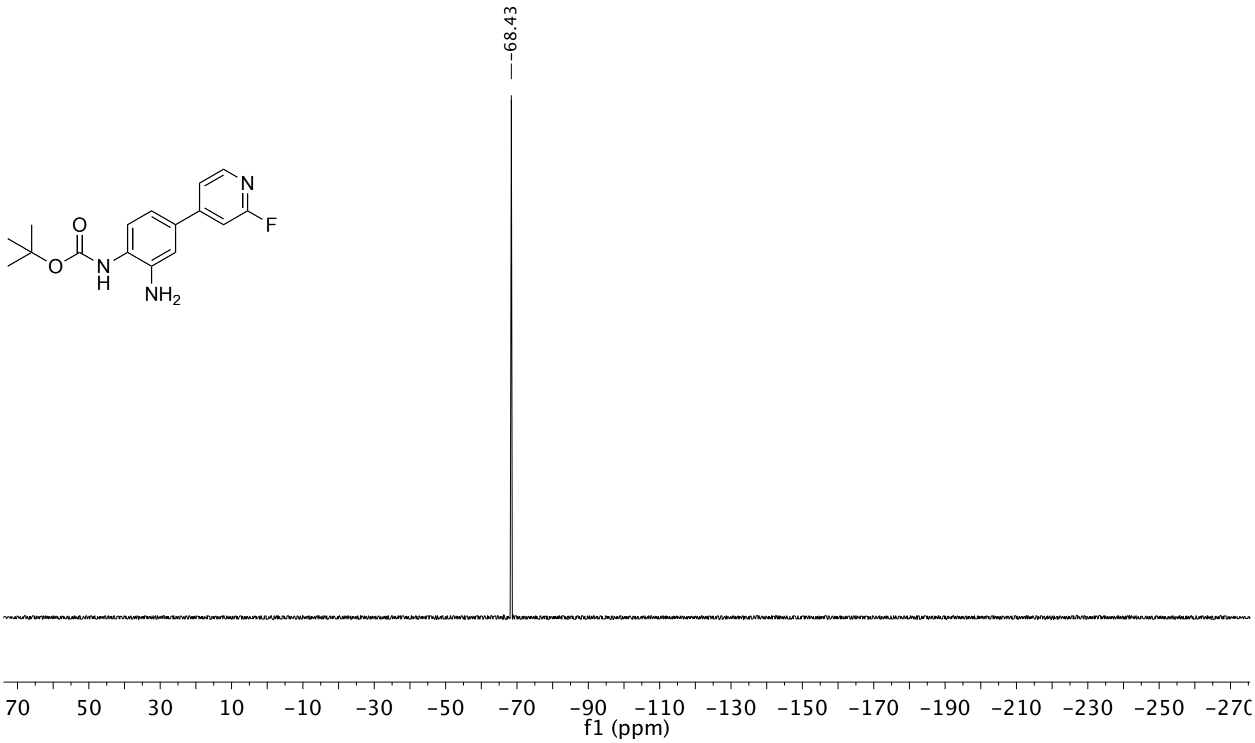

^{19}F NMR (377 MHz, Chloroform-*d*) of **4i**.

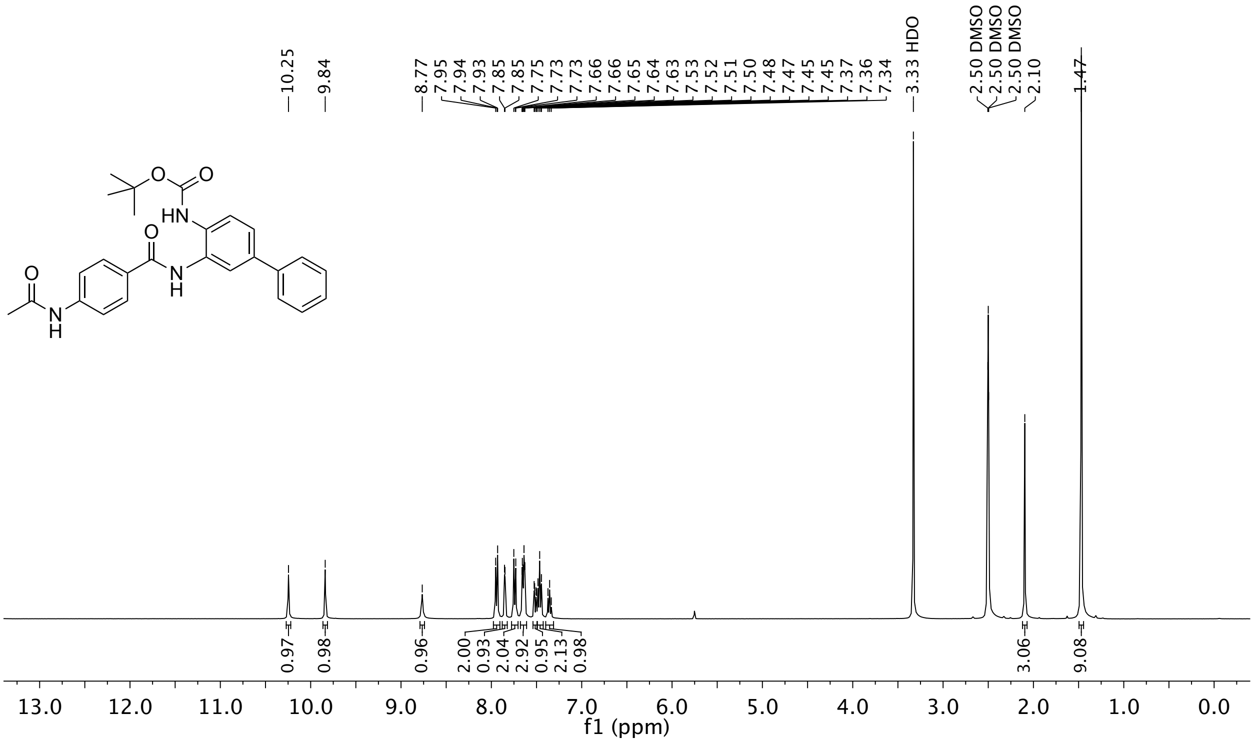

^{1}H NMR (400 MHz, DMSO-d_6) of **5a**.

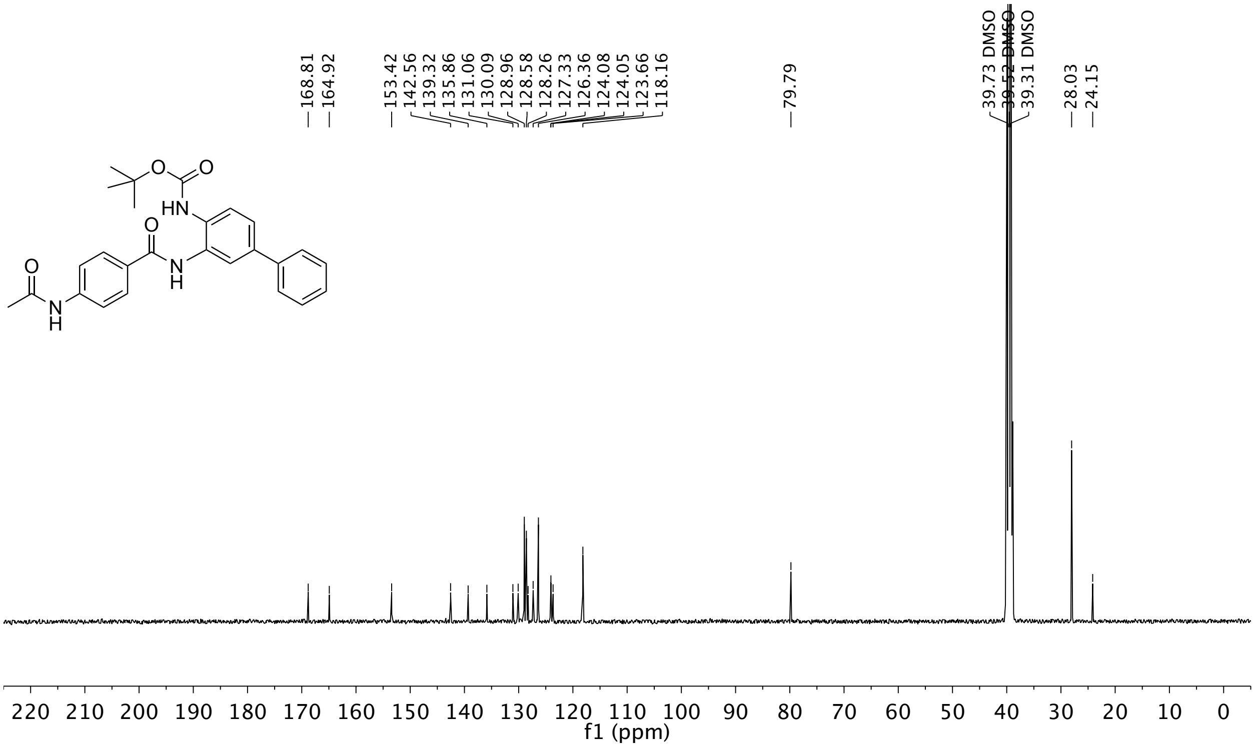

^{13}C NMR (101 MHz, DMSO-d_6) of **5a**.

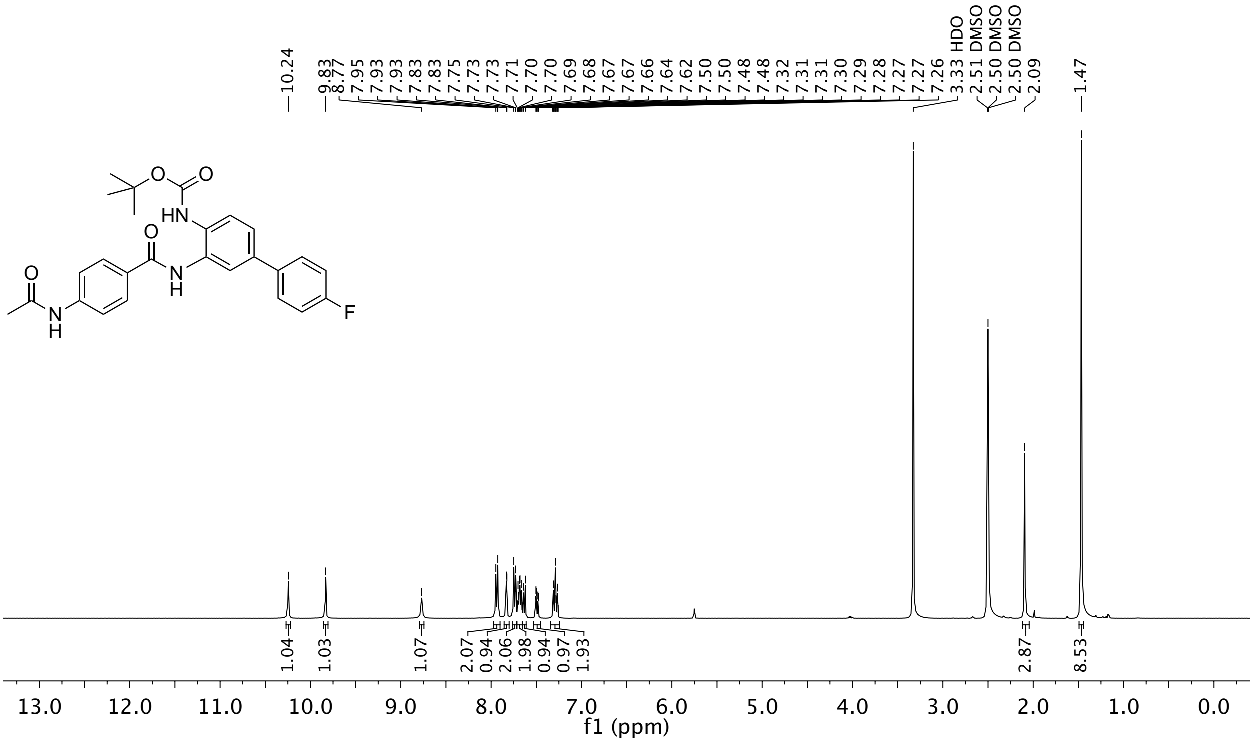

^{1}H NMR (400 MHz, DMSO-d_6) of **5b**.

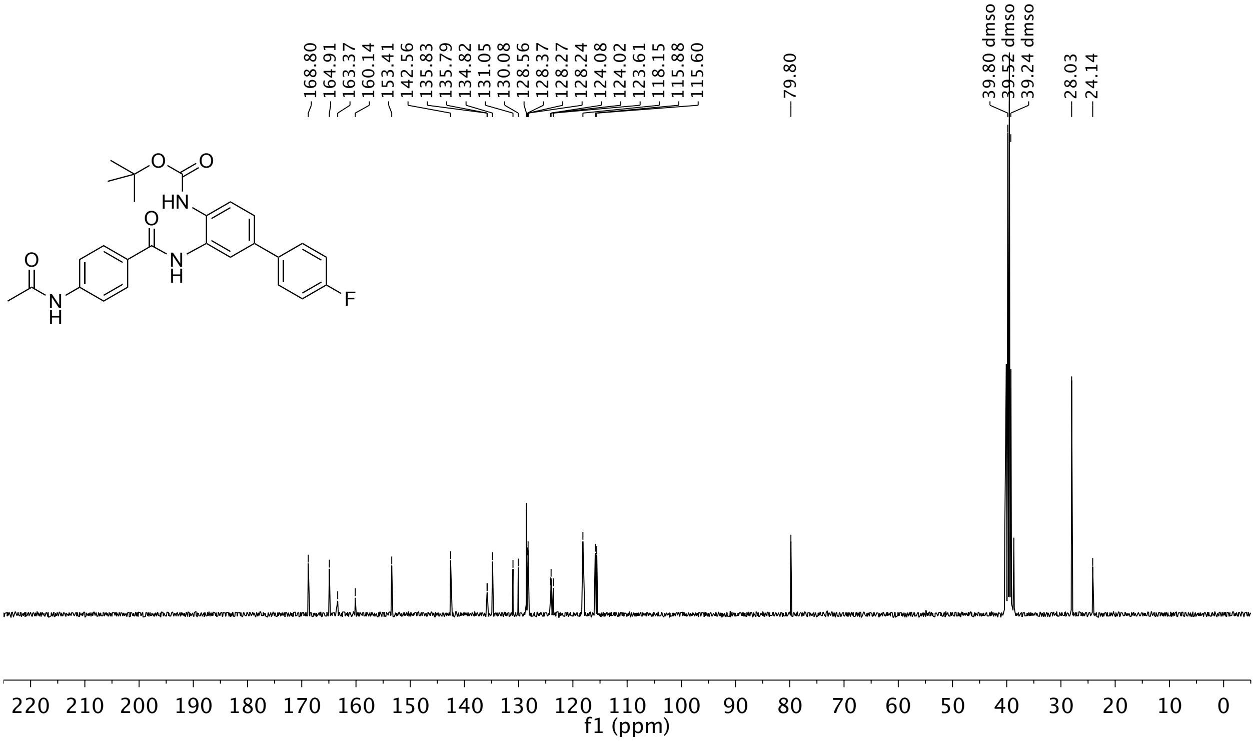

^{13}C NMR (75 MHz, DMSO-d_6) of **5b**.

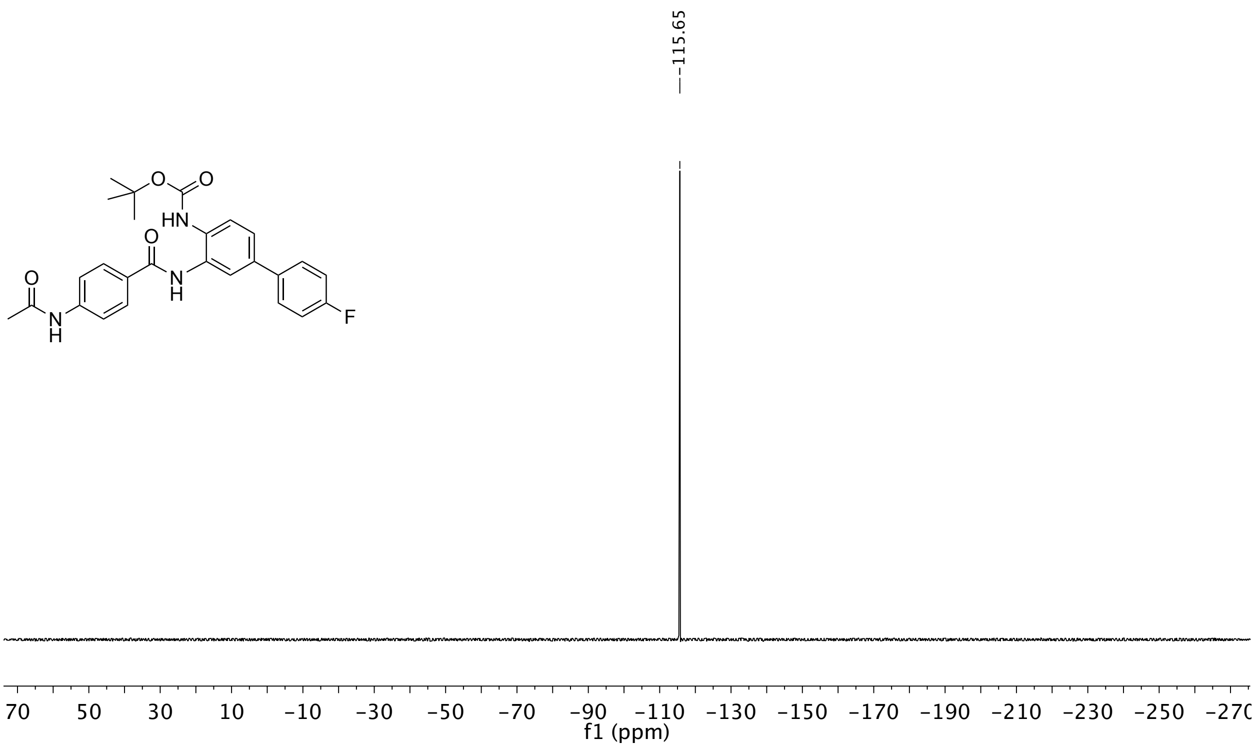

^{19}F NMR (377 MHz, DMSO-d_6) of **5b**.

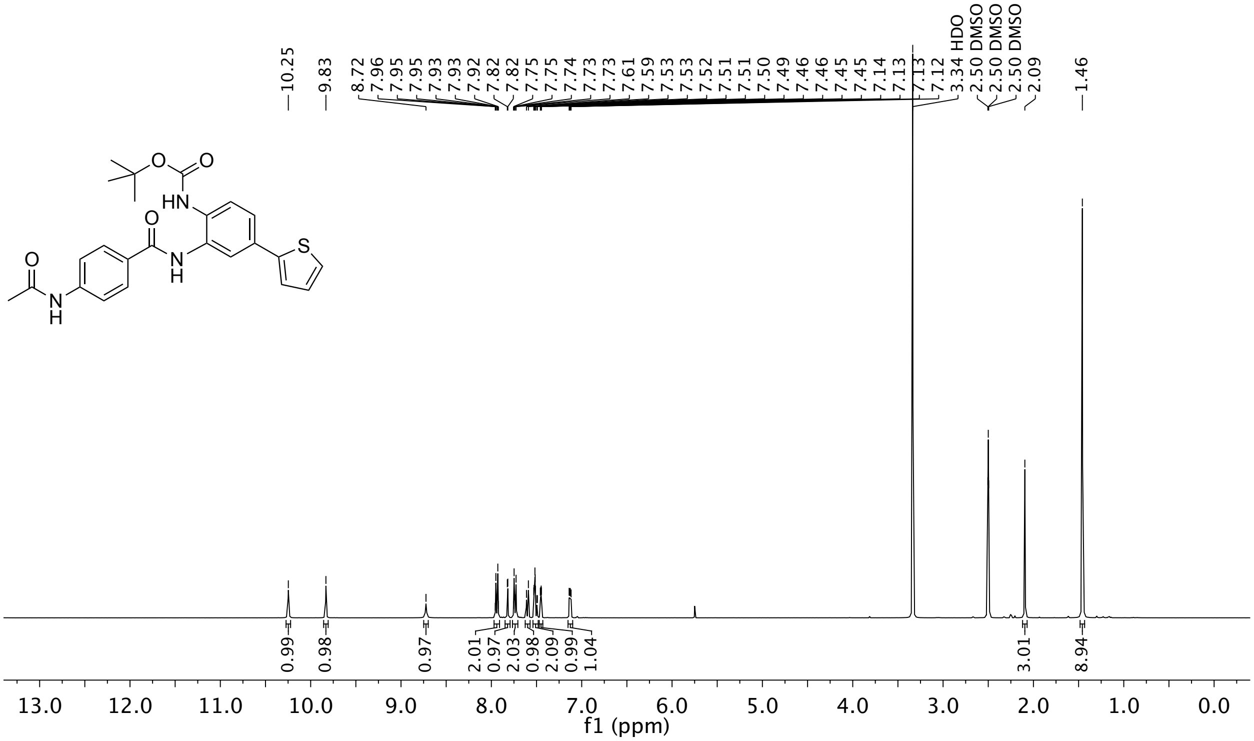

^{1}H NMR (400 MHz, DMSO-d_6) of **5c**.

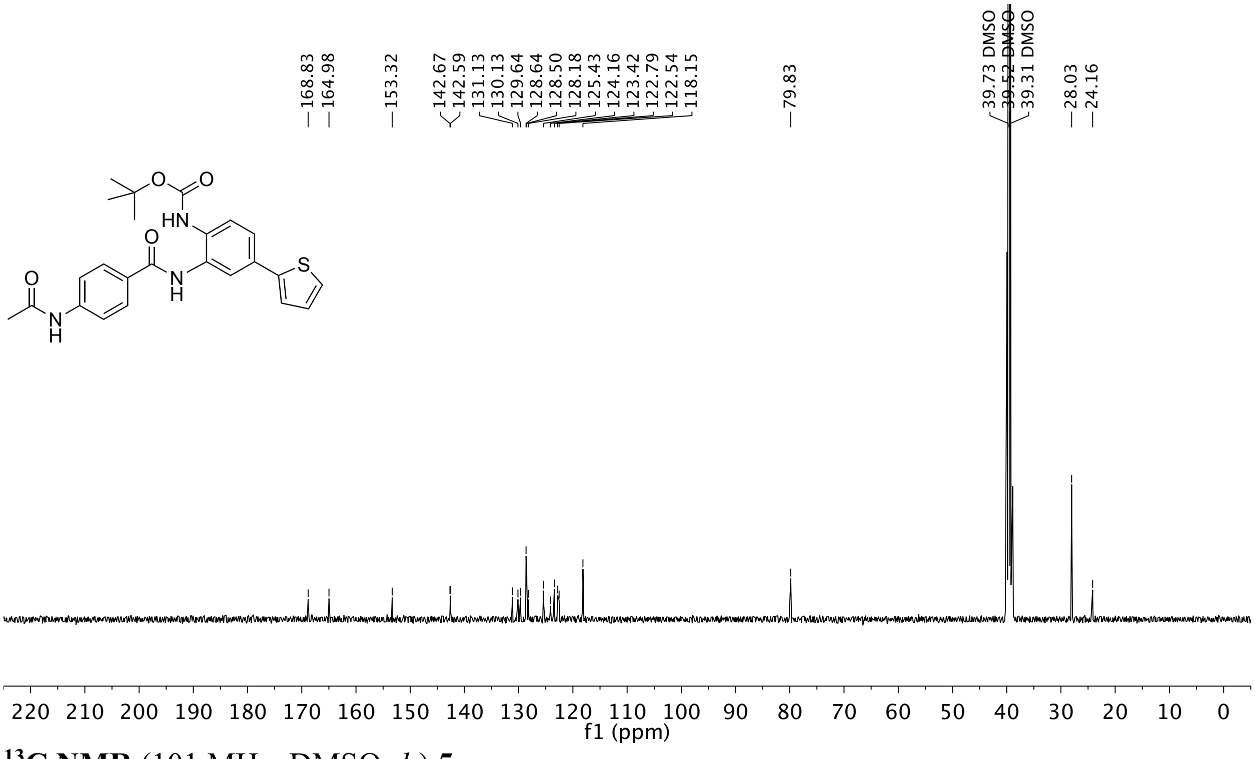

^{13}**C NMR** (101 MHz, DMSO-d_6) **5c**.

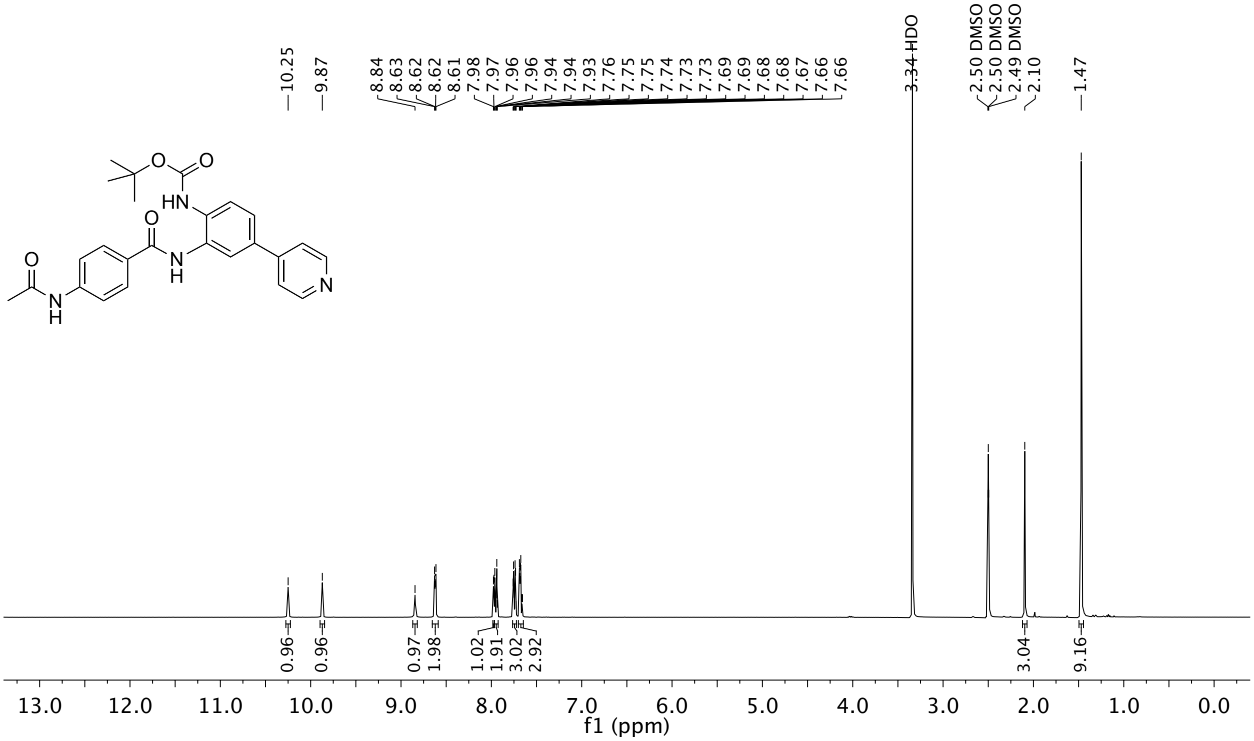

^{1}**H NMR** (400 MHz, DMSO-d_6) of **5d**.

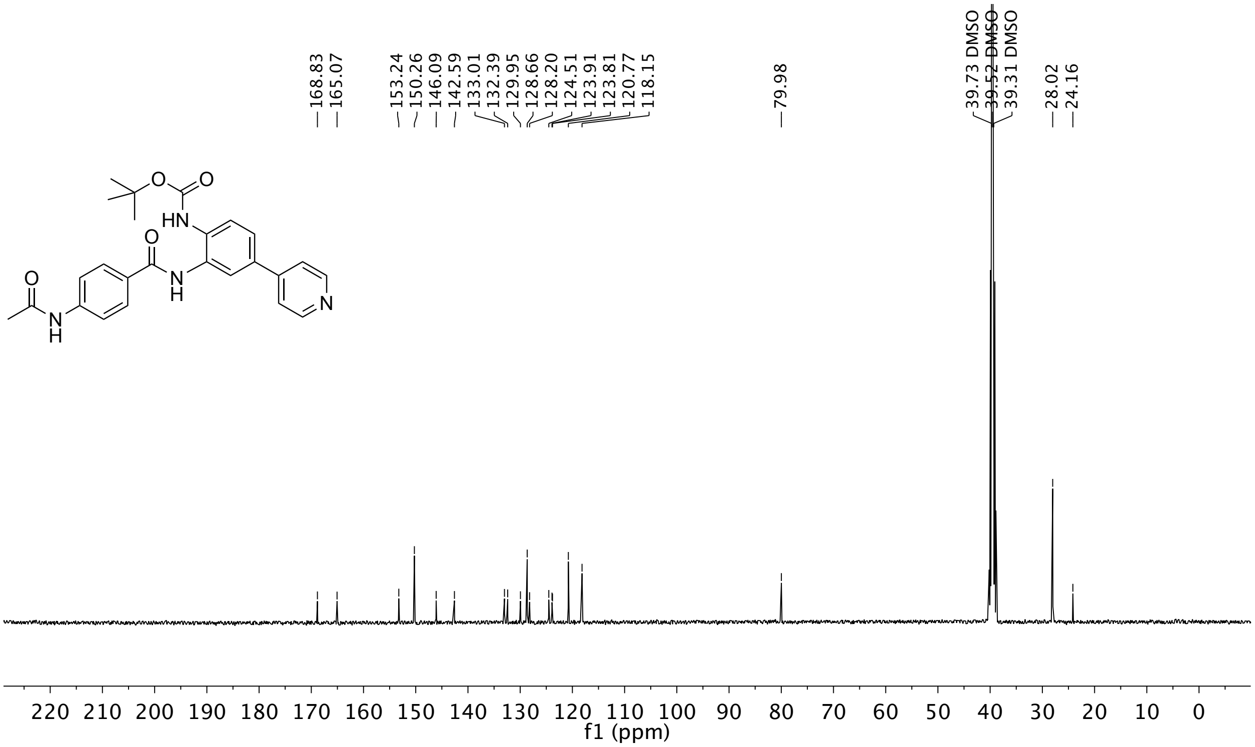

^{13}C NMR (101 MHz, DMSO-d_6) **5d**.

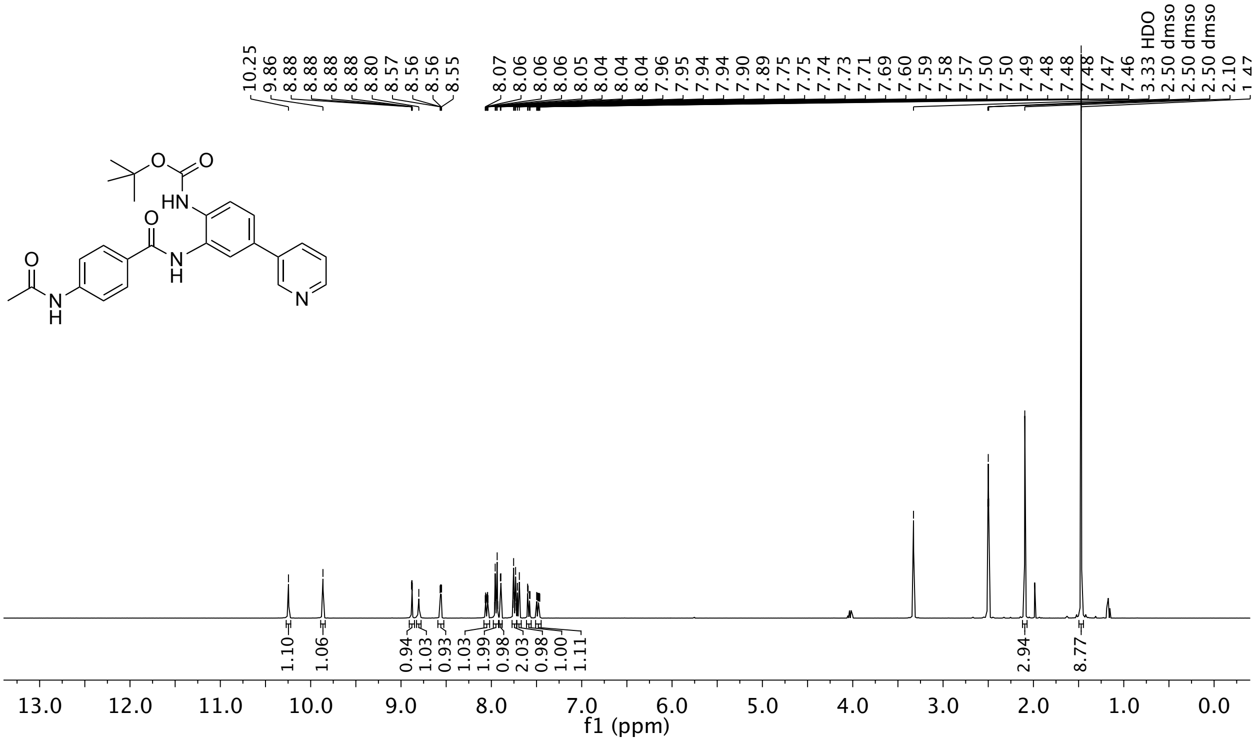

^{1}H NMR (400 MHz, DMSO-d_6) of **5e**.

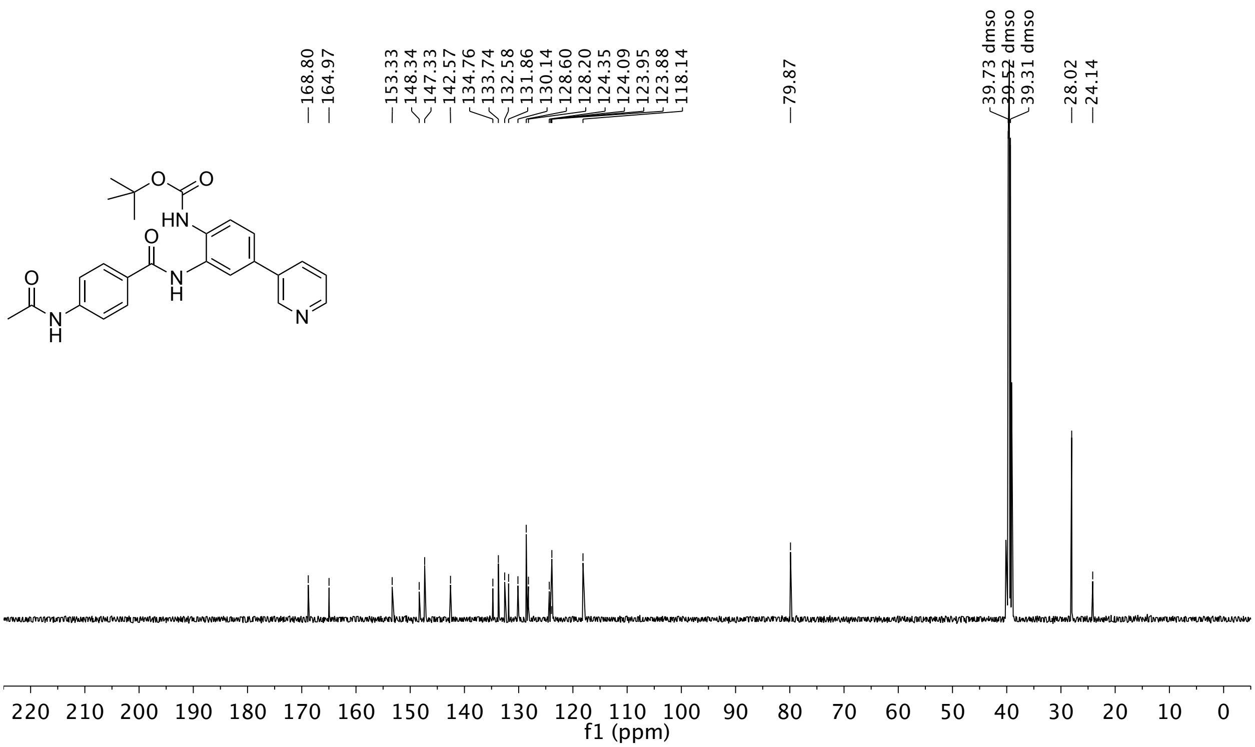

^{13}C NMR (101 MHz, DMSO-d_6) **5e**.

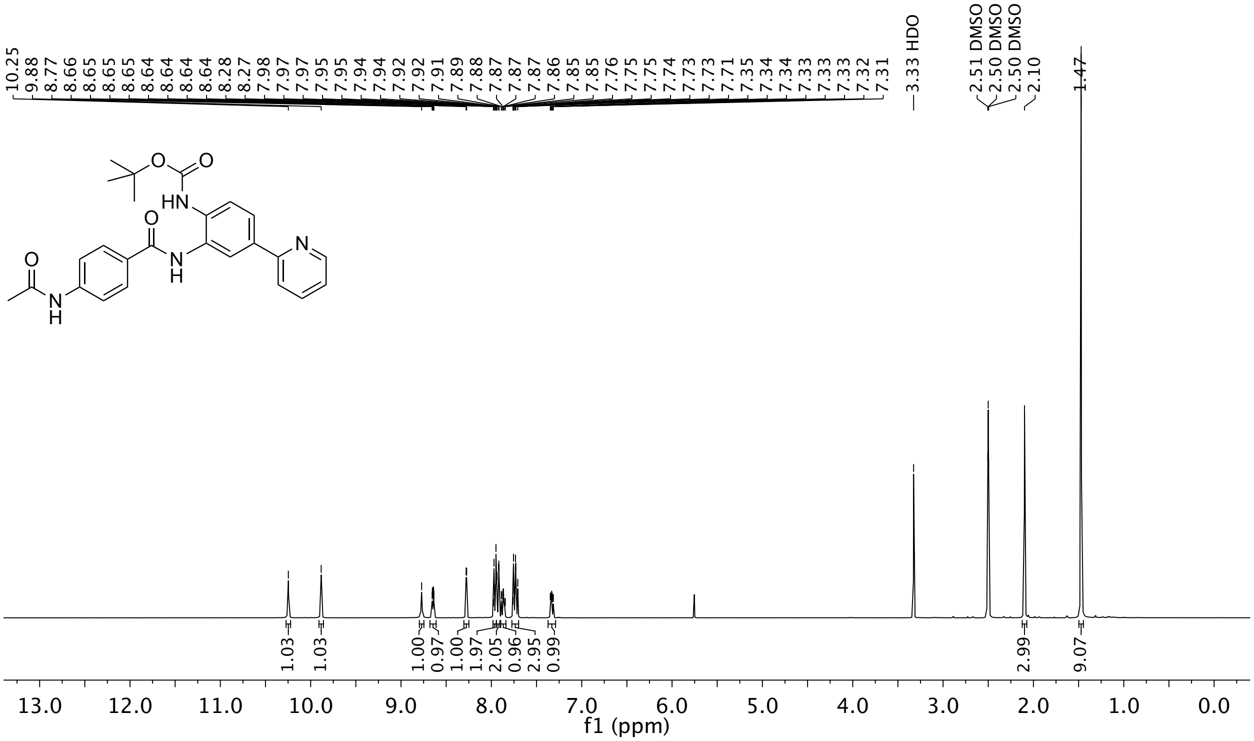

^{1}H NMR (400 MHz, DMSO-d_6) of **5f**.

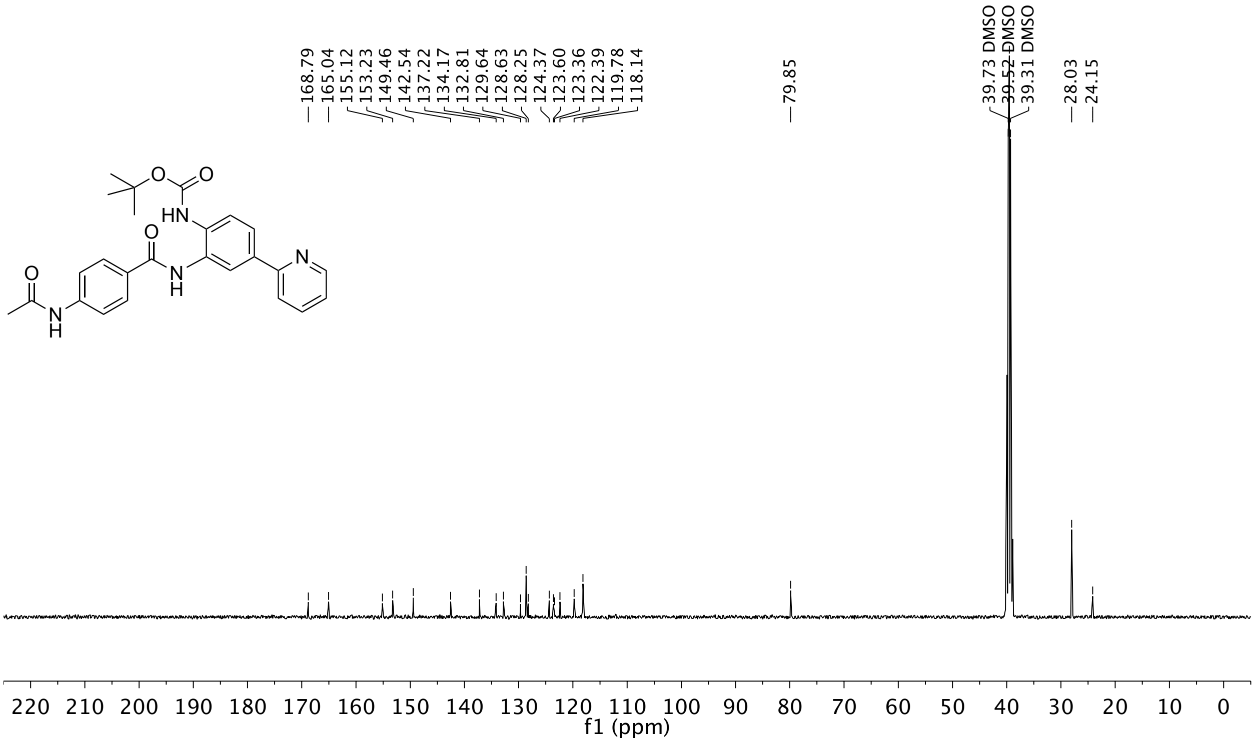

^{13}C NMR (101 MHz, DMSO-d_6) **5f**.

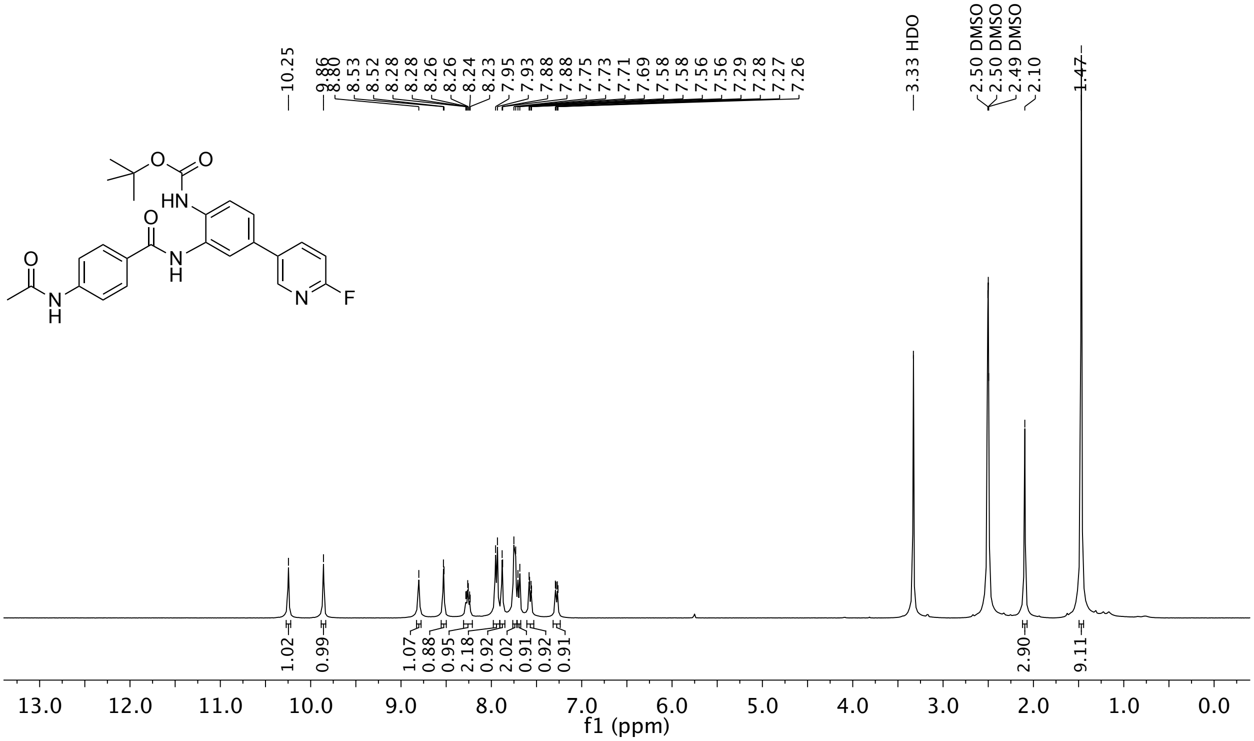

^{1}H NMR (400 MHz, DMSO-d_6) of **5g**.

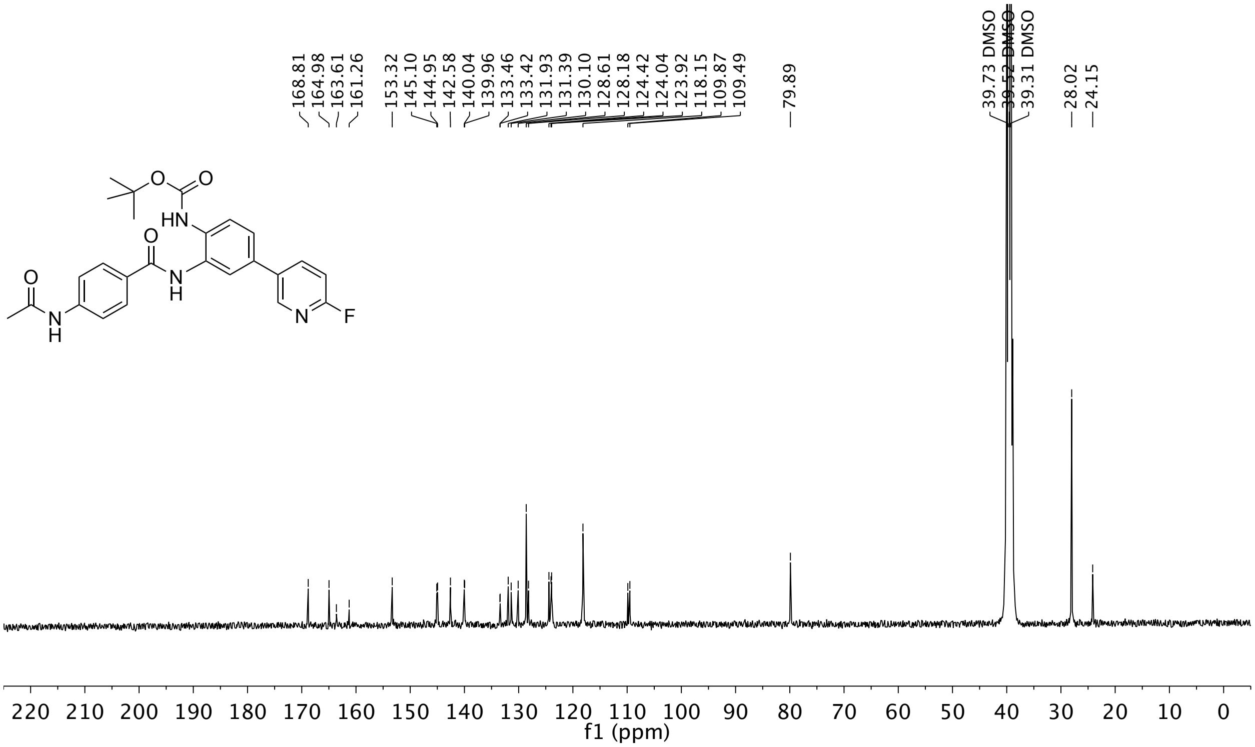

^{13}C NMR (101 MHz, DMSO-d_6) of **5g**.

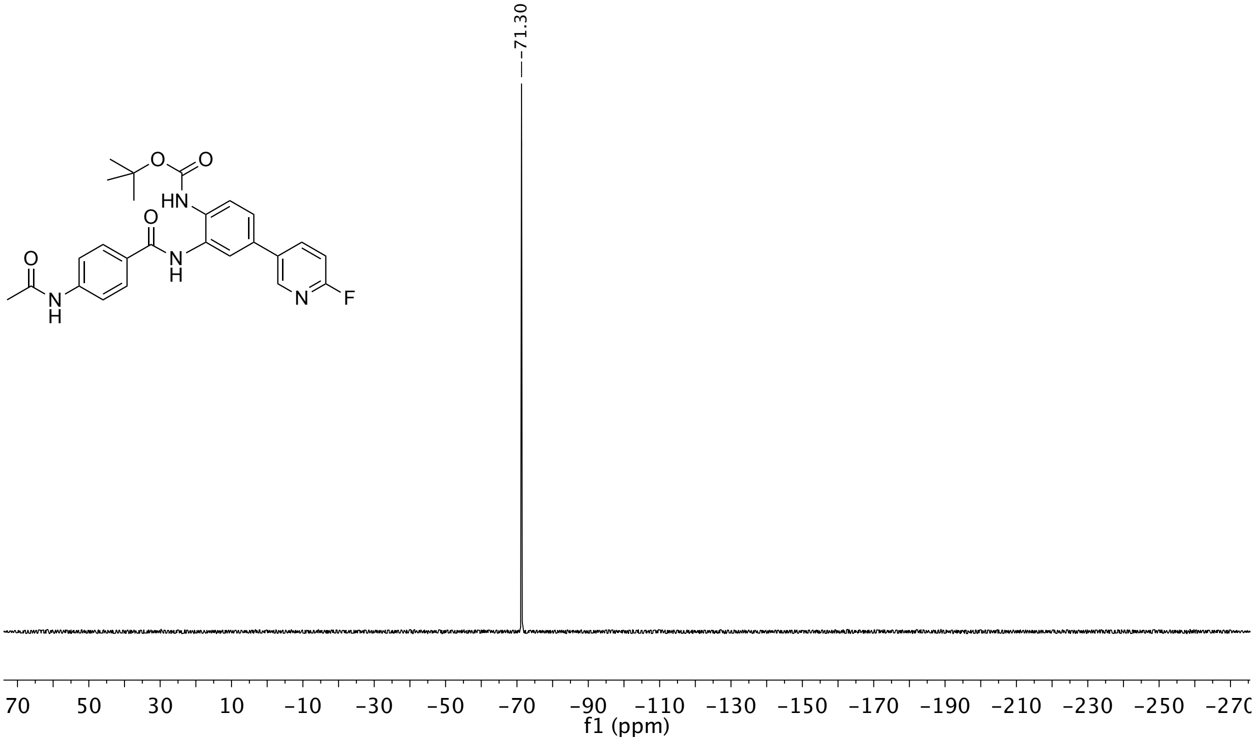

^{19}F NMR (377 MHz, DMSO-d_6) of **5g**.

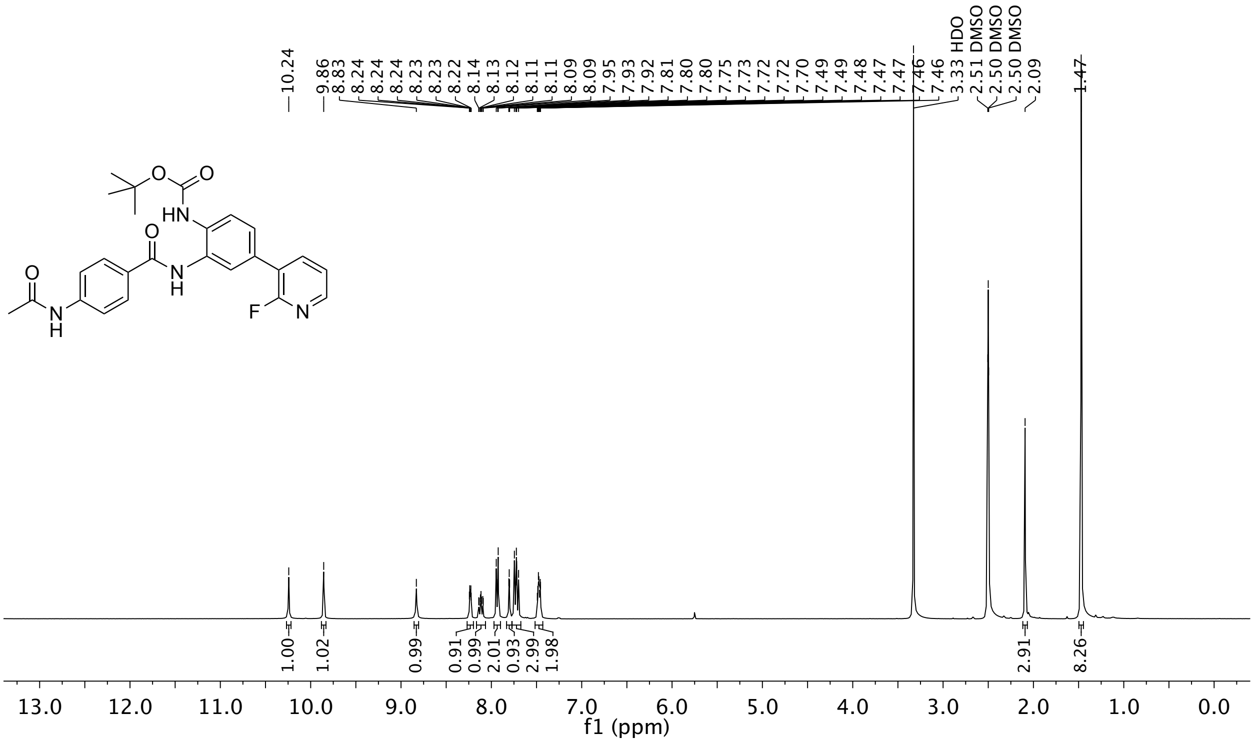

¹H NMR (400 MHz, DMSO-d_6) of **5h**.

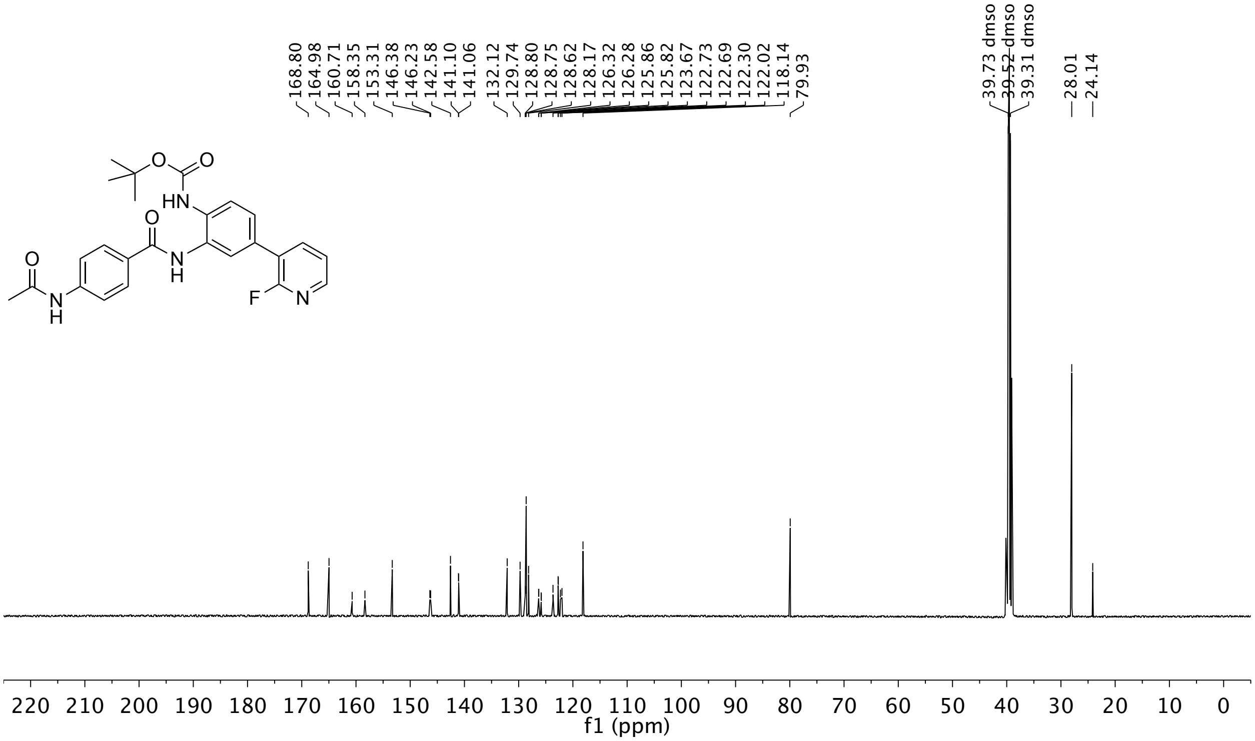

¹³C NMR (101 MHz, DMSO-d_6) **5h**.

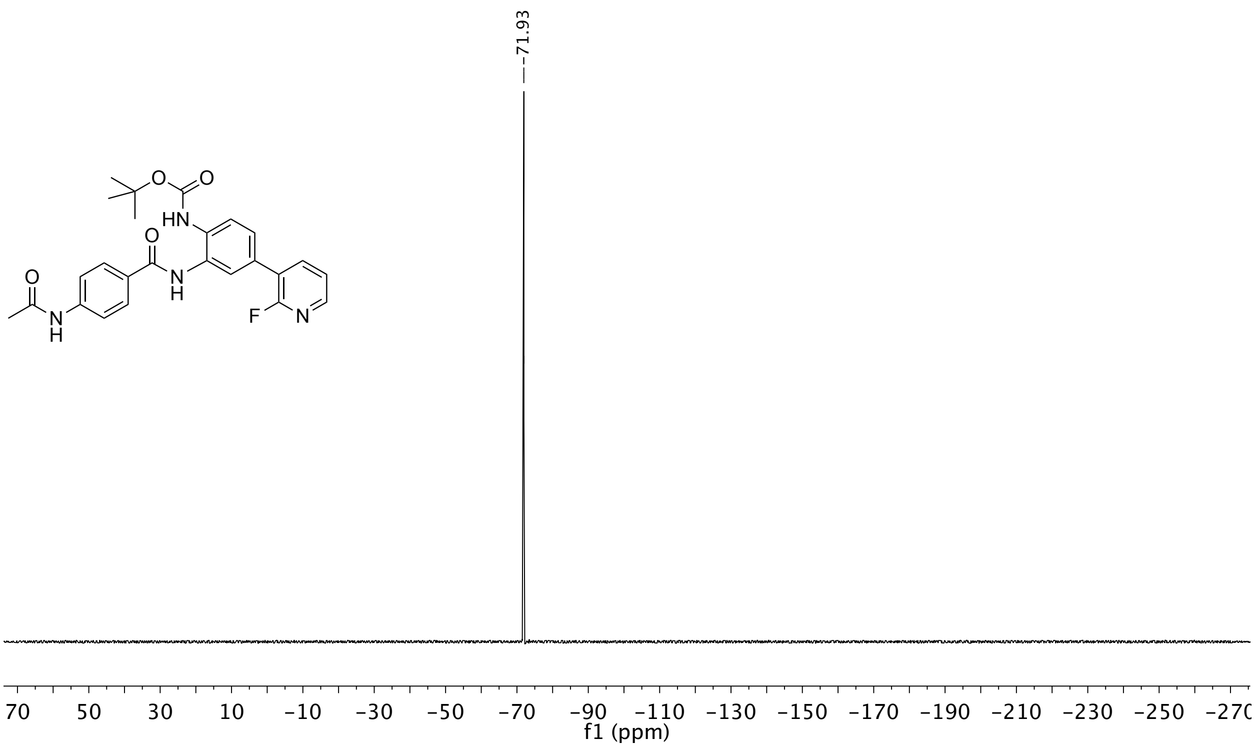

^{19}F NMR (377 MHz, DMSO-d_6) **5h**.

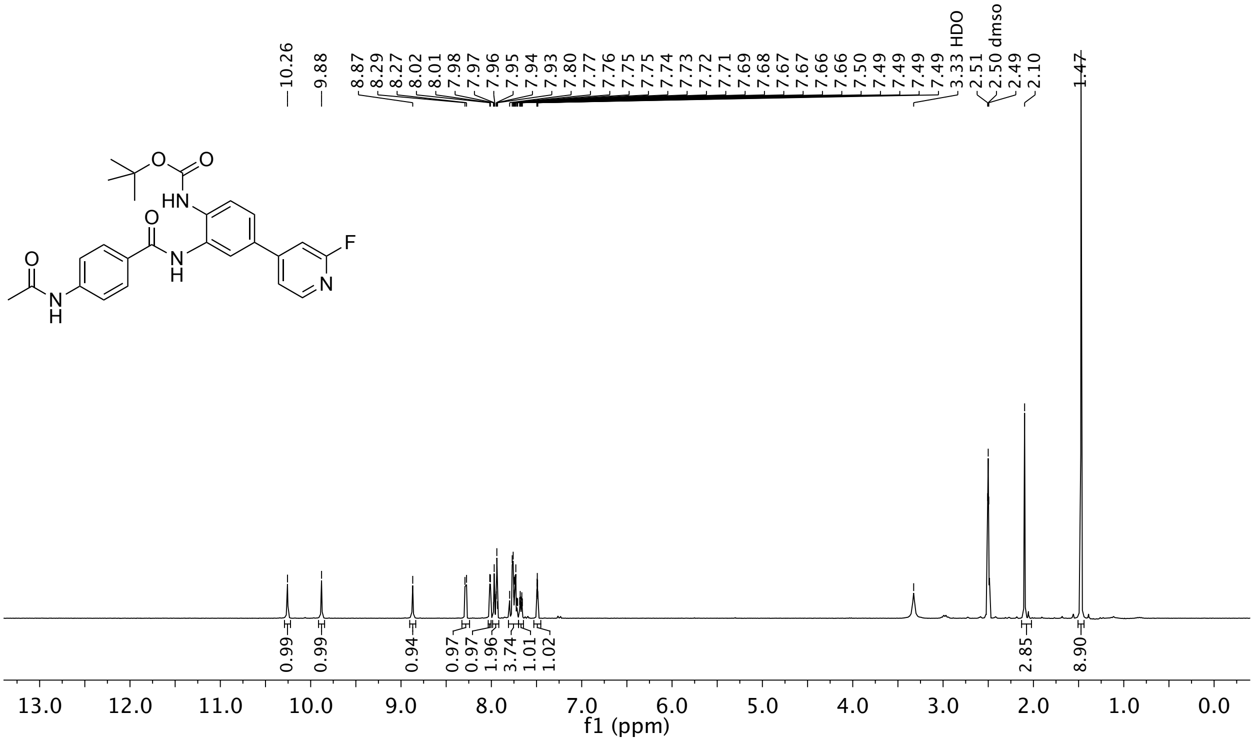

^{1}H NMR (300 MHz, DMSO-d_6) of **5i**.

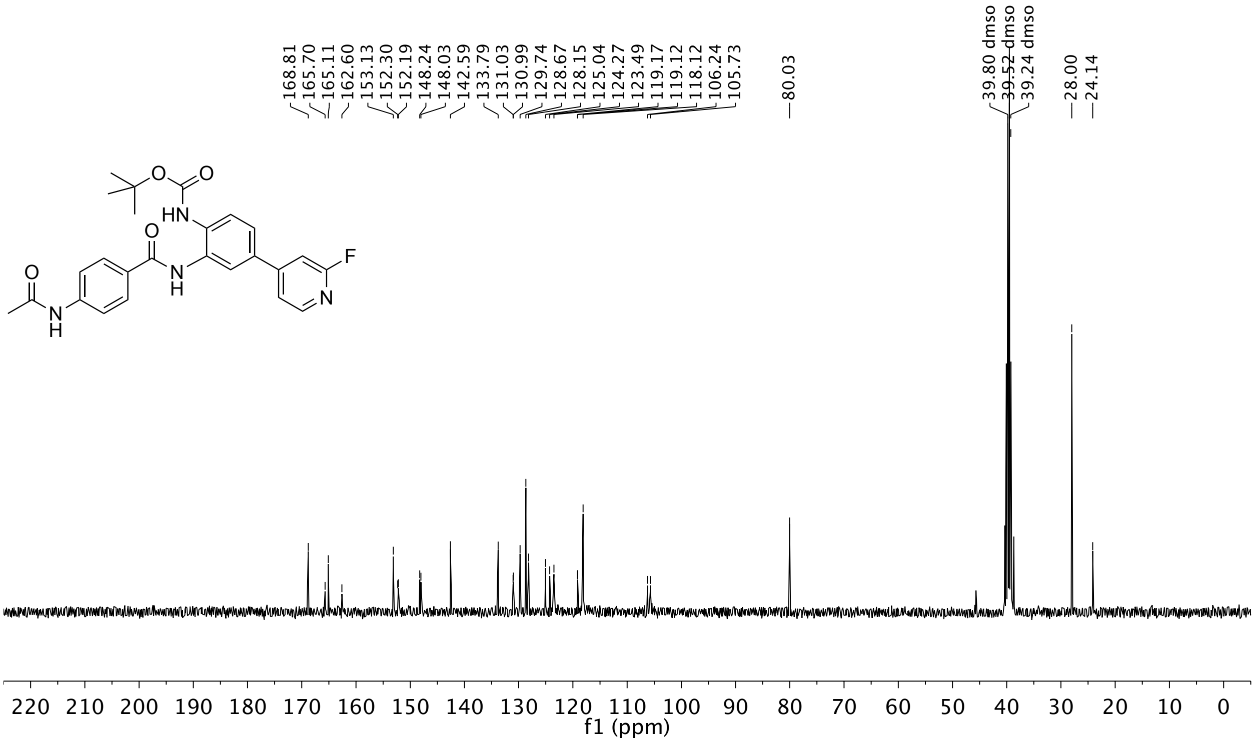

^{13}C NMR (75 MHz, DMSO-d_6) of **5i**.

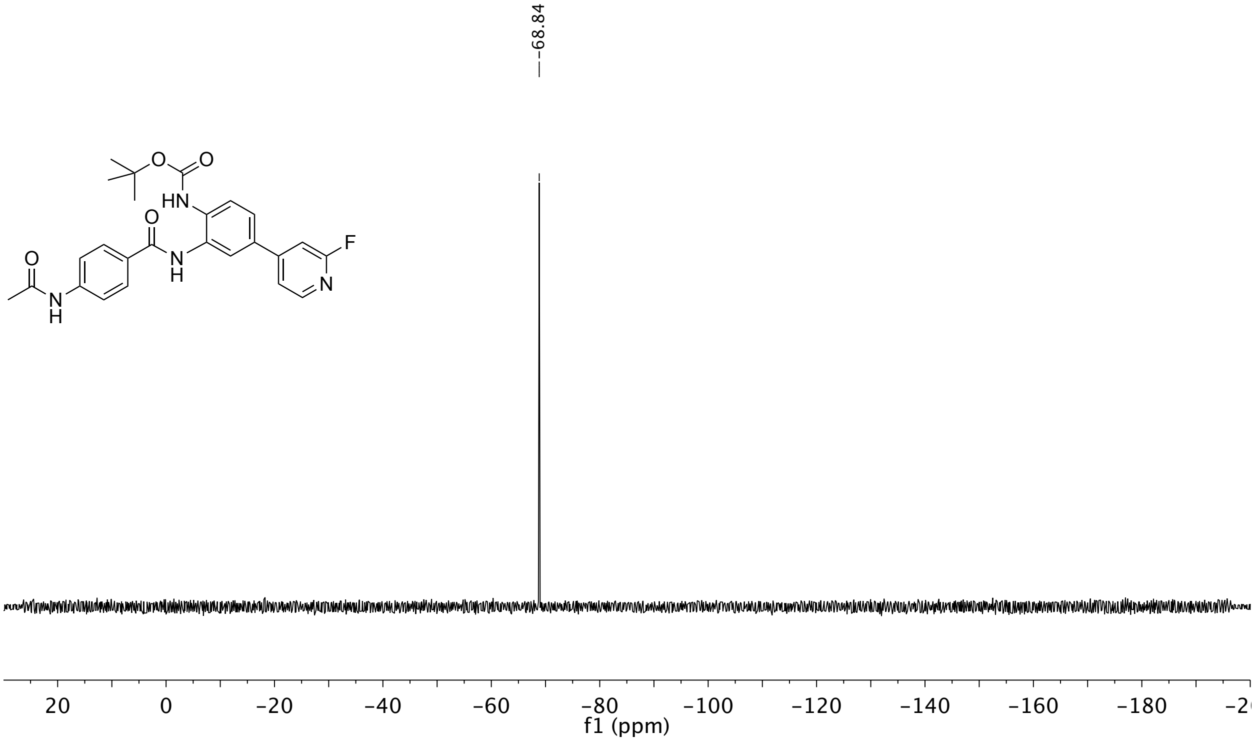

^{19}F NMR (282 MHz, DMSO-d_6) of **5i**.

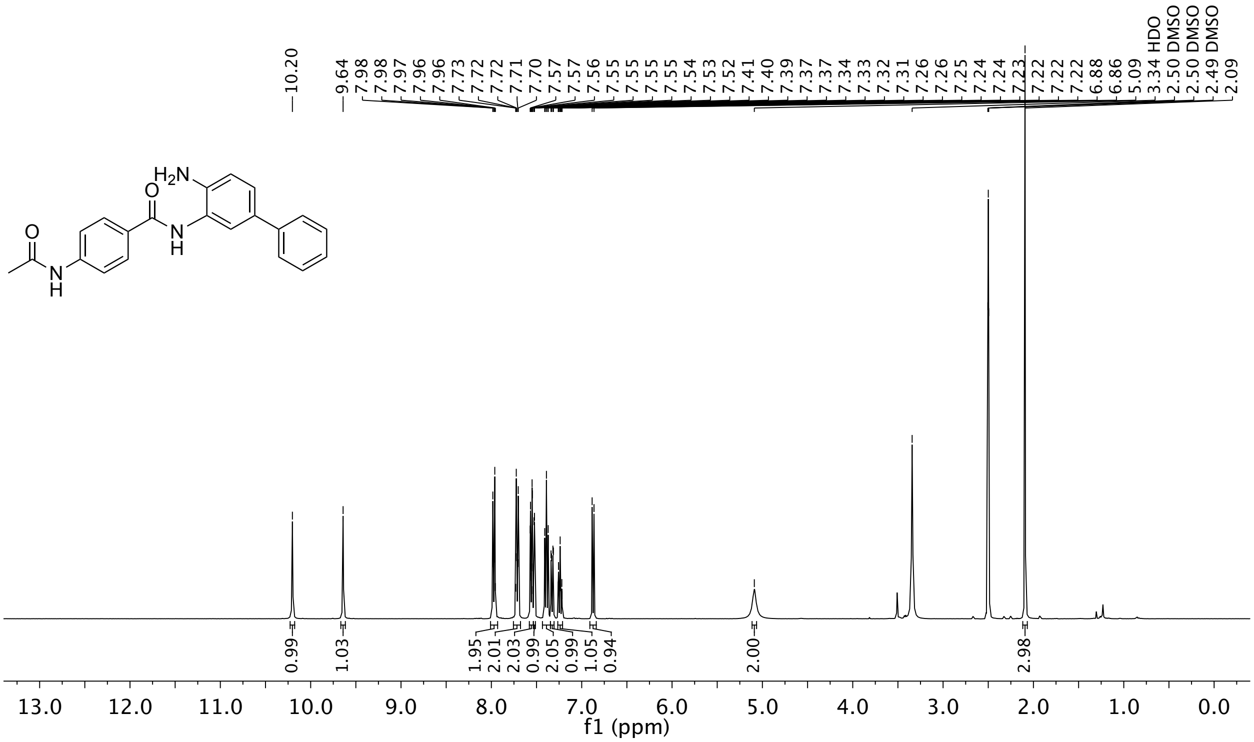

^{1}H NMR (400 MHz, DMSO-d_6) of **6a**.

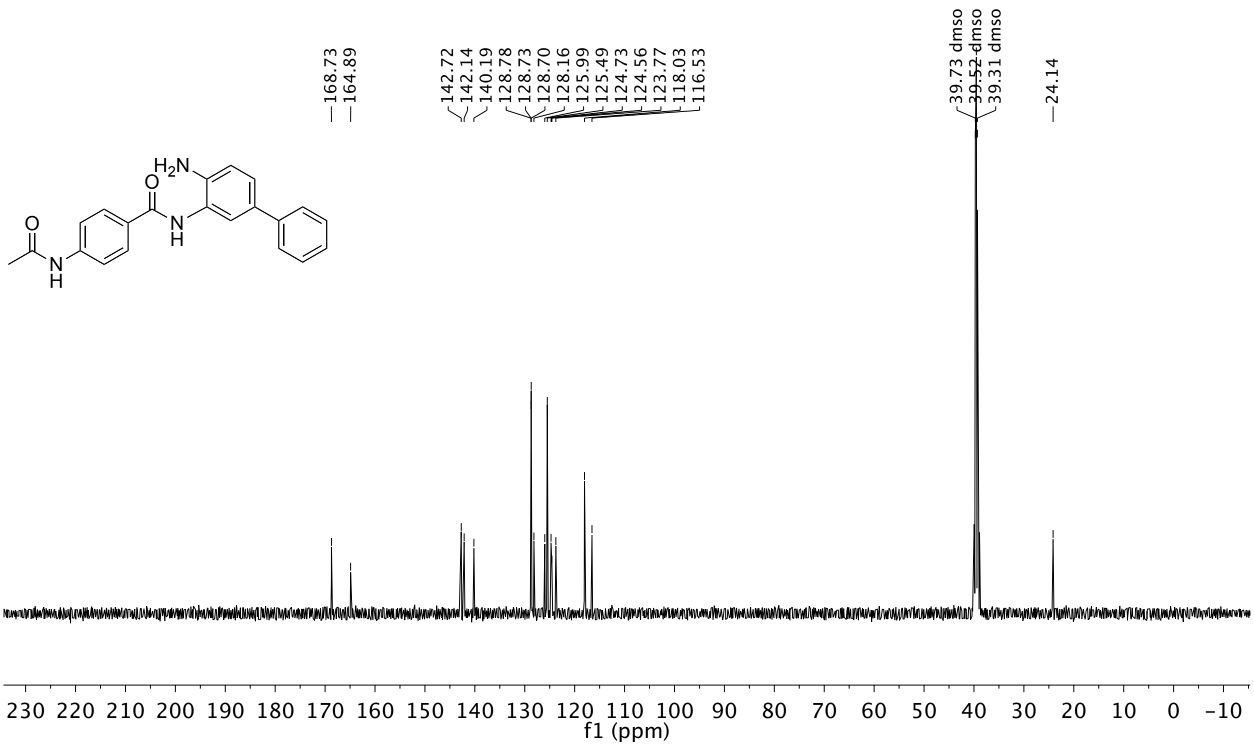

^{13}C NMR (101 MHz, DMSO-d_6) of **6a**.

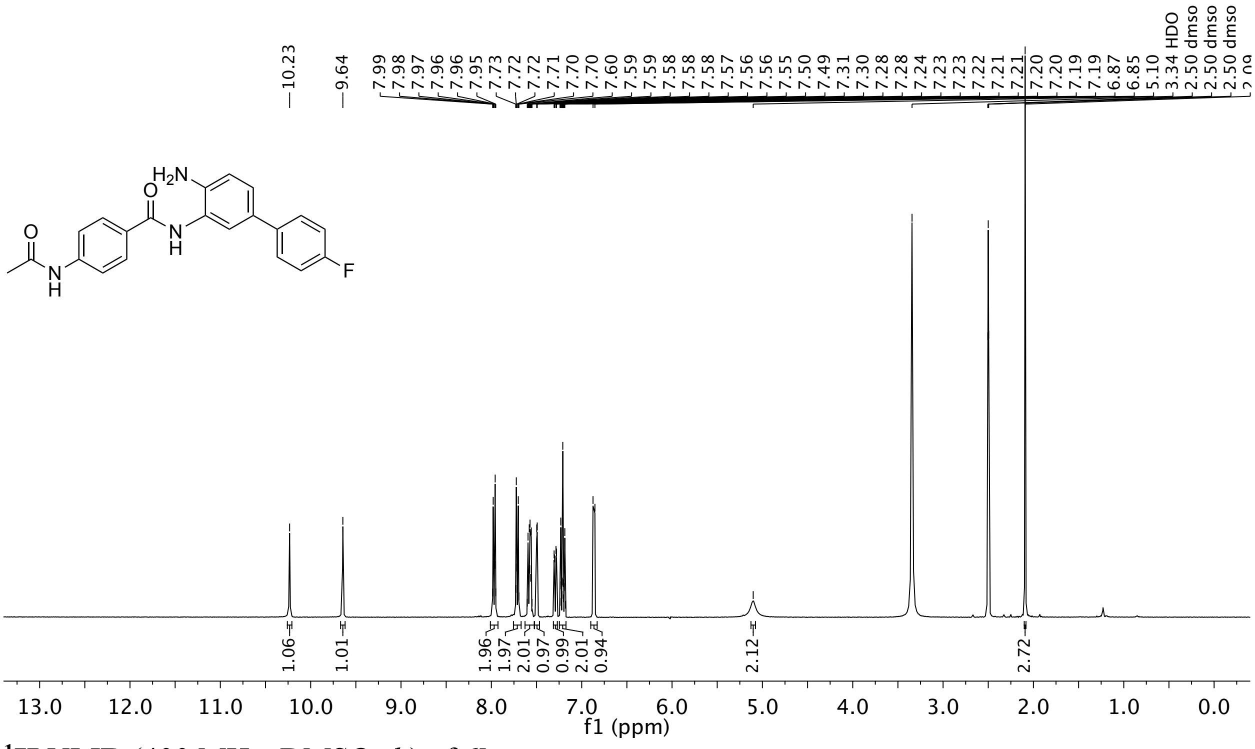

^{1}H NMR (400 MHz, DMSO-d_6) of **6b**.

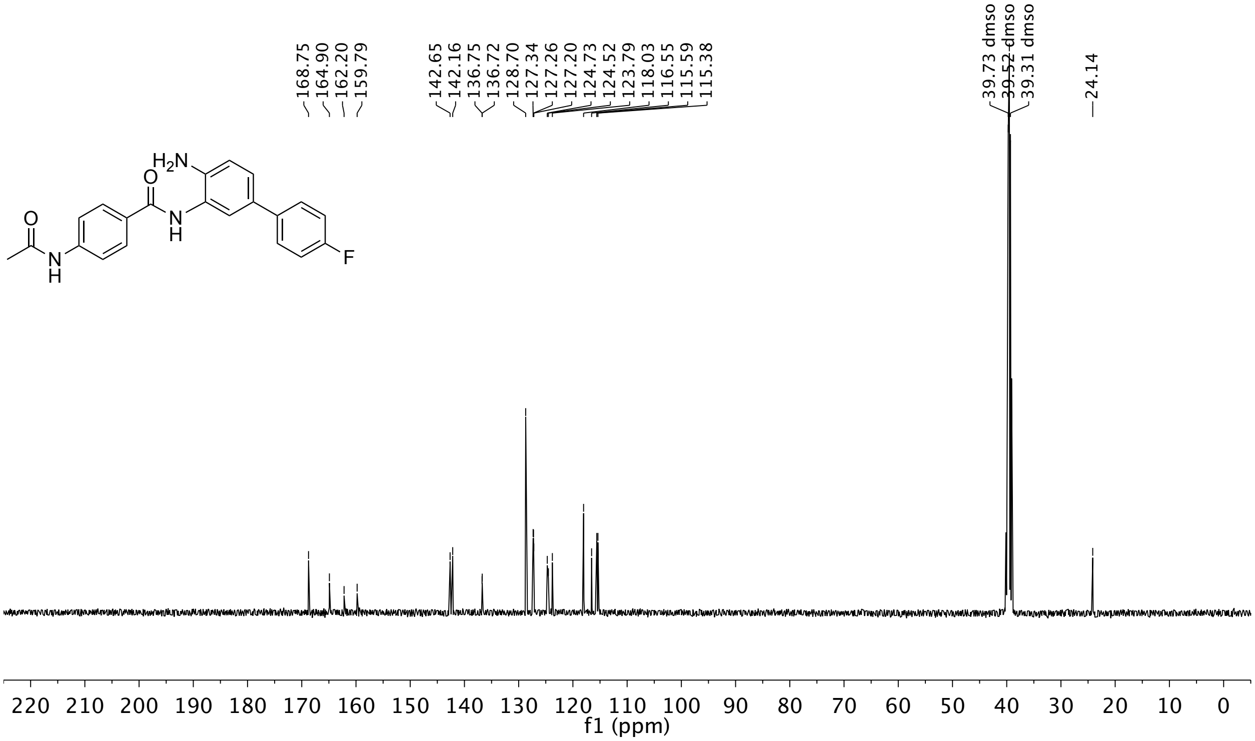

^{13}C NMR (101 MHz, DMSO-d_6) of **6b**.

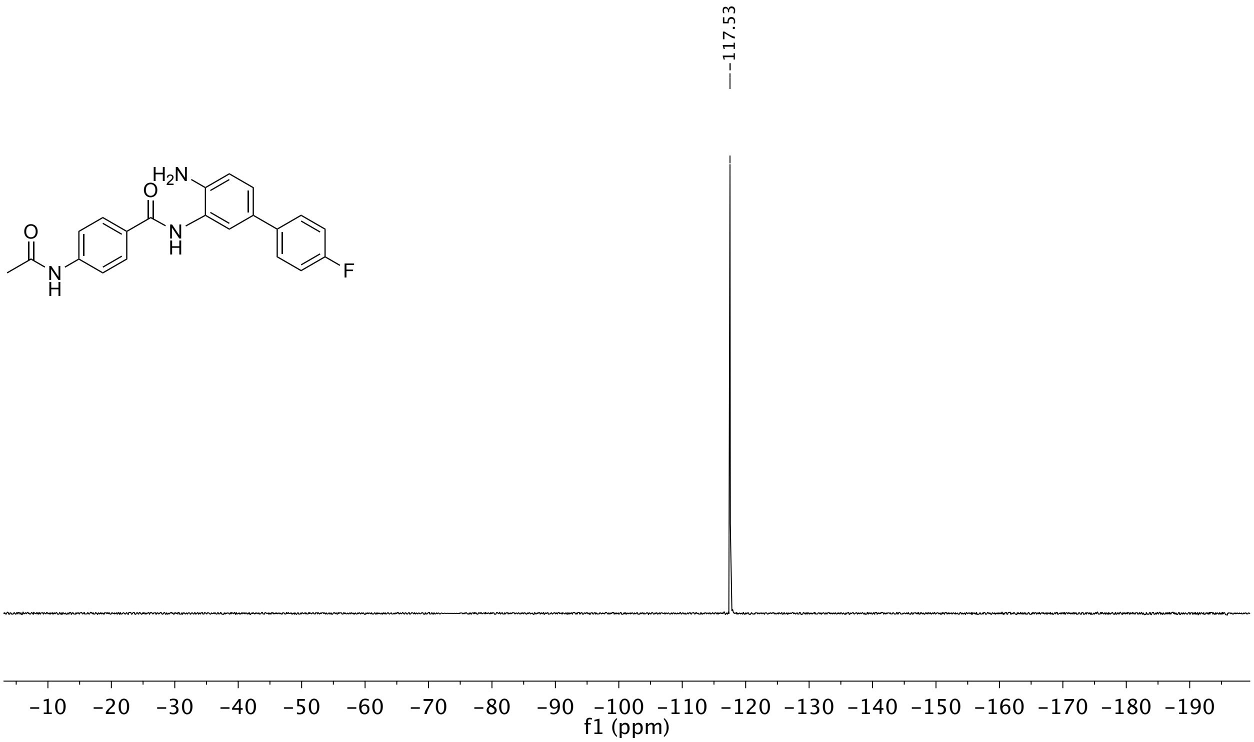

^{19}F NMR (376 MHz, DMSO-d_6) of **6b**.

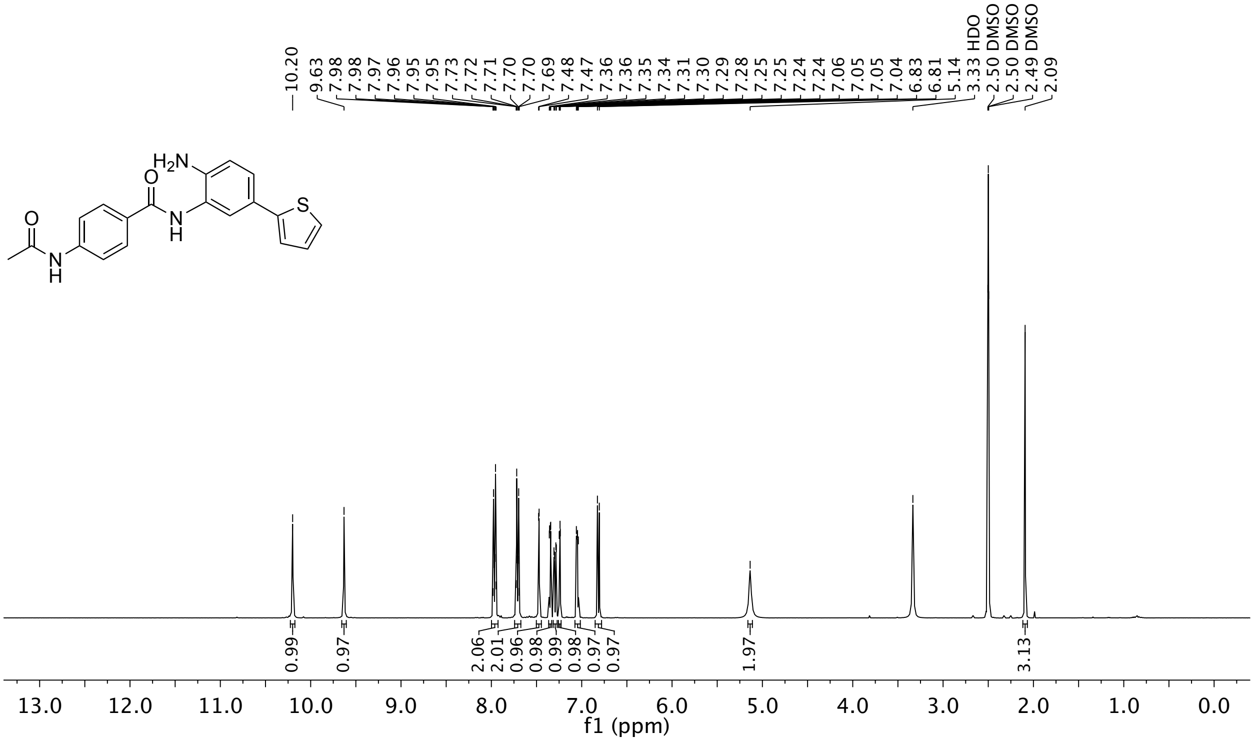

^{1}H NMR (400 MHz, DMSO-d_6) of **6c**.

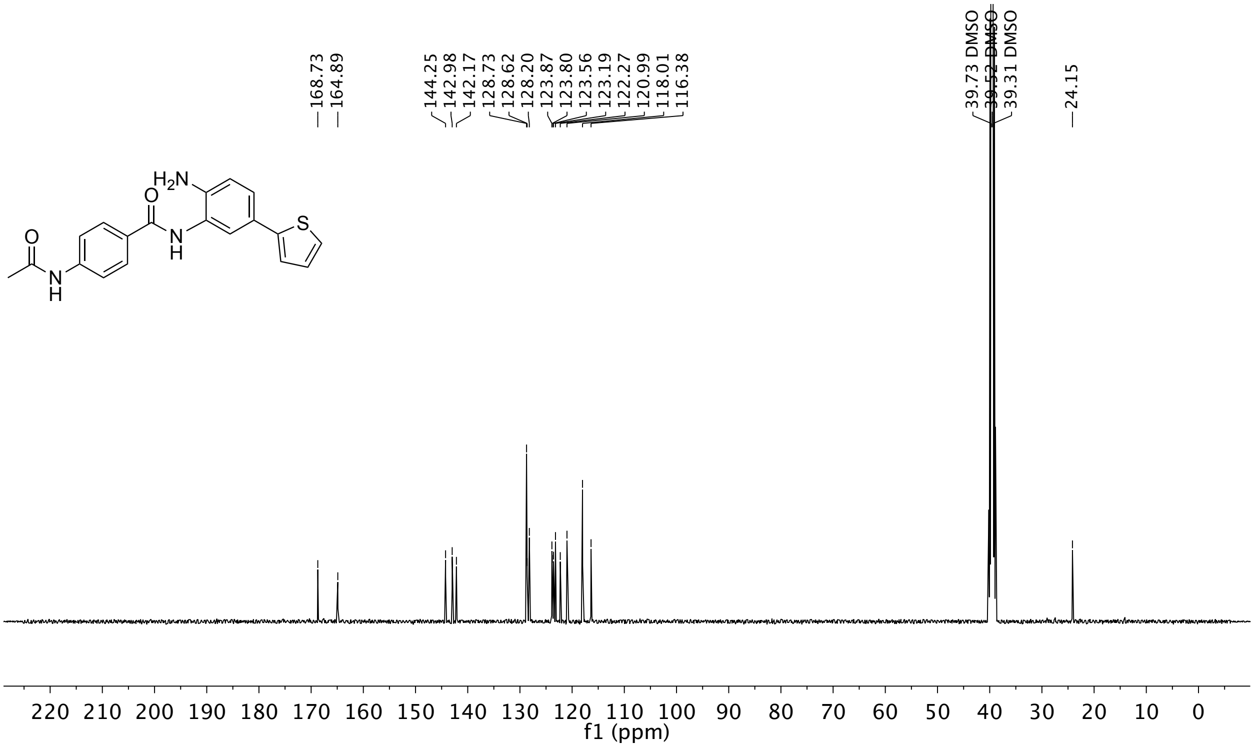

^{13}C NMR (101 MHz, DMSO-d_6) of **6c**.

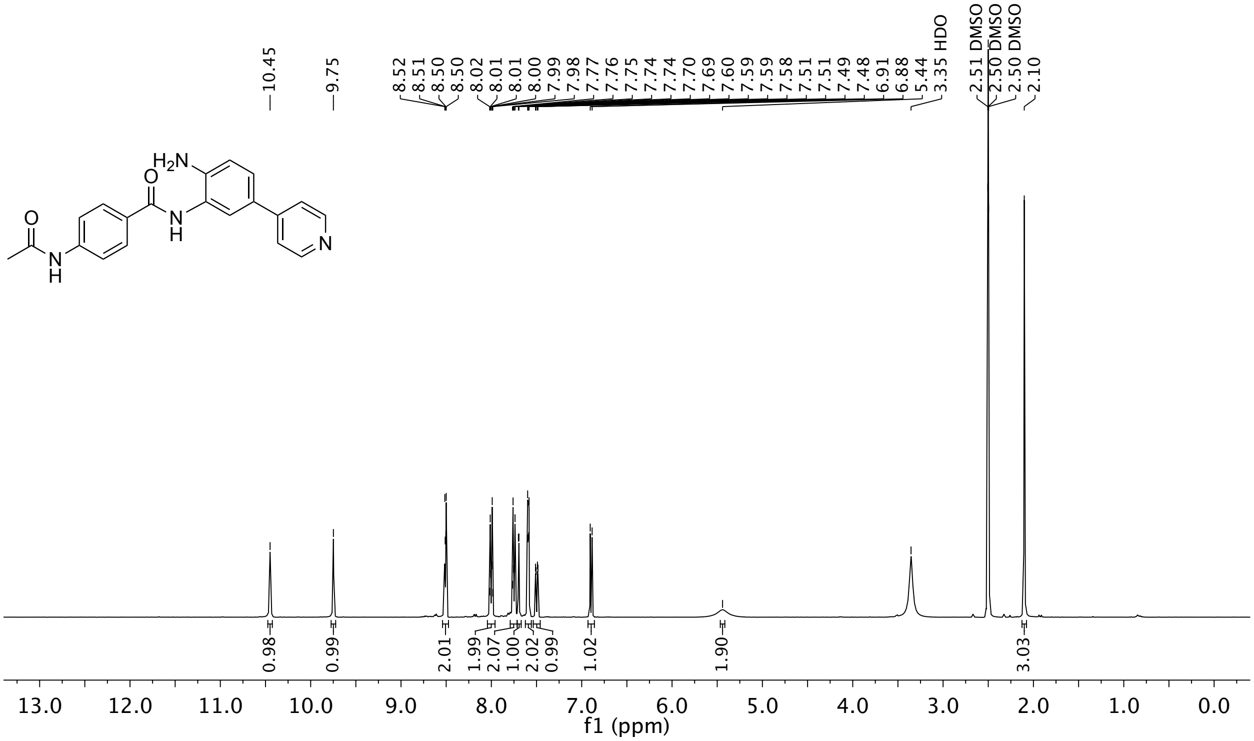

^{1}H NMR (400 MHz, DMSO-d_6) of **6d**.

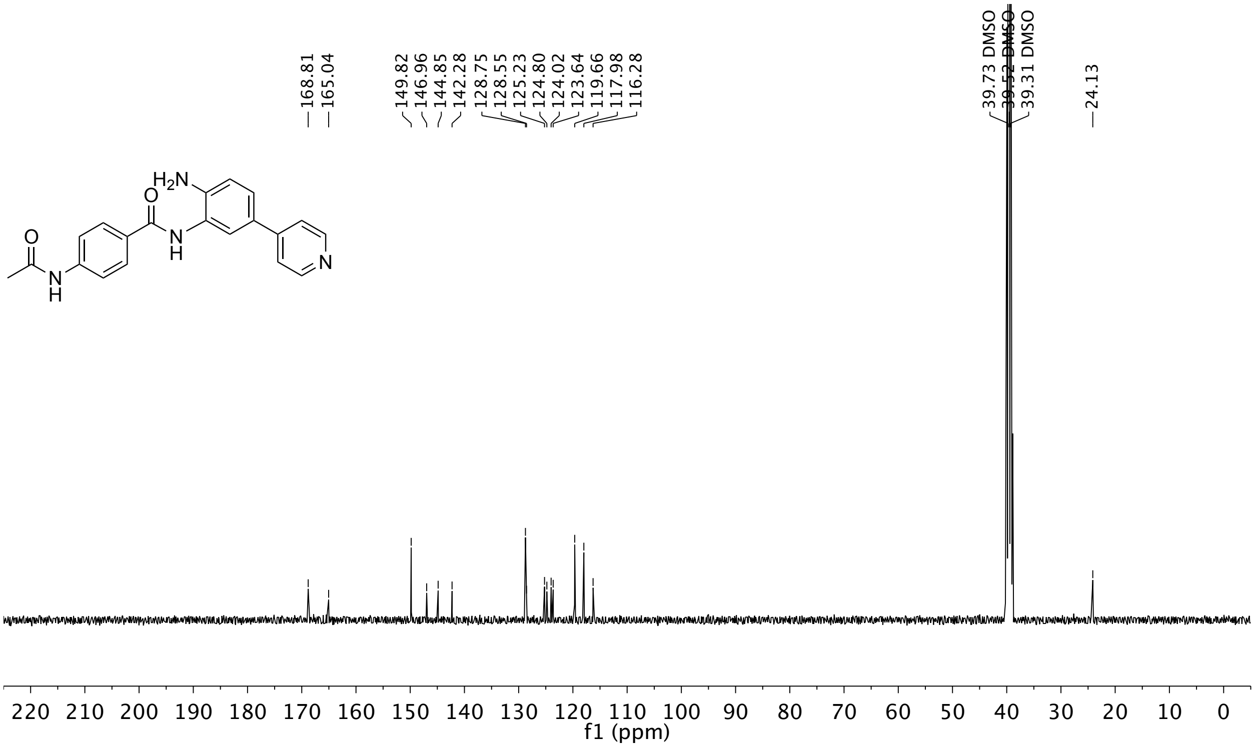

^{13}C NMR (101 MHz, DMSO-d_6) of **6d**.

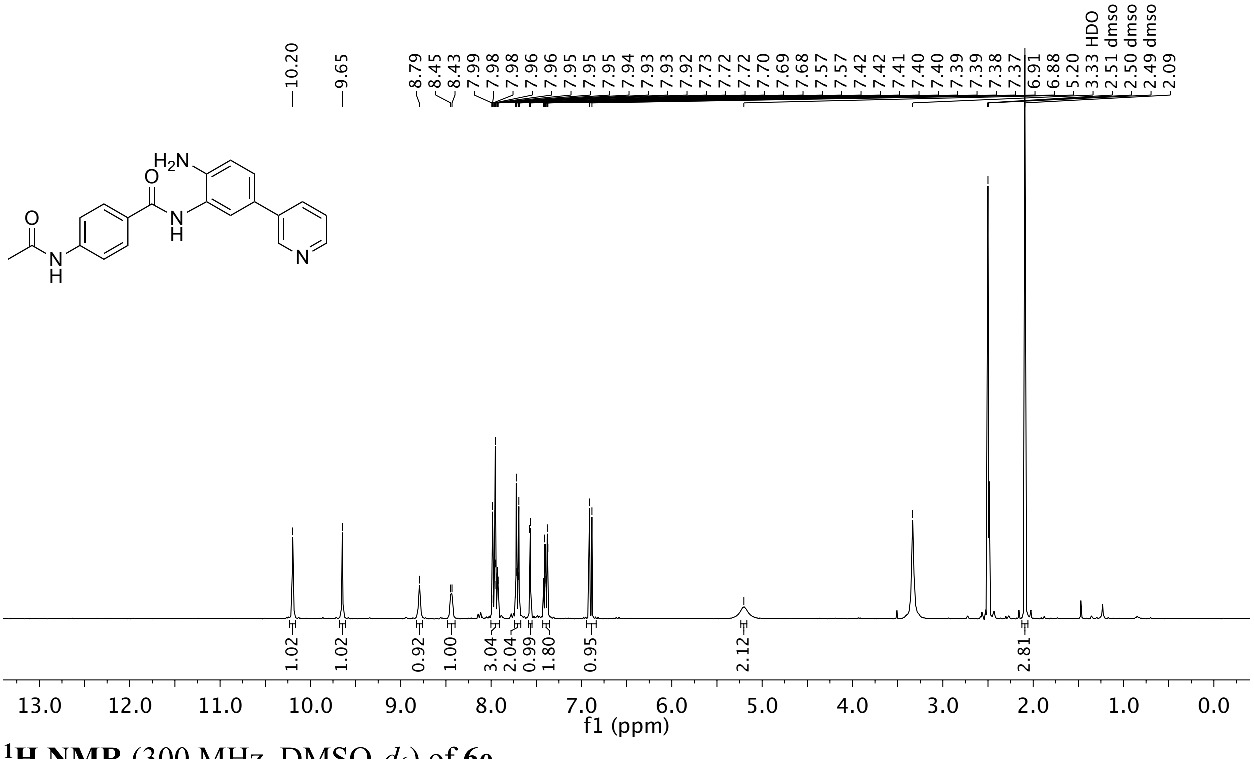

^{1}H NMR (300 MHz, DMSO-d_6) of **6e**.

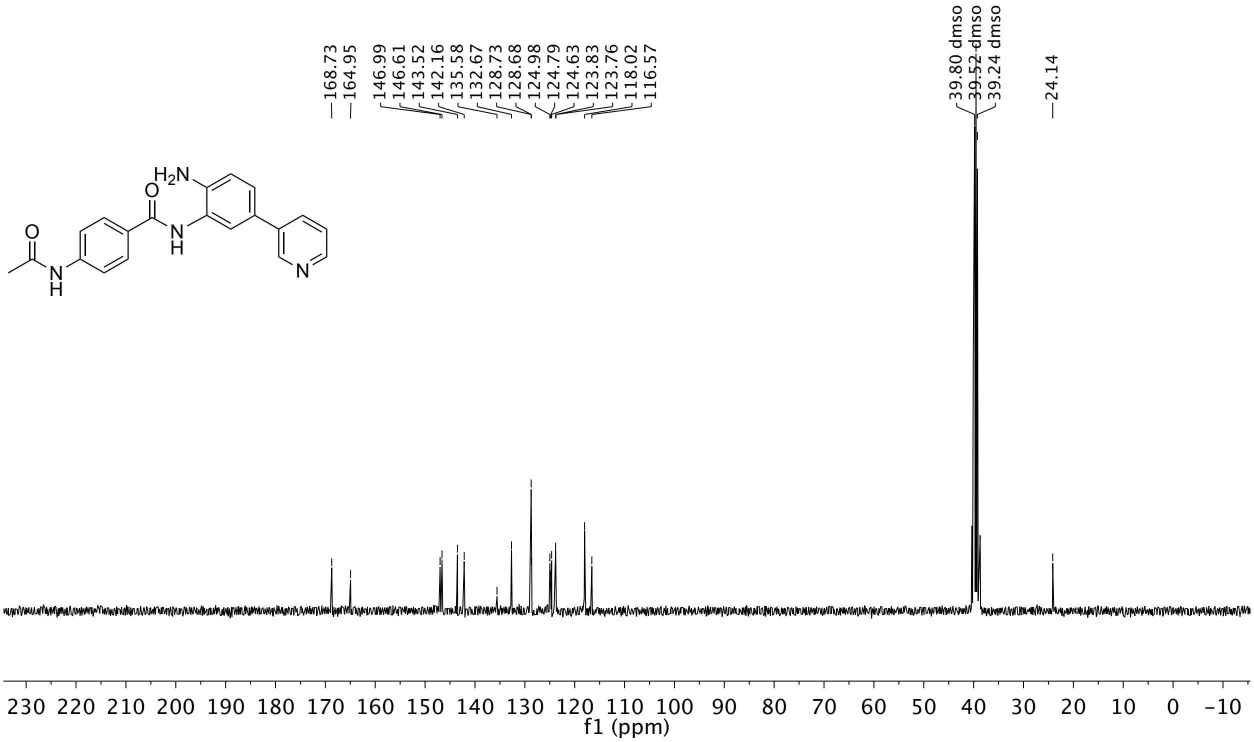

^{13}C NMR (75 MHz, DMSO-d_6) of **6e**.

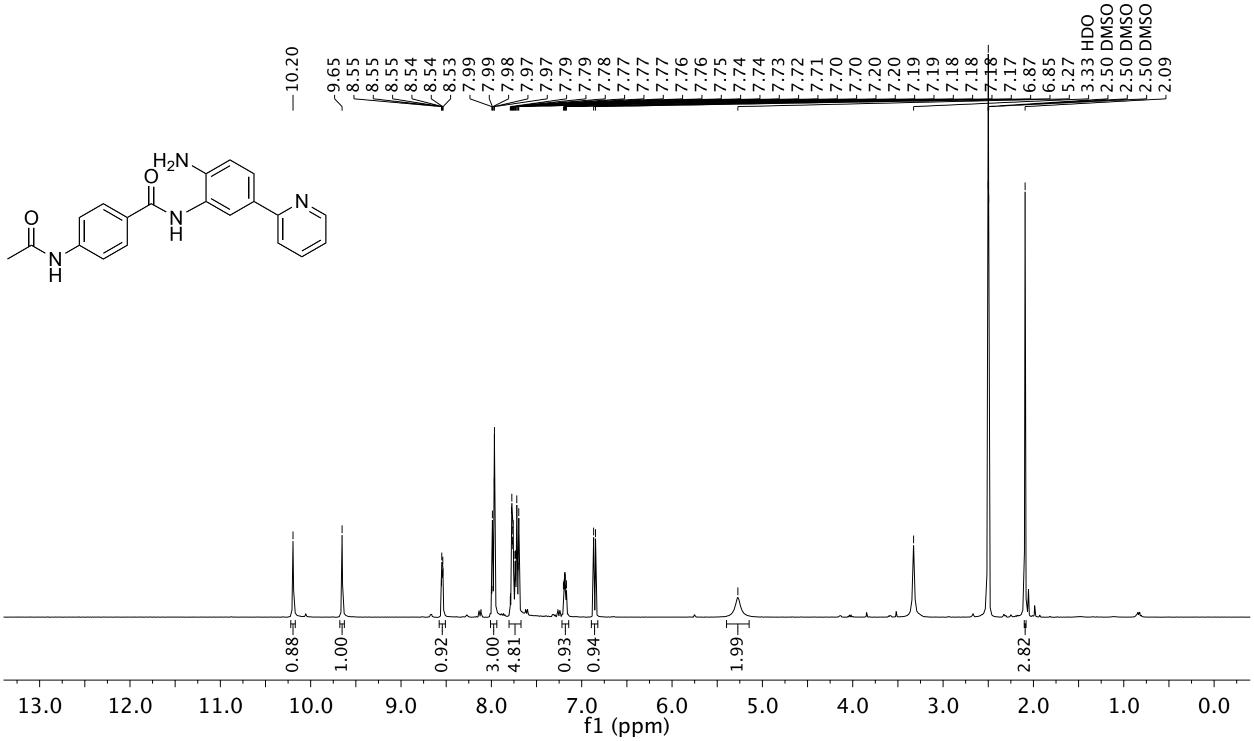

^{1}H NMR (400 MHz, DMSO-d_6) of **6f**.

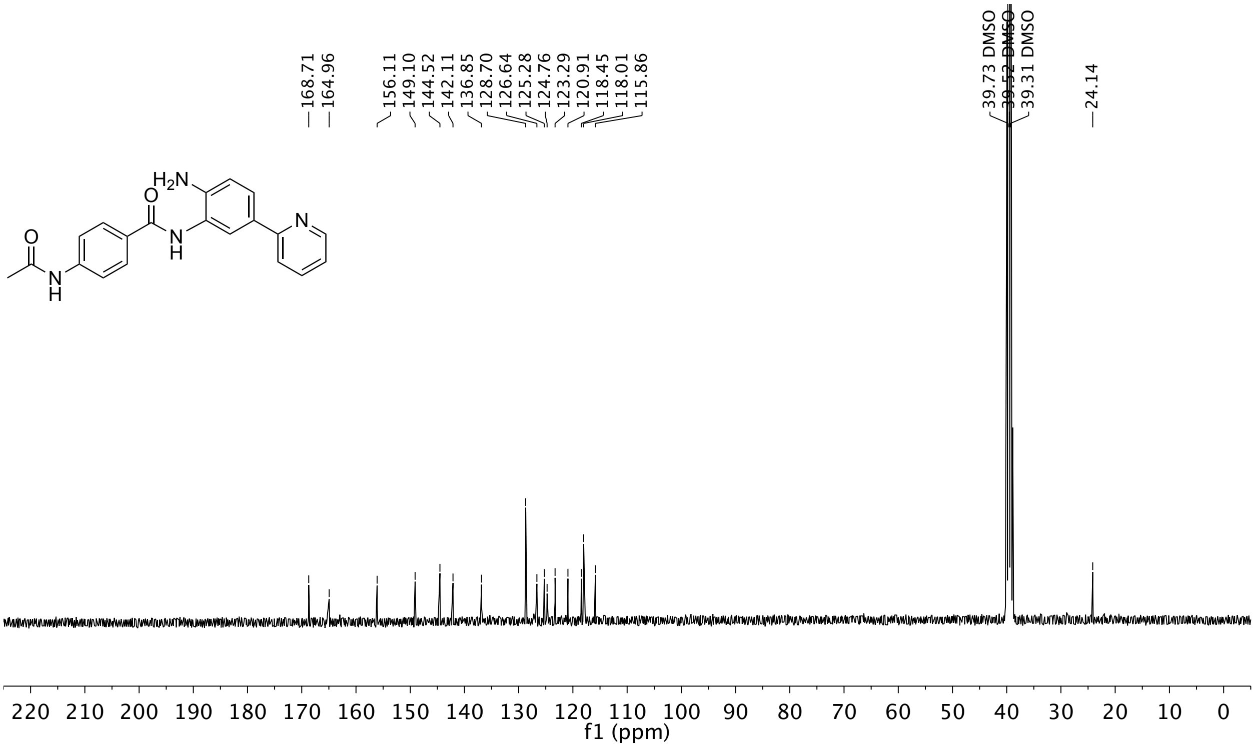

^{13}C NMR (101 MHz, DMSO-d_6) of **6f**.

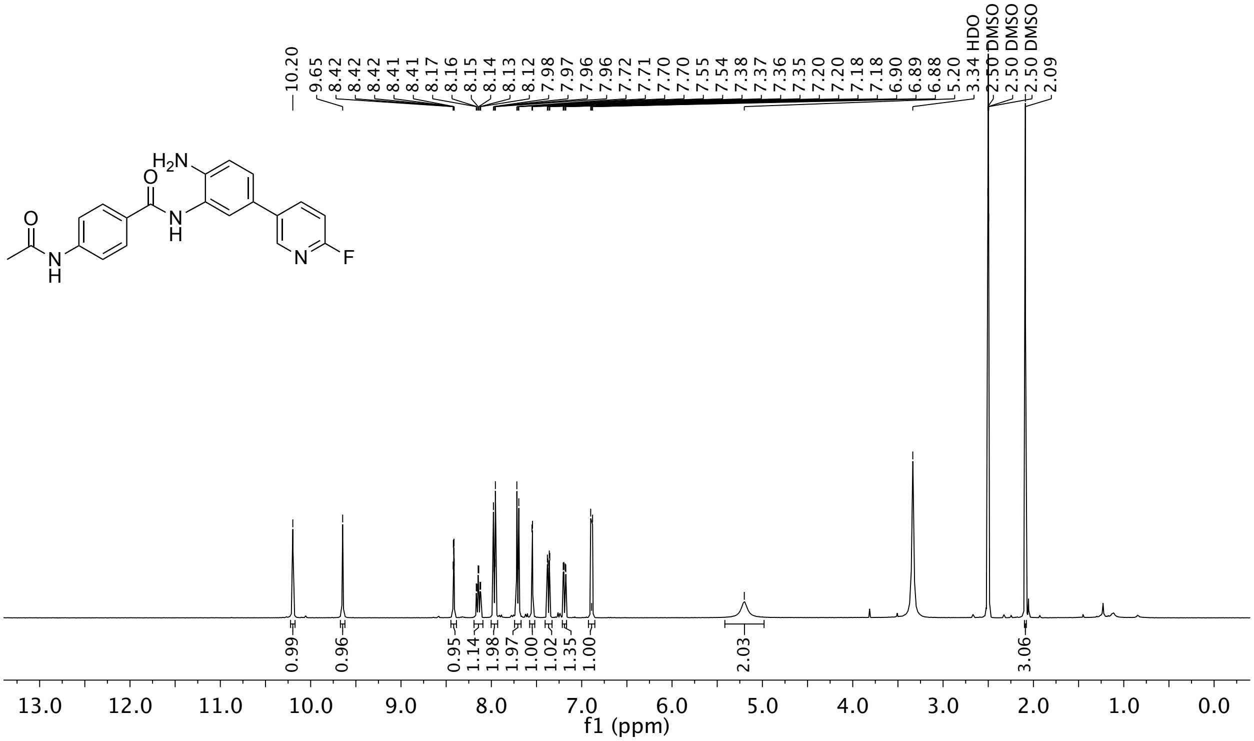

^{1}H NMR (400 MHz, DMSO-d_6) of **6g**.

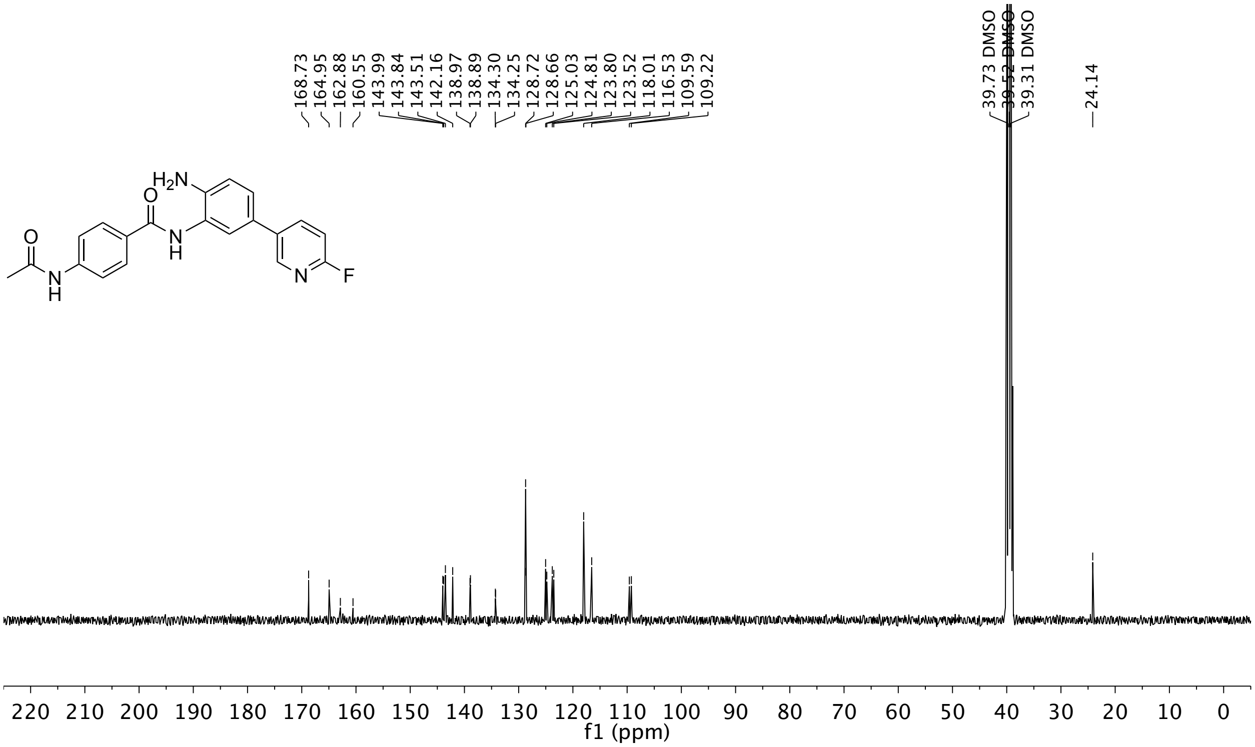

^{13}C NMR (101 MHz, DMSO-d_6) of **6g**.

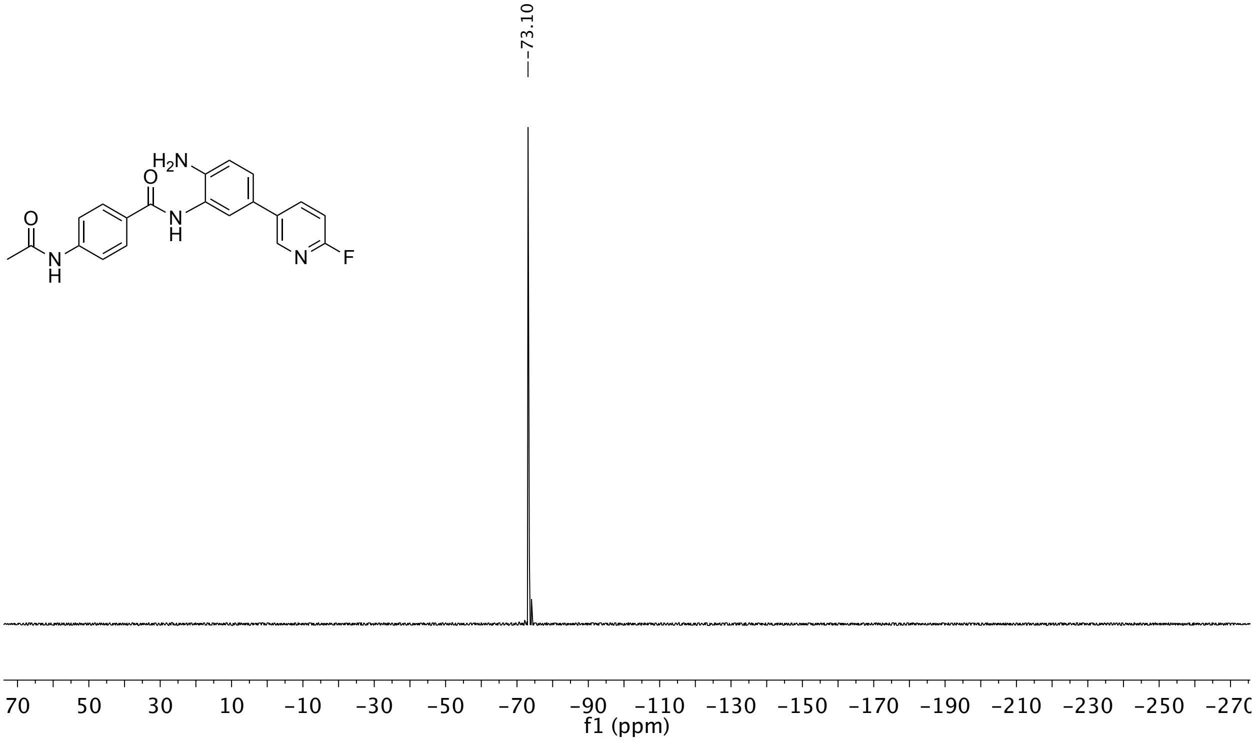

^{19}F NMR (377 MHz, DMSO-d_6) of **6g**.

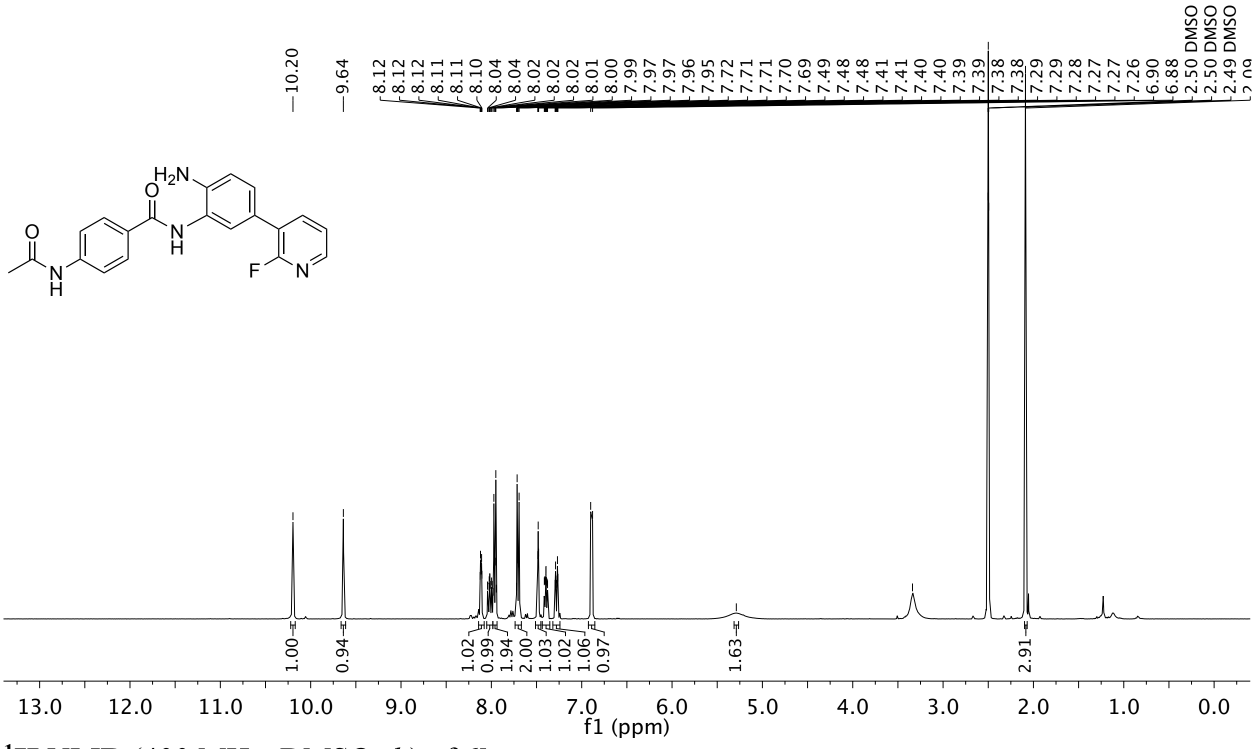

^{1}H NMR (400 MHz, DMSO-d_6) of **6h**.

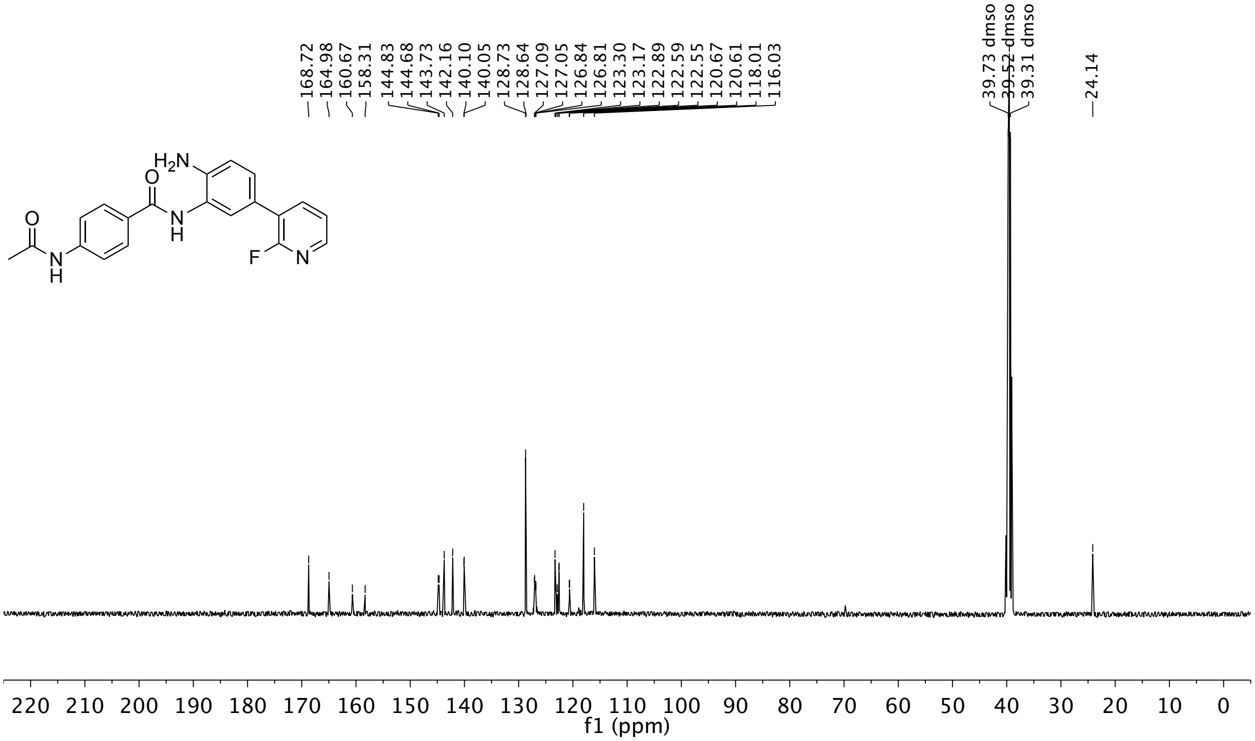

^{13}C NMR (101 MHz, DMSO-d_6) of **6h**.

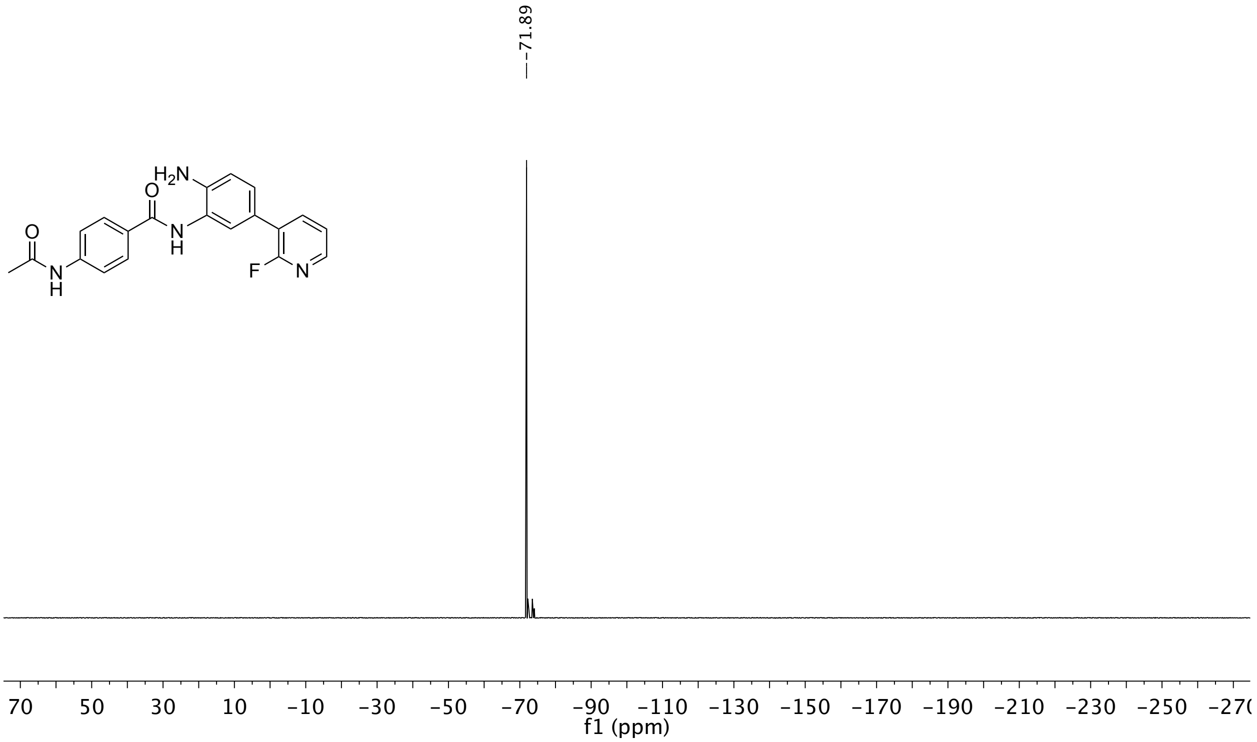

^{19}F NMR (377 MHz, DMSO-d_6) of **6h**.

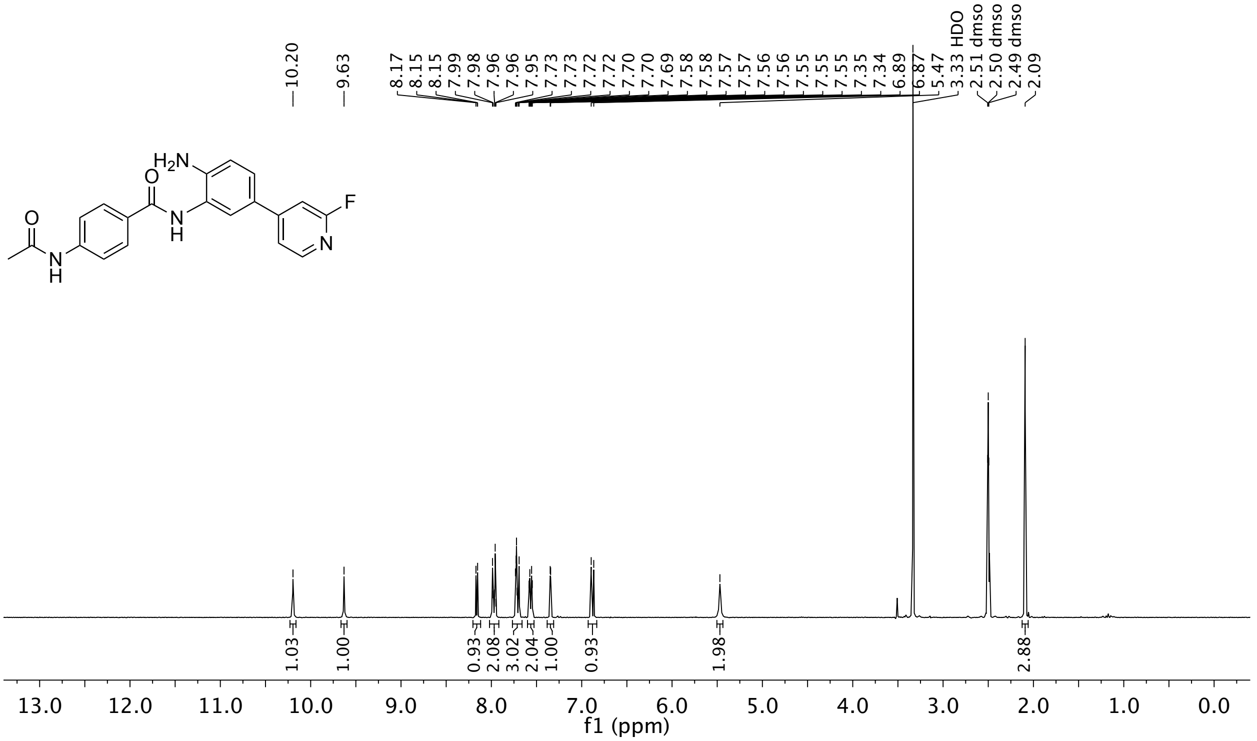

^{1}H NMR (300 MHz, DMSO-d_6) of **6i**.

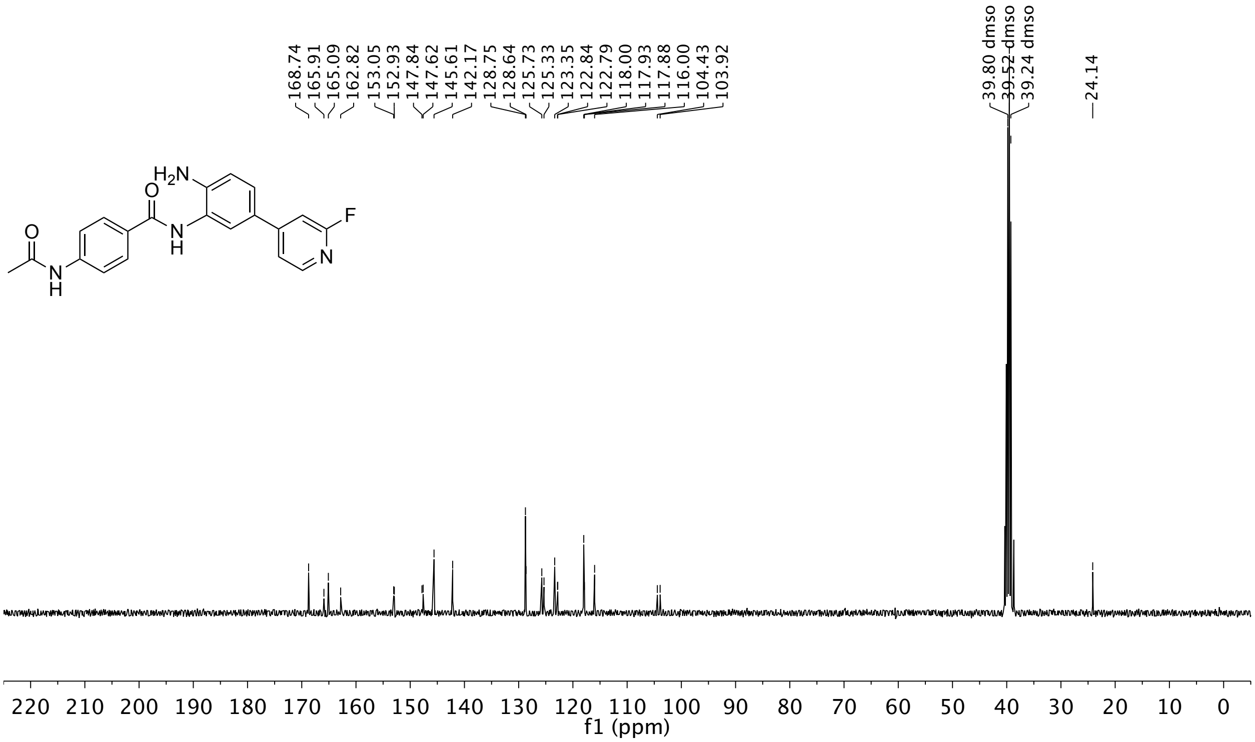

^{13}C NMR (75 MHz, DMSO-d_6) of **6i**.

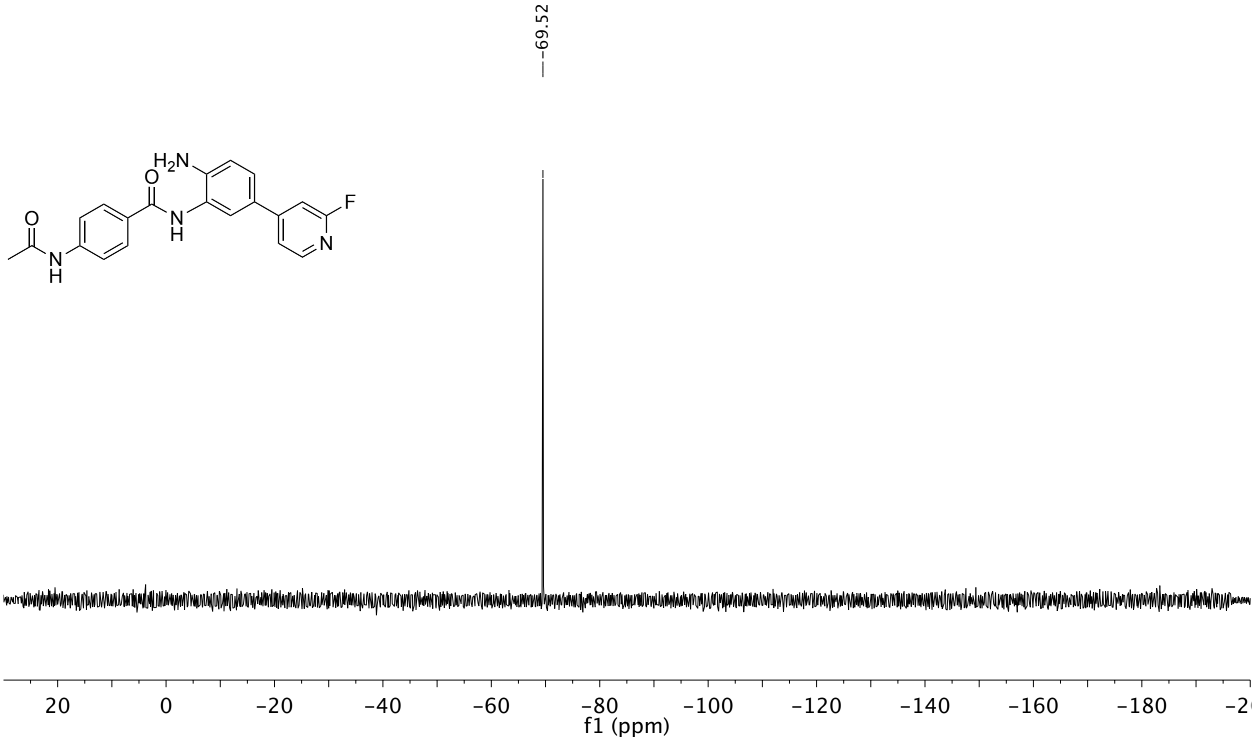

^{19}F NMR (282 MHz, DMSO-d_6) **6i**.

Synthesis of full-sized inhibitors 10a-c

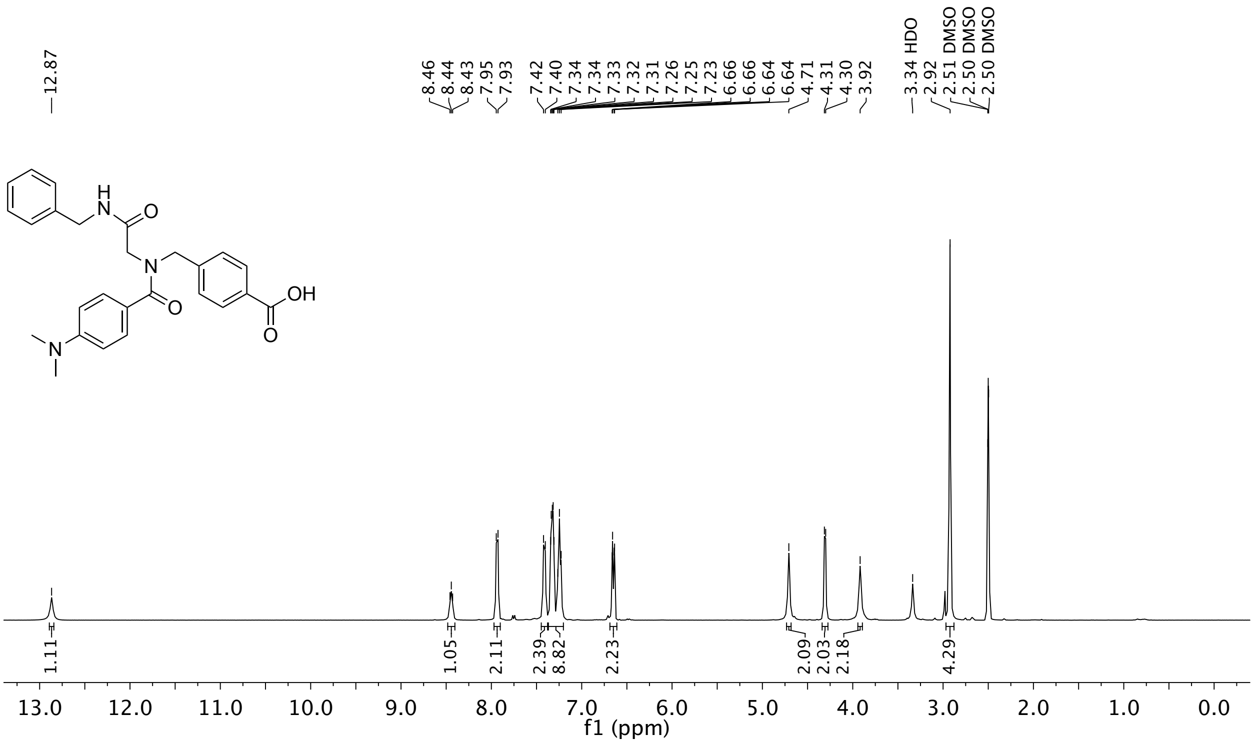

^{1}H NMR (400 MHz, DMSO-d_6) of **8a**.

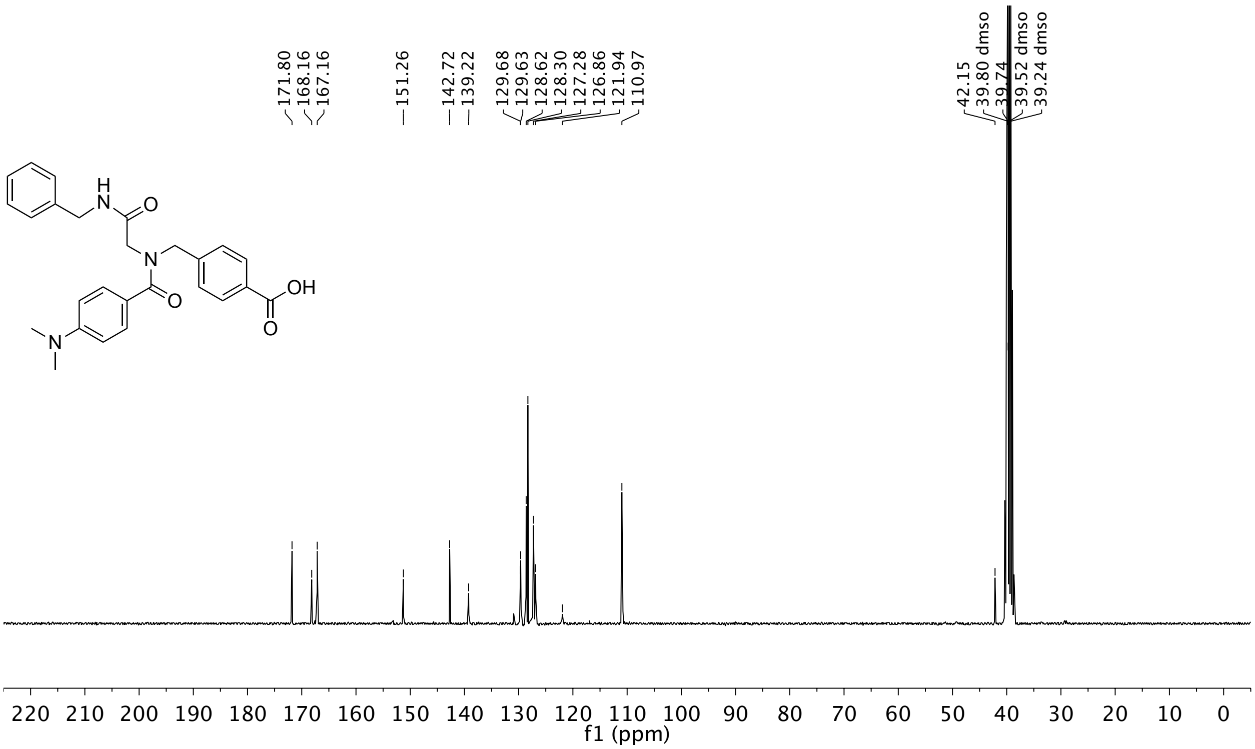

^{13}C NMR (75 MHz, DMSO-d_6) of **8a**.

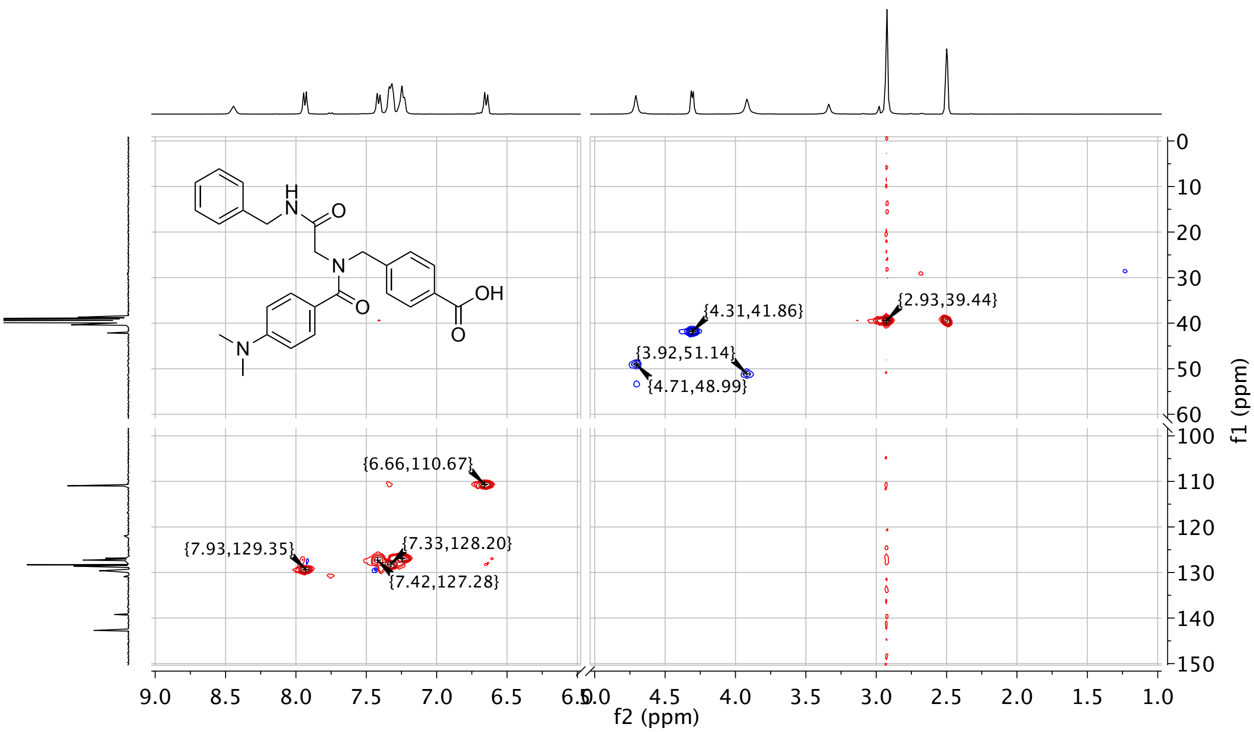

^{1}H, ^{13}C **HSQC** (75 MHz, DMSO-d_6) of **8a**.

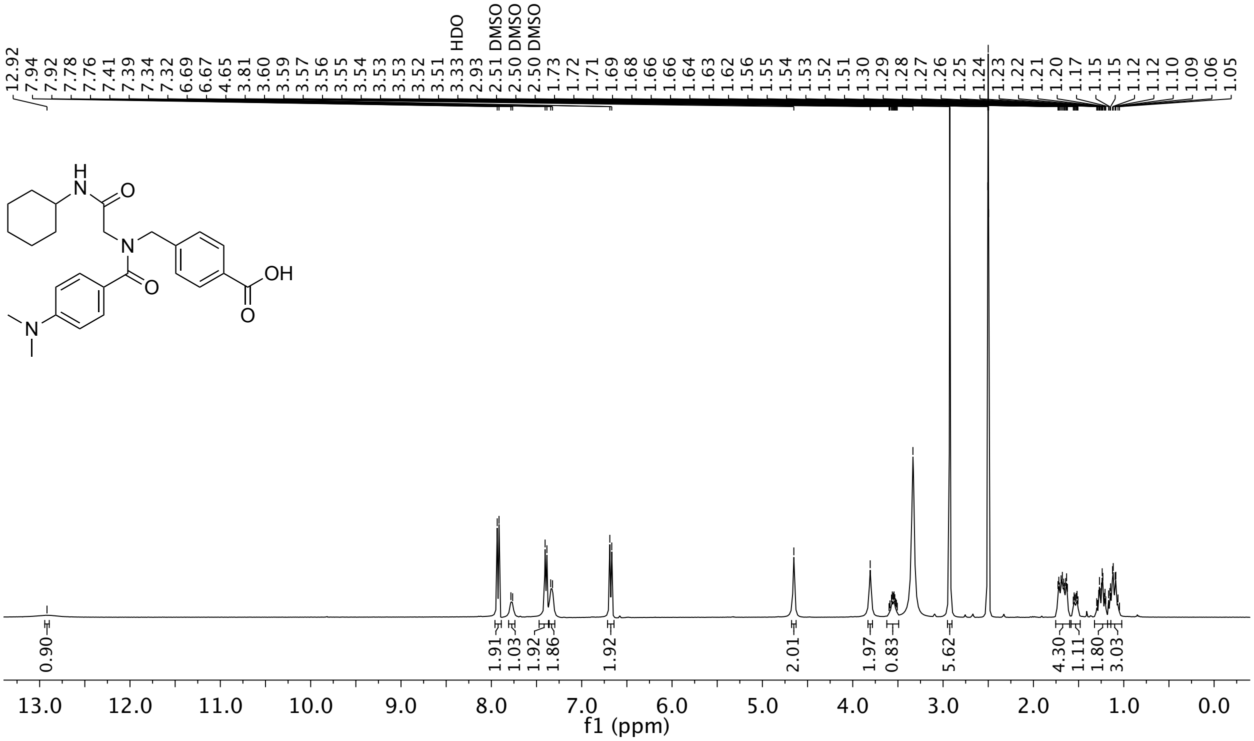

^{1}H **NMR** (400 MHz, DMSO-d_6) of **8b**.

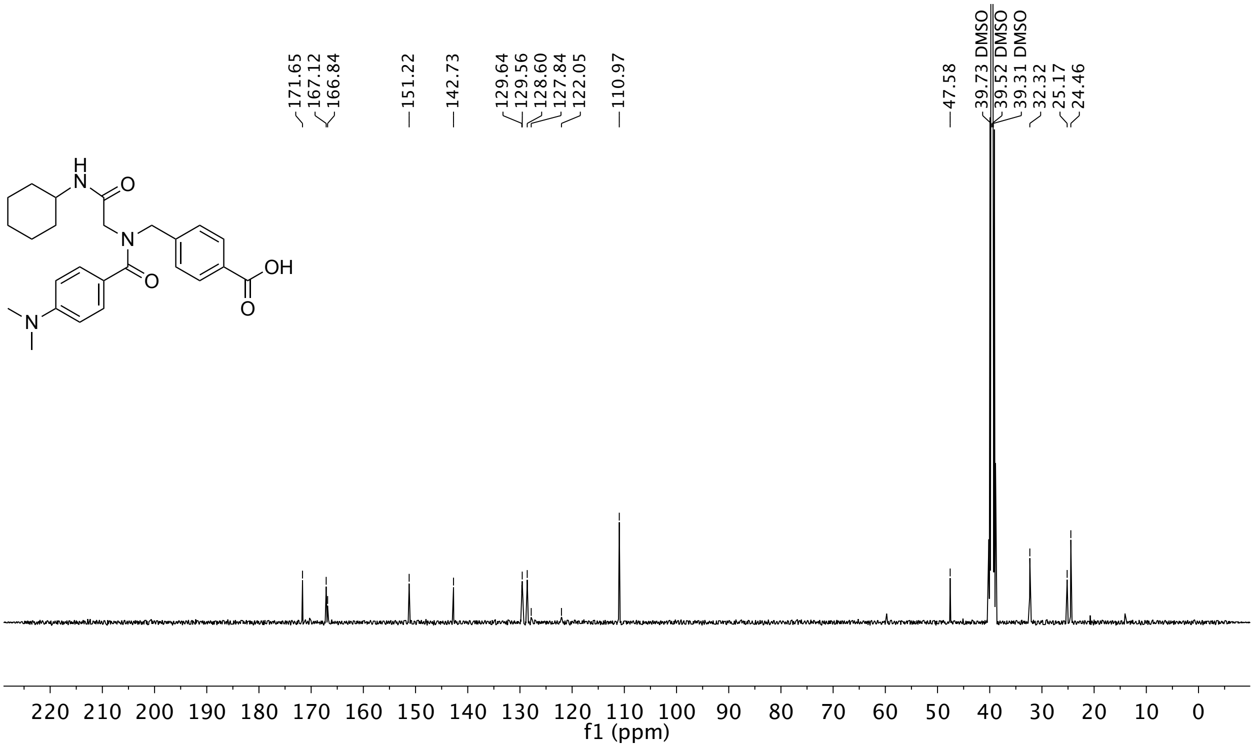

^{13}**C NMR** (101 MHz, DMSO-d_6) of **8b**.

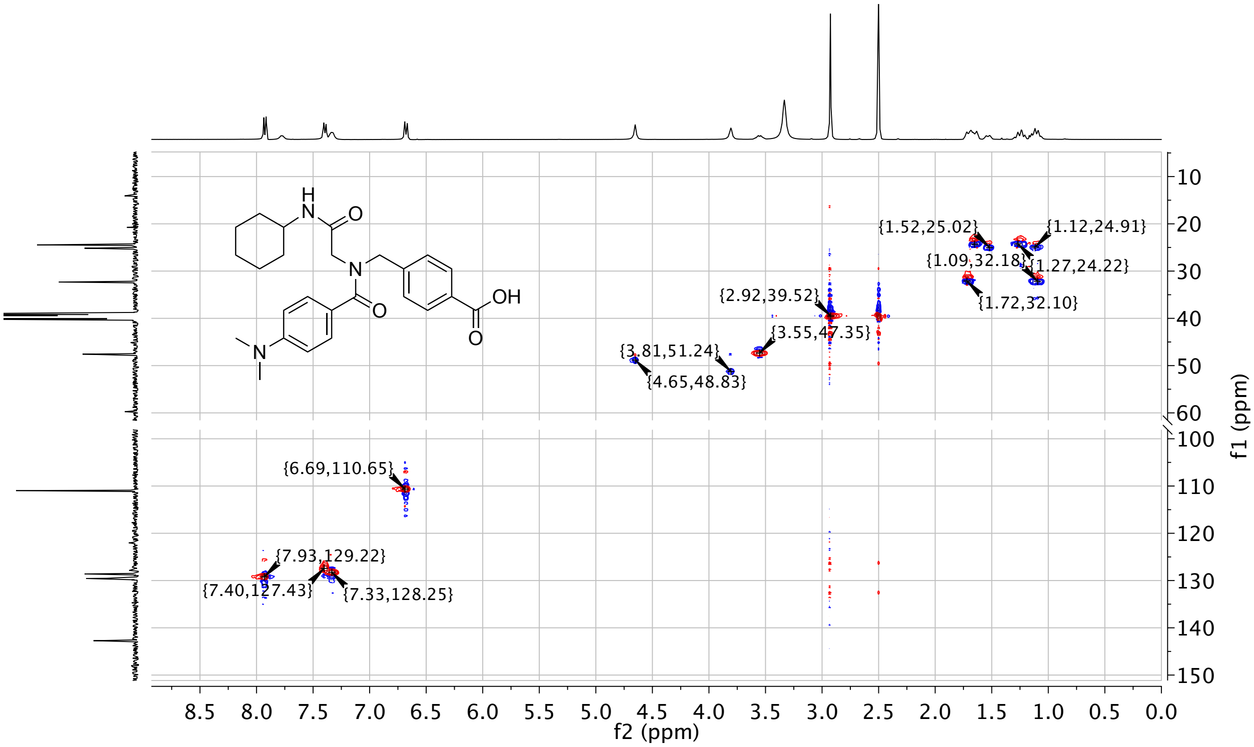

^{1}**H,** ^{13}**C HSQC** (101 MHz, DMSO-d_6) of **8b**.

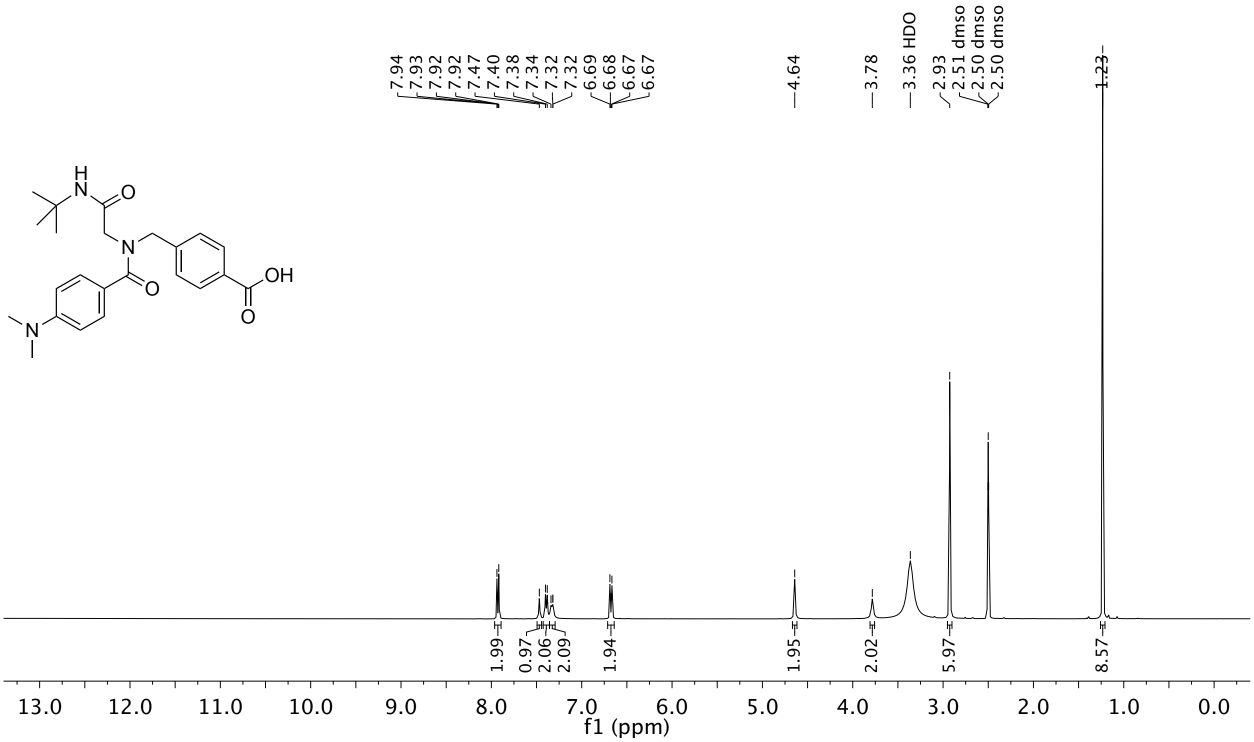

^{1}H NMR (400 MHz, DMSO-d_6) of **8c**.

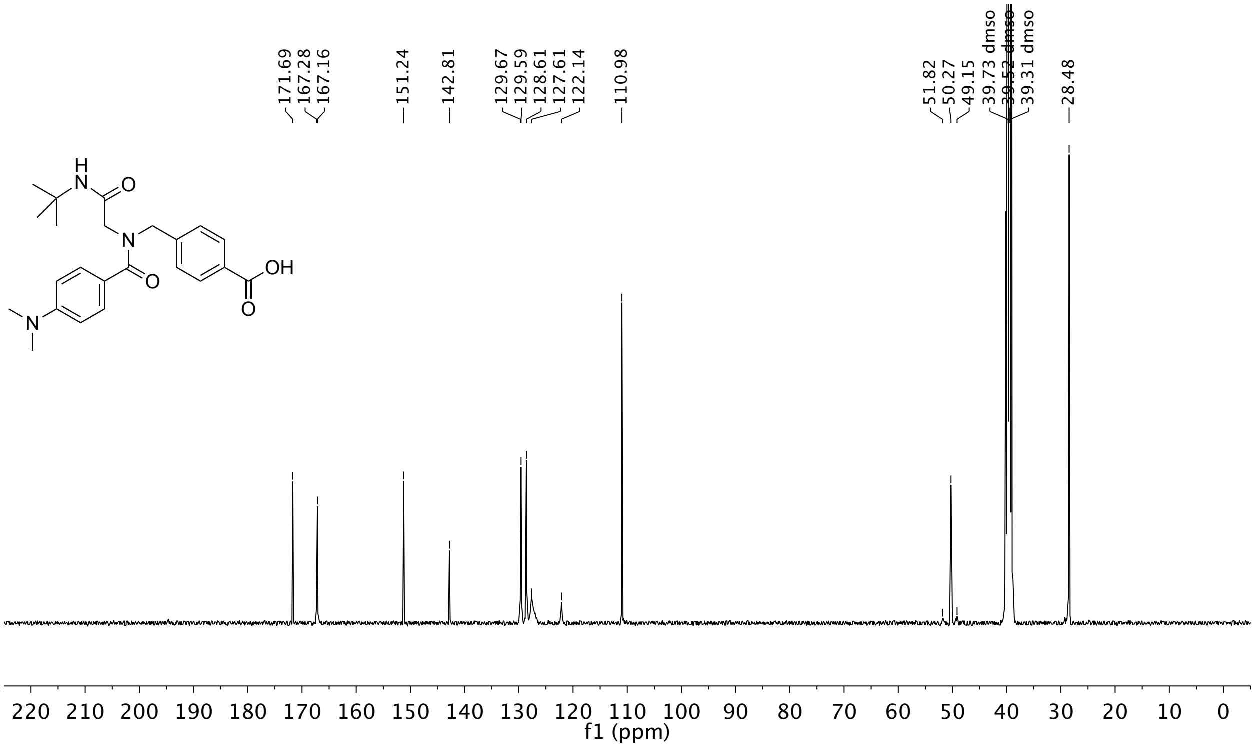

^{13}C NMR (101 MHz, DMSO-d_6) of **8c**.

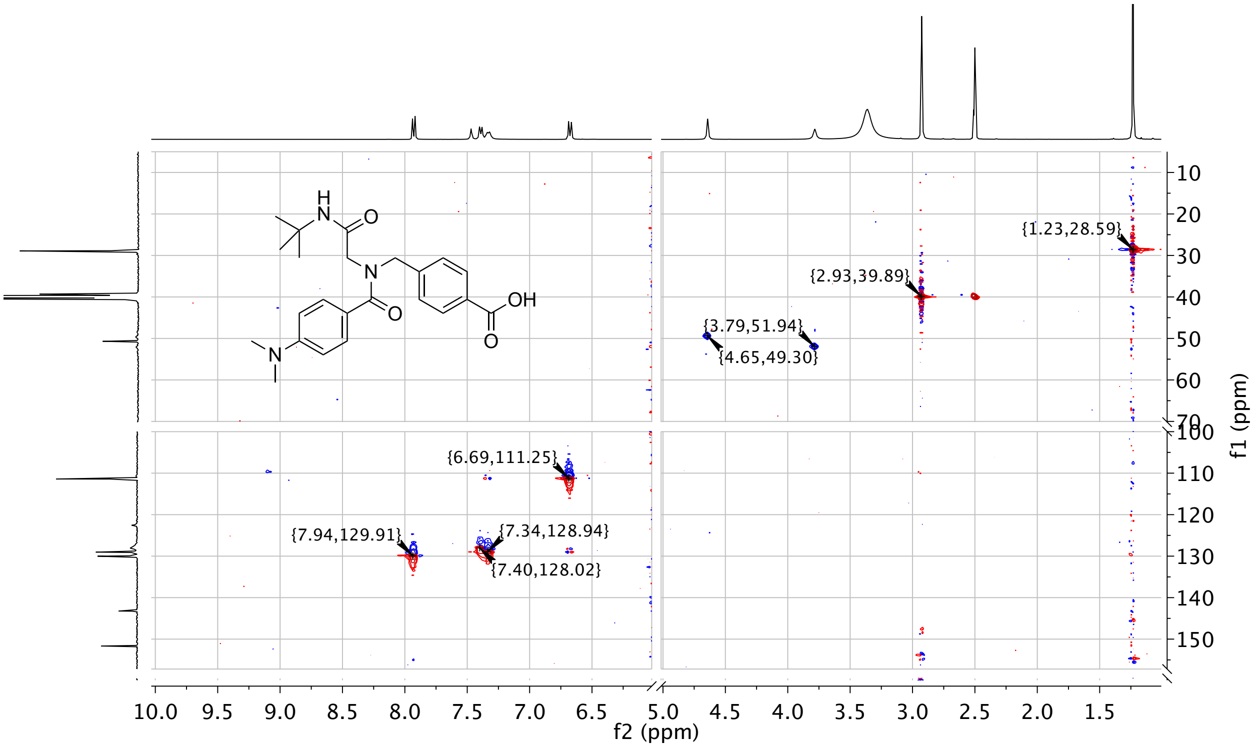

^{1}H, ^{13}C HSQC (101 MHz, DMSO-d_6) of **8c**.

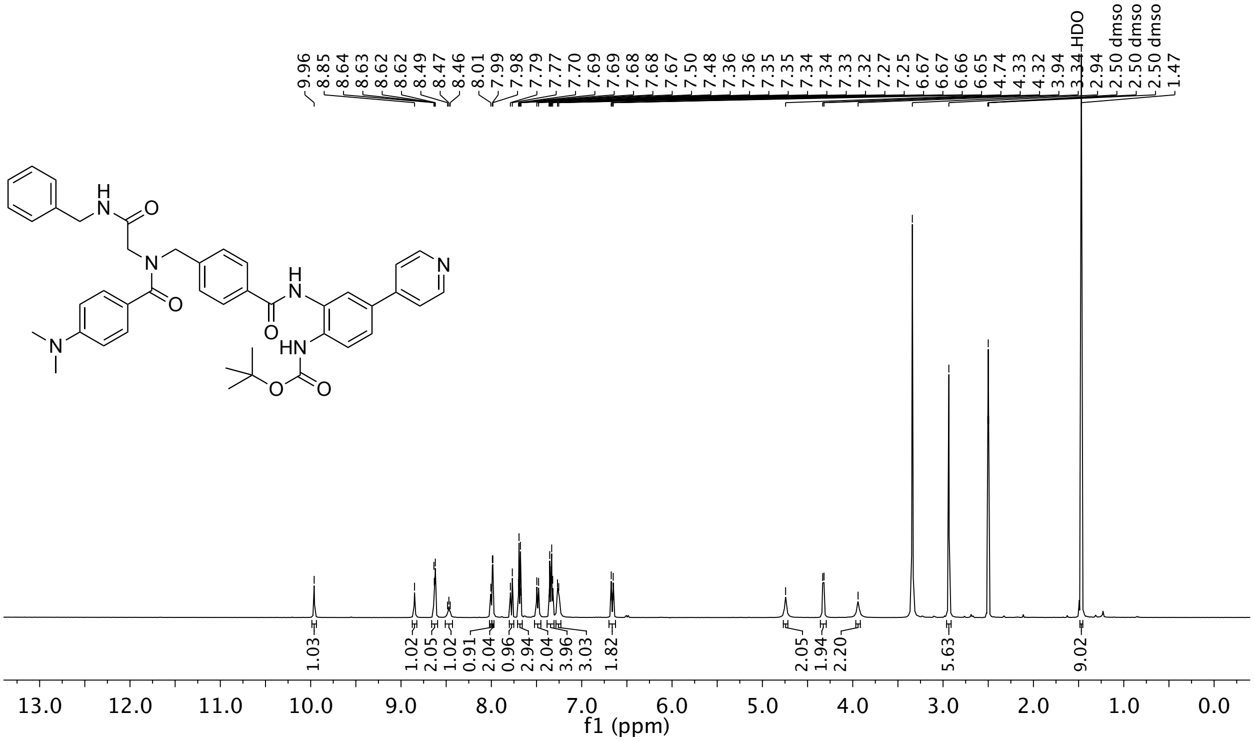

^{1}H NMR (400 MHz, DMSO-d_6) of **9a**.

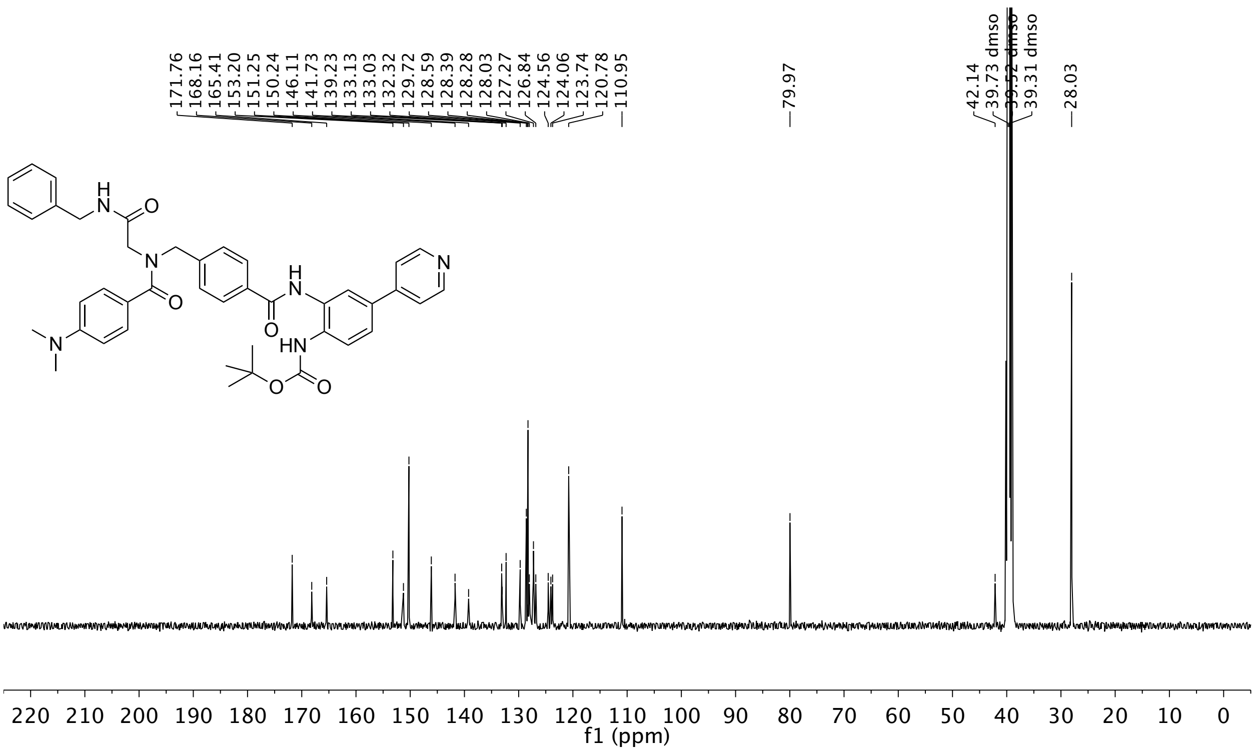

^{13}C NMR (101 MHz, DMSO-d_6) of **9a**.

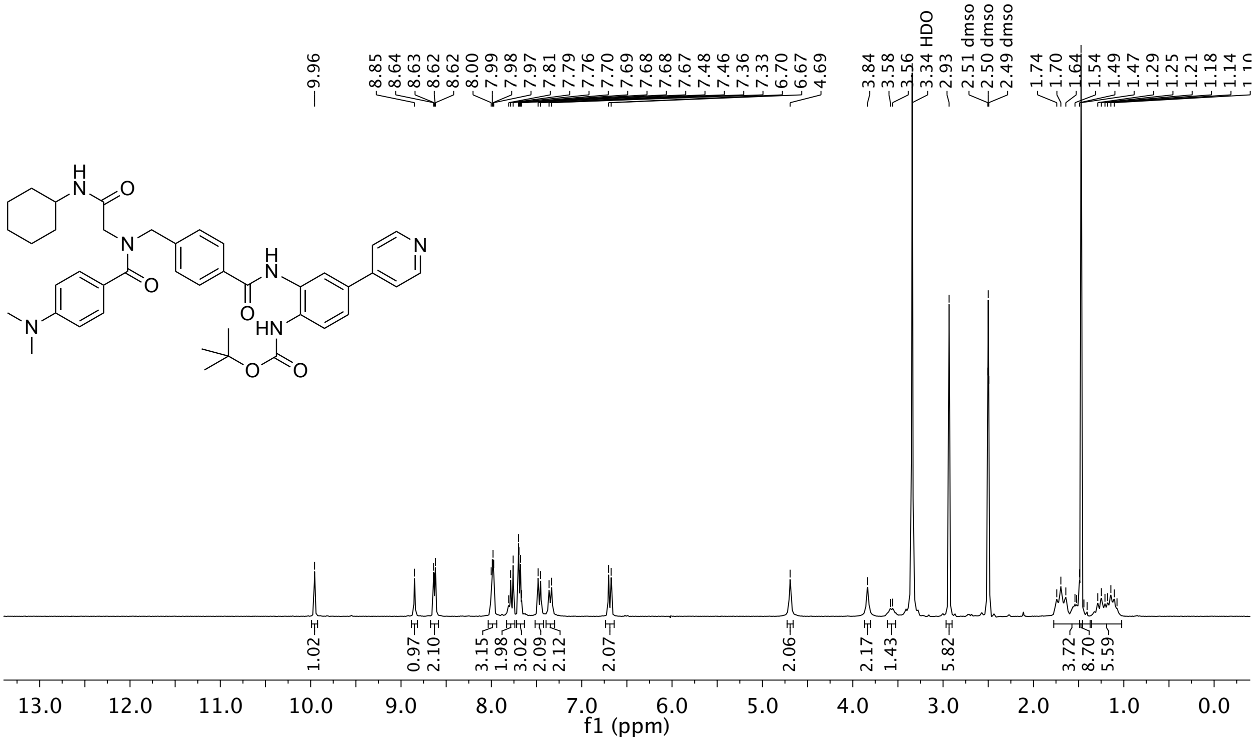

^{1}**H NMR** (300 MHz, DMSO-d_6) of **9b**.

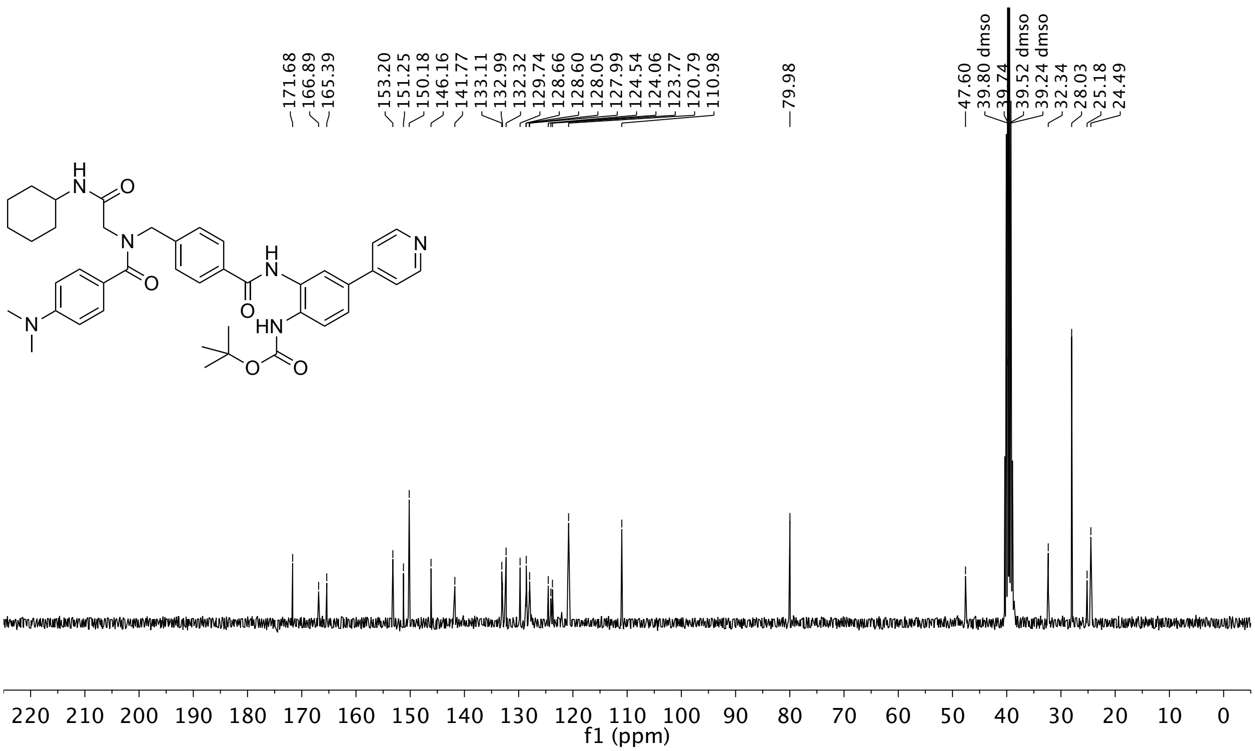

^{13}**C NMR** (75 MHz, DMSO-d_6) of **9b**.

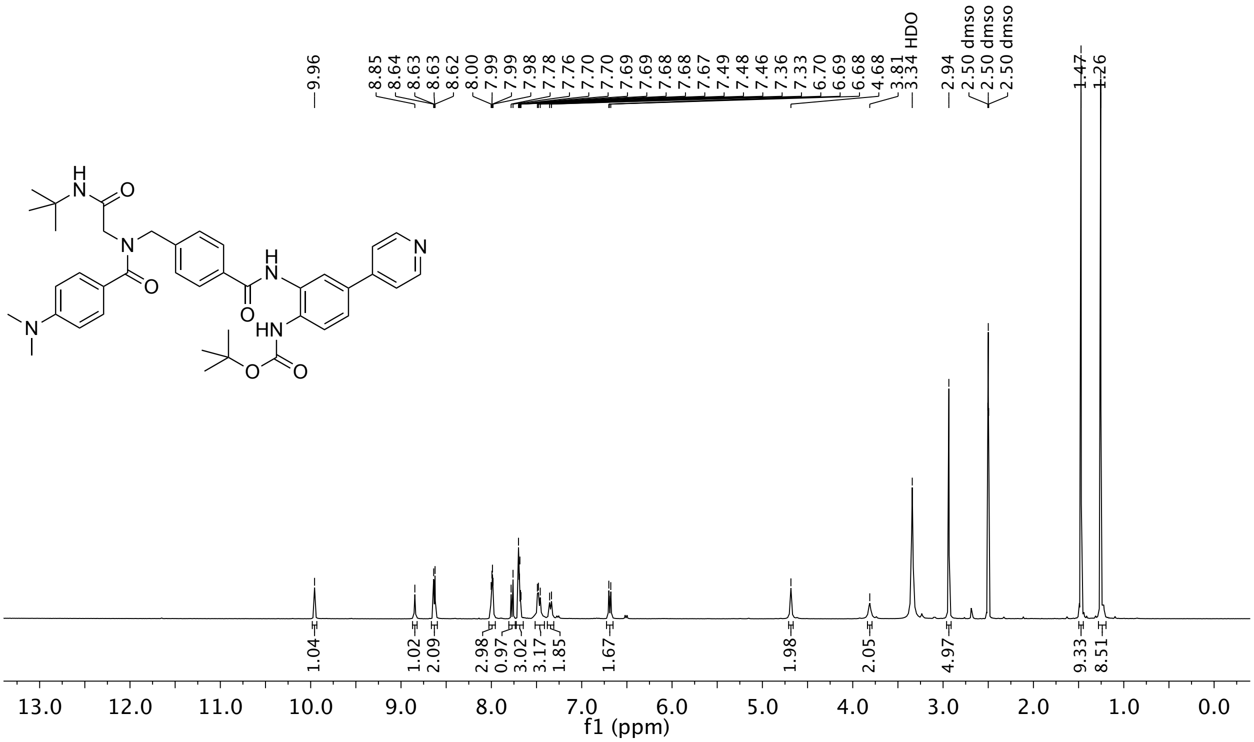

^{1}H NMR (400 MHz, DMSO-d_6) of **9c**.

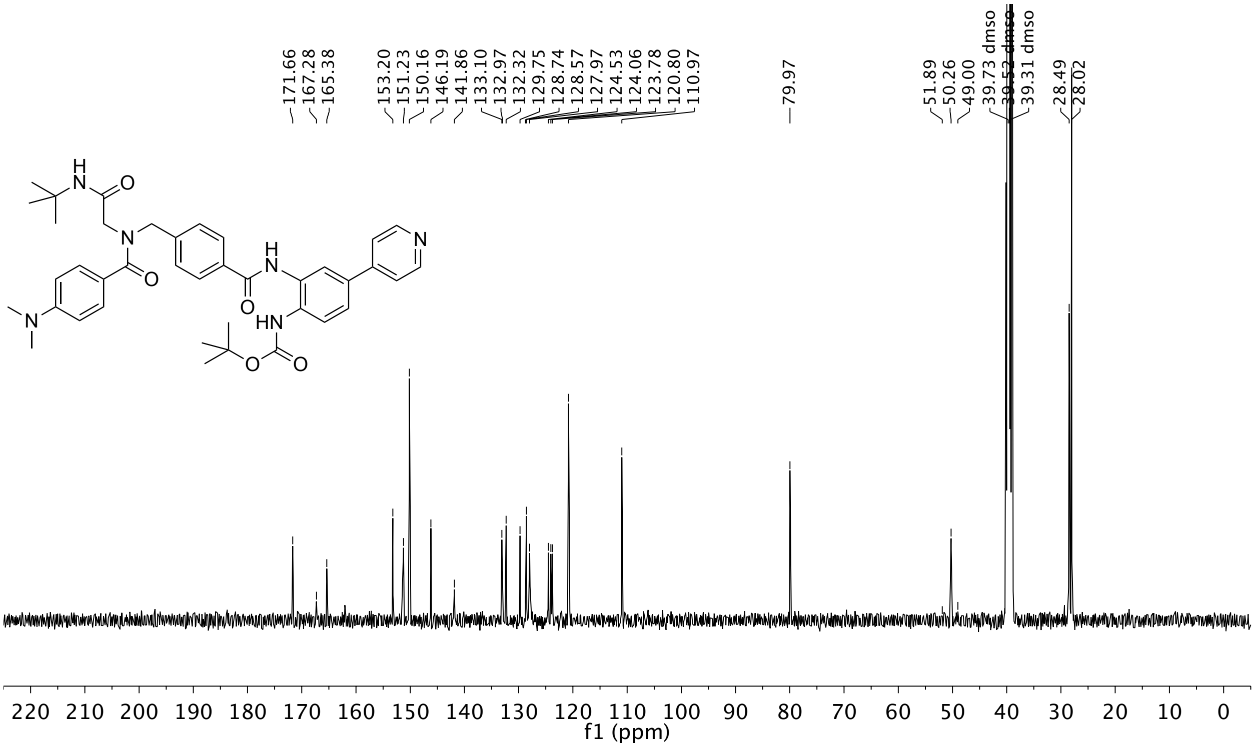

^{13}C NMR (101 MHz, DMSO-d_6) of **9c**.

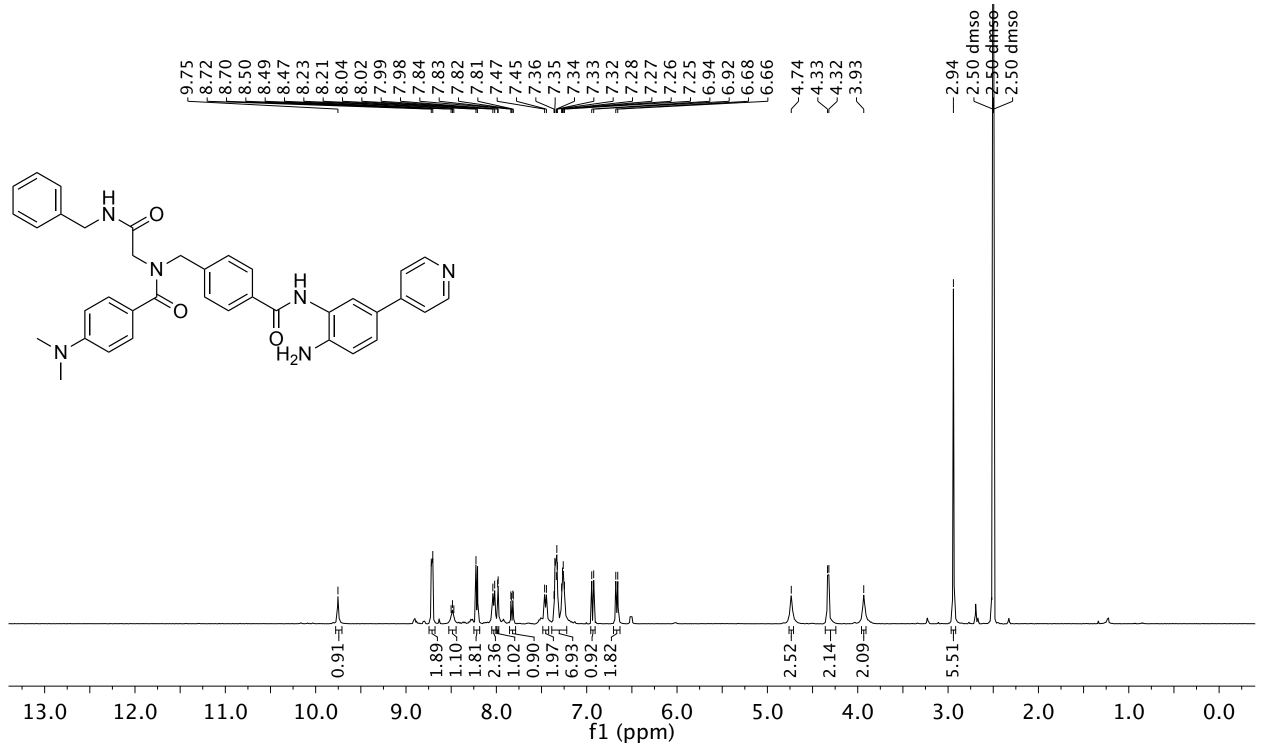

^{1}H NMR (400 MHz, DMSO-d_6 + TFA (1.0 equiv.)) of **10a**.

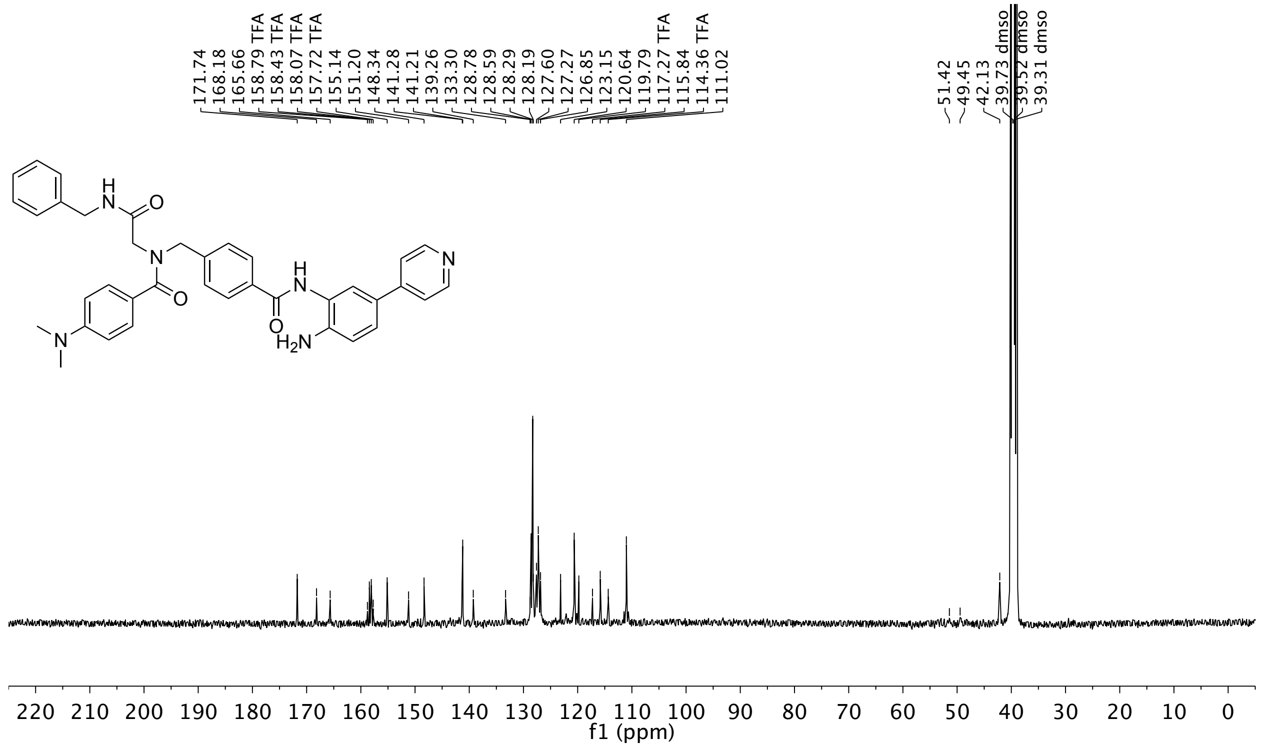

^{13}C NMR (101 MHz, DMSO-d_6 + TFA (1.0 equiv.)) of **10a**.

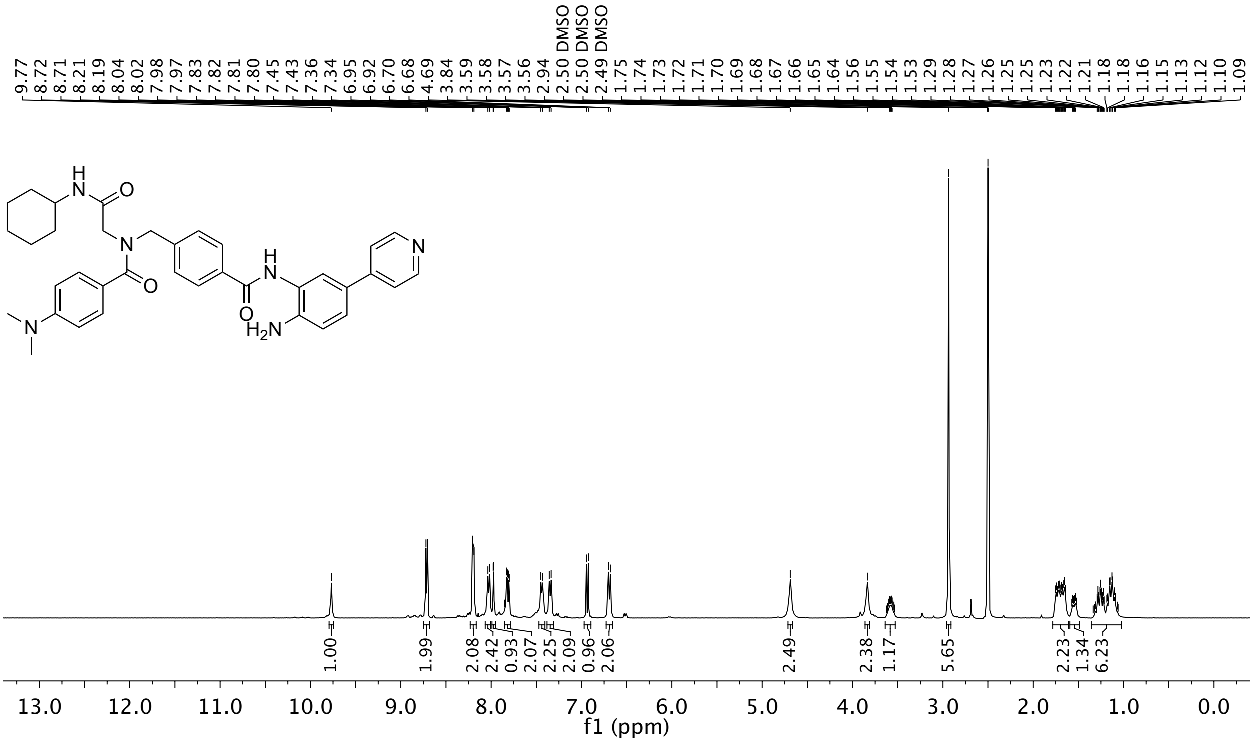

^{1}H NMR (400 MHz, DMSO-d_6 + TFA (1.0 equiv.)) of **10b**.

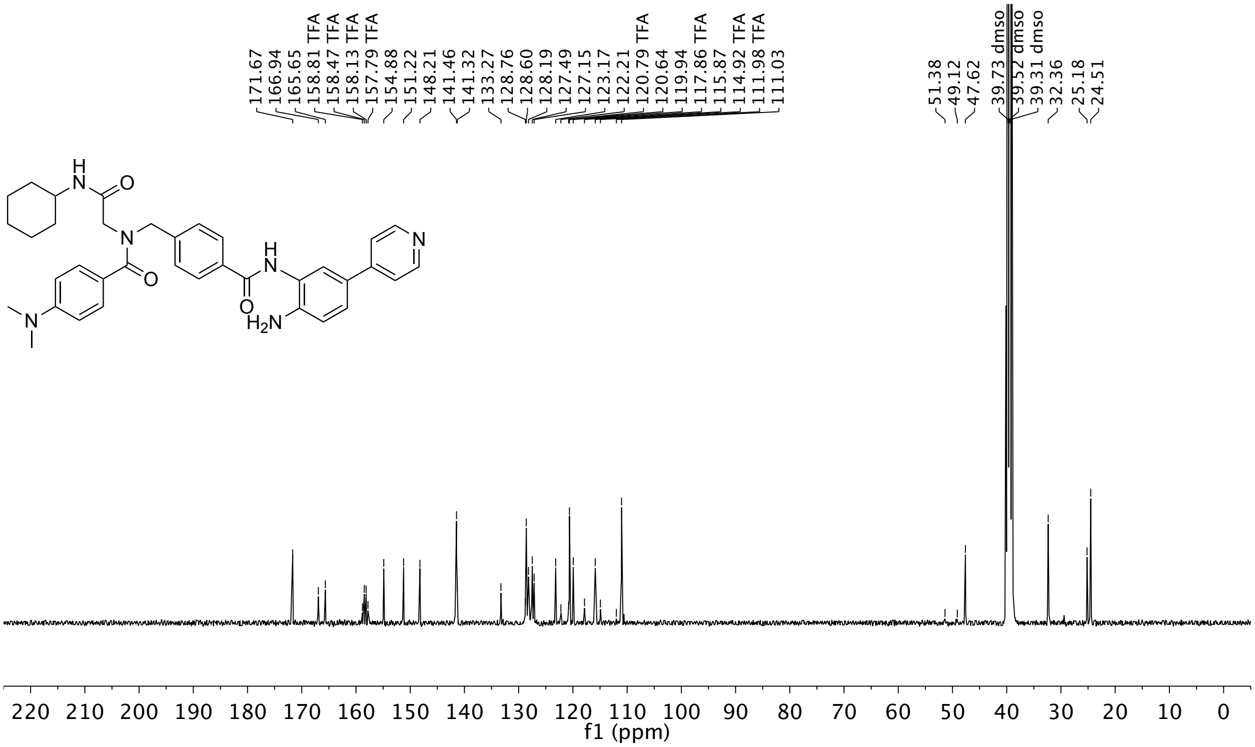

^{13}C NMR (101 MHz, DMSO-d_6 + TFA (1.0 equiv.)) of **10b**.

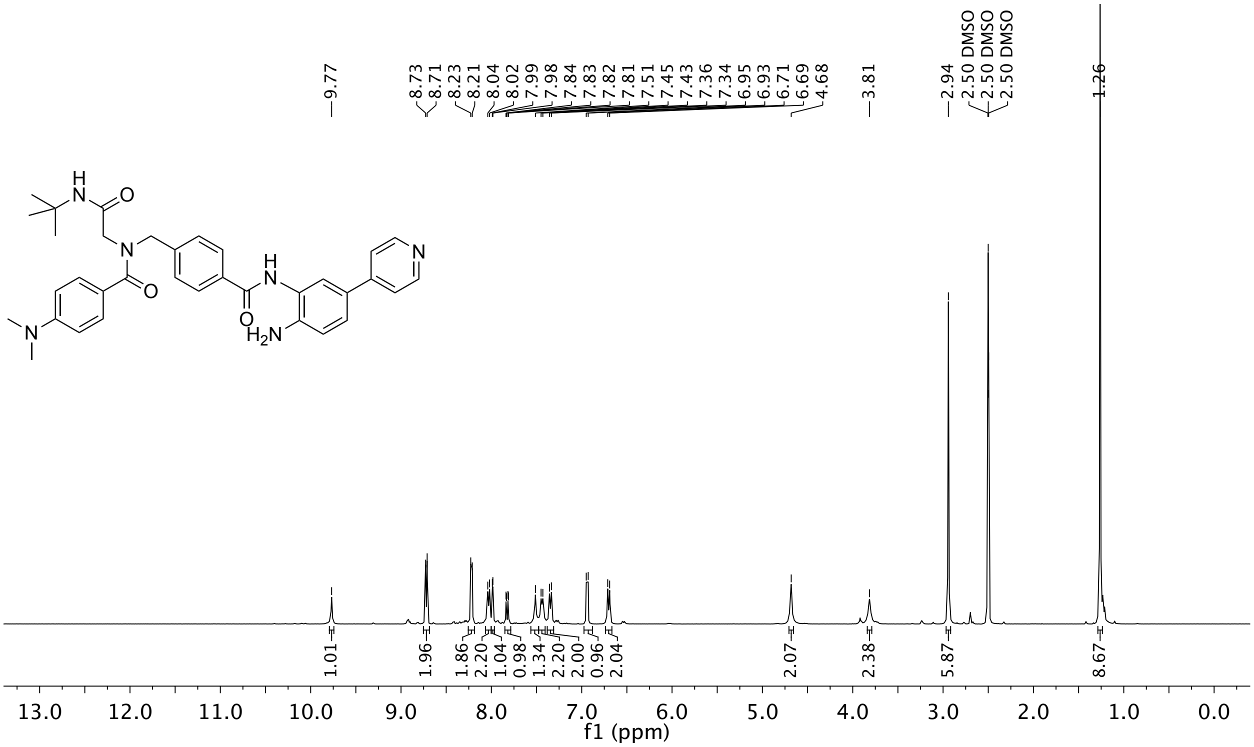

^{1}H NMR (400 MHz, DMSO-d_6 + TFA (1.0 equiv.)) of **10c**.

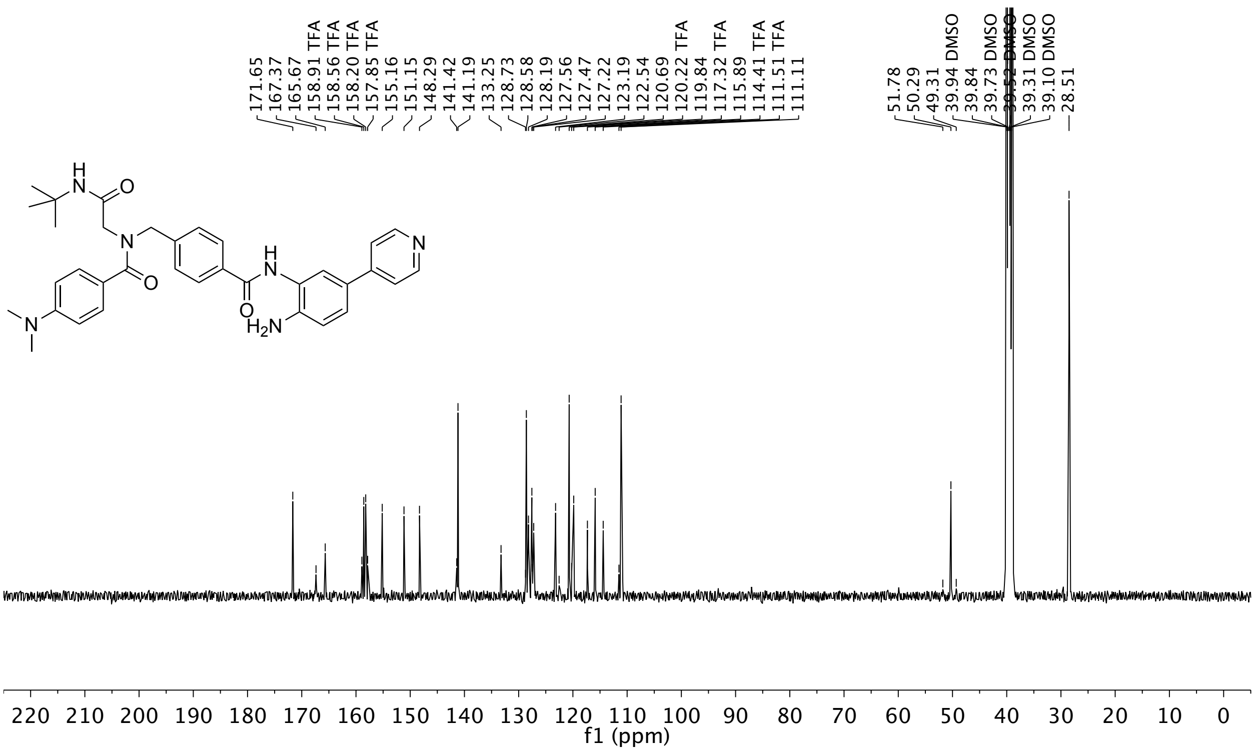

^{13}C NMR (101 MHz, DMSO-d_6 + TFA (1.0 equiv.)) of **10c**.

HPLC CHROMATOGRAMS OF TARGET COMPOUNDS

HPLC chromatogram of **6a**. (*Thermo Fisher Scientific* UltiMate[TM] 3000).

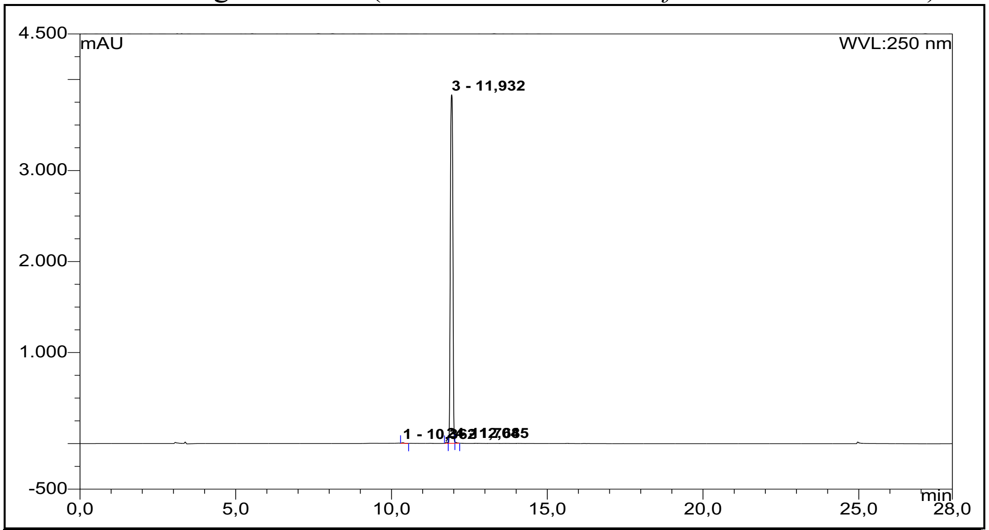

No.	Ret.Time min	Peak Name	Height mAU	Area mAU*min	Rel.Area %	Amount	Type
1	10,36	n.a.	5,942	0,496	0,14	n.a.	MB*
2	11,77	n.a.	15,996	0,978	0,27	n.a.	BM *
3	11,93	n.a.	#######	360,758	99,38	n.a.	M *
4	12,05	n.a.	13,347	0,765	0,21	n.a.	MB*
Total:			#######	362,997	100,00	0,000	

HPLC chromatogram of **6b**. (*Thermo Fisher Scientific* UltiMate[TM] 3000).

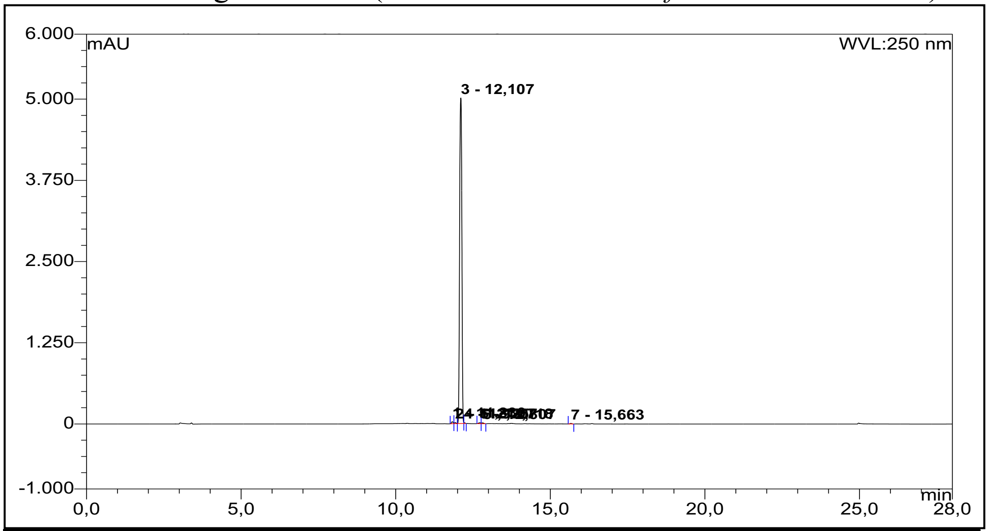

No.	Ret.Time min	Peak Name	Height mAU	Area mAU*min	Rel.Area %	Amount	Type
1	11,83	n.a.	31,204	1,975	0,46	n.a.	BM *
2	11,93	n.a.	16,330	1,065	0,25	n.a.	M *
3	12,11	n.a.	#######	419,956	98,53	n.a.	M *
4	12,21	n.a.	11,567	0,424	0,10	n.a.	MB*
5	12,72	n.a.	14,623	1,184	0,28	n.a.	BM *
6	12,81	n.a.	12,802	1,019	0,24	n.a.	MB*
7	15,66	n.a.	7,928	0,594	0,14	n.a.	BMB*
Total:			#######	426,216	100,00	0,000	

HPLC chromatogram of **6c**. (*Thermo Fisher Scientific* UltiMate^{TM} 3000).

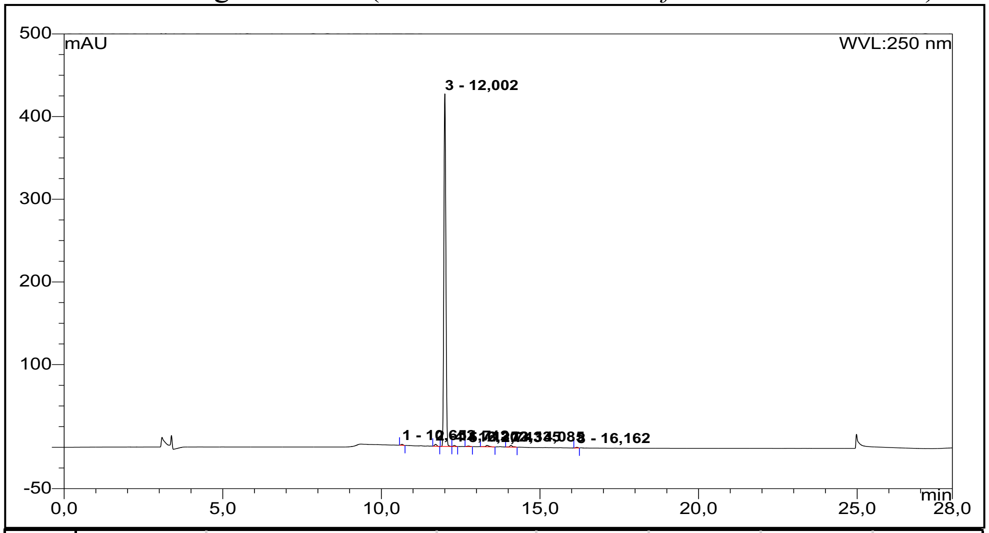

No.	Ret.Time min	Peak Name	Height mAU	Area mAU*min	Rel.Area %	Amount	Type
1	10,65	n.a.	1,214	0,081	0,25	n.a.	BMB
2	11,71	n.a.	2,363	0,197	0,61	n.a.	BMB
3	12,00	n.a.	426,477	31,563	97,12	n.a.	BM
4	12,30	n.a.	1,305	0,106	0,33	n.a.	MB
5	12,74	n.a.	0,782	0,079	0,24	n.a.	BMB
6	13,34	n.a.	1,662	0,199	0,61	n.a.	BMB
7	14,09	n.a.	2,093	0,211	0,65	n.a.	BMB
8	16,16	n.a.	0,817	0,065	0,20	n.a.	BMB
Total:			436,713	32,500	100,00	0,000	

HPLC chromatogram of **6d**. (*Thermo Fisher Scientific* UltiMate^{TM} 3000).

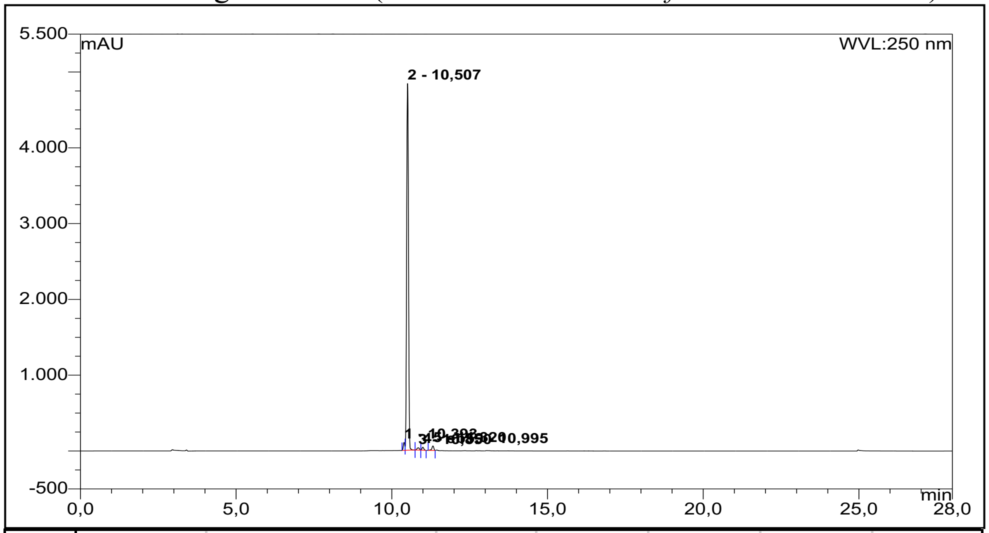

No.	Ret.Time min	Peak Name	Height mAU	Area mAU*min	Rel.Area %	Amount	Type
1	10,39	n.a.	105,704	5,699	1,64	n.a.	BM *
2	10,51	n.a.	#######	332,688	95,60	n.a.	Mb*
3	10,85	n.a.	30,107	2,723	0,78	n.a.	bMB
4	11,00	eb59	39,995	2,669	0,77	n.a.	BMB
5	11,32	n.a.	59,093	4,218	1,21	n.a.	BMB*
Total:			#######	347,998	100,00	0,000	

HPLC chromatogram of **6e**. (*Thermo Fisher Scientific* UltiMate™ 3000).

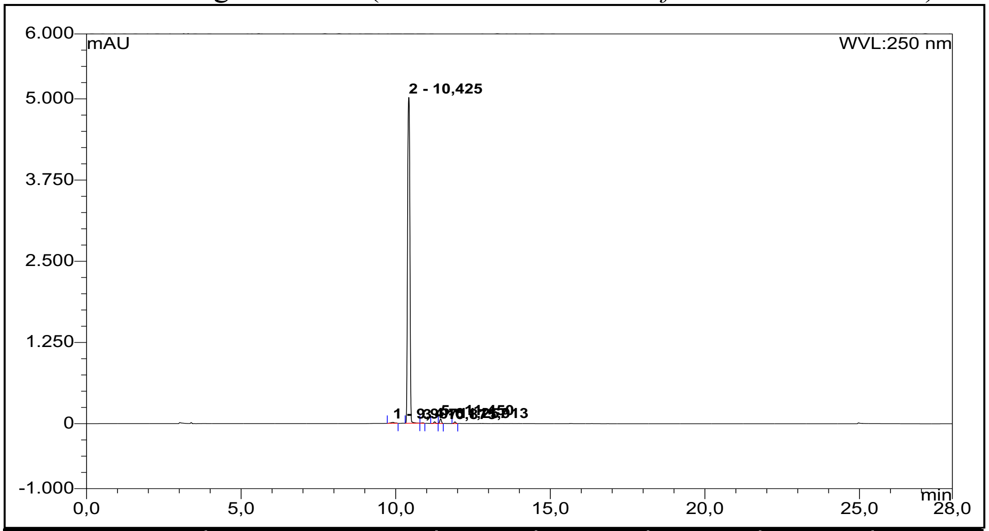

No.	Ret.Time	Peak Name	Height	Area	Rel.Area	Amount	Type
	min		mAU	mAU*min	%		
1	9,91	n.a.	15,339	2,096	0,47	n.a.	BMB*
2	10,43	n.a.	#######	436,027	97,70	n.a.	BMB*
3	10,88	n.a.	6,874	0,548	0,12	n.a.	BMB
4	11,26	n.a.	25,179	1,583	0,35	n.a.	BMB
5	11,45	n.a.	71,702	4,345	0,97	n.a.	BMB
6	11,91	n.a.	25,318	1,679	0,38	n.a.	BMB
Total:			#######	446,276	100,00	0,000	

HPLC chromatogram of **6f**. (*Thermo Fisher Scientific* UltiMate™ 3000).

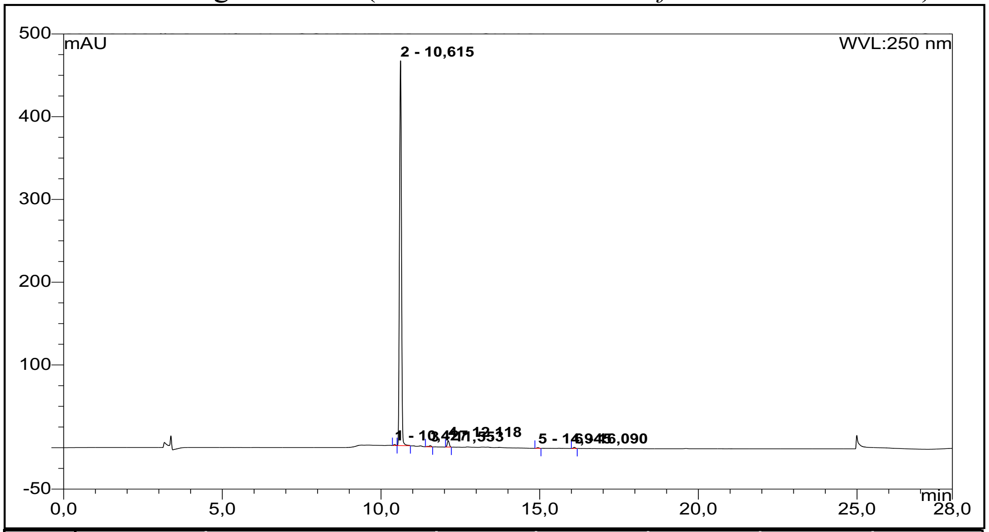

No.	Ret.Time	Peak Name	Height	Area	Rel.Area	Amount	Type
	min		mAU	mAU*min	%		
1	10,43	n.a.	1,289	0,091	0,26	n.a.	BMB*
2	10,62	n.a.	465,009	33,538	97,30	n.a.	BMB*
3	11,55	n.a.	1,464	0,129	0,37	n.a.	BMB*
4	12,12	n.a.	7,679	0,552	1,60	n.a.	BMB*
5	14,95	n.a.	0,820	0,059	0,17	n.a.	BMB
6	16,09	n.a.	1,337	0,100	0,29	n.a.	BMB
Total:			477,599	34,470	100,00	0,000	

HPLC chromatogram of **6g**. (*Thermo Fisher Scientific* UltiMate[TM] 3000).

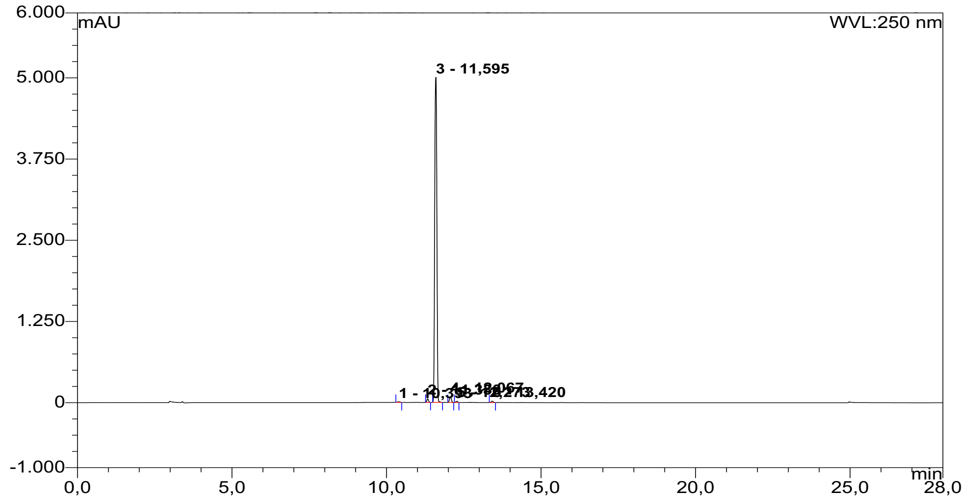

No.	Ret.Time	Peak Name	Height	Area	Rel.Area	Amount	Type
	min		mAU	mAU*min	%		
1	10,39	n.a.	8,414	0,900	0,22	n.a.	BMB*
2	11,33	n.a.	55,088	3,363	0,80	n.a.	BMB*
3	11,60	n.a.	#######	404,694	96,83	n.a.	BMB*
4	12,07	n.a.	87,760	6,163	1,47	n.a.	BMB
5	12,27	n.a.	19,125	1,280	0,31	n.a.	BMB*
6	13,42	n.a.	22,115	1,558	0,37	n.a.	BMB
Total:			#######	417,958	100,00	0,000	

HPLC chromatogram of **6h**. (*Thermo Fisher Scientific* UltiMate[TM] 3000).

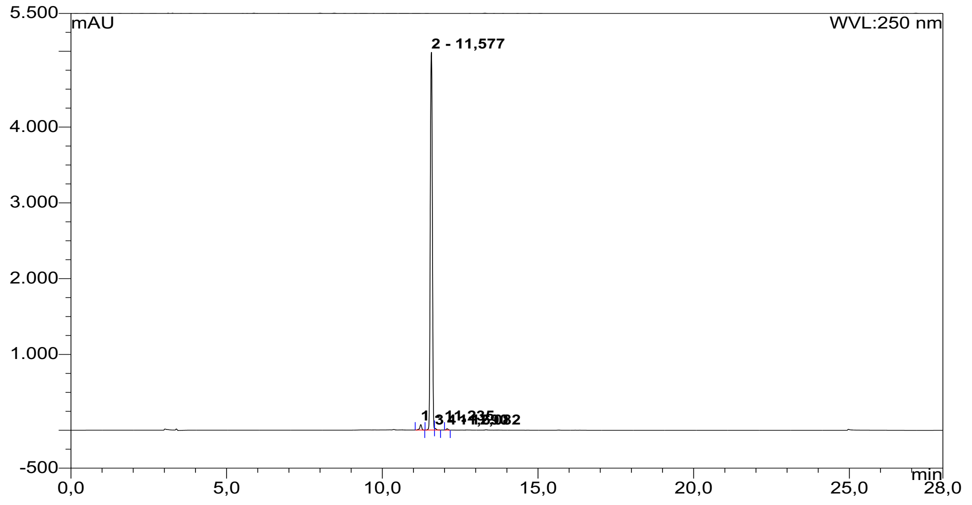

No.	Ret.Time	Peak Name	Height	Area	Rel.Area	Amount	Type
	min		mAU	mAU*min	%		
1	11,24	n.a.	72,461	5,187	1,31	n.a.	BMB
2	11,58	n.a.	#######	387,452	98,02	n.a.	BM
3	11,69	n.a.	22,978	1,376	0,35	n.a.	MB
4	12,08	n.a.	17,774	1,256	0,32	n.a.	BMB
Total:			#######	395,271	100,00	0,000	

HPLC chromatogram of **6i**. (*Thermo Fisher Scientific* UltiMate^{TM} 3000).

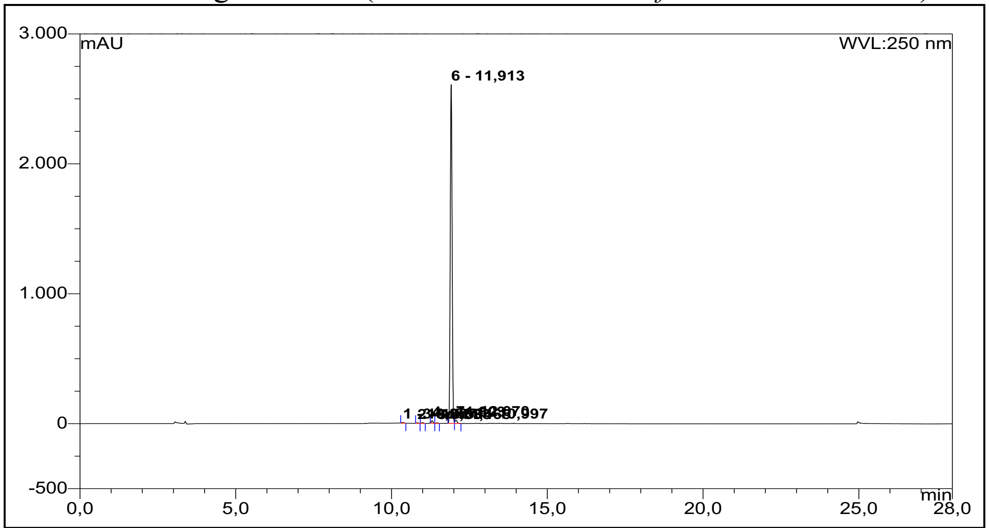

No.	Ret.Time min	Peak Name	Height mAU	Area mAU*min	Rel.Area %	Amount	Type
1	10,37	n.a.	5,366	0,386	0,21	n.a.	BMB*
2	10,84	n.a.	3,683	0,232	0,12	n.a.	BMB*
3	11,00	eb59	4,867	0,303	0,16	n.a.	BMB
4	11,30	n.a.	20,195	1,204	0,65	n.a.	BM *
5	11,47	n.a.	3,535	0,232	0,12	n.a.	MB*
6	11,91	n.a.	#######	181,550	97,74	n.a.	BM
7	12,07	n.a.	24,752	1,837	0,99	n.a.	MB
Total:			#######	185,744	100,00	0,000	

HPLC chromatogram of **10a**. (*Thermo Fisher Scientific* UltiMate^{TM} 3000).

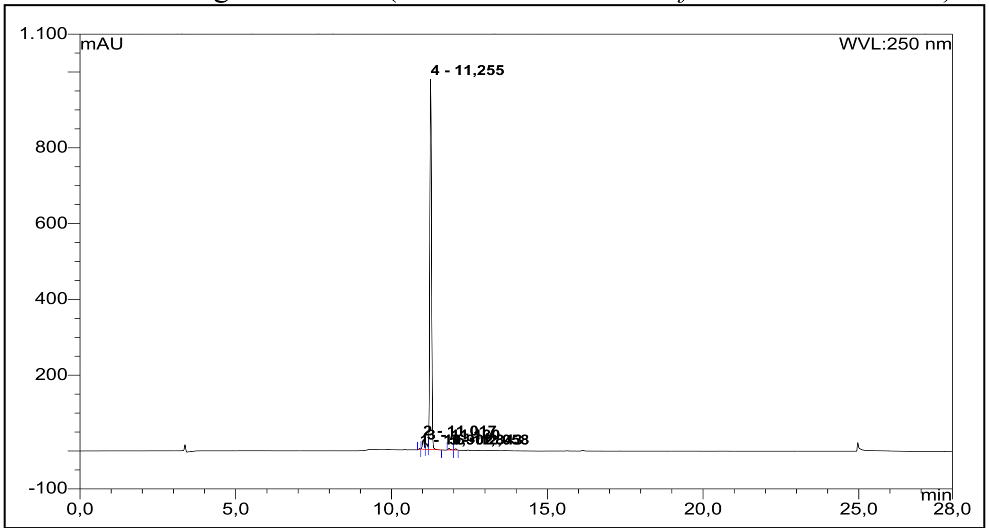

No.	Ret.Time min	Peak Name	Height mAU	Area mAU*min	Rel.Area %	Amount	Type
1	10,90	n.a.	2,292	0,118	0,18	n.a.	BMB*
2	11,02	n.a.	25,283	1,594	2,38	n.a.	BM *
3	11,13	n.a.	15,218	0,976	1,46	n.a.	M *
4	11,26	n.a.	977,066	63,808	95,15	n.a.	MB*
5	11,84	n.a.	3,761	0,324	0,48	n.a.	BMb*
6	12,06	n.a.	3,370	0,243	0,36	n.a.	bMB*
Total:			#######	67,064	100,00	0,000	

HPLC chromatogram of **10b**. (*Thermo Fisher Scientific* UltiMate^{TM} 3000).

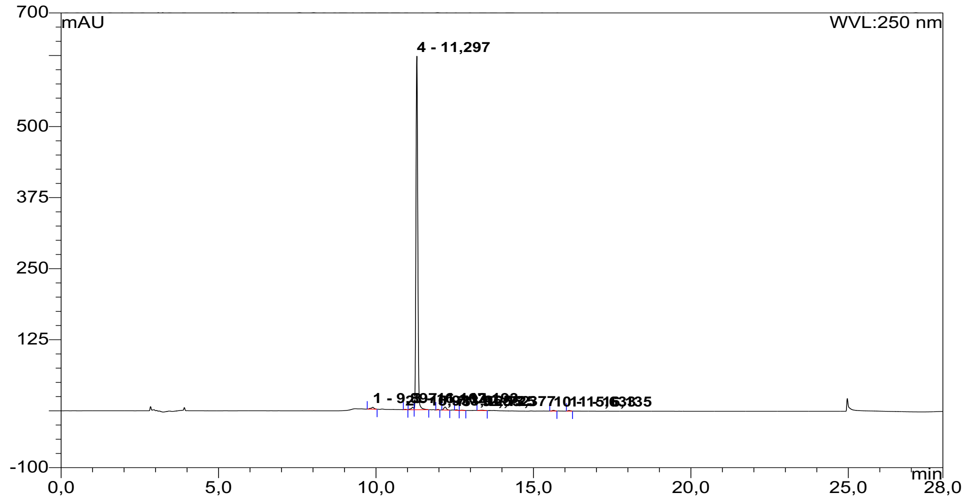

No.	Ret.Time min	Peak Name	Height mAU	Area mAU*min	Rel.Area %	Amount	Type
1	9,90	n.a.	3,332	0,386	0,88	n.a.	BMB*
2	10,93	n.a.	0,760	0,051	0,12	n.a.	BMB
3	11,17	n.a.	4,561	0,405	0,93	n.a.	BM *
4	11,30	n.a.	621,934	41,872	95,92	n.a.	MB*
5	11,97	n.a.	0,807	0,053	0,12	n.a.	BMB*
6	12,19	n.a.	5,264	0,496	1,14	n.a.	BMB
7	12,55	n.a.	0,866	0,068	0,16	n.a.	MB*
8	12,73	n.a.	0,493	0,055	0,13	n.a.	BMB
9	13,38	n.a.	0,630	0,083	0,19	n.a.	BMB*
10	15,63	n.a.	1,216	0,099	0,23	n.a.	BMB*
11	16,14	n.a.	0,946	0,085	0,19	n.a.	BMB*
Total:			640,808	43,652	100,00	0,000	

HPLC chromatogram of **10c**. (*Thermo Fisher Scientific* UltiMate™ 3000).

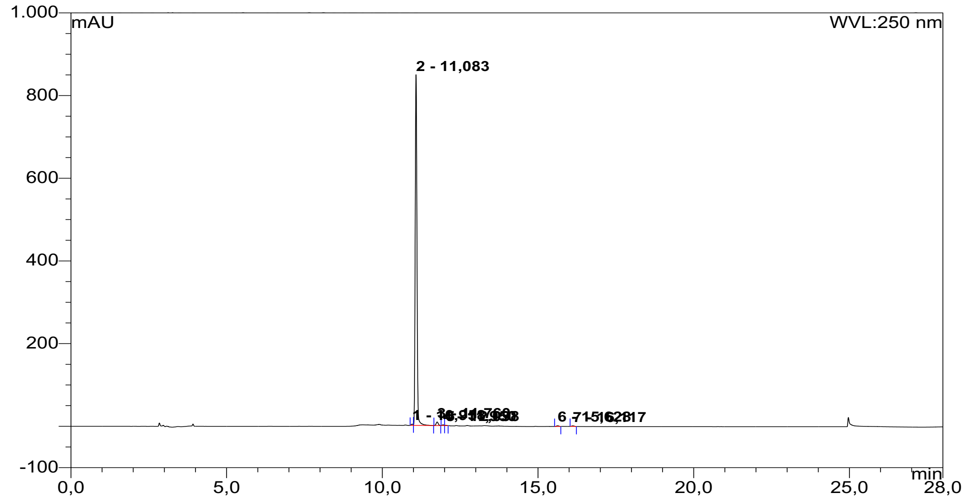

No.	Ret.Time min	Peak Name	Height mAU	Area mAU*min	Rel.Area %	Amount	Type
1	10,96	n.a.	3,031	0,182	0,32	n.a.	BM *
2	11,08	n.a.	848,015	55,935	97,68	n.a.	MB*
3	11,76	n.a.	8,716	0,691	1,21	n.a.	BMB
4	11,95	n.a.	2,004	0,128	0,22	n.a.	BM *
5	12,04	n.a.	0,872	0,060	0,10	n.a.	MB*
6	15,63	n.a.	1,666	0,131	0,23	n.a.	BMB
7	16,12	n.a.	1,475	0,134	0,23	n.a.	BMB*
Total:			865,780	57,262	100,00	0,000	

REFERENCES

[1] B. E. L. Lauffer, R. Mintzer, R. Fong, S. Mukund, C. Tam, I. Zilberleyb, B. Flicke, A. Ritscher, G. Fedorowicz, R. Vallero, D. F. Ortwine, J. Gunzner, Z. Modrusan, L. Neumann, C. M. Koth, P. J. Lupardus, J. S. Kaminker, C. E. Heise, P. Steiner, *J. Biol. Chem.* **2013**, *288*, 26926–26943.

[2] P. J. Watson, C. J. Millard, A. M. Riley, N. S. Robertson, L. C. Wright, H. Y. Godage, S. M. Cowley, A. G. Jamieson, B. V. L. Potter, J. W. R. Schwabe, *Nat. Commun.* **2016**, 11262.

[3] J. S. Laursen, J. Engel-Andreasen, P. Fristrup, P. Harris, C. A. Olsen, *J. Am. Chem. Soc.* **2013**, *135*, 2835–2844.

[4] V. Krieger, A. Hamacher, C. G. W. Gertzen, J. Senger, M. R. H. Zwinderman, M. Marek, C. Romier, F. J. Dekker, T. Kurz, M. Jung, H. Gohlke, M. U. Kassack, F. K. Hansen, *J. Med. Chem.* **2017**, *60*, 5493–5506.

[5] D. Reynolds, M.-H. Hao, J. Wang, S. Prajapati, T. Satoh, A. Selvaraj, *Pyrimidine FGFR4 Inhibitors*, **2015**, WO2015/57938 A1.

[6] S. Dallavalle, R. Cincinelli, R. Nannei, L. Merlini, G. Morini, S. Penco, C. Pisano, L. Vesci, M. Barbarino, V. Zuco, M. De Cesare, F. Zunino, *Eur. J. Med. Chem.* **2009**, *44*, 1900–1912.

[7] S. L. Müller, J. A. Schreiber, D. Schepmann, N. Strutz-Seebohm, G. Seebohm, B. Wünsch, *Eur. J. Med. Chem.* **2017**, *129*, 124–134.

[8] V. Krieger, A. Hamacher, F. Cao, K. Stenzel, C. G. W. Gertzen, L. Schäker-Hübner, T. Kurz, H. Gohlke, F. J. Dekker, M. U. Kassack, F. K. Hansen, *J. Med. Chem.* **2019**, *62*, 11260–11279.

[9] D. Diedrich, A. Hamacher, C. G. W. Gertzen, L. A. Alves Avelar, G. J. Reiss, T. Kurz, H. Gohlke, M. U. Kassack, F. K. Hansen, *Chem. Commun.* **2016**, *52*, 3219–3222.

[10] J. Ji, T. Li, Y. Wang, *Preparation of 3-Quinuclidinyl Amino-Substituted Biaryl Derivatives*, **2005**, US 2005/0159597 A1.

[11] F. Duval, T. A. Van Beek, H. Zuilhof, *Synlett* **2012**, *23*, 1751–1754.

[12] R. Raudszus, R. Nowotny, C. G. W. Gertzen, A. Schöler, A. Krizsan, I. Gockel, H. Kalwa, H. Gohlke, R. Thieme, F. K. Hansen, *Bioorganic Med. Chem.* **2019**, *27*, 115039.

[13] B. Heltweg, F. Dequiedt, E. Verdin, M. Jung, *Anal. Biochem.* **2003**, *319*, 42–48.

[14] J. F. Morrison, C. T. Walsh, in *Adv. Enzymol. Relat. Areas Mol. Biol.*, **1988**, pp. 201–301.

[15] B. Kitir, A. R. Maolanon, R. G. Ohm, A. R. Colaço, P. Fristrup, A. S. Madsen, C. A. Olsen, *Biochemistry* **2017**, *56*, 5134–5146.

[16] J. de Bruijn, F. Busser, W. Seinen, J. Hermens, *Environ. Toxicol. Chem.* **1989**, *8*, 499–512.

[17] OECD, *Test No. 123 Partition Coefficient (1-Octanol/Water): Slow-Stirring Method*, OECD Publishing, Paris, **2006**.

[18] OECD, *Test No. 105: Water Solubility*, OECD Publishing, Paris, **1995**.

[19] L. G. Nivo, R. Moretti, D. Baker, *PLoS One* **2013**, *8*, e59004.

[20] S. Kothiwale, J. L. Mendenhall, J. Meiler, *J. Cheminform.* **2015**, *7*, 1–15.

[21] S. DeLuca, K. Khar, J. Meiler, *PLoS One* **2015**, *10*, e0132508.

[22] K. W. Kaufmann, J. Meiler, *PLoS One* **2012**, *7*, e50769.

[23] J. Meiler, D. Baker, *Proteins* **2006**, *65*, 538–548.
